# USP4‐Dependent CHAF1B Stabilization Regulates Distinct SETDB1 Ubiquitin States Linked to AKT T308 Signaling and Lipogenic Remodeling in HCC

**DOI:** 10.1002/advs.78039

**Published:** 2026-09-29

**Authors:** Saiyan Bian, Yun Tong, Wenkai Ni, Zhangzhi Tang, Weiting Chen, Kexin Ma, Lihan Jiang, Jiayu Shao, Xuyang He, Wenjie Zheng

**Affiliations:** ^1^ Department of Oncology & Department of Clinical and Translational Research Center, Clinical Trials Center Affiliated Hospital of Nantong University, Medical School of Nantong University Nantong China; ^2^ Department of Gastroenterology & Research Center of Clinical Medicine Affiliated Hospital of Nantong University, Medical School of Nantong University Nantong China; ^3^ Department of Pathology Medical School of Nantong University Nantong China

**Keywords:** AKT signaling, ASD, CHAF1B, HCC, SETDB1, ubiquitination, UHRF1, USP4, VHL

## Abstract

Durable responses to current therapies remain limited in hepatocellular carcinoma (HCC), highlighting the need to identify regulators of malignant progression. By integrating multi‐omics analyses, spatial transcriptomics, clinical specimens, and multiple models, we identified chromatin assembly factor 1B (CHAF1B) as a functional regulator of HCC phenotypes. Gain‐ and loss‐of‐function of CHAF1B altered proliferative, migratory, clonogenic, and tumorigenic phenotypes. LC–MS/MS, DIA proteomics, and cell‐based assays revealed CHAF1B‐associated lipogenic remodeling characterized by SREBP1C nuclear localization, lipogenic gene/protein induction, and lipid‐droplet accumulation. Mechanistically, the WD40 repeat‐containing region of CHAF1B contributed to its association with UHRF1 and SETDB1, supporting UHRF1‐associated K63‐linked ubiquitination and CRM1/exportin‐1‐dependent cytoplasmic redistribution of SETDB1. Conversely, CHAF1B depletion enhanced SETDB1 association with VHL and favored a predominantly K11‐associated degradative ubiquitin state linked to proteasomal SETDB1 loss. SETDB1 redistribution and catalytic activity were associated with AKT T308‐linked signaling. A focused CRISPR‐based screen of deubiquitinases identified USP4 as an upstream regulator of CHAF1B protein homeostasis. USP4 depletion or Akebia saponin D (ASD) increased K48‐linked ubiquitination of CHAF1B, reduced CHAF1B protein abundance, attenuated AKT T308‐linked signaling, and suppressed malignant and lipogenic phenotypes. These findings reveal distinct ubiquitin‐dependent states governing SETDB1 stability and identify USP4‐dependent CHAF1B stabilization as an upstream regulatory node in HCC.

## Graphical abstract

USP4 stabilizes CHAF1B by limiting K48‐linked ubiquitination. Stabilized CHAF1B supports UHRF1‐associated K63‐linked SETDB1 ubiquitination and cytoplasmic redistribution, whereas CHAF1B loss favors VHL‐dependent K11‐associated degradative ubiquitination and proteasomal loss of SETDB1. These distinct ubiquitin states link SETDB1 to AKT T308 signaling, SREBP1C‐associated lipogenic remodeling, and aggressive HCC phenotypes, while ASD perturbs the USP4–CHAF1B axis.

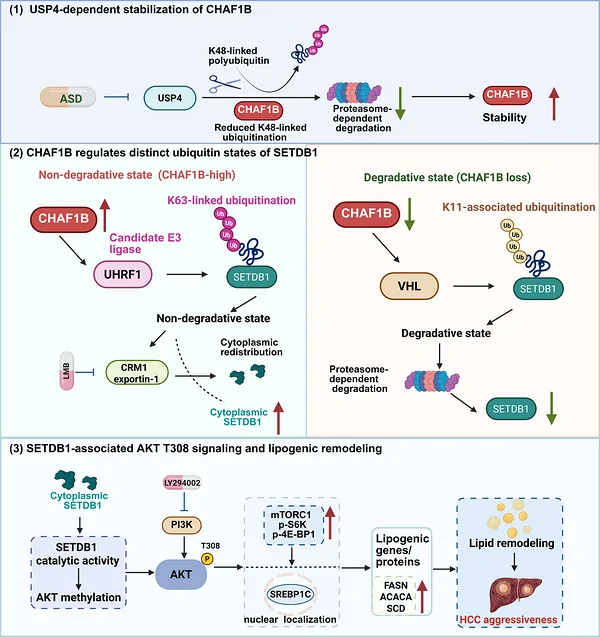

**Abbreviations**
AKT protein kinase B: protein kinase B
AML: acute myeloid leukemia
ASD: Akebia saponin D
ASF1: anti‐silencing function 1
CAF‐1: chromatin assembly factor‐1
CETSA: cellular thermal shift assay
CHAF1A: chromatin assembly factor 1A
CHAF1B: chromatin assembly factor 1B
CHX: cycloheximide
CRM1: chromosome region maintenance 1 (exportin 1)
DIA: data‐independent acquisition
DUB: deubiquitinase
GSEA: gene set enrichment analysis
HBV: hepatitis B virus
HCC: hepatocellular carcinoma
HCV: hepatitis C virus
HDAC: histone deacetylase
IHC: immunohistochemistry
LC–MS/MS: liquid chromatography–tandem mass spectrometry
mIF: multiplex immunofluorescence
MRI: magnetic resonance imaging
mTORC1: mechanistic target of rapamycin complex 1
NuRD: nucleosome remodeling and deacetylase
PCNA: proliferating cell nuclear antigen
PLA: proximity ligation assay
RBBP4: retinoblastoma‐binding protein 4
RT–qPCR: reverse transcription quantitative polymerase chain reaction
SETDB1: SET domain bifurcated histone lysine methyltransferase 1
SREBP1C: sterol regulatory element‐binding protein 1C
ssDNA: single‐stranded DNA
TACE: transarterial chemoembolization
UHRF1: ubiquitin‐like with PHD and RING finger domains 1
USP4: ubiquitin‐specific peptidase 4
UV: ultraviolet

## 1 Introduction

Hepatocellular carcinoma (HCC), the predominant primary liver cancer, accounts for approximately 80%–90% of cases [1]. It imposes a major global burden, ranking sixth worldwide in incidence and third in cancer‐related mortality, with particularly high prevalence in regions endemic for hepatitis B virus (HBV) and hepatitis C virus (HCV), notably China, which contributes nearly half of new liver cancer cases annually [2, 3, 4]. Because early‐stage HCC is often asymptomatic, most patients present at intermediate or advanced stages, when curative resection or liver transplantation is not feasible for the majority. Locoregional therapies such as ablation and transarterial chemoembolization (TACE) provide palliation but seldom achieve durable control. Systemic multikinase inhibitors such as sorafenib and lenvatinib offer modest benefit, yet resistance commonly emerges [5]. Immune checkpoint inhibitors are promising, but monotherapy response rates remain limited [6]. These therapeutic constraints underscore the need to identify the molecular regulators of HCC and clinically relevant targets for more effective and durable interventions [7].

Chromatin assembly factor 1B (CHAF1B) is the p60 subunit of the trimeric chromatin assembly factor‐1 (CAF‐1) complex together with CHAF1A (p150) and RBBP4 (p48) [8]. CAF‐1 mediates replication‐coupled nucleosome assembly by depositing histone H3–H4 onto nascent DNA during S phase [9]. CHAF1A targets the complex to replication forks through its interaction with proliferating cell nuclear antigen (PCNA). CHAF1B receives H3–H4 from the upstream chaperone ASF1 and coordinates transfer to DNA, stabilizing the assembly intermediate [10]. RBBP4, a WD40‐repeat protein, binds histone H4 and is shared with other chromatin‐regulatory complexes such as NuRD and HDAC complexes, thereby functionally connecting CAF‐1 to broader epigenetic regulation [11, 12].

Beyond its role in replication‐coupled nucleosome assembly, CHAF1B has emerged as a regulator of cell identity, transcriptional programs, and DNA repair. In acute myeloid leukemia (AML), CHAF1B overexpression constrains differentiation by displacing lineage‐defining transcription factors from promoters and enhancers, whereas genetic depletion relieves this block and suppresses leukemogenesis in vitro and in vivo [13, 14]. Elevated CHAF1B (CAF‐1/p60) has also been documented in several solid tumors and associates with aggressive behavior and poor outcome, including high‐grade glioma and melanoma [15, 16], with similar observations reported in cervical and lung squamous‐cell carcinoma [17, 18]. Under genotoxic stress, the CAF‐1 pathway contributes to therapy tolerance by maintaining chromatin integrity and managing ssDNA gaps, thereby modulating responses to DNA‐damaging agents [19]. Consistent with a role in genome maintenance, CAF‐1 participates in chromatin restoration after nucleotide excision repair of UV‐induced lesions in human cells [20, 21]. Despite these links to oncogenic phenotypes, the biological significance of CHAF1B in hepatocellular carcinoma remains incompletely defined.

This study examines CHAF1B in HCC using multi‐omics, spatial transcriptomics, clinical specimens, and experimental models to evaluate its clinical association, functional relevance, and signaling context. In parallel, we sought upstream regulatory mechanisms and pharmacologic perturbation, prioritizing the deubiquitinase USP4 and evaluating the natural product Akebia saponin D as a probe compound. Together, this framework enables evaluation of CHAF1B‐associated signaling and USP4‐dependent CHAF1B regulation in HCC and provides a basis for further mechanistic and therapeutic investigation.

## 2 Results

### 2.1 Identification of CHAF1B as a Candidate Regulator of HCC

To nominate candidate HCC‐associated genes, we conducted a cross‐cohort screen integrating five independent GEO datasets. Differential expression analyses identified broad sets of genes that were consistently upregulated in tumors (Figure S1A). Intersecting the upregulated gene lists yielded a stringent 44‐gene consensus module that remained elevated across all cohorts (Figure S1B). Validation in TCGA at the RNA level and in TCPA at the protein level then prioritized candidates that were both overexpressed in tumors and associated with poor outcome, narrowing the list to 11 genes (Figure S1C). Within this 11‐gene panel, CHAF1B showed top‐three Pearson correlations with both the proliferation marker PCNA and the invasion marker MMP9 in TCGA, aligning it with key hallmarks of tumor aggressiveness (Figure S1D). Spatial transcriptomics further mapped CHAF1B enrichment to malignant regions compared with adjacent liver, and cell–cell communication analysis linked CHAF1B‐high neighborhoods to active tumor programs (Figure S1E). Independent validation in 24 paired clinical samples confirmed higher CHAF1B at both protein and mRNA levels in tumors relative to matched non‐tumor liver (Figure S1F). Immunohistochemistry in representative pairs showed strong CHAF1B staining in tumor tissue with minimal signal in adjacent liver (Figure S1G). Collectively, these results identify CHAF1B as a candidate regulator for further functional investigation in HCC.

### 2.2 CHAF1B Contributes to Malignant Phenotypes in HCC Models

We then profiled baseline CHAF1B expression in HCC cell lines and established gain‐/loss‐of‐function models. Immunoblotting showed variable endogenous CHAF1B across HCC cell lines (Figure S2A), with RT–qPCR confirming concordant mRNA levels (Figure S2B). To further address whether endogenous CHAF1B abundance is associated with intrinsic malignant potential, we directly compared the five HCC cell lines used in the baseline expression analysis. Cell lines with relatively higher endogenous CHAF1B expression generally exhibited greater lipid‐droplet accumulation, proliferative activity, and clonogenic capacity than cell lines with lower CHAF1B expression (Figure S2C–E). These cross‐cell‐line observations provide complementary association evidence, whereas reciprocal gain‐ and loss‐of‐function experiments were subsequently used to support a functional contribution of CHAF1B.

We then achieved efficient knockdown in Huh7 and SK‐Hep1 and stable overexpression in HepG2 and MHCC97H, verified at both protein and transcript levels (Figure S2F,G). We next systematically evaluated the effects of CHAF1B on cellular proliferation, clonogenicity, migration, and tumorigenic potential of HCC cells. CCK‐8 assays demonstrated that CHAF1B knockdown significantly attenuated proliferation in Huh7 and SK‐Hep1 cells, whereas ectopic overexpression markedly enhanced growth in HepG2 and MHCC97H cells (Figure 1A). These findings were corroborated by EdU staining assays, which showed decreased or increased EdU‐positive fractions upon CHAF1B silencing or overexpression (Figure 1B). Consistently, colony formation assays showed that CHAF1B depletion reduced, whereas CHAF1B overexpression increased, clonogenic growth (Figure 1C). In parallel, 3D spheroid assays showed that CHAF1B knockdown suppressed spheroid expansion in Huh7 cells, whereas CHAF1B overexpression enhanced spheroid growth in HepG2 cells. Further immunofluorescence staining for Ki‐67 confirmed increased proliferative indices in CHAF1B‐overexpressing spheroids (Figure 1D). Transwell assays further showed that CHAF1B depletion reduced, whereas CHAF1B overexpression increased, HCC cell migration (Figure 1E). To further investigate the effects of CHAF1B loss on HCC, we performed DIA‐based quantitative proteomics in Huh7 cells after CHAF1B knockdown and subjected the differential proteins to GSEA. In the shNC‐versus‐shCHAF1B ranked comparison, the epithelial cell differentiation gene set showed significant negative enrichment (NES = −1.852, P = 0.0059), indicating relative enrichment of epithelial differentiation‐associated proteins in CHAF1B‐depleted cells (Figure 1F). Phase‐contrast imaging showed that CHAF1B overexpression induced a fibroblast‐like, spindle‐shaped morphology, whereas CHAF1B‐depleted cells displayed a more compact epithelial‐like morphology (Figure 1G). Consistently, CHAF1B modulation altered E‐cadherin, N‐cadherin, and Vimentin expression in a pattern associated with EMT‐like changes (Figure 1H).

**FIGURE 1:**
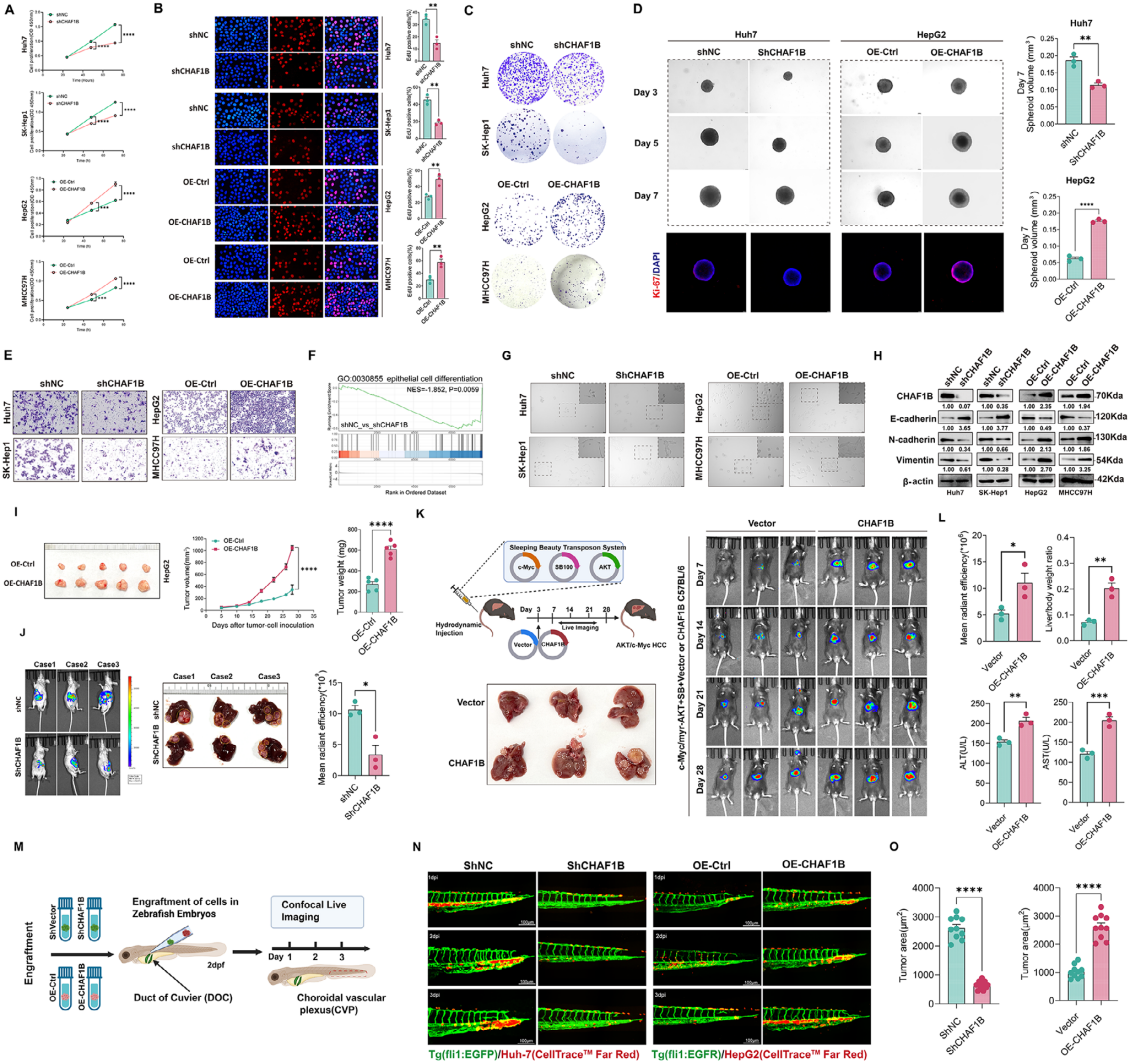
CHAF1B contributes to aggressive phenotypes in HCC models. (A) Cell proliferation assessed by CCK‐8 assay in Huh7, SK‐Hep1, HepG2, and MHCC97H cells following CHAF1B knockdown or overexpression. (B) Representative EdU immunofluorescence images and quantification showing changes in proliferation of HCC cells in response to CHAF1B silencing or overexpression. (C) Colony formation assay demonstrating altered clonogenicity upon CHAF1B knockdown or overexpression. (D) 3D spheroid growth in Huh7 and HepG2 cells upon CHAF1B modulation. Ki‐67 immunofluorescence indicates enhanced proliferative capacity in CHAF1B‐overexpressing spheroids. Day 7 spheroid volume was calculated from three independently cultured spheroids per group. Data are presented as mean ± SD; each data point represents one spheroid. (E) Transwell migration assays showing increased migratory capacity in CHAF1B‐overexpressing cells and impaired motility in knockdown cells. (F) DIA‐based quantitative proteomics of Huh7 cells comparing shCHAF1B with shNC followed by GSEA shows negative enrichment of the epithelial cell differentiation gene set GO:0030855 (NES = −1.852, *p* = 0.0059). (G) Phase‐contrast micrographs illustrating CHAF1B‐associated changes between spindle‐shaped and epithelial‐like cellular morphologies. (H) Immunoblot analysis of EMT‐associated markers following CHAF1B overexpression or silencing. Relative band intensities are shown below the corresponding lanes. Protein signals were normalized to β‐actin, and the corresponding control group within each cell line was set to 1.00. (I) Subcutaneous xenograft tumors derived from HepG2 cells overexpressing CHAF1B or vector control. Longitudinal tumor volume and endpoint tumor weight are shown. (J) Orthotopic liver‐tumor model using luciferase‐labeled Huh7 cells with stable CHAF1B knockdown or control vector. Bioluminescence imaging, endpoint quantification of mean radiant efficiency, and gross liver examination at sacrifice showed reduced intrahepatic tumor burden following CHAF1B depletion. (K and L) Sleeping Beauty (SB)‐based hydrodynamic injection model. CHAF1B cooperates with c‐Myc/myr‐AKT to increase bioluminescent signal, liver tumor burden, liver‐to‐body‐weight ratio, and serum ALT and AST levels. Serial bioluminescence imaging showed increased tumor burden in the CHAF1B co‐expression group, with endpoint mean radiant efficiency quantified as indicated. (M) Schematic illustration of the zebrafish xenograft procedure. (N) Representative fluorescence images of Tg(fli1:EGFP) embryos injected with CellTrace Far Red‐labeled Huh7 cells carrying shNC or shCHAF1B, or HepG2 cells carrying OE‐Ctrl or OE‐CHAF1B constructs, at the indicated days post‐injection. (O) Quantification of fluorescent tumor area in the indicated groups. Each data point represents one embryo. Mouse body weight was monitored longitudinally throughout the corresponding experimental periods. Longitudinal body‐weight data are summarized in Figure S17 as mean ± SD, and the corresponding individual‐animal measurements and baseline‐to‐endpoint changes are provided in Data S1. ****, *p* < 0.0001; ***, *p* < 0.001; **, *p* < 0.01; *, *p* < 0.05. For quantitative cell‐based assays, *n* = 3 independent experiments unless otherwise indicated. For the spheroid analysis in (D), *n* = 3 independently cultured spheroids per group. For the subcutaneous xenograft in (I), *n* = 5 mice per group; for the orthotopic and Sleeping Beauty models in (J–L), *n* = 3 mice per group; and for the zebrafish analysis in (O), *n* = 10 embryos per group. Data are presented as mean ± SD. Two‐group endpoint comparisons were analyzed using unpaired two‐sided Student's t‐tests. Longitudinal tumor‐volume data in (I) were analyzed using two‐way repeated‐measures ANOVA followed by Šídák's multiple‐comparisons test.

To further validate the tumor‐promoting role of CHAF1B, we employed four independent in vivo models. Subcutaneous xenograft assays suggested that CHAF1B‐overexpressing tumors exhibited increased tumor growth and endpoint tumor mass compared to control xenografts (Figure 1I). In the orthotopic liver implantation model, Huh7 cells with stable CHAF1B knockdown or control vector were directly implanted into the left hepatic lobe of immunodeficient mice. Bioluminescence imaging and endpoint liver examination showed reduced intrahepatic tumor burden and bioluminescent signal in the CHAF1B‐depleted group (Figure 1J). Next, the hydrodynamic tail vein injection (HTVI) model combined with the Sleeping Beauty (SB) transposon system was used to assess whether CHAF1B cooperates with a c‐Myc/myr‐AKT‐driven oncogenic background in vivo. Co‐expression of CHAF1B with c‐Myc and myr‐AKT accelerated tumor formation, as shown by increased bioluminescent signal, liver tumor burden, and liver‐to‐body‐weight ratio compared with vector controls (Figure 1K,L). Additionally, zebrafish xenograft assays were performed by injecting CellTrace Far Red‐labeled Huh7 or HepG2 cells into the duct of Cuvier of Tg(fli1:EGFP) embryos. CHAF1B depletion reduced the fluorescent tumor area of Huh7 xenografts, whereas CHAF1B overexpression increased the tumor area of HepG2 xenografts (Figure 1M–O). Collectively, these results support CHAF1B as a functional contributor to aggressive phenotypes in HCC.

### 2.3 CHAF1B‐Associated AKT/mTOR/SREBP Signaling is Linked to Lipogenic Remodeling and Malignant Phenotypes in HCC cells

To obtain an initial overview of CHAF1B‐associated metabolic changes, we performed untargeted LC–MS/MS metabolomics in CHAF1B‐overexpressing HepG2 cells. Across six samples, 582 metabolites in ESI+ mode and 449 metabolites in ESI− mode were annotated. Using VIP > 1.0, |FC| ≥ 1.2 or ≤ 0.833, and *p* < 0.05 as thresholds, we identified 59 differential metabolites in ESI+ mode (35 upregulated, 24 downregulated) and 30 metabolites in ESI− mode (15 upregulated, 15 downregulated) for the G2_OE vs G2_vec comparison (Figure S3A). Heatmaps and Z‐score plots showed coordinated changes in lipid‐related metabolite classes, including oxylipins/eicosanoids (11‐dehydro‐TXB2, 12‐epi‐LTB4, (+/−)8(9)‐DiHETE, Resolvin E1, 15(R)‐PGD2, 6‐keto‐PGF1α), glycerophospholipid remodeling (PC species, LPC 20:4‐sn1, LysoPE 18:2, ether‐PCs), sphingomyelins (SM 42:2;2O, SM 8:1;2O/34:1), acylcarnitines (CAR 12:1/14:1/18:0/20:3) and mevalonate/bile‐acid intermediates (mevalonic acid, lithocholic acid) (Figure 2A). Chord and correlation analyses further suggested coordinated lipid‐metabolite remodeling upon CHAF1B overexpression (Figure S3B,C).

**FIGURE 2:**
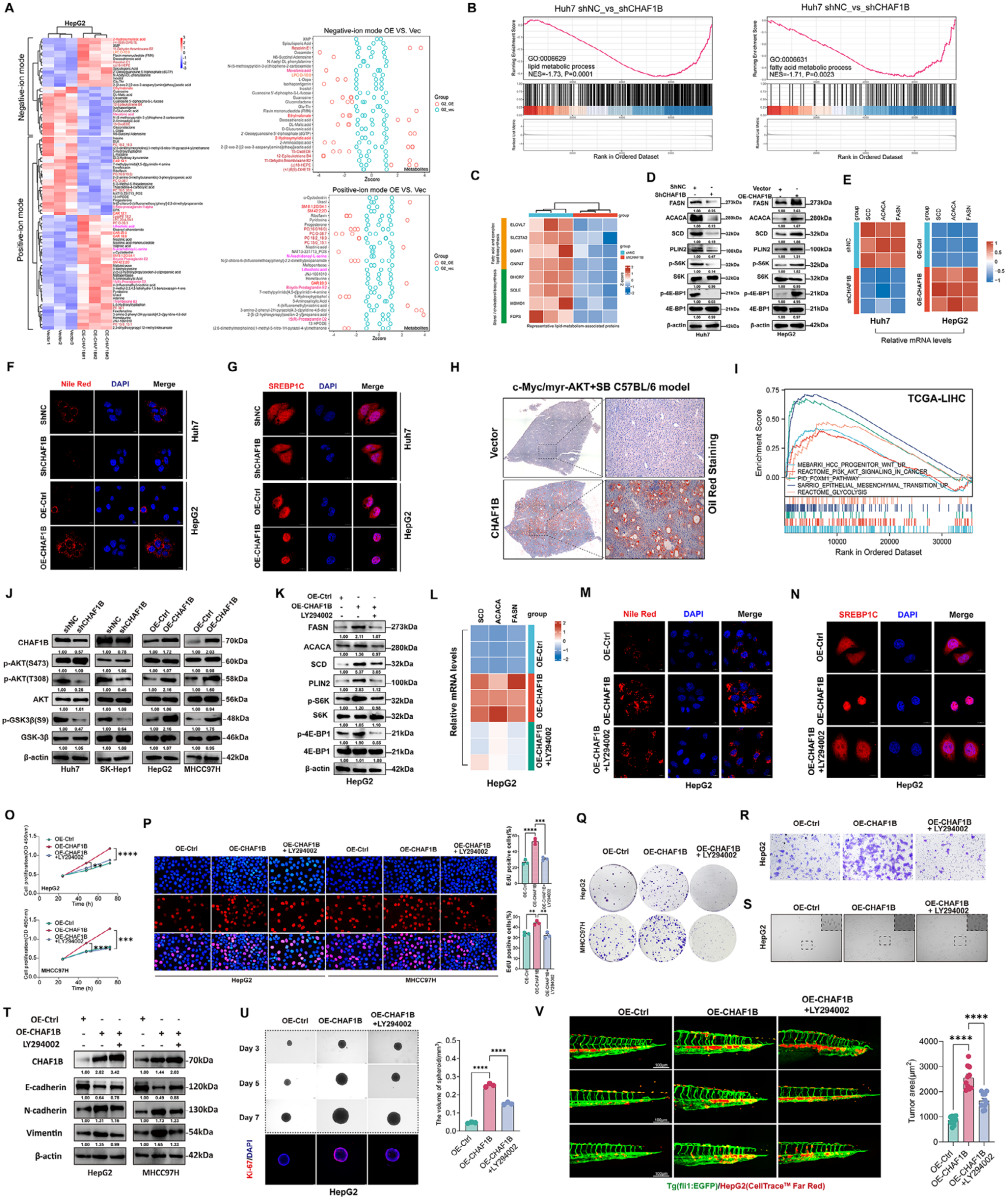
CHAF1B‐associated AKT/mTOR/SREBP signaling contributes to lipogenic remodeling and aggressive phenotypes in HCC cells. (A) Untargeted LC–MS/MS across six samples annotated 582 metabolites (ESI+) and 449 (ESI−). With VIP > 1.0, |FC| ≥ 1.2 (or ≤ 0.833) and *p* < 0.05, we identified 59 differentials in ESI+ (35 up, 24 down) and 30 in ESI‐. Heatmaps and Z‐score plots highlight coordinated shifts in glycerophospholipids, sphingomyelins, oxylipins, acylcarnitines, and mevalonate/bile‐acid intermediates, indicating lipid‐metabolic remodeling with CHAF1B overexpression. (B) GSEA of the shCHAF1B proteome in Huh7 cells shows significant negative enrichment of lipid metabolic process (GO:0006629) and fatty‐acid metabolic process (GO:0006631). (C) Heatmap of representative proteins involved in fatty‐acid, complex‐lipid, and sterol/cholesterol metabolism that were reduced after CHAF1B depletion in Huh7 cells. (D) Immunoblotting in Huh7 and HepG2 cells showing CHAF1B‐associated changes in FASN, ACACA, SCD, PLIN2, and the mTORC1‐associated readouts p‐S6K and p‐4E‐BP1. (E) RT‐qPCR heatmaps showing CHAF1B‐associated changes in FASN, ACACA, and SCD mRNA expression. (F) Nile Red staining showing increased lipid‐droplet accumulation after CHAF1B overexpression and reduced accumulation after CHAF1B depletion. (G) Immunofluorescence showing increased SREBP1C nuclear localization after CHAF1B overexpression and reduced nuclear localization after CHAF1B depletion. (H) Oil Red O staining of liver tumors from the c‐Myc/myr‐AKT Sleeping Beauty model showing increased lipid deposition in the CHAF1B co‐expression group. (I) GSEA of TCGA‐LIHC showing an association between high CHAF1B expression and PI3K/AKT‐related gene sets. (J) Immunoblotting showing that CHAF1B manipulation preferentially alters AKT T308 phosphorylation, with comparatively limited effects on AKT S473 phosphorylation and total AKT. (K) Immunoblotting showing that LY294002 attenuates CHAF1B‐associated AKT/mTORC1 signaling and lipogenic protein expression. (L) RT–qPCR heatmap showing that LY294002 attenuates CHAF1B‐associated increases in FASN, ACACA, and SCD mRNA expression. (M) Nile Red staining showing that LY294002 reduces lipid‐droplet accumulation in CHAF1B‐overexpressing cells. (N) Immunofluorescence showing that LY294002 reduces SREBP1C nuclear localization in CHAF1B‐overexpressing cells. (O) CCK‐8 assays showing that LY294002 attenuates CHAF1B‐associated cell growth. (P) EdU incorporation assays and quantification showing that LY294002 attenuates CHAF1B‐associated proliferative activity. (Q) Colony formation assays showing that LY294002 reduces CHAF1B‐associated clonogenic growth. (R) Transwell assays showing that LY294002 attenuates CHAF1B‐associated cell migration. (S) Phase‐contrast imaging showing that LY294002 attenuates the mesenchymal‐like morphology associated with CHAF1B overexpression. (T) Immunoblotting showing that LY294002 attenuates CHAF1B‐associated changes in E‐cadherin, N‐cadherin, and Vimentin. (U) Three‐dimensional spheroid assays showing that LY294002 reduces CHAF1B‐associated spheroid growth and Ki‐67 positivity. Day 7 spheroid volume was calculated from three independently cultured spheroids per group. Data are presented as mean ± SD; each data point represents one spheroid. (V) Representative fluorescence images and quantification of tumor area in Tg(fli1:EGFP) embryos injected with CellTrace Far Red‐labeled HepG2 cells carrying OE‐Ctrl, OE‐CHAF1B, or OE‐CHAF1B followed by LY294002 treatment. Relative band intensities are shown below the corresponding lanes. All protein signals, including phosphorylated and total proteins, were independently normalized to β‐actin, and the corresponding control group within each cell line was set to 1.00. ****, *p* < 0.0001; ***, *p* < 0.001; **, *p* < 0.01. Metabolomics in (A) and the DIA proteomic dataset underlying (B,C) used n = 3 biological replicates per group. Cell‐based quantitative assays were performed in three independent experiments (*n* = 3); the spheroid analysis in (U) used *n* = 3 spheroids per group and the zebrafish analysis in (V) used n = 10 embryos per group. Data are presented as mean ± SD. Differential‐metabolite analyses used two‐sided Student's *t*‐tests. Two‐group cell‐based comparisons were analyzed using unpaired two‐sided Student's *t*‐tests, whereas comparisons among three groups were analyzed using one‐way ANOVA followed by Tukey's multiple‐comparisons test. GSEA significance was assessed using 1000 permutations.

Because untargeted metabolomics alone provides limited information on the upstream molecular program, we next integrated these data with DIA‐based quantitative proteomics in Huh7 cells after CHAF1B knockdown. GSEA of the proteomic dataset showed negative enrichment of lipid metabolic process (GO:0006629; NES = −1.73, *p* = 0.001) and fatty‐acid metabolic process (GO:0006631; NES = −1.71, *p* = 0.023) in shCHAF1B cells, indicating suppression of lipid‐metabolism‐associated programs after CHAF1B loss (Figure 2B). Consistent with this proteomic trend, representative proteins involved in fatty acid and complex lipid biosynthesis, including SLC27A2, ELOVL7, DGAT1, and GNPAT, as well as sterol/cholesterol biosynthesis, including FDPS, SQLE, MSMO1, and DHCR7, were reduced after CHAF1B knockdown (Figure 2C). Together, these metabolomic and proteomic data suggest that CHAF1B is associated with lipid‐metabolic remodeling in HCC cells.

We then performed targeted molecular and functional validation of this lipogenic phenotype. At the protein level, CHAF1B depletion decreased FASN, ACACA, SCD, PLIN2, and mTORC1‐related readouts, including p‐S6K and p‐4E‐BP1, whereas CHAF1B overexpression increased these proteins (Figure 2D). RT‐qPCR further confirmed CHAF1B‐associated changes in lipogenic genes, including FASN, ACACA, and SCD (Figure 2E). Functionally, CHAF1B enhanced lipid‐droplet accumulation, as shown by Nile Red staining in both Huh7 and HepG2 (Figure 2F), and increased nuclear localization of SREBP1C (Figure 2G). In the c‐Myc/myr‐AKT‐driven murine liver tumor model, CHAF1B also increased Oil Red O‐positive lipid deposition within tumor nodules (Figure 2H). Analysis of TCGA‐LIHC further linked high CHAF1B expression to oncogenic hallmarks, including PI3K/AKT signaling (Figure 2I). Together, these metabolomic, proteomic, molecular, and functional data support CHAF1B‐associated lipogenic remodeling in HCC cells.

Given the close connection between PI3K/AKT/mTORC1 signaling and SREBP1C‐mediated lipogenic regulation, we next assessed whether CHAF1B was associated with altered AKT pathway activity. CHAF1B knockdown preferentially reduced AKT phosphorylation at T308, with comparatively limited effects on total AKT or AKT S473 phosphorylation, whereas CHAF1B overexpression showed the opposite pattern across HCC cell lines (Figure 2J). Pharmacologic inhibition with the PI3K inhibitor LY294002 attenuated CHAF1B‐induced p‐AKT(T308), mTORC1 readouts, and the lipogenic proteins FASN, ACACA, SCD, and PLIN2 (Figure 2K,L and Figure S4A). LY294002 also reduced lipid droplets and SREBP1C nuclear localization (Figure 2M,N), suppressed proliferation (Figure 2O,P), clonogenicity (Figure 2Q), migration and mesenchymal‐like spindle morphology (Figure 2R,S and Figure S4B,C), attenuated CHAF1B‐associated changes in EMT‐related markers (Figure 2T), and limited 3D spheroid growth (Figure 2U and Figure S4D). In Tg(fli1:EGFP) embryos injected with CellTrace Far Red‐labeled HepG2 cells, LY294002 reduced the increase in fluorescent tumor area associated with CHAF1B overexpression (Figure 2V).

To further determine whether SREBP1C‐dependent lipogenic remodeling contributes to CHAF1B‐associated phenotypes, we performed SREBP1C knockdown in CHAF1B‐overexpressing HepG2 cells. SREBP1C knockdown attenuated CHAF1B‐induced increases in the lipogenic proteins FASN, ACACA, SCD, and PLIN2, as well as the mTORC1‐associated readouts p‐S6K and p‐4E‐BP1 (Figure S5A). Consistently, the increased mRNA expression of FASN, ACACA, and SCD was reduced after SREBP1C knockdown (Figure S5B). SREBP1C depletion also decreased CHAF1B‐associated lipid‐droplet accumulation and partially attenuated cell proliferation, clonogenic growth, and migration (Figure S5C–F). These findings support a functional contribution of SREBP1C‐dependent lipogenic remodeling to CHAF1B‐associated malignant phenotypes.

Together, these results suggest that CHAF1B‐associated AKT T308 phosphorylation is linked to enhanced mTORC1 readouts, SREBP1C nuclear localization, and lipogenic marker expression, and that this signaling program contributes to lipogenic remodeling and malignant phenotypes in HCC cells.

### 2.4 SETDB1 Contributes to CHAF1B‐Associated AKT T308 Phosphorylation

To further delineate the mechanistic basis underlying CHAF1B‐mediated oncogenic activity in HCC, we explored potential CHAF1B‐interacting proteins using the BioGRID database. Among the proteins, SETDB1 served as a candidate interactor because of its reported relationship with AKT signaling and malignant phenotypes of HCC cells (Figure 3A). Consistently, proteomic profiling from the LIHC_HBV (PDC000198) dataset demonstrated a positive correlation between CHAF1B and SETDB1 protein levels in HCC tissues (Figure 3B). In support, spatial transcriptomic analyses confirmed that SETDB1 expression was markedly elevated in malignant regions compared to adjacent non‐malignant liver. Moreover, cell–cell communication analysis highlighted SETDB1 enrichment in malignant hepatocytes associated with proliferative signaling (Figure S6A). Functionally, knockdown and overexpression experiments revealed that CHAF1B altered SETDB1 protein abundance, with comparatively limited effects on SETDB1 mRNA levels (Figure S6B,C). Consistently, xenograft models demonstrated increased SETDB1 protein expression in tumors derived from CHAF1B‐overexpressing cells (Figure S6D). Immunofluorescence analysis confirmed co‐localization of CHAF1B and SETDB1 in HCC cells (Figure 3C). PLA assays detected endogenous proximity between CHAF1B and SETDB1 (Figure 3D). Co‐IP assays demonstrated that CHAF1B and SETDB1 interacted endogenously in HCC cells (Figure 3E), as well as exogenously in HEK‐293T cells (Figure 3F). Domain truncation assays suggested that deletion of the WD40 repeat‐containing region markedly impaired CHAF1B–SETDB1 binding, whereas deletion of the p150‐interaction region retained substantial SETDB1‐binding capacity (Figure 3G). These results indicate that the WD40 repeat‐containing region contributes to efficient CHAF1B association with SETDB1.

**FIGURE 3:**
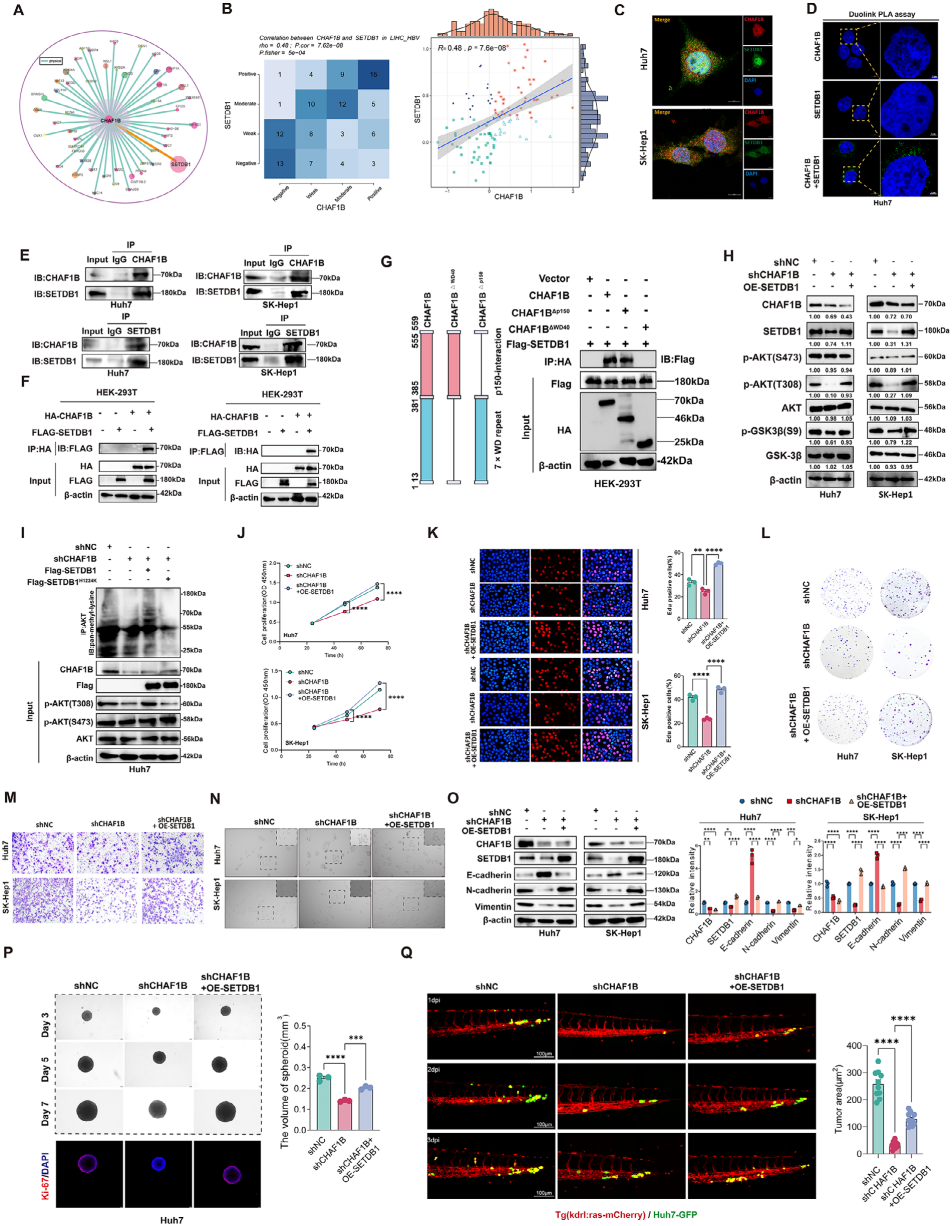
CHAF1B/SETDB1 interaction supports AKT methylation, AKT T308 phosphorylation, and malignant phenotypes in HCC. (A) BioGRID database prediction of CHAF1B‐interacting proteins, highlighting SETDB1 as a candidate interactor. (B) Proteomic analysis of LIHC_HBV (PDC000198) dataset showing a positive correlation between CHAF1B and SETDB1 protein abundance in HCC tissues. (C) Confocal immunofluorescence assays demonstrating nuclear co‐localization of CHAF1B and SETDB1 in HCC cells. (D) Proximity ligation assay detecting endogenous proximity between CHAF1B and SETDB1 in Huh7 cells. (E) Endogenous reciprocal co‐immunoprecipitation assays confirming the association between CHAF1B and SETDB1 in Huh7 and SK‐Hep1 cells, with IgG serving as the negative control. (F) Ectopic co‐immunoprecipitation assays supporting the association between HA‐CHAF1B and FLAG‐SETDB1 in HEK‐293T cells. (G) Truncation mapping assays showing that deletion of the WD40 repeat‐containing region markedly impairs CHAF1B–SETDB1 binding, whereas deletion of the p150‐interaction region retains substantial SETDB1‐binding capacity. (H) Western blotting assays showing that SETDB1 re‐expression partially restores AKT T308 phosphorylation reduced by CHAF1B depletion in Huh7 and SK‐Hep1 cells, with comparatively limited effects on total AKT and AKT S473 phosphorylation. (I) AKT immunoprecipitation followed by pan‐methyl‐lysine immunoblotting showing that CHAF1B depletion reduces AKT methylation. SETDB1‐WT efficiently restores AKT methylation and AKT T308 phosphorylation in CHAF1B‐depleted cells, whereas the catalytically inactive SETDB1‐H1224K mutant is markedly less effective. (J–N) Functional rescue assays demonstrating that SETDB1 re‐expression partially restores proliferation (J, CCK‐8; K, EdU; L, colony formation), migration (M, Transwell), and mesenchymal‐like morphology (N) in CHAF1B‐depleted HCC cells. (O) Western blot analysis and densitometric quantification showing that SETDB1 re‐expression partially reverses the EMT‐associated marker changes caused by CHAF1B depletion, including changes in E‐cadherin, N‐cadherin, and Vimentin. (P) Representative images of three‐dimensional spheroids at Days 3, 5, and 7. CHAF1B depletion reduces spheroid growth, whereas SETDB1 re‐expression partially restores Day 7 spheroid volume. Day 7 spheroid volume was calculated from three independently cultured spheroids per group. Data are presented as mean ± SD; each data point represents one spheroid. (Q) Representative fluorescence images of Tg(kdrl:ras‐mCherry) embryos injected with Huh7‐GFP cells carrying shNC, shCHAF1B, or shCHAF1B plus SETDB1, together with quantification of fluorescent tumor area at the indicated days post‐injection. Relative band intensities are shown below the corresponding lanes. All protein signals, including phosphorylated and total proteins, were independently normalized to β‐actin, and the corresponding control group within each cell line was set to 1.00. ns, not significant; **p < 0.01; ***p < 0.001; ****p < 0.0001. Quantitative cell‐based assays were performed in three independent experiments (*n* = 3). For the spheroid analysis in (P), n = 3 independently cultured spheroids per group; for the zebrafish analysis in (Q), *n* = 10 embryos per group. Data are presented as mean ± SD. Comparisons among the three rescue groups were analyzed using one‐way ANOVA followed by Tukey's multiple‐comparisons test.

Mechanistically, CHAF1B knockdown markedly reduced AKT phosphorylation at T308, with comparatively limited effects on AKT S473 phosphorylation. Re‐expression of SETDB1 restored AKT T308 phosphorylation in CHAF1B‐depleted cells, whereas total AKT and AKT S473 phosphorylation were comparatively less affected (Figure 3H). To determine whether the methyltransferase activity of SETDB1 contributes to AKT T308 phosphorylation, we examined AKT methylation and performed catalytic rescue experiments. AKT immunoprecipitation followed by pan‐methyl‐lysine immunoblotting showed that CHAF1B depletion reduced AKT methylation. Re‐expression of SETDB1‐WT restored AKT methylation and AKT T308 phosphorylation in CHAF1B‐depleted cells, whereas the catalytically inactive SETDB1‐H1224K mutant was markedly less effective. Total AKT protein and AKT S473 phosphorylation showed comparatively limited changes (Figure 3I). These findings support a functional contribution of SETDB1 catalytic activity to CHAF1B‐associated AKT methylation and AKT T308 phosphorylation.

We next examined whether SETDB1 catalytic activity also contributes to the downstream lipogenic and malignant phenotypes associated with CHAF1B. In CHAF1B‐depleted Huh7 cells, SETDB1‐WT restored the protein expression of FASN, PLIN2, and SCD, as well as the mRNA expression of FASN, ACACA, and SCD. In contrast, SETDB1‐H1224K was markedly less effective in restoring these lipogenic readouts (Figure S7A,B). Consistently, SETDB1‐WT restored lipid‐droplet accumulation and EdU incorporation, whereas SETDB1‐H1224K showed limited rescue activity (Figure S7C). SETDB1‐WT also restored cell migration, colony formation, and spheroid growth in CHAF1B‐depleted cells, while the catalytically inactive mutant failed to efficiently restore these phenotypes (Figure S7D,E). These results further indicate that the catalytic activity of SETDB1 contributes to the lipogenic and malignant phenotypes associated with the CHAF1B/SETDB1 module.

We further evaluated the functional contribution of SETDB1 in HCC cells. SETDB1 re‐expression partially restored proliferative and migratory phenotypes suppressed by CHAF1B depletion (Figure 3J–M). Morphological analyses showed that SETDB1 re‐expression partially restored the mesenchymal‐like morphology lost after CHAF1B depletion (Figure 3N). Consistently, SETDB1 re‐expression partially reversed the CHAF1B depletion‐induced changes in E‐cadherin, N‐cadherin, and Vimentin expression (Figure 3O). In three‐dimensional spheroid assays, SETDB1 significantly rescued impaired spheroid growth caused by CHAF1B knockdown (Figure 3P). Additionally, in a zebrafish xenograft model using Tg(kdrl:ras‐mCherry) embryos and Huh7‐GFP cells, SETDB1 re‐expression partially restored the fluorescent tumor area reduced by CHAF1B depletion (Figure 3Q). Collectively, these findings support a model in which SETDB1 contributes to CHAF1B‐associated lipogenic and malignant phenotypes, at least in part through its catalytic activity and its effects on AKT methylation and AKT T308‐linked signaling in HCC cells.

### 2.5 CHAF1B Promotes a K63‐Linked, Non‐Degradative SETDB1 State Associated With SETDB1 Persistence, Cytoplasmic Redistribution, and AKT T308‐Linked Signaling

To investigate how CHAF1B controls SETDB1 protein abundance, we first examined SETDB1 protein stability. CHAF1B knockdown reduced SETDB1 protein levels in Huh7 and SK‐Hep1 cells, and this reduction was partially restored by the proteasome inhibitor MG132 but not by the lysosome/autophagy inhibitor chloroquine (CQ) (Figure 4A). CHX‐chase assays further showed that CHAF1B depletion accelerated SETDB1 degradation, whereas CHAF1B overexpression prolonged SETDB1 persistence in HepG2 and MHCC97H cells (Figure 4B,C). Additional CHX‐chase experiments showed that inhibition of CRM1‐dependent export by LMB or disruption of K63 linkage by HA‐Ub‐K63R accelerated SETDB1 loss in CHAF1B‐overexpressing cells (Figure S8A). These findings indicate that SETDB1 becomes more susceptible to proteasome‐dependent loss when CHAF1B is depleted and that K63‐competent ubiquitination and CRM1‐dependent redistribution are associated with SETDB1 persistence.

**FIGURE 4:**
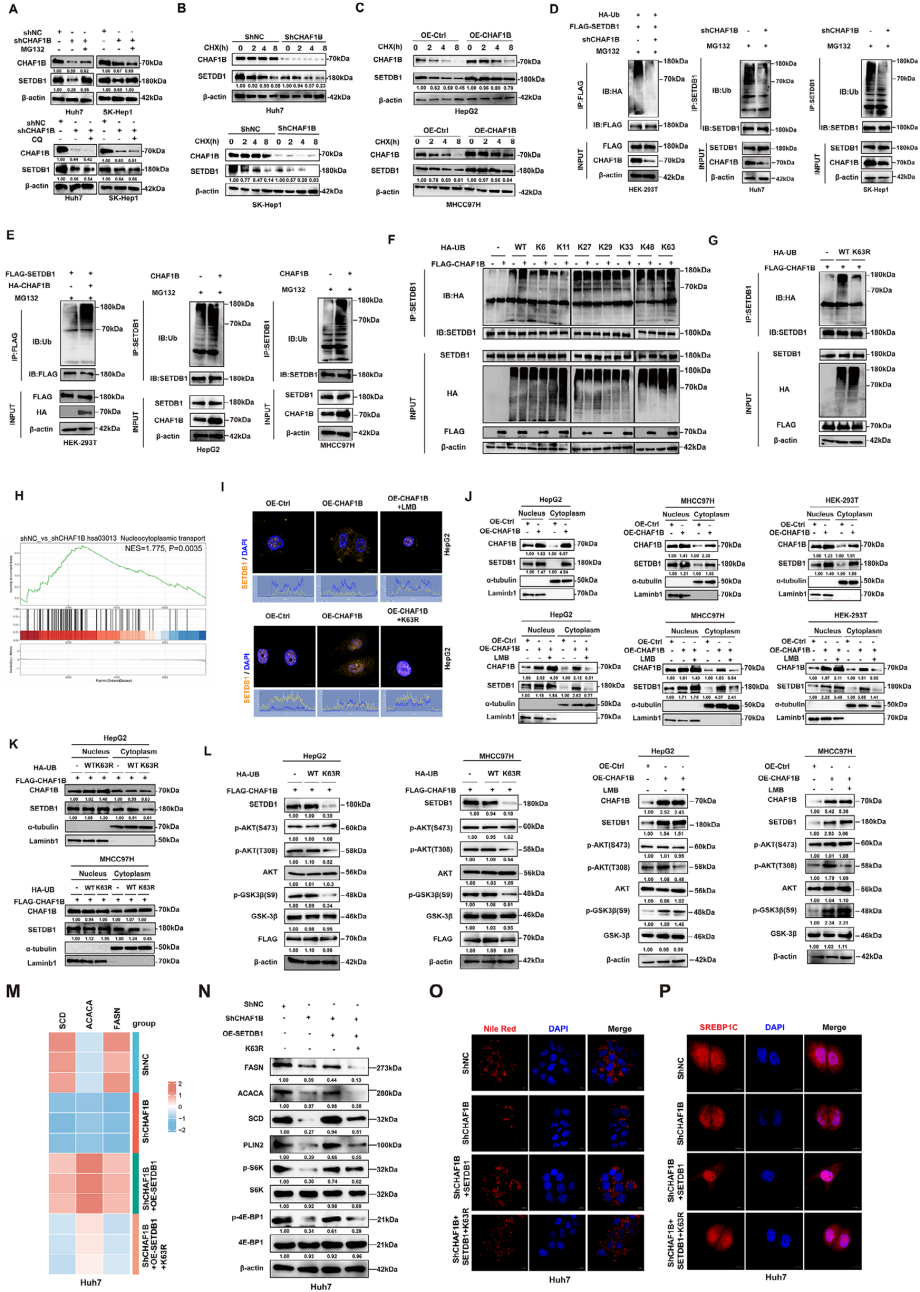
CHAF1B promotes a K63‐linked, non‐degradative SETDB1 state associated with cytoplasmic redistribution, AKT T308‐linked signaling, and lipogenic readouts. (A) Immunoblotting in Huh7 and SK‐Hep1 cells shows that CHAF1B knockdown reduces SETDB1 protein. MG132, rather than CQ, partially restores SETDB1, supporting proteasome‐dependent rather than lysosomal/autophagic loss of SETDB1 after CHAF1B depletion. (B) CHX‐chase assays showing that CHAF1B depletion accelerates SETDB1 turnover in Huh7 and SK‐Hep1 cells. (C) CHX‐chase assays showing that CHAF1B overexpression prolongs SETDB1 persistence in HepG2 and MHCC97H cells. (D) Ubiquitination assays showing that CHAF1B depletion reduces SETDB1‐associated ubiquitination in HEK‐293T cells co‐expressing HA‐Ub and FLAG‐SETDB1 and reduces endogenous SETDB1 ubiquitination in Huh7 and SK‐Hep1 cells. (E) Ubiquitination assays showing that CHAF1B overexpression increases SETDB1‐associated polyubiquitination in HEK‐293T, HepG2, and MHCC97H cells. (F) Ubiquitin‐linkage profiling showing that CHAF1B most prominently enhances K63‐linked ubiquitination of SETDB1, whereas K6‐, K11‐, K27‐, K29‐, K33‐, and K48‐linked ubiquitination shows comparatively limited changes. (G) SETDB1 ubiquitination assays showing that HA‐Ub‐K63R markedly attenuates CHAF1B‐induced SETDB1 ubiquitination compared with HA‐Ub‐WT. (H) GSEA of DIA‐based quantitative proteomic data showing positive enrichment of the KEGG nucleocytoplasmic transport pathway (hsa03013) in shNC relative to shCHAF1B Huh7 cells (NES = 1.775, *p* = 0.0035). (I) Confocal microscopy and line‐scan analysis showing that CHAF1B overexpression promotes cytoplasmic redistribution of SETDB1, whereas LMB or HA‐Ub‐K63R attenuates this redistribution. (J) Nuclear/cytoplasmic fractionation showing that CHAF1B overexpression increases cytoplasmic SETDB1 accumulation in HepG2, MHCC97H, and HEK‐293T cells, whereas LMB reduces this redistribution. (K) Nuclear/cytoplasmic fractionation showing that HA‐Ub‐K63R attenuates CHAF1B‐induced cytoplasmic accumulation of SETDB1 in HepG2 and MHCC97H cells. (L) Whole‐cell lysate analysis showing that disruption of K63 linkage by HA‐Ub‐K63R or inhibition of CRM1‐dependent export by LMB reduces SETDB1 abundance, AKT T308 phosphorylation, and GSK3β S9 phosphorylation in CHAF1B‐overexpressing HepG2 and MHCC97H cells, with comparatively limited effects on total AKT and AKT S473 phosphorylation. (M) RT–qPCR analysis of FASN, ACACA, and SCD mRNA expression in shNC, shCHAF1B, shCHAF1B plus SETDB1, and shCHAF1B plus SETDB1/HA‐Ub‐K63R Huh7 cells. (N) Immunoblotting showing that SETDB1 re‐expression restores FASN, ACACA, SCD, and PLIN2 abundance and mTORC1‐associated readouts, including p‐S6K and p‐4E‐BP1, in CHAF1B‐depleted Huh7 cells, whereas HA‐Ub‐K63R markedly weakens these rescue effects. (O) Nile Red staining showing that SETDB1 re‐expression restores lipid‐droplet accumulation in CHAF1B‐depleted Huh7 cells, whereas HA‐Ub‐K63R markedly attenuates this rescue. (P) Immunofluorescence showing that SETDB1 re‐expression restores SREBP1C nuclear localization in CHAF1B‐depleted Huh7 cells, whereas HA‐Ub‐K63R weakens this effect. For immunoblot panels with relative band intensities shown below the corresponding lanes, whole‐cell lysate signals, including phosphorylated and total proteins, were independently normalized to β‐actin. Nuclear and cytoplasmic protein signals in the subcellular fractionation panels were normalized to Lamin B1 and α‐tubulin, respectively. Within each cell line and experiment, the corresponding control group was set to 1.00. CHX‐chase decay curves in (B,C) were generated from three independent experiments (*n* = 3). The DIA proteomic dataset underlying (H) comprised *n* = 3 biological replicates per group, and GSEA significance was assessed using 1000 permutations.

We next assessed whether CHAF1B regulates SETDB1 ubiquitination. In HEK‐293T cells co‐expressing HA‐ubiquitin and FLAG‐SETDB1, CHAF1B depletion reduced SETDB1‐associated ubiquitination. Similar reductions were observed for endogenous SETDB1 in Huh7 and SK‐Hep1 cells (Figure 4D). Conversely, CHAF1B overexpression increased SETDB1‐associated polyubiquitination in HEK‐293T, HepG2, and MHCC97H cells (Figure 4E). Ubiquitin‐linkage profiling showed that the most prominent CHAF1B‐associated increase occurred in K63‐linked ubiquitination of SETDB1, whereas K6‐, K11‐, K27‐, K29‐, K33‐, and K48‐linked ubiquitination showed comparatively limited changes (Figure 4F). Consistently, HA‐Ub‐K63R markedly attenuated CHAF1B‐induced SETDB1 ubiquitination (Figure 4G).

Reciprocal loss‐of‐function analysis further showed that CHAF1B depletion reduced total and K63‐linked ubiquitination of SETDB1, whereas K48‐linked ubiquitination did not show a reproducible increase (Figure S8B). Moreover, comparison of HA‐Ub‐WT, HA‐Ub‐K48R, and HA‐Ub‐K63R showed that K63R produced a substantially greater reduction in CHAF1B‐induced SETDB1 ubiquitination than K48R (Figure S8C). These findings support a predominantly K63‐dependent, non‐degradative ubiquitin state on SETDB1.

Because CHAF1B is a CAF‐1 subunit that engages ASF1A during replication‐coupled nucleosome assembly, we tested whether this specific ASF1A‐binding interface is required for CHAF1B‐dependent regulation of SETDB1 and AKT signaling. The ASF1A‐binding–deficient CHAF1B ^RR482/483AA^ mutant behaved similarly to CHAF1B‐WT in HEK‐293T and HepG2 cells. It increased SETDB1 abundance, preferentially enhanced AKT T308 phosphorylation with comparatively limited effects on AKT S473 phosphorylation, and retained the ability to promote SETDB1 ubiquitination (Figure S9A–C). These findings indicate that CHAF1B‐dependent regulation of SETDB1 abundance, SETDB1 ubiquitination, and AKT T308‐linked signaling is not strictly dependent on the tested ASF1A‐binding interface.

Given that K63‐linked polyubiquitin commonly mediates non‐degradative signaling and trafficking functions [22], and because DIA proteomics showed enrichment of nucleocytoplasmic transport‐associated proteins (Figure 4H), we examined whether CHAF1B regulates SETDB1 subcellular localization. Confocal imaging showed that CHAF1B overexpression promoted cytoplasmic redistribution of SETDB1. This redistribution was impaired by LMB, an inhibitor of CRM1/exportin‐1, and by HA‐Ub‐K63R (Figure 4I). Nuclear/cytoplasmic fractionation in HepG2, MHCC97H, and HEK‐293T cells further confirmed that CHAF1B increased cytoplasmic SETDB1 accumulation, whereas LMB or HA‐Ub‐K63R reduced this redistribution (Figure 4J,K).

In CHAF1B‐depleted cells, SETDB1 re‐expression partially restored cytoplasmic SETDB1 accumulation, whereas HA‐Ub‐K63R markedly impaired this rescue, as shown by confocal imaging and nuclear/cytoplasmic fractionation (Figure S8D,E). Additional fractionation experiments showed that MG132 increased SETDB1 abundance when K63 linkage or CRM1‐dependent export was disrupted but did not re‐establish cytoplasmic SETDB1 accumulation (Figure S8F).

In parallel, whole‐cell lysate analyses showed that disruption of K63 linkage by HA‐Ub‐K63R or inhibition of CRM1‐dependent export by LMB attenuated AKT T308 phosphorylation and downstream GSK3β S9 phosphorylation, with comparatively limited effects on total AKT and AKT S473 phosphorylation (Figure 4L). These findings indicate that SETDB1 protein abundance and SETDB1 signaling competence represent related but separable layers of regulation. Proteasome inhibition can increase SETDB1 abundance, but it cannot substitute for the K63‐competent, cytoplasmically redistributed SETDB1 state associated with efficient AKT T308‐linked signaling.

To assess whether this K63‐dependent SETDB1 state contributes to the lipogenic output downstream of CHAF1B, we performed rescue experiments in CHAF1B‐depleted Huh7 cells. Re‐expression of SETDB1 restored mRNA expression of FASN, ACACA, and SCD (Figure 4M), increased the protein abundance of FASN, ACACA, SCD, and PLIN2, and restored mTORC1‐associated readouts, including p‐S6K and p‐4E‐BP1 (Figure 4N). In contrast, HA‐Ub‐K63R markedly weakened these rescue effects. Consistently, SETDB1 re‐expression restored lipid‐droplet accumulation and SREBP1C nuclear localization, whereas HA‐Ub‐K63R was substantially less effective in supporting these phenotypes (Figure 4O,P).

Collectively, these results support a model in which CHAF1B/UHRF1‐associated K63‐dependent ubiquitination contributes to a non‐degradative, redistribution‐competent SETDB1 state associated with protein persistence and signaling competence. CHAF1B depletion reduces K63‐linked SETDB1 ubiquitination and increases susceptibility to proteasome‐dependent loss without producing a reproducible increase in K48‐linked ubiquitination. Disruption of K63 linkage or CRM1‐dependent export further impairs SETDB1 persistence, cytoplasmic accumulation, AKT T308‐linked signaling, and downstream lipogenic readouts.

### 2.6 UHRF1 Promotes CHAF1B‐Associated K63‐Dependent Ubiquitination of SETDB1

To identify candidate E3 ligases associated with CHAF1B‐dependent SETDB1 ubiquitination, endogenous CHAF1B complexes were immunoprecipitated from Huh7 and SK‐Hep1 cells and analyzed by mass spectrometry. UHRF1 emerged as a candidate interactor in both lines (Figure 5A). UHRF1 contains a RING E3 ligase domain and has previously been implicated in K63‐linked ubiquitination [23]. Additionally, analysis of the LIHC_HBV proteomic cohort (PDC000198) showed a positive correlation between UHRF1 and SETDB1 protein abundance in HCC tissues (Figure 5B). In HEK‐293T cells, UHRF1 overexpression partially restored SETDB1 abundance reduced by CHAF1B depletion, indicating a functional relationship among CHAF1B, UHRF1, and SETDB1 (Figure 5C). Spatial transcriptomics further showed higher UHRF1 expression in malignant regions than in adjacent liver and linked UHRF1 to malignant cell activity by cell–cell communication analysis (Figure 5D). Proximity ligation assays in Huh7 cells detected endogenous proximity between UHRF1 and CHAF1B and between UHRF1 and SETDB1 (Figure 5E). Consistently, co‐immunoprecipitation confirmed both interactions in Huh7 and SK‐Hep1 cells (Figure 5F,G). To determine whether CHAF1B contributes to the endogenous UHRF1–SETDB1 association, we performed UHRF1 immunoprecipitation in control and CHAF1B‐depleted Huh7 cells. CHAF1B depletion reduced the amount of SETDB1 recovered in UHRF1 immunoprecipitates, whereas the IgG controls showed no specific enrichment (Figure S10A). These findings suggest that CHAF1B facilitates the endogenous association between UHRF1 and SETDB1. We next examined whether AKT or p‐AKT(T308) was stably associated with the CHAF1B–UHRF1–SETDB1 regulatory module. CHAF1B, SETDB1, and UHRF1 were efficiently enriched in their corresponding immunoprecipitates. However, neither AKT nor p‐AKT(T308) showed specific enrichment above the corresponding IgG controls under the endogenous Co‐IP conditions used (Figure S10B). We therefore interpret AKT T308 phosphorylation as a downstream functional readout rather than evidence that AKT is a stable component of this regulatory module.

**FIGURE 5:**
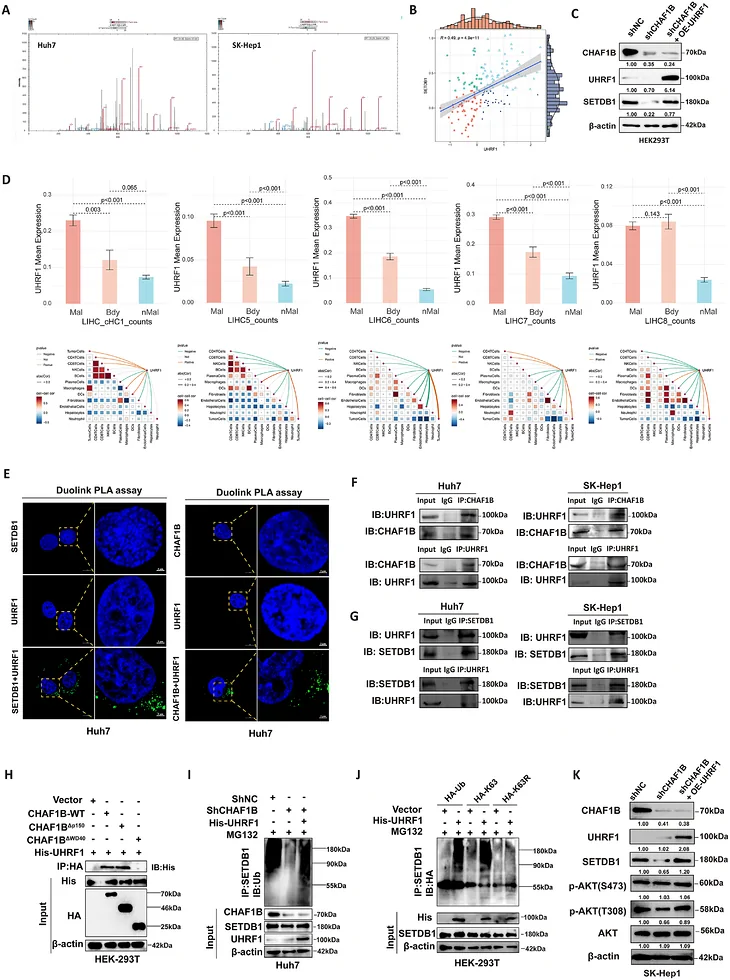
CHAF1B facilitates UHRF1‐associated K63‐dependent ubiquitination of SETDB1 and AKT T308‐linked signaling. (A) IP–MS of endogenous CHAF1B complexes in Huh7 and SK‐Hep1 identifies UHRF1 as a candidate interactor; representative spectra shown. (B) LIHC_HBV (PDC000198) proteomic analysis shows a positive correlation between UHRF1 and SETDB1 protein abundance in HCC. (C) Immunoblotting in HEK‐293T shows that UHRF1 overexpression restores SETDB1 protein reduced by CHAF1B knockdown. (D) Spatial transcriptomics shows higher UHRF1 expression in malignant regions than in adjacent liver and links UHRF1 to malignant cell programs by cell–cell communication analysis. (E) Duolink PLA in Huh7 detects endogenous proximity between UHRF1 and CHAF1B or SETDB1. (F&G) Co‐immunoprecipitation in Huh7 and SK‐Hep1 confirms endogenous interaction between UHRF1 and CHAF1B or SETDB1. (H) Truncation‐based interaction assays showing that deletion of the CHAF1B WD40 repeat‐containing region markedly weakens CHAF1B–UHRF1 association, whereas deletion of the p150‐interaction region retains substantial UHRF1‐binding capacity. (I) SETDB1 ubiquitination assays in Huh7 cells showing that UHRF1 overexpression partially restores SETDB1‐associated ubiquitination reduced by CHAF1B depletion. (J) SETDB1 ubiquitination assays in HEK‐293T cells showing that UHRF1 enhances SETDB1‐associated ubiquitination, whereas HA‐Ub‐K63R markedly attenuates this effect. (K) Immunoblotting showing that UHRF1 overexpression partially restores SETDB1 abundance and AKT T308 phosphorylation in CHAF1B‐depleted cells, with comparatively limited effects on total AKT and AKT S473 phosphorylation. For the immunoblot panels with densitometric values shown below the lanes, all protein signals, including phosphorylated and total proteins, were independently normalized to β‐actin, and the corresponding control group within each cell line was set to 1.00. Pearson correlation analysis was used in (B).

Truncation‐based assays suggested that deletion of the WD40 repeat‐containing region markedly weakened CHAF1B–UHRF1 binding, whereas deletion of the p150‐interaction region retained substantial UHRF1‐binding capacity (Figure 5H). Together with the SETDB1 truncation‐mapping results, these data suggest that the WD40 repeat‐containing region of CHAF1B contributes to efficient association with both UHRF1 and SETDB1. To further examine the role of the CHAF1B WD40 repeat‐containing region in this module, we performed CHAF1B depletion and rescue experiments followed by UHRF1 immunoprecipitation. CHAF1B depletion reduced the amount of SETDB1 recovered in UHRF1 immunoprecipitates and was accompanied by decreased SETDB1 abundance and AKT T308 phosphorylation. Re‐expression of CHAF1B‐WT restored the UHRF1–SETDB1 association and SETDB1 abundance, whereas CHAF1B‐ΔWD40 showed a comparatively weaker rescue. Total AKT and AKT S473 phosphorylation showed comparatively limited changes (Figure S10C). These findings support a contribution of the CHAF1B WD40 repeat‐containing region to the organization of the UHRF1–SETDB1 functional module.

To determine whether UHRF1 promotes K63‐dependent ubiquitination of SETDB1, we performed complementary ubiquitination assays. UHRF1 overexpression partially restored SETDB1‐associated ubiquitination reduced by CHAF1B depletion in Huh7 cells (Figure 5I). In HEK‐293T cells, UHRF1 increased SETDB1‐associated ubiquitination, whereas HA‐Ub‐K63R markedly attenuated this effect compared with HA‐Ub‐WT or the linkage‐restricted HA‐Ub‐K63 construct (Figure 5J). Additional linkage analyses showed that UHRF1 overexpression preferentially restored K63‐linked SETDB1 ubiquitination in CHAF1B‐depleted cells, whereas K48‐linked ubiquitination showed comparatively limited restoration (Figure S10D). Consistently, HA‐Ub‐K63R markedly impaired the UHRF1 overexpression‐associated restoration of SETDB1‐associated ubiquitination (Figure S10E). Functionally, UHRF1 overexpression partially restored AKT T308 phosphorylation reduced by CHAF1B depletion, with comparatively limited effects on total AKT and AKT S473 phosphorylation (Figure 5K). Collectively, these findings support UHRF1 as a candidate CHAF1B‐associated E3 ligase that promotes K63‐dependent ubiquitination of SETDB1. CHAF1B facilitates the UHRF1–SETDB1 association through its WD40 repeat‐containing region, supporting a non‐degradative, redistribution‐competent SETDB1 state associated with AKT T308‐linked signaling.

### 2.7 CHAF1B Depletion Promotes VHL‐Dependent K11‐Associated Degradative Ubiquitination of SETDB1

Having defined the CHAF1B/UHRF1‐associated K63‐dependent, non‐degradative state of SETDB1, we next sought to identify the ubiquitin linkage associated with the proteasome‐dependent loss of SETDB1 following CHAF1B depletion. We therefore performed additional loss‐of‐function ubiquitin‐linkage profiling under proteasome inhibition. Among the tested linkage‐restricted ubiquitin constructs, CHAF1B depletion most prominently increased K11‐associated ubiquitination of SETDB1 (Figure S11A). To further examine the contribution of K11 linkage, we compared HA‐Ub‐WT, linkage‐restricted HA‐Ub‐K11, and the K11‐deficient HA‐Ub‐K11R mutant. HA‐Ub‐K11 markedly enhanced SETDB1‐associated ubiquitination in CHAF1B‐depleted cells, whereas HA‐Ub‐K11R substantially attenuated this ubiquitination signal (Figure S11B). Together with the accelerated SETDB1 turnover and MG132‐sensitive SETDB1 loss observed after CHAF1B depletion, these findings support a predominant contribution of K11‐associated ubiquitination to the degradative SETDB1 state following CHAF1B loss. We next investigated the degradative E3 pathway associated with this state. VHL was prioritized because SETDB1 has previously been identified as a substrate of the CRL2/VHL E3 ubiquitin ligase complex, in which VHL functions as the substrate‐recognition component and promotes ubiquitin‐dependent proteasomal degradation of SETDB1 [24]. VHL depletion partially restored SETDB1 protein abundance in CHAF1B‐depleted Huh7 cells (Figure S11C). Reciprocal endogenous co‐immunoprecipitation further showed that CHAF1B depletion increased the SETDB1–VHL association, whereas CHAF1B re‐expression attenuated this interaction (Figure S11D,E). Importantly, VHL depletion markedly reduced the increase in K11‐associated SETDB1 ubiquitination following CHAF1B depletion (Figure S11F). Collectively, these results support a VHL‐dependent degradative pathway in which CHAF1B loss favors SETDB1–VHL association and a predominantly K11‐associated ubiquitin state linked to proteasomal SETDB1 turnover.

### 2.8 The Clinical Implications of the CHAF1B/SETDB1 Axis in HCC

We next assessed the clinical relevance of the CHAF1B/SETDB1 axis. In the Sleeping Beauty–based hydrodynamic injection model, CHAF1B cooperated with the c‐Myc/myr‐AKT oncogenic background to enhance hepatocarcinogenesis. Primary tumors from the CHAF1B co‐expression group showed stronger histological evidence of malignancy and higher SETDB1 staining than vector controls (Figure 6A). In human specimens, representative multiplex immunofluorescence (mIF) images showed stronger CHAF1B, SETDB1, and UHRF1 signals in HCC than in adjacent non‐tumor liver (Figure 6B). On an HCC tissue microarray, IHC revealed higher CHAF1B and SETDB1 staining in tumors than in paired non‐tumor liver tissues (Figure 6C). Quantitative scoring confirmed higher CHAF1B and SETDB1 levels in tumors than in adjacent tissue (Figure 6D). Patients stratified by CHAF1B or SETDB1 expression showed a higher proportion of metastasis in the corresponding high‐expression groups (Figure 6E). Representative axial MRI images were included for descriptive illustration of tumor morphology across the indicated CHAF1B–SETDB1 expression groups (Figure 6F). Kaplan–Meier analysis of the tissue microarray cohort indicated that high CHAF1B or high SETDB1 expression was associated with poorer overall survival, and the dual‐high subgroup showed the least favorable overall survival among the analyzed groups (Figure 6G). Analysis of the TCGA‐LIHC and GSE14520 cohorts yielded a consistent pattern, with concurrent high CHAF1B and SETDB1 expression being associated with unfavorable prognosis (Figure 6H).

**FIGURE 6:**
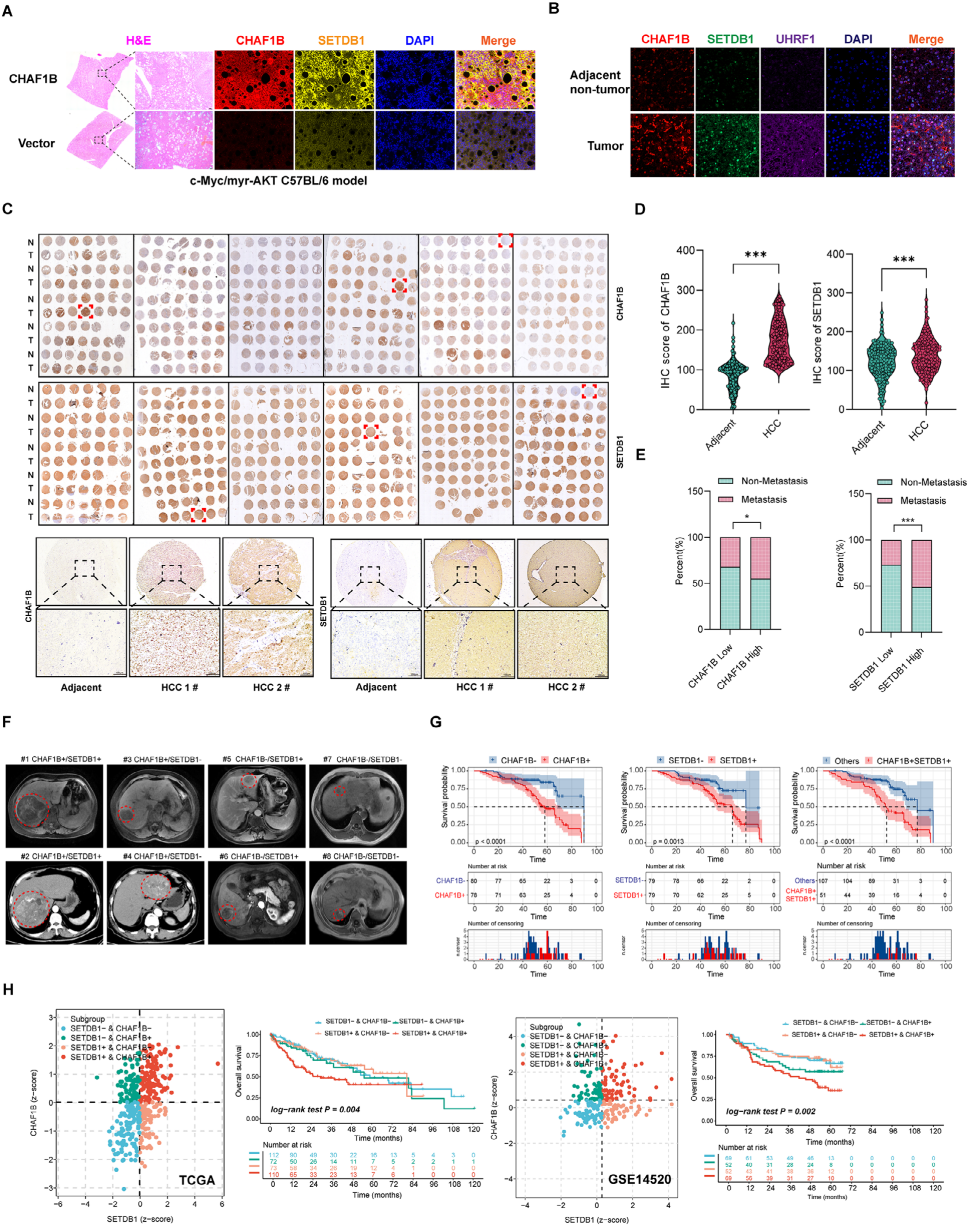
Clinical significance of the CHAF1B–SETDB1 axis in HCC. (A) In the Sleeping Beauty hydrodynamic injection model (c‐Myc/myr‐AKT ± CHAF1B), primary tumors from the CHAF1B group show enhanced malignancy on H&E and increased SETDB1 staining. (B) Representative multiplex immunofluorescence (mIF) images show stronger CHAF1B, SETDB1, and UHRF1 signals in HCC than in adjacent non‐tumor liver samples. (C) Tissue microarray IHC illustrates strong CHAF1B and SETDB1 in HCC tissues. Red dashed boxes indicate the representative cores shown at higher magnification below. (D) Quantitative IHC scoring demonstrates higher CHAF1B and SETDB1 expression in tumors compared with matched non‐tumor tissue. (E) Patients with high CHAF1B or SETDB1 expression display a higher proportion of patients with metastasis. (F) Representative axial liver MRI from patients stratified by tumoral CHAF1B/SETDB1 status. Red dashed circles denote tumor foci. (G) Kaplan–Meier analysis of the microarray cohort shows worse overall survival with high CHAF1B or high SETDB1, with the dual‐high group having the poorest outcome. (H) Analyses of TCGA‐LIHC and GSE14520 cohorts confirm that concurrent high CHAF1B and SETDB1 associates with unfavorable prognosis. Paired tumor versus adjacent non‐tumor comparisons were analyzed using paired two‐sided Student's *t*‐tests. Categorical variables were compared using the χ^2^ test or Fisher's exact test, as appropriate. Survival curves were compared using two‐sided log‐rank tests. Data are presented as mean ± SD where applicable. ***, *p* < 0.001; *, *p* < 0.05. For the Sleeping Beauty model in (A), *n* = 3 mice per group. The tissue microarray cohort comprised *n* = 158 HCC cases. For (D), paired tumor‐versus‐adjacent comparisons were analyzed using paired two‐sided Student's t‐tests; categorical variables in (E) were analyzed using the χ^2^ test or Fisher's exact test, as appropriate; and survival curves in (G,H) were compared using two‐sided log‐rank tests.

### 2.9 USP4‐Dependent Stabilization of CHAF1B Supports Malignant and Lipogenic Phenotypes

To identify upstream regulators of CHAF1B protein stability, we systematically interrogated deubiquitinases. A focused CRISPR‐based screen targeting 90 deubiquitinases identified USP4 as one of the most consistent regulators of CHAF1B protein abundance (Figure 7A). Independent IP–MS in HCC cells also identified USP4 in CHAF1B complexes, supporting a physical association (Figure S12A). Confocal microscopy showed intracellular colocalization of CHAF1B and USP4 (Figure 7B). Endogenous and ectopic co‐immunoprecipitation confirmed the interaction in Huh7 and HEK‐293T cells (Figure 7C, D).

**FIGURE 7:**
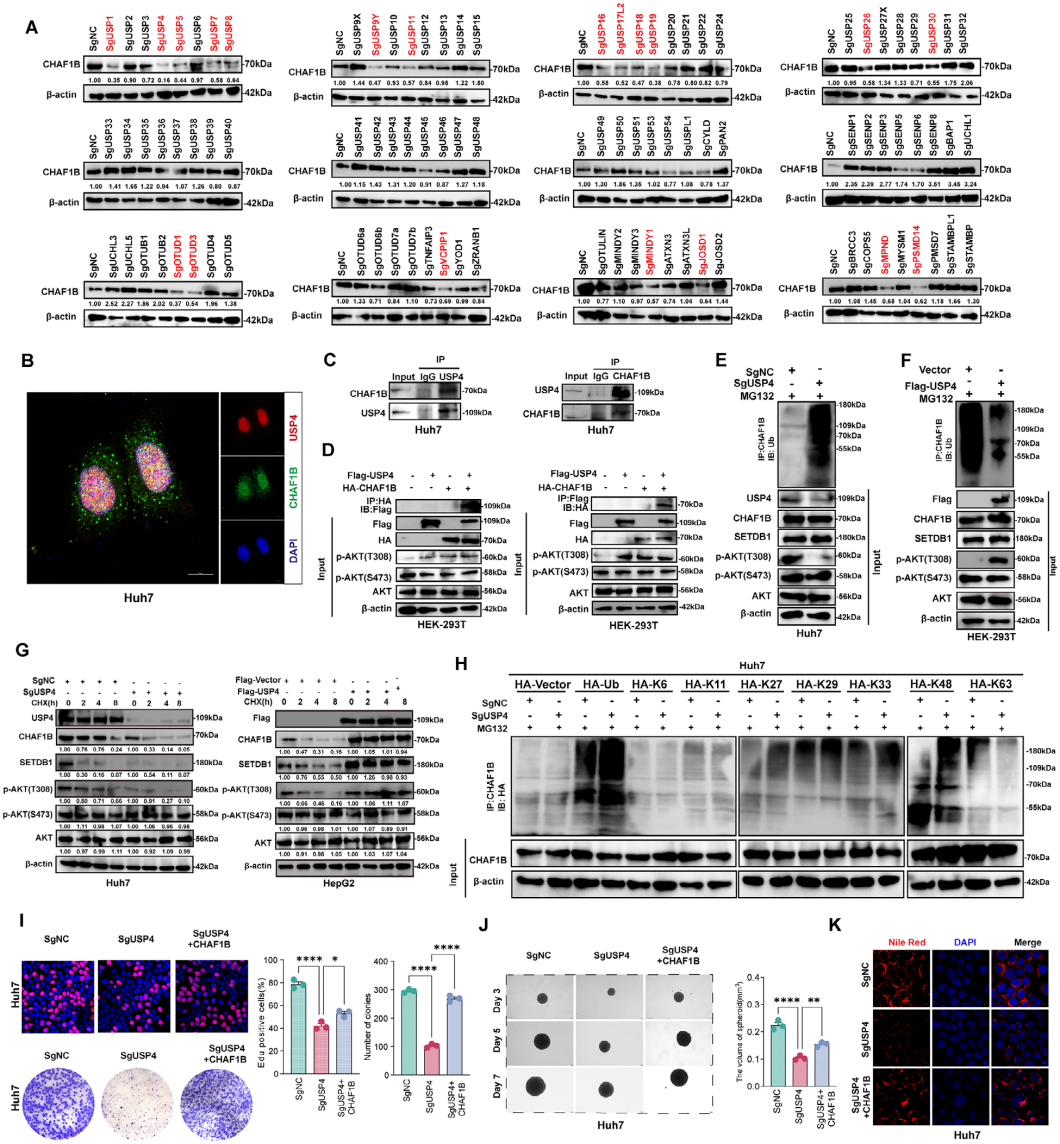
USP4‐dependent stabilization of CHAF1B supports malignant and lipogenic phenotypes. (A) A focused CRISPR‐based screen of deubiquitinases identifying USP4 as a prominent regulator of CHAF1B protein abundance. (B) Confocal immunofluorescence showing intracellular colocalization of CHAF1B and USP4 in Huh7 cells. (C) Endogenous co‐immunoprecipitation in Huh7 confirming the CHAF1B–USP4 interaction. (D) Ectopic Co‐IP in HEK‐293T validating the interaction between FLAG‐USP4 and HA‐CHAF1B. (E) Ubiquitination assays showing increased CHAF1B‐associated ubiquitination after USP4 depletion. Corresponding input analyses show reduced CHAF1B abundance and AKT T308 phosphorylation, with comparatively limited effects on total AKT and AKT S473 phosphorylation. (F) Ubiquitination assays in HEK‐293T showing reduced CHAF1B polyubiquitination upon USP4 overexpression. (G) Cycloheximide‐chase analysis demonstrating that USP4 prolongs CHAF1B half‐life, whereas USP4 loss accelerates degradation. Whole‐cell lysate analyses show that altered CHAF1B stability is accompanied by corresponding changes in AKT T308 phosphorylation. (H) Ubiquitin‐linkage profiling showing that USP4 depletion most prominently increases K48‐linked ubiquitination of CHAF1B, with comparatively limited effects on the other tested linkages. (I–K) Rescue experiments showing that CHAF1B re‐expression in USP4‐deficient cells partially restores EdU incorporation and colony formation (I), spheroid growth (J), and lipid‐droplet accumulation (K). For the immunoblot panels with densitometric values shown below the lanes, all protein signals, including phosphorylated and total proteins, were independently normalized to β‐actin, and the corresponding control group within each cell line was set to 1.00. ****p < 0.0001, **p < 0.01, *p < 0.05. The CHX‐chase experiment in (G) was performed in three independent experiments (*n* = 3). Quantitative analyses in (I) were performed in three independent experiments (*n* = 3), and the spheroid analysis in (J) used *n* = 3 independently cultured spheroids per group. Data are presented as mean ± SD. Comparisons among the three groups in the rescue experiments were analyzed using one‐way ANOVA followed by Tukey's multiple‐comparisons test.

USP4 modulation altered malignant phenotypes in a manner consistent with changes in CHAF1B abundance. USP4 silencing reduced proliferation, clonogenicity, migration, spheroid expansion, and lipid‐droplet accumulation, whereas USP4 overexpression enhanced these phenotypes (Figure S12B–F). Modulating USP4 changed CHAF1B protein levels without altering mRNA levels, supporting USP4‐mediated post‐translational regulation of CHAF1B protein stability (Figure S12G,H). MG132, but not chloroquine or 3‐MA, restored CHAF1B levels after USP4 knockdown, indicating that USP4 depletion promotes proteasome‐dependent degradation of CHAF1B (Figure S12I). Ubiquitination assays showed that USP4 depletion increased CHAF1B ubiquitination, whereas USP4 overexpression decreased it (Figure 7E,F). In parallel, input and total lysate analyses showed that USP4 depletion reduced AKT T308 phosphorylation together with CHAF1B loss, whereas USP4 overexpression preserved CHAF1B abundance and AKT T308 phosphorylation. Total AKT and AKT S473 phosphorylation were comparatively less affected. Cycloheximide‐chase experiments demonstrated that USP4 prolonged CHAF1B half‐life, while USP4 loss accelerated its turnover (Figure 7G). Ubiquitin‐linkage profiling showed that USP4 depletion most prominently increased K48‐linked ubiquitination of CHAF1B, with comparatively limited changes in the other tested linkages (Figure 7H). Expression of the K48R ubiquitin mutant blunted the increase in CHAF1B ubiquitination caused by USP4 depletion, supporting a predominant contribution of K48‐linked ubiquitination (Figure S12J,K). Re‐expression of CHAF1B in USP4‐deficient cells partially restored proliferation, spheroid expansion, migration, and Nile Red staining (Figure 7I–K and Figure S12L). These findings support USP4 as a CHAF1B‐associated deubiquitinase that limits K48‐linked polyubiquitination and promotes CHAF1B stability, thereby supporting CHAF1B‐associated malignant and lipogenic phenotypes in HCC.

To determine whether the USP4/CHAF1B relationship is reflected at the protein level in patient‐derived HCC tissues, we performed USP4 immunohistochemistry in the same tissue microarray cohort used for CHAF1B evaluation. Representative staining and quantitative analysis showed higher USP4 protein expression in HCC tissues than in adjacent non‐tumor liver tissues (Figure S13A–C). The USP4‐high group also contained a higher proportion of patients with metastasis than the USP4‐low group (Figure S13D). Importantly, Pearson correlation analysis of USP4 and CHAF1B IHC scores from the same patient specimens demonstrated a significant positive association between their protein levels (Figure S13E). Kaplan–Meier analysis further showed that USP4‐high patients had poorer overall survival than USP4‐low patients. Patients with concurrent high expression of USP4, CHAF1B, and SETDB1 also had poorer overall survival than the remaining patients (Figure S13F). These clinical data support a positive association between USP4 and CHAF1B protein abundance in HCC.

### 2.10 Akebia Saponin D Pharmacologically Perturbs the USP4/CHAF1B Axis and Attenuates Malignant and Lipogenic Phenotypes

We next evaluated Akebia saponin D (ASD), previously reported to inhibit USP4, as a pharmacologic probe to perturb the USP4/CHAF1B axis. Cellular thermal shift assays showed an ASD‐associated alteration in USP4 thermal stability, supporting cellular engagement of USP4 by ASD (Figure 8A). ASD attenuated malignant phenotypes in vitro in a concentration‐dependent manner, reducing EdU incorporation, colony formation, migration, and spheroid expansion (Figure S14A–D). We next examined the effects of ASD on CHAF1B proteostasis and pathway‐associated readouts. ASD did not materially change CHAF1B mRNA yet reduced CHAF1B protein (Figure 8B). MG132 restored CHAF1B after ASD exposure, whereas CQ or 3‐MA had little effect, consistently indicating proteasome‐dependent loss rather than lysosomal or autophagic degradation (Figure 8C). Cycloheximide‐chase analysis showed that ASD significantly accelerated CHAF1B degradation (Figure 8D). As shown in Figure 8E, ASD increased CHAF1B ubiquitination, whereas linkage profiling indicated a preferential rise in K48‐linked chains with minimal change in other linkages (Figure 8F). In contrast, the K48R ubiquitin mutant blunted ASD‐induced ubiquitination and mitigated the reduction of CHAF1B protein (Figure 8G,H). Consistent with a CHAF1B‐directed mechanism, ectopic CHAF1B partially rescued ASD‐induced suppression of proliferation and clonogenicity (Figure 8I), and similarly restored migration of HCC cells (Figure S14E). Lipid‐droplet accumulation, reduced by ASD, was partially restored by CHAF1B (Figure 8J). Spheroid growth inhibition was also alleviated by CHAF1B re‐expression (Figure 8K).

**FIGURE 8:**
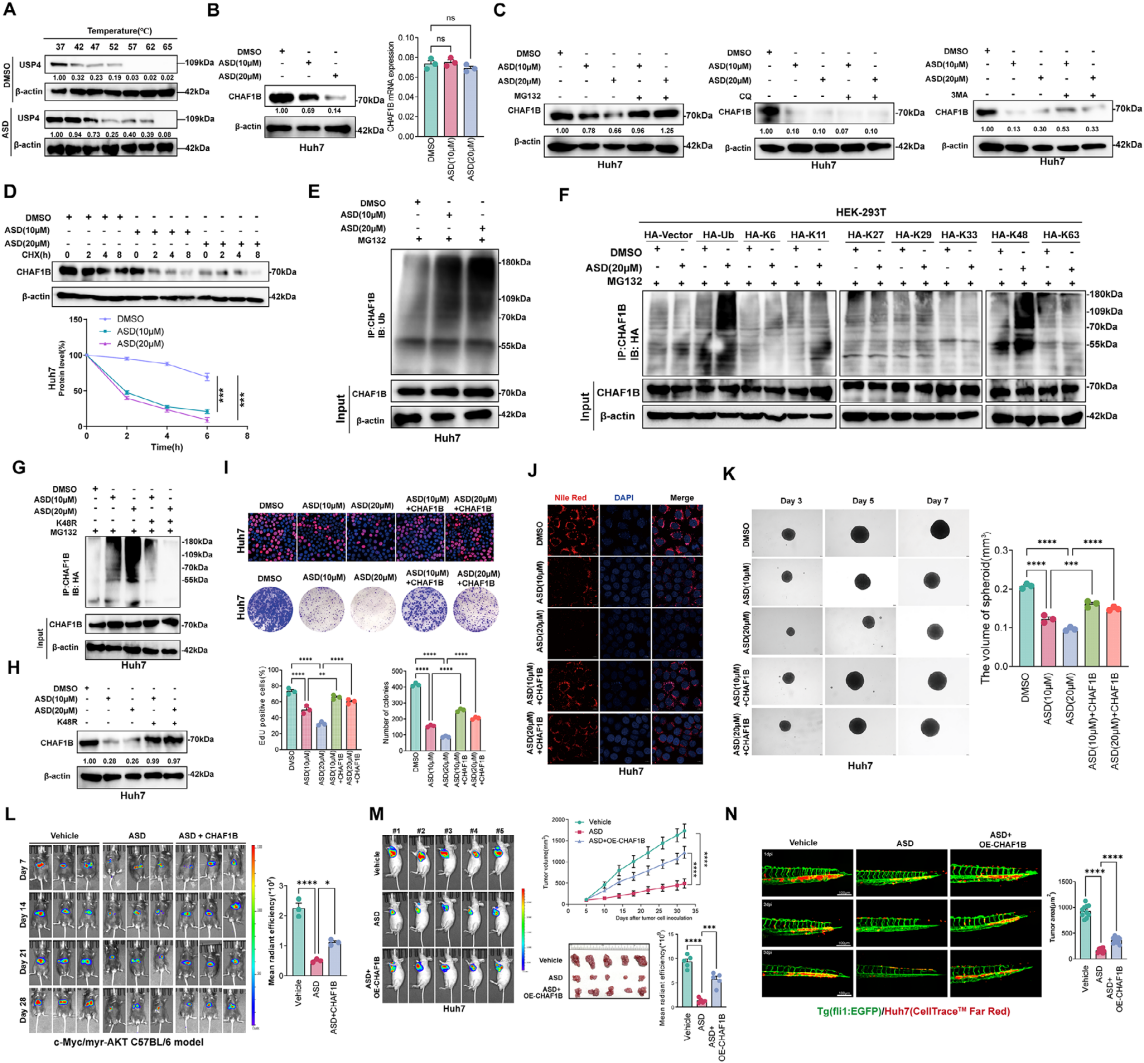
Pharmacologic perturbation of the USP4–CHAF1B axis with Akebia saponin D. (A) Representative CETSA immunoblot showing an ASD‐associated alteration in USP4 thermal stability after treatment with 100 µM ASD for 12 h. (B) RT–qPCR and immunoblotting in Huh7 cells showing that ASD (10 or 20 µM) does not materially change CHAF1B mRNA while reducing CHAF1B protein abundance. (C) Proteostasis assays indicating that MG132, but not chloroquine or 3‐methyladenine, restores CHAF1B after ASD treatment. (D) Cycloheximide‐chase analysis showing accelerated CHAF1B degradation after ASD treatment. (E) Ubiquitination assays in Huh7 cells showing increased CHAF1B polyubiquitination in the presence of ASD and MG132. (F) Ubiquitin‐linkage profiling in HEK‐293T cells showing a preferential increase in K48‐linked ubiquitination of CHAF1B with comparatively limited changes in the other tested linkages. (G) The ubiquitin K48R mutant attenuates ASD‐induced CHAF1B ubiquitination. (H) K48R mitigates the reduction in CHAF1B protein abundance caused by ASD. (I) EdU incorporation and colony‐formation assays showing that ASD suppresses proliferation and clonogenic growth, with partial rescue by ectopic CHAF1B expression. (J) Nile Red staining showing reduced lipid‐droplet accumulation after ASD treatment and partial restoration by CHAF1B re‐expression. (K) Three‐dimensional spheroid assays showing that ASD inhibits spheroid expansion and that CHAF1B re‐expression partially alleviates this effect. (L) Serial bioluminescence imaging in the c‐Myc/myr‐AKT Sleeping Beauty liver‐tumor model treated with vehicle, ASD, or ASD combined with CHAF1B overexpression, together with quantification of mean radiant efficiency at the experimental endpoint. (M) Subcutaneous Huh7 xenograft experiment showing endpoint bioluminescence imaging and quantification of mean radiant efficiency, longitudinal tumor‐volume changes, and representative excised tumors in mice treated with vehicle, ASD, or ASD combined with CHAF1B overexpression. Tumor volume was measured immediately before treatment initiation and at the indicated time points thereafter. The first measurement shown represents the pre‐treatment baseline. (N) Representative fluorescence images and quantification of tumor area in Tg(fli1:EGFP) embryos injected with CellTrace Far Red‐labeled Huh7 cells in the control, ASD‐treated, and ASD plus CHAF1B‐overexpression groups. For the immunoblot panels with densitometric values shown below the lanes, all protein signals were independently normalized to β‐actin, and the corresponding control group within each cell line was set to 1.00. Longitudinal body‐weight data are summarized in Figure S17 as mean ± SD, and the corresponding individual‐animal body‐weight, tumor‐volume, tumor‐weight, and mean radiant efficiency measurements are provided in Data S1. Data are presented as mean ± SD. ns, not significant; ****p < 0.0001; ***p < 0.001; **p < 0.01; *, *p* < 0.05. Cell‐based quantitative analyses in (B,D,I) were performed in three independent experiments (n = 3), and the spheroid analysis in (K) used *n* = 3 independently cultured spheroids per group. For the Sleeping Beauty model in (L), *n* = 3 mice per group; for the subcutaneous xenograft in (M), *n* = 5 mice per group; and for the zebrafish analysis in (N), *n* = 10 embryos per group. Comparisons among three or more groups for endpoint measurements were analyzed using one‐way ANOVA followed by Tukey's multiple‐comparisons test. Longitudinal tumor‐volume and body‐weight data were analyzed using two‐way repeated‐measures ANOVA followed by Šídák's multiple‐comparisons test.

To further assess the USP4 dependence of ASD activity, we performed complementary genetic epistasis and rescue experiments. In Huh7 cells, USP4 depletion reduced CHAF1B and SETDB1 abundance, AKT T308 phosphorylation, and the expression of the lipogenic proteins FASN, PLIN2, and SCD. ASD produced comparatively limited additional suppression of these molecular readouts in USP4‐depleted cells relative to its effects in sgNC cells (Figure S15A). Consistently, the additional inhibitory effects of ASD on EdU incorporation, colony formation, and Transwell migration were attenuated in USP4‐depleted cells (Figure S15B–D). Conversely, USP4‐WT overexpression increased CHAF1B and SETDB1 abundance, AKT T308 phosphorylation, and lipogenic protein expression in HepG2 cells. Under ASD treatment, USP4‐WT partially restored CHAF1B, SETDB1, p‐AKT(T308), FASN, PLIN2, and SCD, together with proliferative, clonogenic, and migratory phenotypes. Together, these genetic epistasis and USP4 rescue experiments support a USP4‐dependent component of ASD activity and indicate that ASD suppresses CHAF1B‐associated AKT T308 signaling and lipogenic phenotypes, at least in part, through perturbation of the USP4–CHAF1B axis. In vivo, ASD reduced tumor burden in the hydrodynamic Sleeping Beauty model, the subcutaneous xenograft model and the zebrafish xenograft model, whereas enforced CHAF1B expression partially reversed these effects (Figure 8L–N). These findings show that ASD suppresses tumor growth across multiple in vivo models and that enforced CHAF1B expression partially counteracts these effects.

## 3 Discussion

In the current study, we identify CHAF1B as a functionally relevant regulator of malignant HCC phenotypes and provide evidence for a noncanonical CHAF1B/UHRF1/SETDB1 regulatory module associated with AKT T308‐linked signaling and lipogenic remodeling. Across public cohorts, spatial transcriptomic datasets, and patient specimens, CHAF1B was consistently upregulated in HCC and associated with proliferation‐ and migration‐related programs. CHAF1B gain‐ and loss‐of‐function experiments further showed corresponding changes in proliferative, clonogenic, migratory, spheroid‐forming, and tumorigenic phenotypes. These effects were accompanied by altered SREBP1C nuclear localization, lipogenic gene and protein expression, and lipid‐droplet accumulation.

Mechanistically, the data support a functional regulatory model in which CHAF1B facilitates the association between UHRF1 and SETDB1. Domain mapping experiments showed that the deletion of the CHAF1B WD40 repeat‐containing region weakened its association with both SETDB1 and UHRF1, whereas deletion of the p150‐interaction region retained substantial binding capacity. Consistently, CHAF1B depletion reduced the endogenous UHRF1–SETDB1 association, and CHAF1B‐WT restored this association more effectively than CHAF1B‐ΔWD40. These findings support a contribution of the WD40 repeat‐containing region to the organization of the UHRF1–SETDB1 regulatory module. However, they do not establish the structural architecture of a stable scaffold complex. UHRF1 may have a noncanonical role within this regulatory module. Beyond its established function in DNMT1‐coupled maintenance methylation [25], UHRF1 associated with CHAF1B and SETDB1 and promoted K63‐dependent SETDB1 ubiquitination in the present experiments. Because UHRF1 loss‐of‐function, RING‐deficient mutants, and reconstituted in vitro ubiquitination assays were not examined, UHRF1 should be regarded as a candidate CHAF1B‐associated E3 ligase rather than a fully established SETDB1 E3 ligase. Pairwise Co‐IP and PLA experiments supported associations among CHAF1B, UHRF1, and SETDB1, whereas AKT and p‐AKT(T308) were not consistently recovered in the corresponding immunoprecipitates. AKT T308 phosphorylation is therefore interpreted as a downstream functional readout rather than evidence that AKT is a stable component of the CHAF1B–UHRF1–SETDB1 module.

The present data further distinguish SETDB1 protein abundance from its redistribution‐associated signaling competence. CHAF1B depletion reduced SETDB1 abundance and shortened its persistence in CHX‐chase assays, and MG132 partially restored SETDB1 protein, indicating increased susceptibility to proteasome‐dependent degradation. The additional loss‐of‐function linkage analysis provides a mechanistic explanation for this observation. Although K48‐linked ubiquitination did not show a reproducible increase, CHAF1B depletion preferentially enhanced K11‐associated ubiquitination of SETDB1, and the K11‐deficient HA‐Ub‐K11R mutant markedly attenuated this modification. K11‐linked ubiquitin chains are established proteasomal degradation signals in mammalian cells [26], supporting a predominant contribution of the K11‐associated ubiquitin state observed here to SETDB1 turnover. The degradative pathway was further linked to VHL. SETDB1 has previously been identified as a substrate of the CRL2/VHL E3 ubiquitin ligase complex, with hydroxylation‐dependent VHL recognition promoting SETDB1 ubiquitination and proteasomal degradation [24]. Extending this regulatory framework, our data show that CHAF1B depletion enhances the SETDB1–VHL association, whereas CHAF1B re‐expression attenuates this interaction. VHL depletion partially restores SETDB1 abundance and, importantly, suppresses the CHAF1B‐loss‐associated increase in K11‐associated SETDB1 ubiquitination. These findings support a model in which CHAF1B loss shifts SETDB1 toward a VHL‐dependent, predominantly K11‐associated degradative ubiquitin state. In parallel, CHAF1B facilitates UHRF1‐associated K63‐dependent ubiquitination, supporting a non‐degradative, redistribution‐competent SETDB1 state. Thus, the VHL/K11‐associated degradative pathway is primarily linked to SETDB1 proteasomal turnover, whereas the CHAF1B/UHRF1/K63‐dependent regulatory pathway supports SETDB1 persistence, cytoplasmic redistribution, and signaling competence associated with AKT T308‐linked signaling.

SETDB1 catalytic activity provided a further connection between this regulatory module and AKT T308‐linked signaling [27]. AKT immunoprecipitation followed by pan‐methyl‐lysine immunoblotting showed reduced AKT methylation after CHAF1B depletion. SETDB1‐WT restored AKT methylation and AKT T308 phosphorylation more effectively than the catalytically inactive SETDB1‐H1224K mutant. These data support a contribution of SETDB1 catalytic activity to CHAF1B‐associated AKT methylation and AKT T308 phosphorylation. However, the pan‐methyl‐lysine assay does not identify the relevant AKT methylation site, and the molecular interface connecting cytoplasmic SETDB1 to AKT or upstream AKT regulators remains to be established. In addition, total AKT and AKT S473 phosphorylation were comparatively less affected in the experimental systems used. The data therefore support preferential association with AKT T308‐linked signaling but do not establish that AKT S473 is dispensable.

CHAF1B‐associated AKT T308 phosphorylation was accompanied by increased mTORC1‐associated readouts, SREBP1C nuclear localization, and expression of FASN, ACACA, SCD, and PLIN2. LY294002 attenuated these molecular changes together with lipid‐droplet accumulation and malignant phenotypes. SREBP1C knockdown further reduced lipogenic markers and lipid‐droplet accumulation and partially suppressed proliferation, clonogenic growth, and migration. These observations support a functional contribution of SREBP1C‐associated lipogenic remodeling to CHAF1B‐associated phenotypes. The partial attenuation of proliferation, clonogenic growth, and migration indicates that SREBP1C‐dependent lipogenesis contributes to, but is not the sole or indispensable mediator of, CHAF1B‐associated aggressiveness.

These findings extend the known functions of CHAF1B beyond its classical role in the CAF‐1 complex. CAF‐1 mediates replication‐coupled nucleosome assembly through CHAF1A/p150, CHAF1B/p60, and RBBP4/p48 and contributes to chromatin restoration and genome maintenance [8, 28, 29]. The ASF1A‐binding‐deficient CHAF1B RR482/483AA mutant retained the ability to regulate SETDB1 abundance, SETDB1 ubiquitination, and AKT T308 phosphorylation. This suggests that the SETDB1‐associated regulatory function is not strictly dependent on the tested ASF1A‐binding interface. Nevertheless, this result does not exclude contributions from other CAF‐1‐related activities, including CHAF1A/RBBP4‐dependent nucleosome assembly, replication‐coupled chromatin restoration, or broader chromatin‐state regulation.

The clinical data were consistent with the experimental findings. CHAF1B and SETDB1 protein expression was elevated in HCC tissues, and concurrent high expression was associated with unfavorable survival across the tissue microarray and public cohorts. The hydrodynamic c‐Myc/myr‐AKT model further showed that CHAF1B cooperated with an established oncogenic background to enhance tumor formation. Because constitutively active AKT was introduced in this model, these results support in vivo cooperation rather than proving that CHAF1B acts upstream of AKT.

We further identified USP4 as an upstream regulator of CHAF1B protein stability. USP4 depletion increased K48‐linked ubiquitination, accelerated CHAF1B turnover, and reduced CHAF1B abundance without materially changing CHAF1B mRNA. Conversely, USP4 overexpression increased CHAF1B protein abundance. The positive correlation between USP4 and CHAF1B IHC scores in human HCC tissues supports the clinical relevance of this post‐translational regulatory layer. These findings do not exclude transcriptional mechanisms contributing to CHAF1B upregulation; rather, they suggest that transcriptional dysregulation and USP4‐associated protein‐stability control may jointly influence CHAF1B abundance.

ASD was evaluated as a pharmacologic probe of the USP4–CHAF1B axis. ASD altered USP4 thermal stability in CETSA, increased K48‐linked ubiquitination of CHAF1B, accelerated CHAF1B degradation, and suppressed CHAF1B‐associated malignant and lipogenic phenotypes. USP4‐depletion epistasis and USP4‐WT rescue experiments supported a USP4‐dependent component of ASD activity. CHAF1B overexpression also partially counteracted the effects of ASD in vitro and in vivo. Nevertheless, these results do not establish ASD as a selective USP4 inhibitor. Direct biochemical inhibition of USP4, broader selectivity across deubiquitinases, pharmacodynamic biomarkers, toxicity, and in vivo genetic on‐target dependence remain to be established.

From a therapeutic perspective, modulation of USP4‐dependent CHAF1B stability may provide a complementary approach in tumors with high USP4/CHAF1B expression. However, the present data do not demonstrate that targeting USP4 or CHAF1B is preferable to direct PI3K/AKT pathway inhibition. LY294002 attenuated CHAF1B‐associated signaling, lipogenic readouts, and malignant phenotypes, supporting PI3K/AKT signaling as a functional downstream component. Future studies should compare USP4/CHAF1B‐directed approaches with clinically relevant PI3K/AKT inhibitors and evaluate rational combinations with SETDB1‐, UHRF1‐, or lipid‐metabolism‐directed interventions.

This study has several limitations. The architecture and dynamics of the CHAF1B–UHRF1–SETDB1 regulatory module remain to be characterized using approaches such as crosslinking mass spectrometry, proximity‐dependent labeling, or structural analysis. The molecular basis by which the CHAF1B WD40 repeat‐containing region facilitates UHRF1–SETDB1 association and favors K63‐dependent ubiquitination remains unclear. How K63‐dependent ubiquitination influences CRM1/exportin‐1‐dependent SETDB1 redistribution also requires further study. The relevant AKT methylation sites and their functional requirements remain to be systematically defined [30]. Although the current data support a VHL‐dependent, predominantly K11‐associated degradative ubiquitin state of SETDB1 after CHAF1B depletion, the precise ubiquitin‐chain architecture remains to be fully defined. Similarly, the molecular basis by which CHAF1B loss enhances SETDB1–VHL association remains to be established. Future work combining targeted lipidomics with isotope‐tracing analysis will be needed to quantify metabolic flux and define the contribution of individual lipid pathways. Finally, the ASF1A‐binding‐deficient RR482/483AA mutant tests only one CAF‐1‐related interface and does not exclude broader chromatin‐assembly‐related contributions.

In conclusion, the present findings support a noncanonical CHAF1B–UHRF1–SETDB1 regulatory module associated with AKT T308‐linked signaling and lipogenic remodeling in HCC. Through its WD40 repeat‐containing region, CHAF1B facilitates association with UHRF1 and SETDB1 and supports K63‐dependent ubiquitination and cytoplasmic redistribution of SETDB1. Conversely, CHAF1B depletion enhances SETDB1 association with VHL and favors a predominantly K11‐associated degradative ubiquitin state linked to proteasomal SETDB1 loss. USP4 contributes to CHAF1B protein stability by limiting K48‐linked ubiquitination, while ASD pharmacologically perturbs this regulatory pathway in experimental models (Figure S16). Together, these findings identify ubiquitin‐dependent mechanisms governing SETDB1 abundance and support the USP4–CHAF1B–UHRF1–SETDB1 network as a candidate regulatory framework for further mechanistic and therapeutic investigation.

## 4 Experimental Section

### 4.1 Human HCC Samples and Tissue Microarray Analysis

Human HCC specimens were obtained from patients who underwent surgical resection at the Affiliated Hospital of Nantong University. All tumor samples were pathologically confirmed as hepatocellular carcinoma by histological examination. In this study, 24 paired freshly frozen HCC and adjacent non‐tumor liver tissues were used for western blotting and RT‐qPCR validation of CHAF1B expression. In addition, formalin‐fixed, paraffin‐embedded HCC specimens, including a tissue microarray cohort comprising 158 HCC cases with available clinicopathological information, were used for immunohistochemical evaluation of CHAF1B, SETDB1, and USP4 expression and for clinicopathological and survival analyses. Representative paired HCC and adjacent non‐tumor liver tissues were also used for IHC and multiplex immunofluorescence staining. The study involving human HCC specimens and retrospective clinical data was approved by the Ethics Committee of the Affiliated Hospital of Nantong University (Approval No. 2025‐L186) and was conducted in accordance with the Declaration of Helsinki. The requirement for informed consent was waived by the Ethics Committee because the study involved retrospective analysis of de‐identified specimens and clinical data. Preoperative liver MRI examinations were retrospectively retrieved from the institutional picture archiving and communication system for patients included in the HCC tissue cohort who had available diagnostic‐quality imaging. MRI examinations performed within two months before surgical resection were considered. Patients who had received locoregional or systemic antitumor treatment before MRI were excluded. Tumoral CHAF1B and SETDB1 expression status was determined from the corresponding resected tissue specimens using the predefined IHC scoring criteria. Representative MRI images were selected from the indicated CHAF1B/SETDB1 expression groups based on image availability and adequate diagnostic quality.

### 4.2 Immunohistochemistry and Immunofluorescence

IHC was conducted following a standardized two‐step staining protocol. Detection was achieved using horseradish peroxidase (HRP)‐conjugated secondary antibodies and 3,3'‐diaminobenzidine (DAB; Sigma‐Aldrich). For immunofluorescence, fluorescein isothiocyanate (FITC)‐conjugated secondary antibodies and DAPI (Sigma) were used. Multiplex immunofluorescence was performed using a four‐color multiplex immunofluorescence kit (Absin, abs50012‐20T) according to the manufacturer's instructions. Imaging was conducted using a laser‐scanning confocal microscope (Leica, 400× magnification), and fluorescence intensity was quantified using ImageJ software. Images within the same experiment were acquired using identical microscope settings. Background fluorescence was subtracted using cell‐free regions, and the same threshold was applied to all groups within an experiment. At least five randomly selected fields were analyzed per condition. Fluorescence intensity or positive area was normalized to the number of DAPI‐positive nuclei, as appropriate for the corresponding assay. Protein expression in HCC tissue specimens was quantified using the Vectra Automated Quantitative Pathology Imaging System (version 3.0; PerkinElmer, USA). IHC staining was evaluated based on staining intensity and the percentage of positively stained tumor cells. Staining intensity was scored as 0 (negative), 1 (weak), 2 (moderate), or 3 (strong), while the percentage of positive tumor cells was recorded on a scale from 0% to 100%. The final IHC score was calculated by multiplying the staining‐intensity score by the percentage of positive tumor cells, resulting in a score ranging from 0 to 300. For categorical analyses, patients were classified into high‐ and low‐expression groups according to the median IHC score of the corresponding protein in the evaluable HCC cohort. The same predefined cutoff was applied in the clinicopathological and survival analyses.

### 4.3 Sleeping Beauty (SB) Hydrodynamic Transposition Model

Seven‐week‐old male C57BL/6 mice were randomly assigned to the indicated experimental groups (n = 3 mice per group). Hepatic tumors were induced by hydrodynamic tail‐vein delivery of Sleeping Beauty transposons as previously described [31]. Coding sequences for human CHAF1B, c‐Myc, and constitutively active AKT1 (myr‐AKT1) were cloned into pT2 transposon backbones and mobilized using the pCMV‐SB100X transposase construct. For each mouse, the plasmid mixture was freshly prepared in sterile saline immediately before injection. In the CHAF1B co‐expression experiment, mice received pT2‐c‐Myc, pT2‐myr‐AKT1, and pT2‐CHAF1B, whereas control mice received pT2‐c‐Myc, pT2‐myr‐AKT1, and pT2‐empty vector to ensure an equivalent total amount of transposon DNA. A total of 20 µg of pT2 transposon plasmids was administered per mouse, and pCMV‐SB100X was included at an SB100X‐to‐transposon mass ratio of 1:5. The plasmid mixture was diluted in sterile saline to a final volume corresponding to 10% of body weight and injected through the lateral tail vein within 5 s. Tumor development was monitored by in vivo bioluminescence imaging once weekly for 4 consecutive weeks after hydrodynamic injection. Mice were euthanized at week 5. Livers were collected, photographed, and weighed, and tumor burden was assessed using the liver‐to‐body‐weight ratio. Liver tissues were processed for hematoxylin and eosin staining and immunohistochemical analysis of CHAF1B and SETDB1, with the remaining tissues snap‐frozen for protein analysis. All animal procedures were reviewed and approved by the Animal Care and Use Committee of Nantong University (approval no. P20250305‐010).

### 4.4 Cell Lines and Culture Conditions

Human hepatocellular carcinoma‐derived Huh7 (catalog no. ZQ0025; RRID: CVCL_0336), MHCC97H (catalog no. ZQ0020; RRID: CVCL_4972), and HCCLM3 cells (catalog no. ZQ0023; RRID: CVCL_6832); the hepatoblastoma‐derived hepatic tumor cell line HepG2 (catalog no. ZQ0022; RRID: CVCL_0027); the endothelial‐like liver tumor‐associated cell line SK‐Hep1 (catalog no. ZQ0030; RRID: CVCL_0525); and human embryonic kidney‐derived cell line HEK‐293T (catalog no. ZQ0033; RRID: CVCL_0063), were obtained from Shanghai Zhong Qiao Xin Zhou Biotechnology Co., Ltd. in June 2023. All cell lines were cultured in high‐glucose DMEM supplemented with 10% fetal bovine serum and maintained at 37°C in a humidified incubator containing 5% CO_2_. Cells were maintained under logarithmic growth conditions and used at passages below 50. Cell‐line identities were authenticated by Shanghai Zhong Qiao Xin Zhou Biotechnology Co., Ltd. using short tandem repeat profiling before release. The STR profiles were matched against the corresponding reference profiles and met the supplier's authentication criteria, with no evidence of cell‐line misidentification or cross‐contamination. Mycoplasma contamination was assessed in all cell lines at the latest passages used in this study using a validated mycoplasma detection assay, and all cultures tested negative. SK‐Hep1 was included as a complementary aggressive endothelial‐like liver tumor model. HepG2 was used as a hepatic tumor gain‐of‐function model because of its relatively low endogenous CHAF1B abundance and suitability for stable overexpression. HEK‐293T cells were used only for experiments requiring high transient‐transfection efficiency, including plasmid‐expression, ubiquitination, and protein‐interaction assays.

### 4.5 Western Blotting

Cells or tissue samples were lysed in RIPA buffer containing protease and phosphatase inhibitor cocktails on ice for 30 min. Lysates were centrifuged at 12 000 ×*g* for 15 min at 4°C, and the supernatants were collected. Protein concentrations were determined using a BCA protein assay kit. Equal amounts of protein, typically 30 µg per lane, were separated by SDS‐PAGE and transferred onto PVDF membranes. Membranes were blocked with 5% BSA for 1 h at room temperature and incubated with primary antibodies overnight at 4°C, followed by HRP‐conjugated secondary antibodies for 1 h at room temperature. Protein bands were visualized using enhanced chemiluminescence. Densitometric analysis was performed using ImageJ. In conventional whole‐cell lysate blots, both phosphorylated and total protein signals were independently normalized to β‐actin. For nuclear and cytoplasmic fractionation experiments, protein signals were normalized to Lamin B1 and α‐tubulin, respectively. Within each cell line and experiment, the value of the corresponding control group was set to 1.00. Detailed antibody information is provided in Table S1.

### 4.6 Co‐Immunoprecipitation (Co‐IP)

For Co‐IP assays, cells were lysed in ice‐cold NP‐40 lysis buffer containing 50 mM Tris‐HCl, pH 7.5, 150 mM NaCl, 1% NP‐40, 1 mM EDTA, 10% glycerol, and protease and phosphatase inhibitor cocktails. Cell lysates were cleared by centrifugation at 12 000 ×*g* for 15 min at 4°C. For each immunoprecipitation, 100 µg of total protein was incubated with 4 µg of the indicated antibody or control IgG overnight at 4°C with rotation, followed by incubation with Protein A/G agarose beads for 2 h. Beads were washed four times with lysis buffer and eluted in SDS sample buffer. Immunocomplexes were analyzed by western blotting.

For AKT methylation analysis, cell lysates were immunoprecipitated with anti‐AKT antibody, and methylated AKT was detected by immunoblotting with a pan‐methyl‐lysine antibody. For SETDB1 catalytic rescue experiments, CHAF1B‐depleted cells were transfected with SETDB1‐WT or the catalytically inactive SETDB1‐H1224K mutant, followed by analysis of AKT methylation, p‐AKT(T308), total AKT, and downstream signaling proteins.

### 4.7 Immunoprecipitation–Mass Spectrometry

To identify CHAF1B‐associated proteins, endogenous CHAF1B immunocomplexes were isolated from Huh7 and SK‐Hep1 cells. Cells were lysed in NP‐40 lysis buffer containing protease and phosphatase inhibitor cocktails. Equal amounts of clarified protein lysates were incubated overnight at 4°C with anti‐CHAF1B antibody or species‐matched control IgG, followed by incubation with Protein A/G agarose beads. After extensive washing, bound proteins were eluted, subjected to tryptic digestion, and analyzed by liquid chromatography–tandem mass spectrometry. Raw data were processed using Proteome Discoverer and searched against the UniProt human reference proteome. Peptide and protein identifications were filtered at a false‐discovery rate of < 1%. Candidate interactors were selected based on detection in CHAF1B immunoprecipitates and enrichment relative to the corresponding IgG controls.

### 4.8 Focused CRISPR‐Based DUB Screen

A focused CRISPR‐based screen targeting 90 human deubiquitinase genes was performed to identify regulators of CHAF1B protein abundance. For each target gene, two independent sgRNAs were included and used together as a pooled sgRNA pair. Cas9‐expressing Huh7 cells were transduced with the corresponding pooled sgRNAs or a non‐targeting sgRNA control. Following transduction, cells were selected with 2 µg/mL puromycin for 48 h. CHAF1B protein abundance was then evaluated by western blotting and normalized to β‐actin. Candidate DUBs were defined as genes whose depletion reproducibly altered CHAF1B protein abundance relative to the sgNC control. USP4 was prioritized for subsequent validation because its depletion consistently reduced CHAF1B protein abundance. The sgUSP4‐1 and sgUSP4‐2 target sequences are listed in Table S2.

### 4.9 Untargeted Metabolomics

HepG2 cells stably overexpressing CHAF1B (OE‐CHAF1B) and matched vector controls (three biological replicates per group; total *n* = 6) were harvested, and 1‐mL aliquots of each extract were freeze‐dried. Pellets were resuspended in pre‐chilled 80% methanol, vortexed thoroughly, incubated on ice for 5 min, and centrifuged at 15 000 ×*g* and 4°C for 15 min. Supernatants were diluted with LC–MS–grade water to a final 53% methanol, transferred to fresh tubes, and centrifuged again at 15 000 ×*g* and 4°C for 15 min. The clarified supernatants were injected for UHPLC–MS/MS analysis on a Vanquish UHPLC system (Thermo Fisher, Germany) coupled to an Orbitrap Q Exactive HF mass spectrometer (Thermo Fisher, Germany) at GeneChem Co., Ltd. (Shanghai, China). Data were acquired in both electrospray positive‐ion (ESI+) and negative‐ion (ESI−) modes. Raw files were processed in Compound Discoverer 3.3 (Thermo Fisher) for peak alignment, peak picking, and integration, followed by putative metabolite annotation against KEGG, HMDB, and LIPID MAPS. Multivariate analyses including PCA and PLS‐DA were performed with metaX. Univariate statistics used two‐sided *t*‐tests, and differential metabolites were defined by VIP > 1.0, *p* < 0.05, and fold change ≥ 1.2 or ≤ 0.833. Volcano plots were generated from log2(fold change) and –log10(P) using ggplot2 in R. For clustering heatmaps, intensity values of differential metabolites were z‐score normalized and plotted with pheatmap. Pairwise correlations among differential metabolites were calculated using Pearson's correlation coefficient in R. The statistical significance of each correlation was assessed using two‐sided correlation tests, and correlation matrices were visualized using the corrplot R package. Correlations with *p* < 0.05 were considered statistically significant.

### 4.10 DIA Quantitative Proteomics

Huh7 cells stably expressing shCHAF1B or shNC (3 biological replicates per group; total *n* = 6) were processed by Biotree (Shanghai, China). Cells were lysed in RIPA buffer with protease/phosphatase inhibitors, sonicated on ice, and clarified by centrifugation (12 000 rpm, 10 min, 4°C). Protein concentrations were determined by BCA. For each sample, aliquots were reduced, alkylated, and digested with trypsin at 37°C. Peptides were desalted on C18. Two hundred nanograms of peptides were separated on a nanoElute2 UPLC with a PePSep C18 column using 0.1% formic acid in H_2_O and 0.1% formic acid in acetonitrile, and analyzed on a timsTOF Pro2 (Bruker) operated in DIA‐PASEF mode. Raw files were processed in Spectronaut (v19) using the Pulsar engine and searched against the UniProt human reference. Intensities were globally normalized and log_2_‐transformed. Group separation and differential analysis used two‐sided Student's *t*‐test; proteins with *p* < 0.05 and fold change ≥ 1.2 or ≤ 0.833 were considered differentially expressed. GSEA on GO Biological Process categories was performed on the ranked protein list to infer CHAF1B‐regulated pathways.

### 4.11 RNA Extraction and Quantitative Real‐Time PCR

Total RNA was extracted using TRIzol reagent (Invitrogen), and 500 ng RNA was reverse transcribed into cDNA. Quantitative PCR was performed using TB Green Premix Ex Taq∖ II (Takara) on a QuantStudio Real‐Time PCR System (Thermo Fisher). Gene expression levels were normalized to β‐actin and analyzed using the 2^−^^∆∆Ct^ method. The primer sequences used for RT‐qPCR are listed in Table S3.

### 4.12 Plasmids and Cell Transfection

Expression constructs encoding CHAF1B‐WT, CHAF1B‐ΔWD40, CHAF1B‐Δp150, CHAF1B‐RR482/483AA, SETDB1‐WT, SETDB1‐H1224K, UHRF1, and USP4‐WT were generated in the indicated mammalian expression vectors. HA‐tagged ubiquitin constructs included HA‐Ub‐WT, linkage‐restricted HA‐Ub‐K6, HA‐Ub‐K11, HA‐Ub‐K27, HA‐Ub‐K29, HA‐Ub‐K33, HA‐Ub‐K48, and HA‐Ub‐K63, together with the HA‐Ub‐K11R, HA‐Ub‐K48R and HA‐Ub‐K63R mutants. CHAF1B shRNA and USP4 sgRNA constructs and their corresponding control constructs were generated using the indicated targeting sequences. SETDB1‐WT and SETDB1‐H1224K constructs were generated in the pcDNA3.1 vector. Transfections were carried out using Lipofectamine 2000 (Invitrogen) according to the manufacturer's protocol. The shRNA, siRNA, and sgUSP4 sequences used for downstream validation are provided in Table S2.

### 4.13 Cell Migration Assay

Migration assays were conducted using 24‐well transwell chambers (8.0 µm pore size; Corning). A total of 1 × 10^5^ cells in 100 µL of DMEM containing 1% FBS were seeded into the upper chambers. The lower chambers contained 600 µL DMEM with 10% FBS. After 24 h, migrated cells were fixed with 4% paraformaldehyde and stained with 0.1% crystal violet. Quantification was performed using ImageJ.

### 4.14 Cell Proliferation and Colony Formation Assays

Cell proliferation was assessed using the CCK‐8 assay (Dojindo) according to the manufacturer's instructions. Absorbance was measured at 450 nm. For colony formation assays, 500 cells per well were seeded into 6‐well plates and cultured for 10 days. Colonies were fixed with paraformaldehyde, stained with crystal violet, and quantified microscopically.

### 4.15 EdU Incorporation Assay

Cell proliferation was further assessed using an EdU incorporation assay kit (Beyotime, C0078S) according to the manufacturer's instructions. Cells were seeded onto coverslips in 24‐well plates at a density of 2 × 10^4^ cells per well and subjected to the indicated genetic or pharmacologic treatments. Cells were incubated with 10 µM EdU for 2 h at 37°C, fixed with 4% paraformaldehyde for 15 min, and permeabilized with Triton X‐100. The click‐reaction mixture was then applied, and nuclei were counterstained with DAPI. Images were acquired using a confocal fluorescence microscope under identical settings within each experiment. The percentage of EdU‐positive cells was calculated as the number of EdU‐positive nuclei divided by the total number of DAPI‐positive nuclei. At least five randomly selected fields were analyzed per condition in each of three independent experiments.

### 4.16 Three‐Dimensional Spheroid Formation Assay

Three‐dimensional spheroid formation assays were performed using ultra‐low‐attachment 96‐well U‐bottom plates. Cells were seeded at 1 × 10^3^ cells per well in complete spheroid‐forming medium, followed by the indicated genetic manipulation or pharmacologic treatment. Spheroids were maintained at 37°C in a humidified atmosphere containing 5% CO_2_ and imaged on days 3, 5, and 7 using an inverted microscope. The longest diameter (L) and the perpendicular diameter (W) of each spheroid were measured using ImageJ. Spheroid volume was calculated using the formula V = π/6 × L × W^2^. Three independently cultured spheroids from separate wells were analyzed per group. Each data point represents one spheroid. For whole‐mount Ki‐67 immunofluorescence, spheroids were fixed, permeabilized, blocked, and stained using the antibody conditions described in the Cellular Immunofluorescence section.

### 4.17 Nile Red and Oil Red O Staining

For Nile Red staining, cells were seeded onto coverslips and subjected to the indicated treatments. Cells were washed with phosphate‐buffered saline, fixed with 4% paraformaldehyde for 30 min, and incubated with Nile Red (1:1000, Sigma) for 30 min at 37°C in the dark. Nuclei were counterstained with DAPI. Fluorescence images were acquired using identical microscope settings within each experiment. For Oil Red O staining, freshly frozen liver tissues were sectioned at 5 µm, fixed in 4% paraformaldehyde for 30 min, and stained with freshly prepared Oil Red O working solution (Oil Red O: H_2_O = 3:2, meilunbio, MA0120) for 15 min. Sections were differentiated and washed according to the staining protocol. Images were acquired using a light microscope.

### 4.18 Cycloheximide‐Chase Assay

Protein stability was assessed by cycloheximide‐chase analysis. Following the indicated genetic manipulation or pharmacologic pretreatment, cells were exposed to cycloheximide at 100 µg/mL to inhibit de novo protein synthesis. Cells were harvested at 0, 2, 4, and 8 h after cycloheximide addition. Whole‐cell lysates were analyzed by western blotting for CHAF1B or SETDB1, as indicated. Band intensities were normalized to β‐actin and then expressed relative to the corresponding signal at 0 h, which was set to 1.00. Protein‐decay curves were generated from three independent experiments.

### 4.19 Nuclear and Cytoplasmic Fractionation

Nuclear and cytoplasmic fractions were prepared using a Nuclear and Cytoplasmic Extraction Kit (Beyotime, P0027) according to the manufacturer's instructions. Briefly, cells were harvested in ice‐cold phosphate‐buffered saline and lysed in cytoplasmic extraction buffer. Following centrifugation at 12 000 ×*g* for 10 min, the supernatant was collected as the cytoplasmic fraction. The nuclear pellet was washed and subsequently lysed in nuclear extraction buffer. Equal percentages of the total nuclear and cytoplasmic fractions were loaded for western blotting. Lamin B1 and α‐tubulin were used as nuclear and cytoplasmic fraction markers, respectively. The distribution of SETDB1 was quantified by densitometry and normalized to the corresponding compartment‐specific loading control.

### 4.20 Pharmacologic Treatments

Akebia saponin D, LY294002, leptomycin B, MG132, chloroquine, 3‐methyladenine, and cycloheximide were dissolved in the corresponding solvent and stored according to the manufacturers’ instructions. Vehicle‐control cells received the same final concentration of solvent. For the indicated in vitro experiments, ASD was used at 10 or 20 µM for 24 h, unless otherwise specified. For CETSA, Huh7 cells were treated with 100 µM ASD for 12 h. LY294002 was used at 10 µM for 24 h to inhibit PI3K‐dependent signaling. Leptomycin B was used at 100 nM for 24 h to inhibit CRM1/exportin‐1‐dependent nuclear export. MG132 was used at 10 µM for 6 h before cell collection. Chloroquine was used at 10 µM for 6 h, and 3‐methyladenine was used at 10 µM for 6 h. Cycloheximide was used at 100 µg/mL for the indicated chase periods.

### 4.21 Datasets Processing

Public datasets included TCGA‐LIHC, TCPA, and five independent GEO HCC cohorts (GSE46408, GSE60502, GSE67764, GSE147888, and GSE207435). GSE14520 was additionally used as an independent HCC cohort for survival validation. Proteomic correlation was analyzed in Proteomic Data Commons (PDC, https://pdc.cancer.gov/pdc/; study ID: PDC000198) [32]. RNA‐seq differential expression was analyzed using DESeq2, whereas microarray datasets were analyzed using limma after quantile normalization. Significance was set at *p* < 0.05 and |log2FC| ≥ 1. Cross‐study candidates required concordant upregulation across discovery GEO sets and verification in TCGA/TCPA. Gene set enrichment analysis (GSEA) used genome‐wide ranks by DE statistic against MSigDB Hallmark and GO‐BP (1,000 permutations). Gene sets with nominal *p* < 0.05 were considered significantly enriched. Publicly available human HCC 10x Genomics Visium datasets were processed using Space Ranger and Seurat [33, 34].

### 4.22 Subcutaneous and Orthotopic Xenograft Models

For the subcutaneous xenograft model, 5 × 10^6^ HepG2 cells stably expressing CHAF1B or the corresponding vector control were suspended in 150 µL of PBS without Matrigel and injected subcutaneously into the left axillary region of four‐week‐old male BALB/c nude mice. Mice were randomly assigned to the indicated groups, with five mice per group. Four weeks after cell inoculation, the mice were euthanized, and the tumors were excised, photographed, and weighed. For the orthotopic xenograft model, 1 × 10^5^ Huh7‐luc‐GFP cells stably expressing shNC or shCHAF1B were suspended in 50 µL of PBS and injected into the left hepatic lobe of six‐week‐old male BALB/c nude mice. Mice were randomly assigned to the indicated groups. Tumor growth was monitored by bioluminescence imaging using the IVIS Spectrum In Vivo Imaging System (PerkinElmer), and endpoint mean radiant efficiency was quantified using Living Image software. Mice were euthanized at 4 weeks post injection, after which the livers were collected, weighed, photographed, and processed for subsequent analyses. Mouse body weight was recorded at baseline and throughout the experimental period. For subcutaneous xenograft models, tumor dimensions were measured longitudinally, and tumor weights were recorded at sacrifice. For orthotopic and multifocal Sleeping Beauty liver‐tumor models above, tumor burden was assessed using bioluminescence imaging and endpoint gross liver examination or the liver‐to‐body‐weight ratio because individual intrahepatic tumor volumes and isolated tumor weights could not be reliably determined.

### 4.23 Zebrafish Xenograft Model

The assay was conducted as previously reported [35]. Zebrafish xenograft assays were performed using two transgenic reporter lines. For the CHAF1B gain‐ and loss‐of‐function experiments, the LY294002 intervention experiment, and the ASD rescue experiment, Tg(fli1:EGFP) embryos were used, and HepG2 or Huh7 cells were labeled with CellTrace Far Red before injection. For the SETDB1 rescue experiment, Tg(kdrl:ras‐mCherry) embryos and Huh7‐GFP cells were used. At 48 hours post‐fertilization, embryos were anesthetized with 0.04% tricaine. Approximately 500 tumor cells suspended in 5 nL were injected into the duct of Cuvier using a microinjector. After injection, embryos were maintained at 34°C to support tumor‐cell survival and engraftment. Fluorescence images were acquired at 1, 2, and 3 days post‐injection using a confocal fluorescence microscope. Tumor burden was quantified as fluorescent tumor area using ImageJ under identical acquisition and analysis settings within each experiment. Embryos with injection artifacts, abnormal development, or unsuccessful engraftment were excluded before quantitative analysis.

### 4.24 ASD Treatment in Mouse and Zebrafish Models

For the Sleeping Beauty hydrodynamic model, seven‐week‐old male C57BL/6 mice were randomly assigned to the indicated vehicle‐ and ASD‐treatment groups (*n* = 3 mice per group). ASD was formulated in 10% DMSO, 40% PEG300, 5% Tween 80, and 45% saline and administered intraperitoneally at 20 mg/kg once weekly for five consecutive weeks after hydrodynamic injection. Vehicle‐treated mice received an equivalent volume of the same formulation without ASD. Tumor development was monitored by weekly bioluminescence imaging, and mice were euthanized at week 5. Livers were collected, weighed, photographed, and processed for histological and molecular analyses. For the subcutaneous xenograft model, 5 × 10^6^ luciferase‐expressing Huh7 cells suspended in 150 µL of PBS without Matrigel were injected subcutaneously into the left axillary region of four‐week‐old male BALB/c nude mice obtained from the Animal Center of Nantong University. Mice with established tumors of approximately 100 mm^3^ were randomly assigned to the indicated treatment groups on Day 5 after tumor‐cell inoculation. Baseline tumor volume was measured immediately before treatment initiation, after which mice received ASD at 20 mg/kg by intraperitoneal injection once weekly for four consecutive weeks. Tumor length and width were measured at the indicated intervals, and tumor volume was calculated as length × width^2^/2. At the endpoint, bioluminescence imaging was performed using the IVIS Spectrum system, and mean radiant efficiency was quantified using Living Image software. Tumors were subsequently excised, weighed, and processed for subsequent analyses. For zebrafish drug‐treatment experiments, embryos bearing HCC xenografts were exposed to ASD at 10 µM in embryo medium beginning 6 h post‐injection. Drug‐containing medium was renewed every 24 h, and vehicle‐control embryos received the same final concentration of the corresponding solvent.

### 4.25 Cellular Immunofluorescence

Cells grown on coverslips were fixed in 4% paraformaldehyde, permeabilized with 0.1% Triton X‐100, and blocked with 5% goat serum. Cells were incubated overnight at 4°C with primary antibodies, followed by fluorescent secondary antibodies at 37°C. Nuclei were stained with DAPI, and images were acquired using a confocal microscope.

### 4.26 Cellular Thermal Shift Assay

Cellular thermal shift assay (CETSA) was performed to assess cellular engagement of USP4 by ASD. Huh7 cells were treated with ASD at 100 µM for 12 h before collection. Cell suspensions were divided into equal aliquots and heated at 37°C, 42°C, 47°C, 52°C, 57°C, 62°C, and 65°C for 3 min. After lysis and centrifugation, soluble protein fractions were analyzed by western blotting for USP4. Changes in USP4 thermal stability were interpreted as evidence of cellular target engagement.

### 4.27 Ubiquitination Assay

The ubiquitination assay was conducted as previously reported [36]. HEK‐293T or HCC cells were treated with 10 µM MG132 (TargetMol) for 6 h prior to harvest. After transfection with the indicated constructs, cell lysates were immunoprecipitated with anti‐SETDB1, anti‐FLAG, or the indicated target‐protein antibody. Target‐associated ubiquitination was detected by immunoblotting with anti‐HA. Where indicated, ubiquitination signals were normalized to the amount of immunoprecipitated target protein. Where indicated, VHL was depleted using siVHL before SETDB1 ubiquitination or co‐immunoprecipitation analyses.

### 4.28 Proximity Ligation Assay (PLA)

PLA was performed using the Duolink In Situ Red Kit (Sigma‐Aldrich) according to the manufacturer's protocol. Briefly, cells were incubated with primary antibodies (1:2000), followed by PLA probes and rolling circle amplification. DAPI was used for nuclear staining. Fluorescence signals were imaged using a confocal microscope, and foci were quantified using CellProfiler pipelines.

### 4.29 Animal Welfare Monitoring

Mice were monitored throughout the experimental period for body weight and general welfare. Body weight was recorded at baseline and at the indicated longitudinal time points. Welfare‐related observations included premature death, the need for early euthanasia, visible tumor‐related abnormalities, and other clinically apparent abnormalities during routine observation. No premature deaths, early euthanasia events, or visible tumor‐related abnormalities were recorded in the analyzed cohorts, and no animal reached a humane endpoint before the scheduled experimental termination. Animals were euthanized at the prespecified experimental endpoints described above, after which tumors or livers were collected for the indicated downstream analyses. Longitudinal body‐weight data are summarized in Figure S17, whereas individual‐animal body‐weight measurements and welfare‐related observations are provided in Data S1.

### 4.30 Statistical Analysis

Statistical analyses were performed using GraphPad Prism version 10.1.2. Assay‐specific data preprocessing and normalization procedures are described in the corresponding subsections of the Experimental Section. Western blot signals were normalized to the indicated loading controls or immunoprecipitated target proteins, RT–qPCR data were analyzed using the 2^−ΔΔCt^ method, and metabolomic and proteomic data were normalized and transformed as described above. No data points were excluded solely on the basis of statistical outlier testing. Data are presented as mean ± SD unless otherwise indicated. The experimental unit and exact sample size, including the numbers of independent biological replicates, patients, animals, spheroids, or other experimental units, are provided in the corresponding subsections of the Experimental Section or figure legends. Parametric tests were used for prespecified comparisons of continuous data. For datasets with sufficient sample sizes, distributional characteristics and homogeneity of variance were evaluated before parametric testing. Formal normality testing was not routinely performed for experiments with only three biological replicates because of the limited statistical power of such tests at small sample sizes. Comparisons between two independent groups were performed using unpaired two‐sided Student's t‐tests, whereas paired tumor and adjacent non‐tumor specimens were analyzed using paired two‐sided Student's t‐tests. Comparisons among three or more groups were performed using one‐way ANOVA followed by Tukey's multiple‐comparisons test. Longitudinal tumor‐volume and body‐weight data obtained from the same animals were analyzed using two‐way repeated‐measures ANOVA followed by Šídák's multiple‐comparisons test. Pearson correlation analysis was used for continuous variables with approximately linear relationships, whereas Spearman rank correlation analysis was used for ordinal or non‐normally distributed variables, as specified in the corresponding figure legends. Survival curves were estimated using the Kaplan–Meier method and compared using the two‐sided log‐rank test. Categorical variables were compared using the χ^2^ test or Fisher's exact test, as appropriate. All statistical tests were two‐sided, and *p* < 0.05 was considered statistically significant.

## Author Contributions

S.B. and Y.T. performed the experiments, analyzed the data, and drafted the manuscript. Y.T. conducted the animal experiments and immunohistochemical analyses. W.N. contributed to study conception, experimental design, data interpretation, and manuscript revision. K.M., L.J., X.H., and J.S. performed the bioinformatic analyses. Z.T. and W.C. collected and curated the clinical specimens. W.Z. conceived and supervised the study, acquired funding, and revised the manuscript. All authors reviewed and approved the final manuscript.

## Ethics Statement

The study involving human HCC specimens and retrospective clinical data was approved by the Ethics Committee of the Affiliated Hospital of Nantong University (Approval No. 2025‐L186) and was conducted in accordance with the Declaration of Helsinki. The requirement for informed consent was waived by the Ethics Committee. All mouse and zebrafish experiments were approved by the Animal Care and Use Committee of Nantong University (Approval No. P20250305‐010) and were conducted in accordance with institutional guidelines.

## Conflicts of Interest

The authors declare no conflicts of interest.

## Supporting information




**Supporting File 1**: advs78039‐sup‐0001‐SuppMat.docx.

**Supporting File 2**: advs78039‐sup‐0002‐SuppMat.pdf.

**Supporting File 3**: advs78039‐sup‐0003‐SuppMat.pdf.

**Supporting File 4**: advs78039‐sup‐0004‐DataS1.xlsx.

## Data Availability

The data that support the findings of this study are available from the corresponding author upon reasonable request.

## References

[1] S. L. Chan , A. Lamarca , C. Hsu , V. Moreno , L. L. Chan , and B. P. Keenan , “New Targets and New Drugs for Hepatobiliary Cancers,” Journal of Hepatology 84, no. 1 (2025): 213–228, 10.1016/j.jhep.2025.08.018. 40865646

[2] H. Wang , Y. Lun , D. Xu , H. Jiang , Y. Yan , and X. Yang , “Research Progress and Therapeutic Strategies in Hepatocellular Carcinoma Metabolic Reprogramming,” Journal of Advanced Research 83 (2025): 439–453, 10.1016/j.jare.2025.08.023. PMC13131400 40850678

[3] K. A. McGlynn , C. Z. Watling , B. Y. Hernandez , and J. D. Groopman , “Environmental Risk Factors for Liver Cancer,” Hepatology (2025), 10.1097/HEP.0000000000001463. PMC13054843 40622863

[4] H. Yuan , J. Liu , R. Xu , et al., “The Spatiotemporal Heterogeneity of Reactive Oxygen Species In The Malignant Transformation of Viral Hepatitis To Hepatocellular Carcinoma: A New Insight,” Cellular & Molecular Biology Letters 30, no. 1 (2025): 70, 10.1186/s11658-025-00745-3. PMC12167593 40517270

[5] Y. Qiang , S. Bian , Y. Tong , et al., “USP1 Driven Mitotic Dysregulation and PLK1 Stabilization Confer Lenvatinib Resistance in Hepatocellular Carcinoma,” Journal of Experimental & Clinical Cancer Research 45, no. 1 (2026): 87, 10.1186/s13046-026-03683-w. PMC13045141 41820949

[6] D. Moris , A. Martinino , S. Schiltz , et al., “Advances in the Treatment of Hepatocellular Carcinoma: An Overview of the Current and Evolving Therapeutic Landscape for Clinicians,” CA: A Cancer Journal for Clinicians 75, no. 6 (2025): 498–527, 10.3322/caac.70018. PMC12593328 40392748

[7] S. Sidali , C. Campani , J. An , J. H. Shim , and J.‐C. Nault , “Tumor‐Based Biomarkers and Circulating Tumor DNA for Precision Medicine In Advanced Hepatocellular Carcinoma,” Clinical and Molecular Hepatology 32, no. 1 (2025): 69–90, 10.3350/cmh.2025.0746. PMC12835800 40831037

[8] A. Verreault , P. D. Kaufman , R. Kobayashi , and B. Stillman , “Nucleosome Assembly by a Complex of CAF‐1 and Acetylated Histones H3/H4,” Cell 87, no. 1 (1996): 95–104, 10.1016/s0092-8674(00)81326-4. 8858152

[9] K. Shibahara and B. Stillman , “Replication‐Dependent Marking of DNA by PCNA Facilitates CAF‐1‐Coupled Inheritance of Chromatin,” Cell 96, no. 4 (1999): 575–585, 10.1016/s0092-8674(00)80661-3. 10052459

[10] J. A. Mello , H. H. W. Silljé , D. M. J. Roche , D. B. Kirschner , E. A. Nigg , and G. Almouzni , “Human Asf1 and CAF‐1 Interact and Synergize in a Repair‐Coupled Nucleosome Assembly Pathway,” EMBO Reports 3, no. 4 (2002): 329–334, 10.1093/embo-reports/kvf068. PMC1084056 11897662

[11] N. V. Murzina , X. Y. Pei , W. Zhang , et al., “Structural Basis for the Recognition of Histone H4 by the Histone‐Chaperone RbAp46,” Structure 16, no. 7 (2008): 1077–1085, 10.1016/j.str.2008.05.006. PMC2572730 18571423

[12] Y. Zhang , H.‐H. Ng , H. Erdjument‐Bromage , P. Tempst , A. Bird , and D. Reinberg , “Analysis of the NuRD Subunits Reveals A Histone Deacetylase Core Complex And A Connection With DNA Methylation,” Genes & Development 13, no. 15 (1999): 1924–1935, 10.1101/gad.13.15.1924. PMC316920 10444591

[13] A. Volk , K. Liang , P. Suraneni , et al., “A CHAF1B‐Dependent Molecular Switch in Hematopoiesis and Leukemia Pathogenesis,” Cancer Cell 34, no. 5 (2018): 707–723.e7, 10.1016/j.ccell.2018.10.004. PMC6235627 30423293

[14] Q. Li , X. Zhang , and Z. Zhang , “CHAF1B Overexpression: A Brake for the Differentiation of Leukemia Cells,” Cancer Cell 34, no. 5 (2018): 693–694, 10.1016/j.ccell.2018.10.011. PMC6615053 30423290

[15] M. Mascolo , M. L. Vecchione , G. Ilardi , et al., “Overexpression of Chromatin Assembly Factor‐1/p60 Helps to Predict the Prognosis of Melanoma Patients,” BMC cancer 10, no. 1 (2010): 63, 10.1186/1471-2407-10-63. PMC2843674 20178651

[16] M. de Tayrac , S. Saikali , M. Aubry , et al., “Prognostic Significance of EDN/RB, HJURP, p60/CAF‐1 and PDLI4, Four New Markers in High‐Grade Gliomas,” PLoS ONE 8, no. 9 (2013): 73332, 10.1371/journal.pone.0073332. PMC3770632 24039914

[17] H.‐Y. Han , J.‐T. Mou , W.‐P. Jiang , X.‐M. Zhai , and K. Deng , “Five Candidate Biomarkers Associated With The Diagnosis and Prognosis Of Cervical Cancer,” Bioscience Reports 41, no. 3 (2021): BSR20204394, 10.1042/BSR20204394. PMC7955105 33616161

[18] Z. Zheng , Y. Lin , H. Guo , et al., “CHAF1B Promotes the Progression of Lung Squamous‐Cell Carcinoma by Inhibiting SETD7 Expression,” Frontiers of Medicine 19, no. 2 (2025): 318–328, 10.1007/s11684-024-1122-2. 39862337

[19] J. Straka , J.‐B. Khatib , L. Pale , C.‐M. Nicolae , and G.‐L. Moldovan , “CAF‐1 Promotes Efficient PrimPol Recruitment to Nascent DNA for Single‐Stranded DNA Gap Formation,” Nucleic Acids Research 52, no. 22 (2024): 13865–13880, 10.1093/nar/gkae1068. PMC11662685 39558157

[20] S. E. Polo , D. Roche , and G. Almouzni , “New Histone Incorporation Marks Sites of UV Repair in Human Cells,” Cell 127, no. 3 (2006): 481–493, 10.1016/j.cell.2006.08.049. 17081972

[21] C. M. Green and G. Almouzni , “Local Action of the Chromatin Assembly Factor CAF‐1 at Sites Of Nucleotide Excision Repair In Vivo,” EMBO Journal 22, no. 19 (2003): 5163–5174, 10.1093/emboj/cdg478. PMC204462 14517254

[22] N. Xu , Y. Tong , K. Ma , L. Jiang , and W. Zheng , “Dynamic Ubiquitination Networks In Liver Cancer: Decoding E3 Ligases and Deubiquitinases as Gatekeepers of Therapeutic Resistance,” Medical Oncology 42, no. 8 (2025): 352, 10.1007/s12032-025-02912-0. 40684404

[23] H. Zhang , H. Liu , Y. Chen , et al., “A Cell Cycle‐Dependent BRCA1–UHRF1 Cascade Regulates DNA Double‐Strand Break Repair Pathway Choice,” Nature Communications 7, no. 1 (2016): 10201, 10.1038/ncomms10201. PMC4728409 26727879

[24] S. Park , J. H. Cho , J.‐H. Kim , et al., “Hypoxia Stabilizes SETDB1 to Maintain Genome Stability,” Nucleic Acids Research 51, no. 20 (2023): 11178–11196, 10.1093/nar/gkad796. PMC10639076 37850636

[25] M. Bostick , J. K. Kim , P.‐O. Este`ve , A. Clark , S. Pradhan , and S. E. Jacobsen , “UHRF1 Plays a Role in Maintaining DNA Methylation in Mammalian Cells,” Science 317, no. 5845 (2007): 1760–1764, 10.1126/science.1147939. 17673620

[26] M. L. Matsumoto , K. E. Wickliffe , K. C. Dong , et al., “K11‐Linked Polyubiquitination In Cell Cycle Control Revealed by a K11 Linkage‐Specific Antibody,” Molecular Cell 39, no. 3 (2010): 477–484, 10.1016/j.molcel.2010.07.001. 20655260

[27] G. Wang , J. Long , Y. Gao , et al., “SETDB1‐Mediated Methylation of Akt Promotes its K63‐Linked Ubiquitination and Activation Leading to Tumorigenesis,” Nature Cell Biology 21, no. 2 (2019): 214–225, 10.1038/s41556-018-0266-1. PMC6414065 30692626

[28] A. Volk and J. D. Crispino , “The Role of The Chromatin Assembly Complex (CAF‐1) and its p60 Subunit (CHAF1b) in Homeostasis and Disease,” Biochimica et Biophysica Acta (BBA) ‐ Gene Regulatory Mechanisms 1849, no. 8 (2015): 979–986, 10.1016/j.bbagrm.2015.05.009. PMC4515380 26066981

[29] L. Cheng , X. Zhang , Y. Wang , et al., “Chromatin Assembly Factor 1 (CAF‐1) Facilitates The Establishment of Facultative Heterochromatin During Pluripotency Exit,” Nucleic Acids Research 47, no. 21 (2019): 11114–11131, 10.1093/nar/gkz858. PMC6868363 31586391

[30] J. Guo , X. Dai , B. Laurent , et al., “AKT Methylation by SETDB1 Promotes AKT Kinase Activity and Oncogenic Functions,” Nature Cell Biology 21, no. 2 (2019): 226–237, 10.1038/s41556-018-0261-6. PMC6377565 30692625

[31] Y. Saito , D. Yin , N. Kubota , et al., “A Therapeutically Targetable TAZ‐TEAD2 Pathway Drives the Growth of Hepatocellular Carcinoma via ANLN and KIF23,” Gastroenterology 164, no. 7 (2023): 1279–1292, 10.1053/j.gastro.2023.02.043. PMC10335360 36894036

[32] Z. Yang , C. Yan , J. Ma , et al., “Lactylome Analysis Suggests Lactylation‐Dependent Mechanisms of Metabolic Adaptation In Hepatocellular Carcinoma,” Nature Metabolism 5, no. 1 (2023): 61–79, 10.1038/s42255-022-00710-w. 36593272

[33] Y. Liu , Z. Xun , K. Ma , et al., “Identification of a Tumour Immune Barrier in the HCC Microenvironment That Determines the Efficacy of Immunotherapy,” Journal of Hepatology 78, no. 4 (2023): 770–782, 10.1016/j.jhep.2023.01.011. 36708811

[34] R. Wu , W. Guo , X. Qiu , et al., “Comprehensive Analysis of Spatial Architecture in Primary Liver Cancer,” Science Advances 7, no. 51 (2021): abg3750, 10.1126/sciadv.abg3750. PMC8683021 34919432

[35] S. Bian , W. Ni , L. Zhou , et al., “Ubiquitin‐Specific Protease 1 Facilitates Hepatocellular Carcinoma Progression By Modulating Mitochondrial Fission And Metabolic Reprogramming Via Cyclin‐Dependent Kinase 5 Stabilization,” Cell Death & Differentiation 31, no. 9 (2024): 1202–1218, 10.1038/s41418-024-01342-1. PMC11369097 39009653

[36] C. Dai , Y. Tong , N. Bai , et al., “Decoding the Role of Nucleic Acid Binding Protein 2 in Lipid Dysregulation and Hepatocellular Carcinoma Progression Through LKB1‐Mediated Mitochondrial Dysfunction,” Cellular Signalling 132 (2025): 111820, 10.1016/j.cellsig.2025.111820. 40250697

