# Supplementary material for: USP4‐Dependent CHAF1B Stabilization Regulates Distinct SETDB1 Ubiquitin States Linked to AKT T308 Signaling and Lipogenic Remodeling in HCC
Source: Adv Sci (Weinh). 2026 Sep 29:e78039. Online ahead of print. doi: 10.1002/advs.78039 (PMC13624420; doi:10.1002/advs.78039)

Fig1B

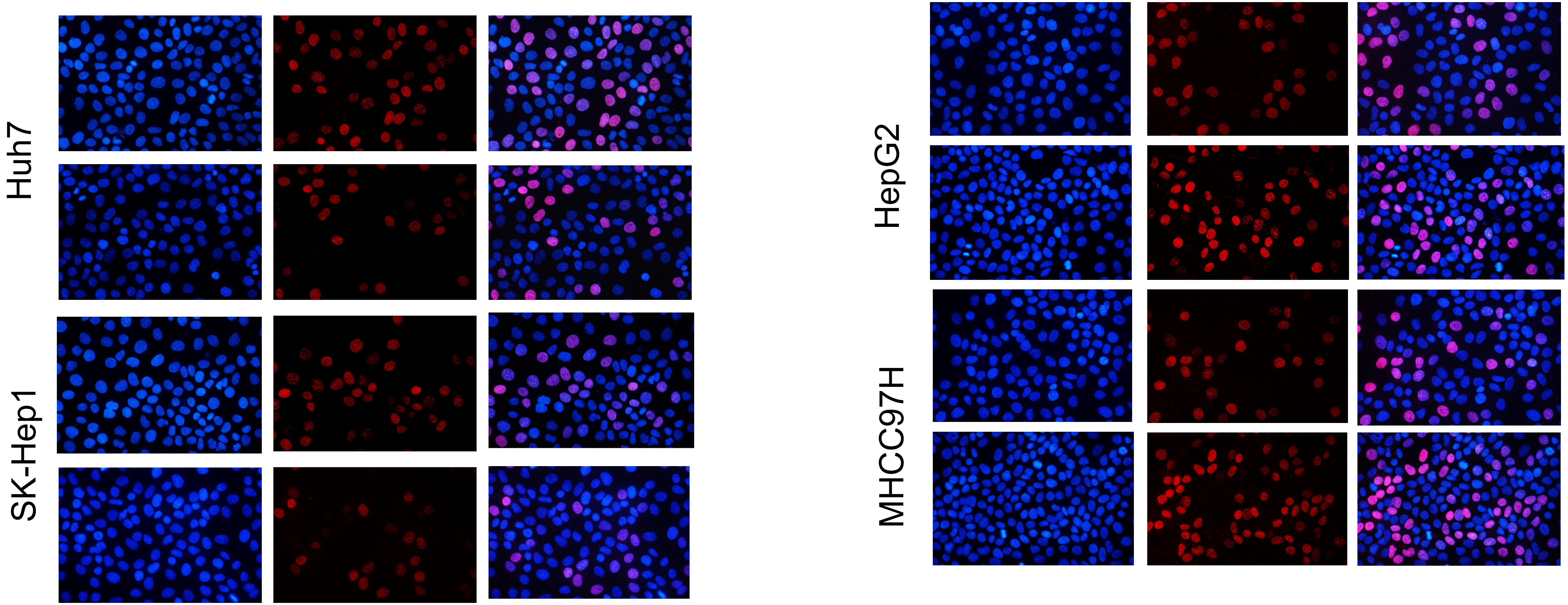

Fig1D

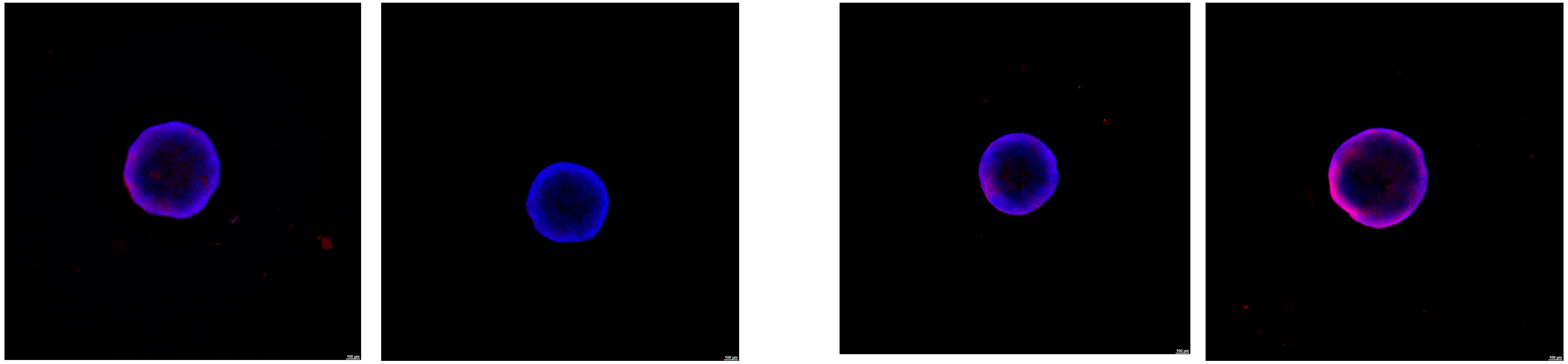

Huh7 HepG2

Fig2F

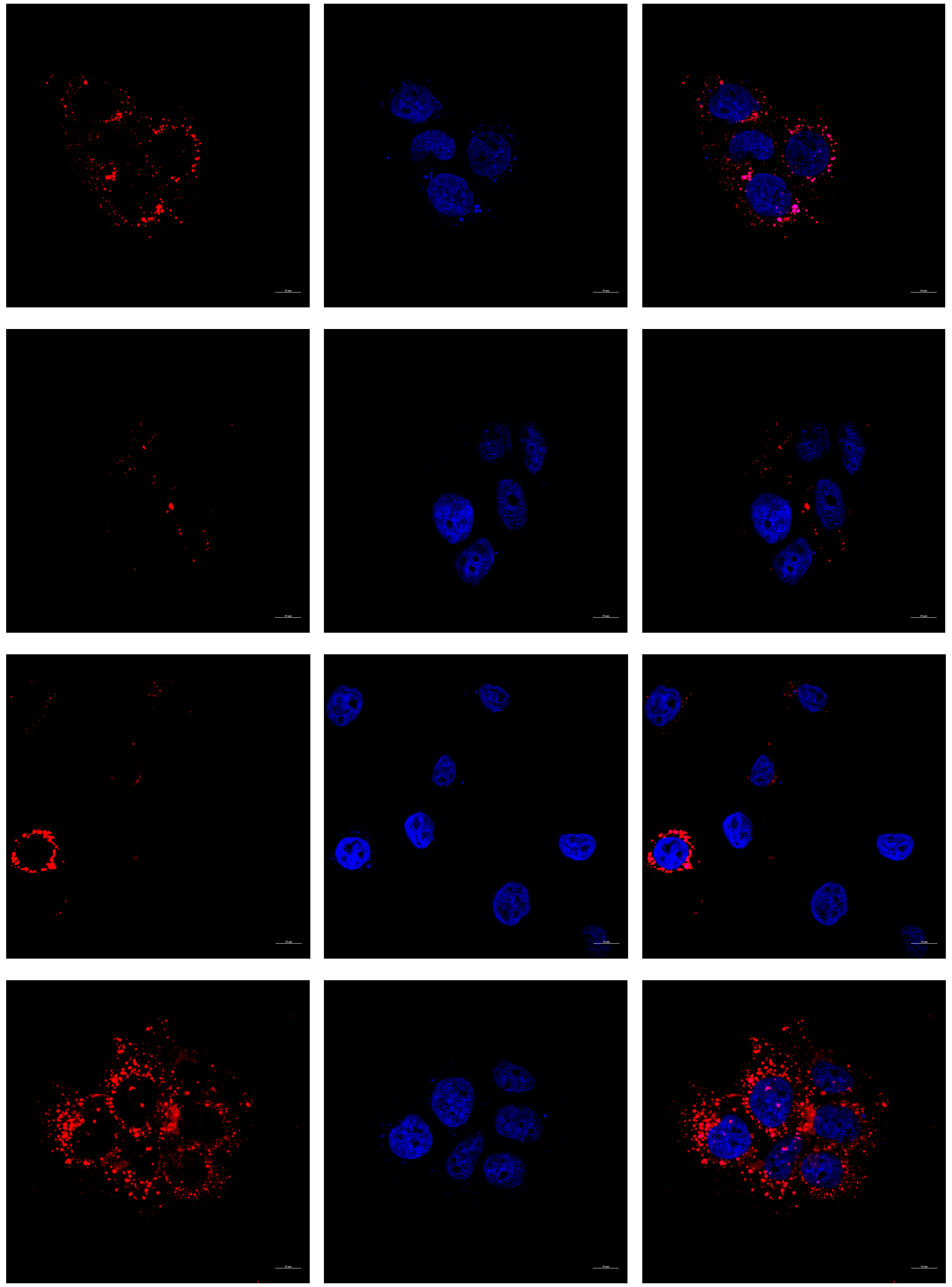

Fig2G

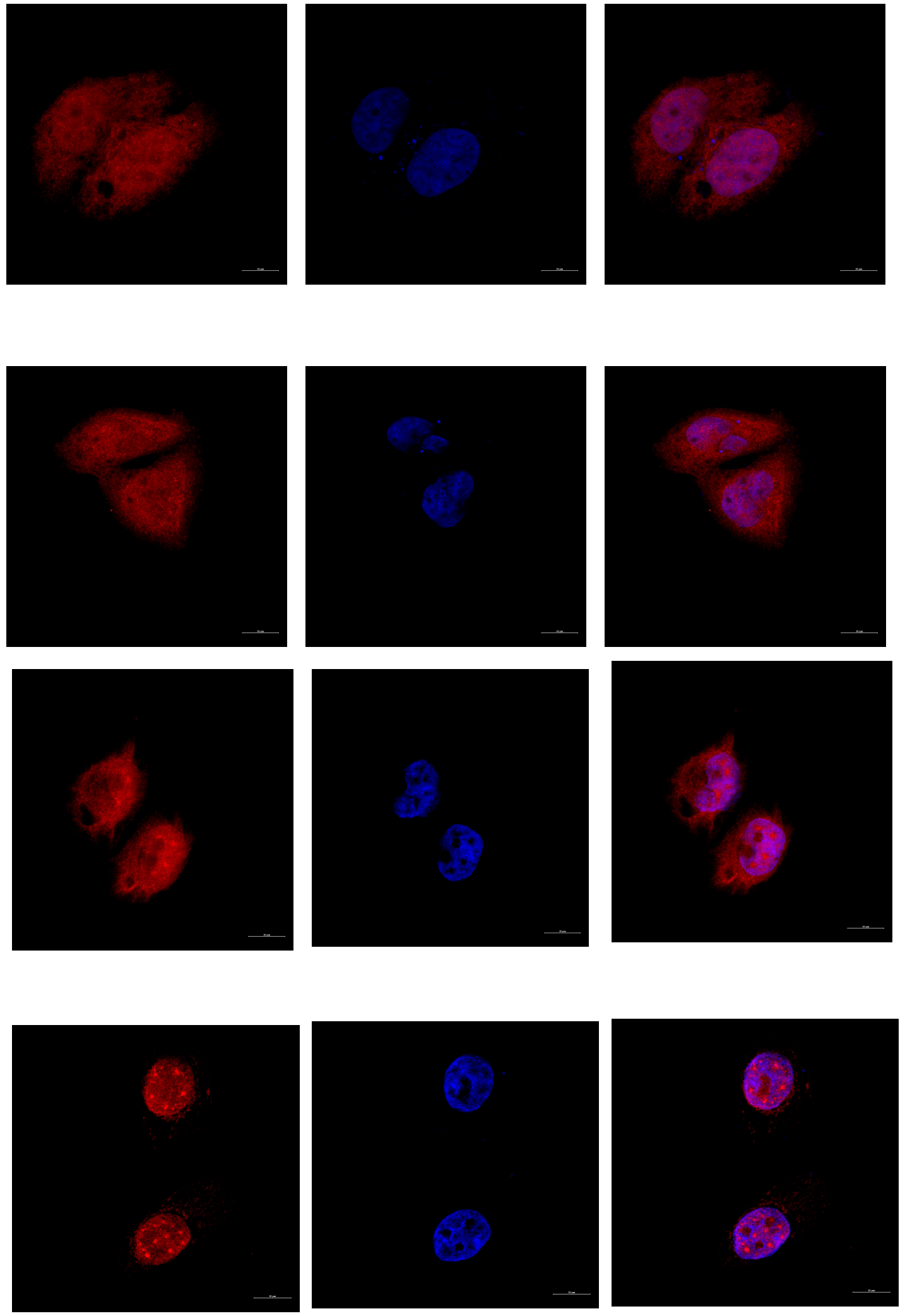

Fig2M

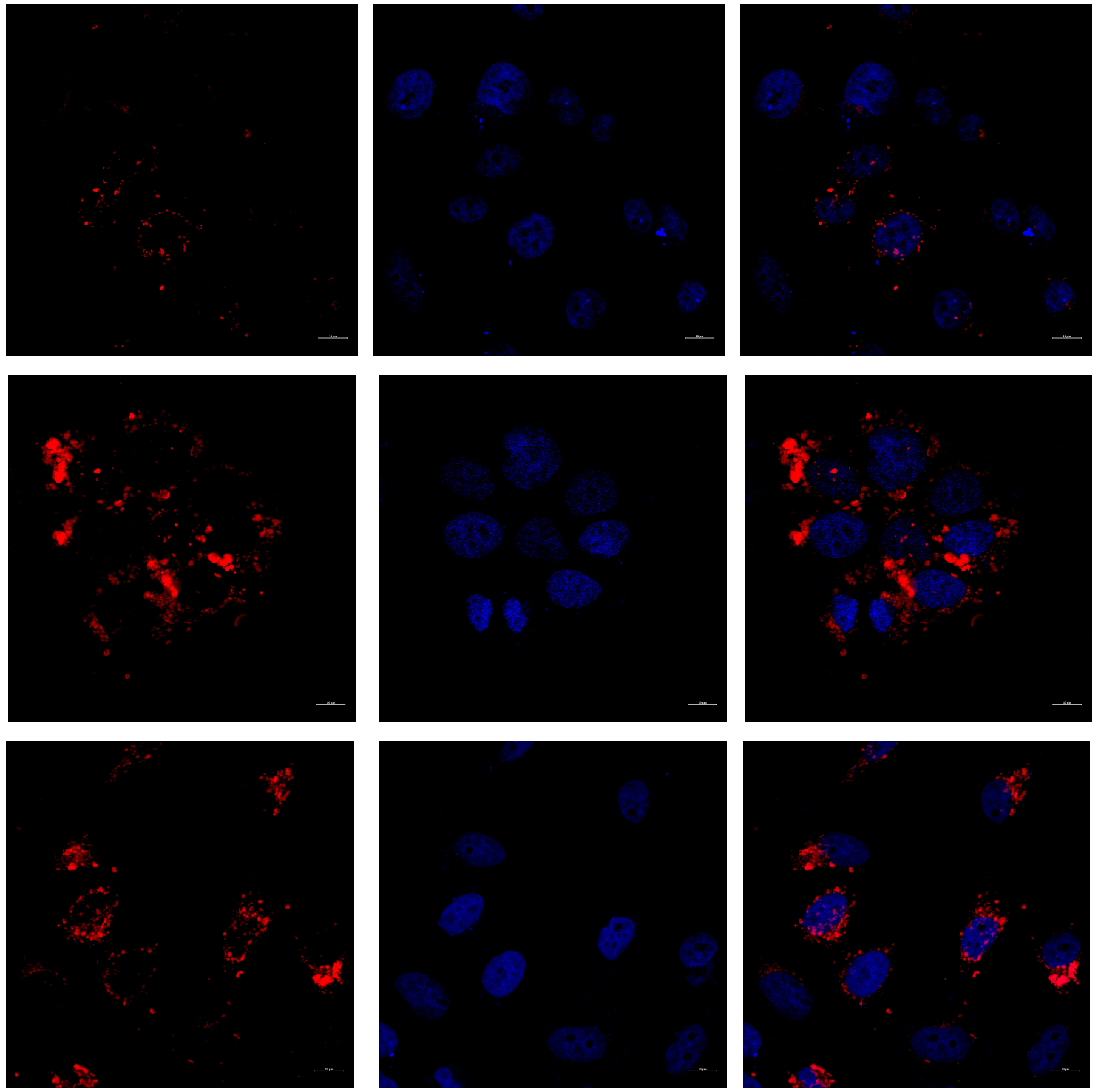

Fig2N

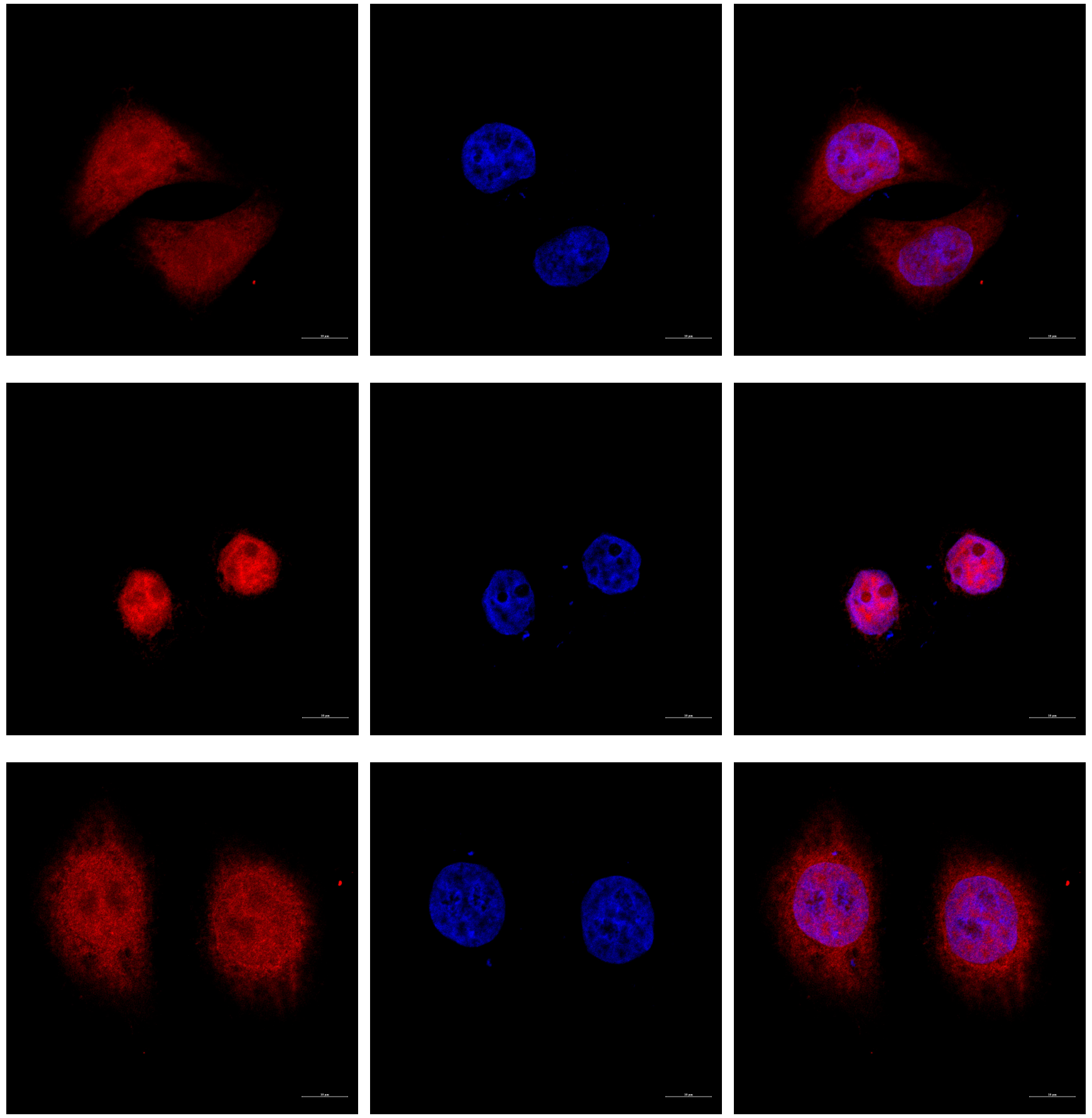

Fig2P

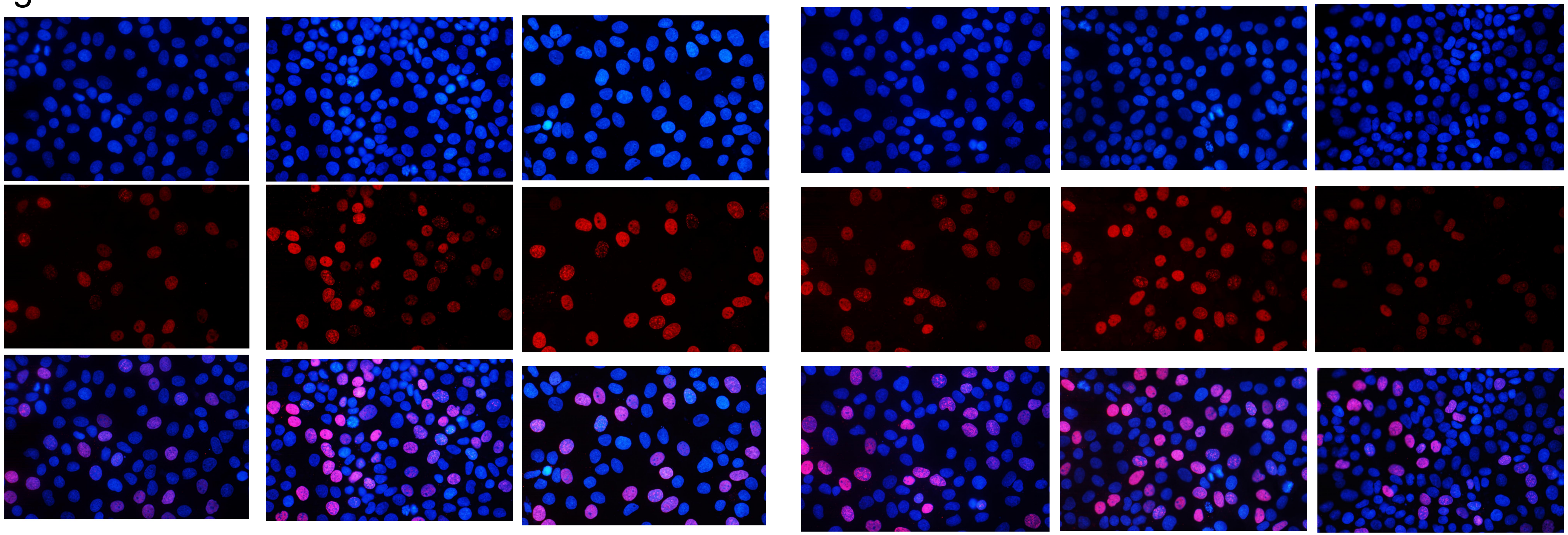

Fig2U

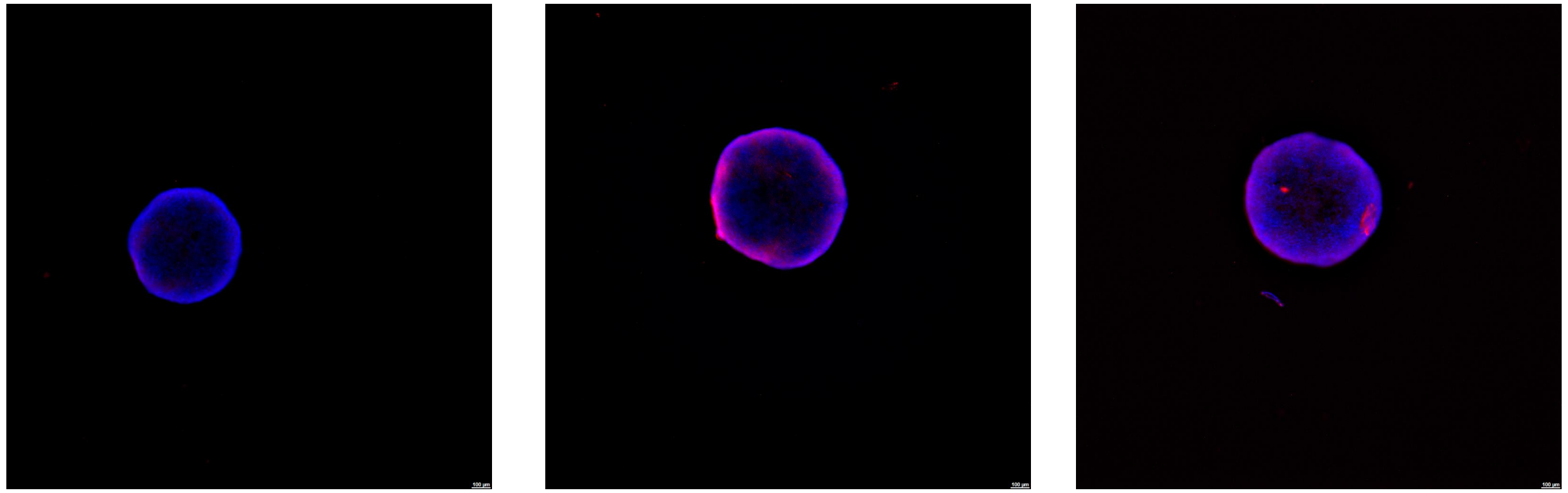

Fig3C

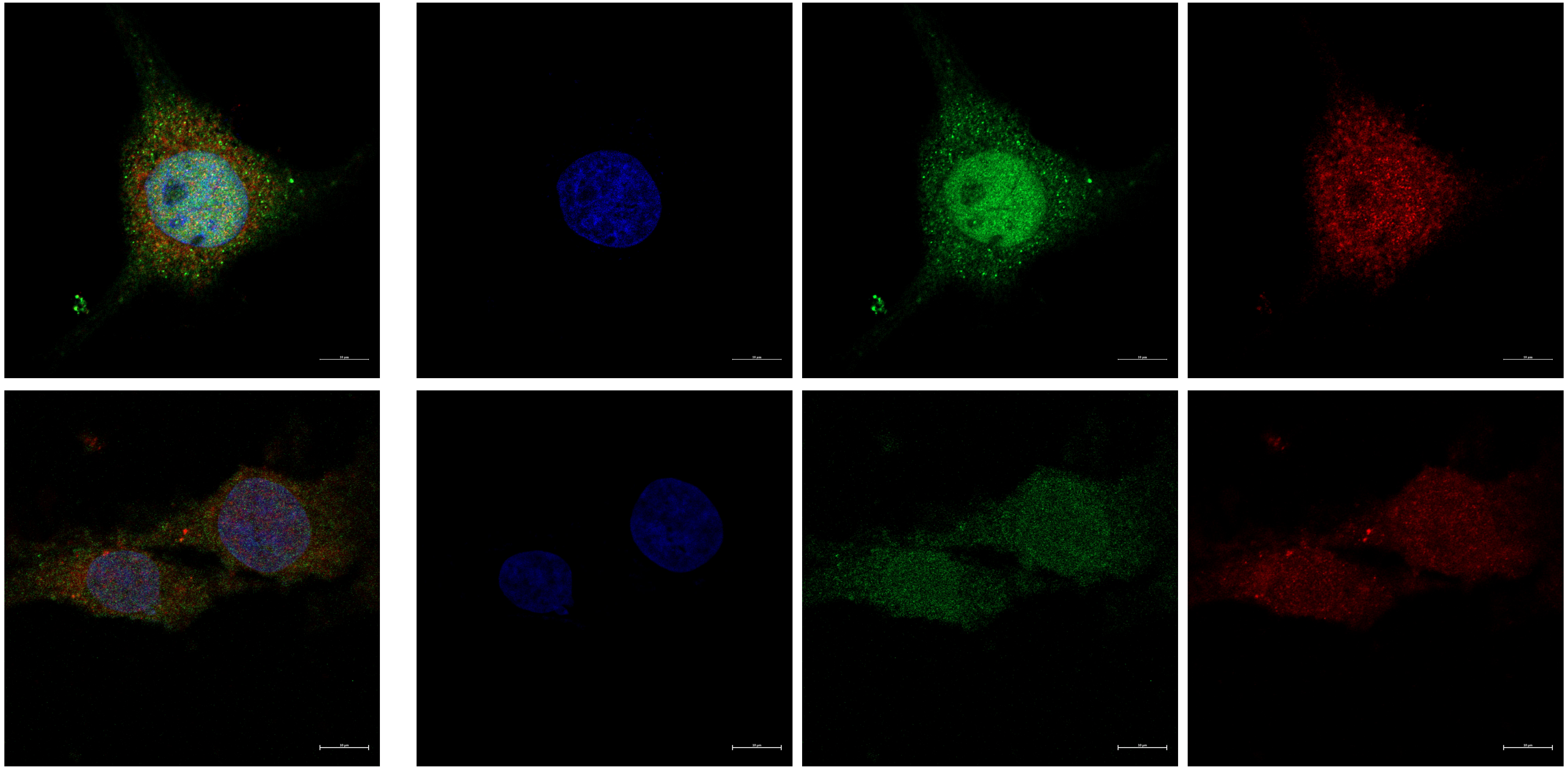

Fig3D

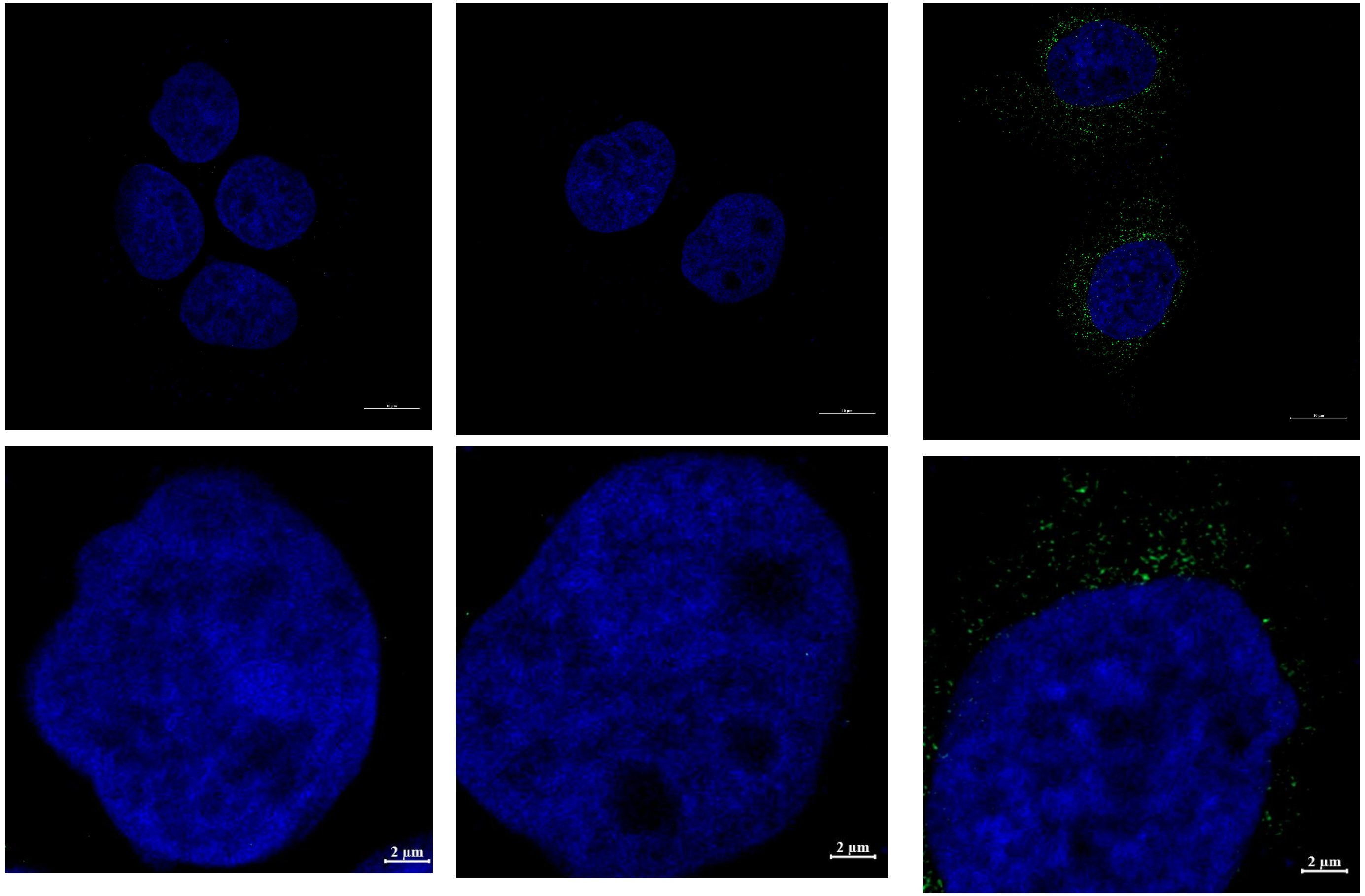

Fig3K

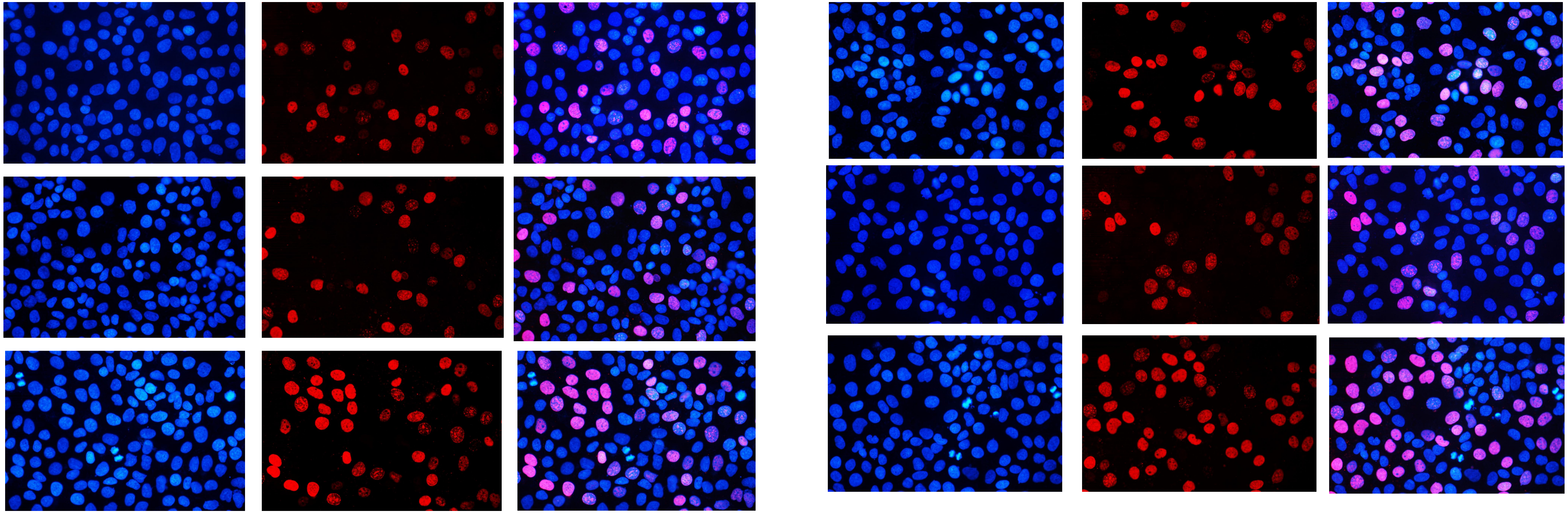

Fig3P

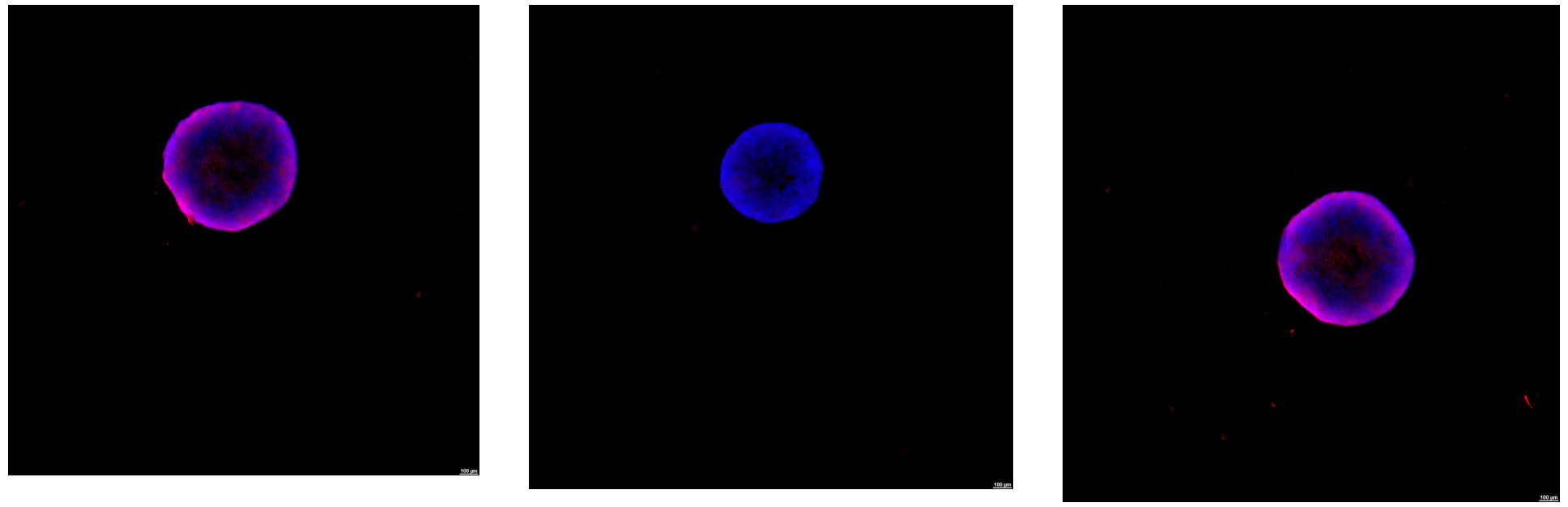

Fig4I

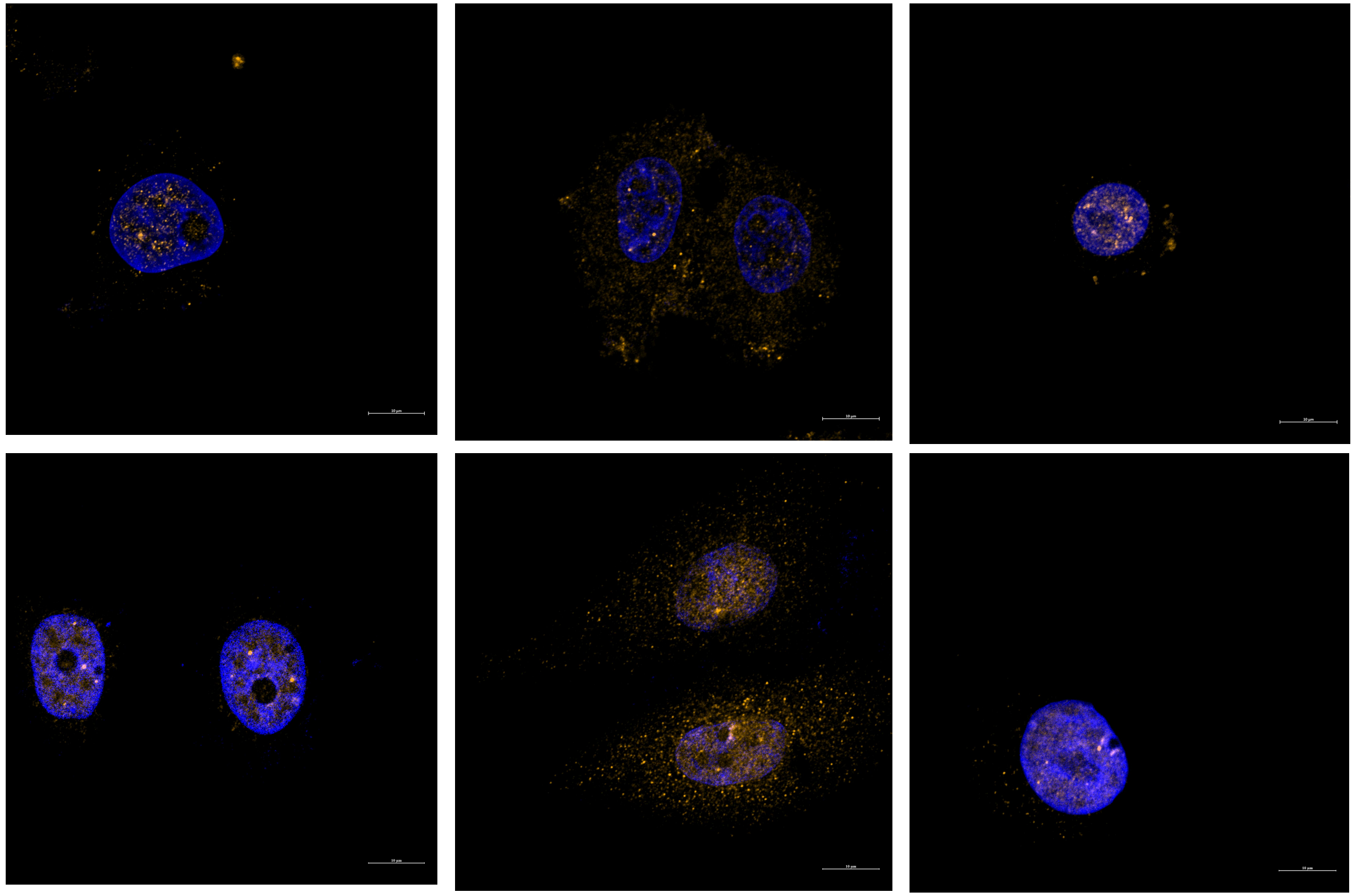

Fig4O

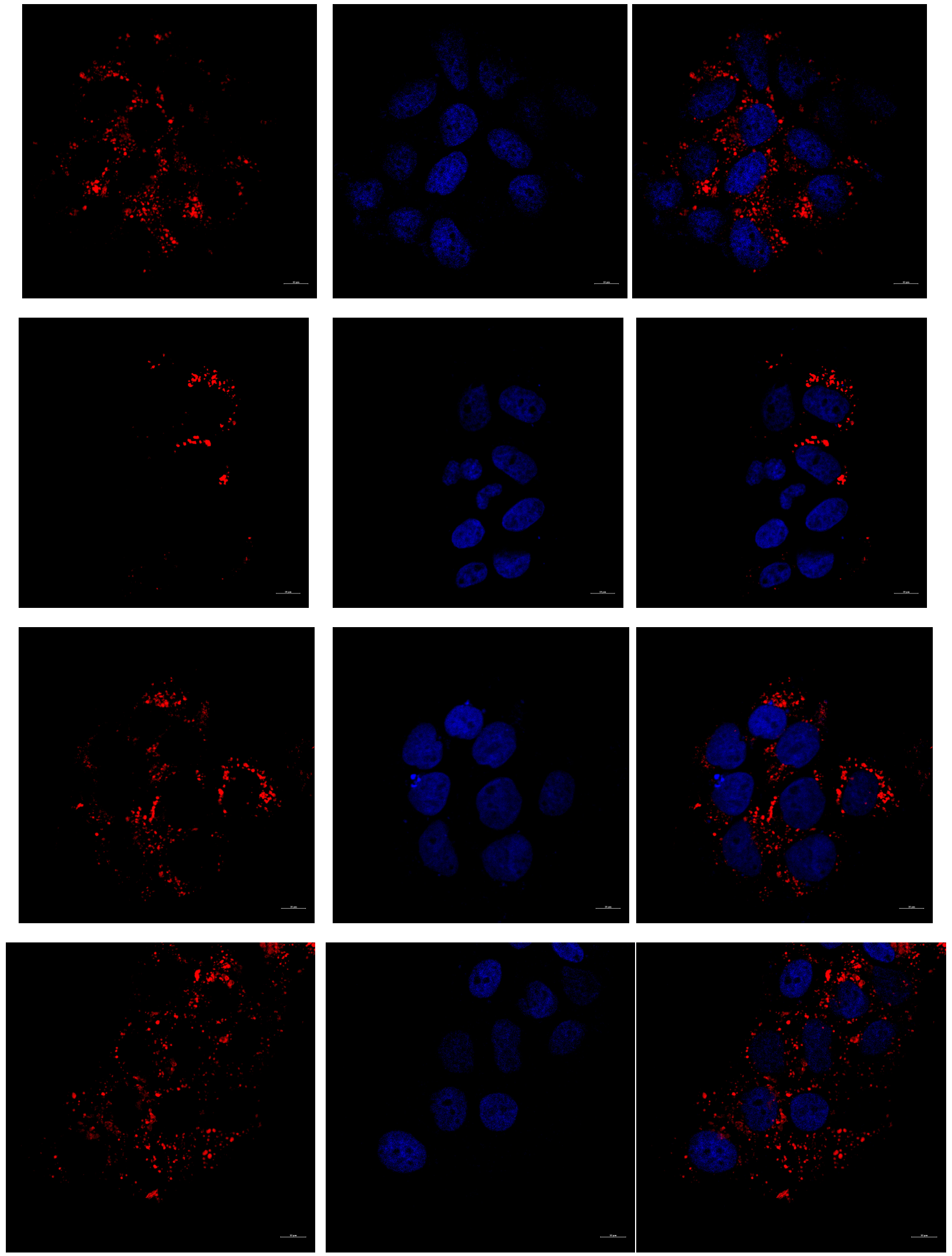

Fig4P

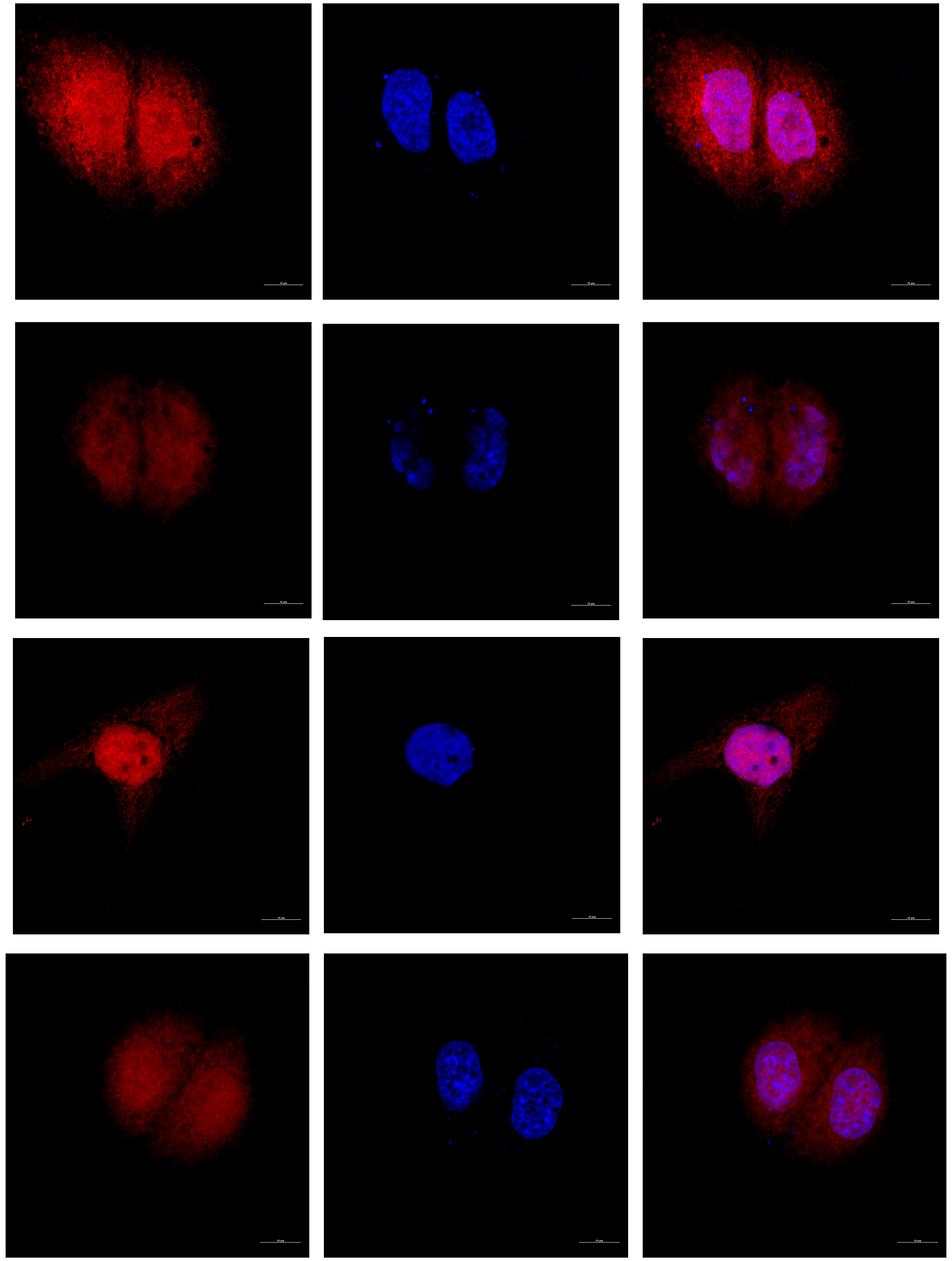

Fig5E

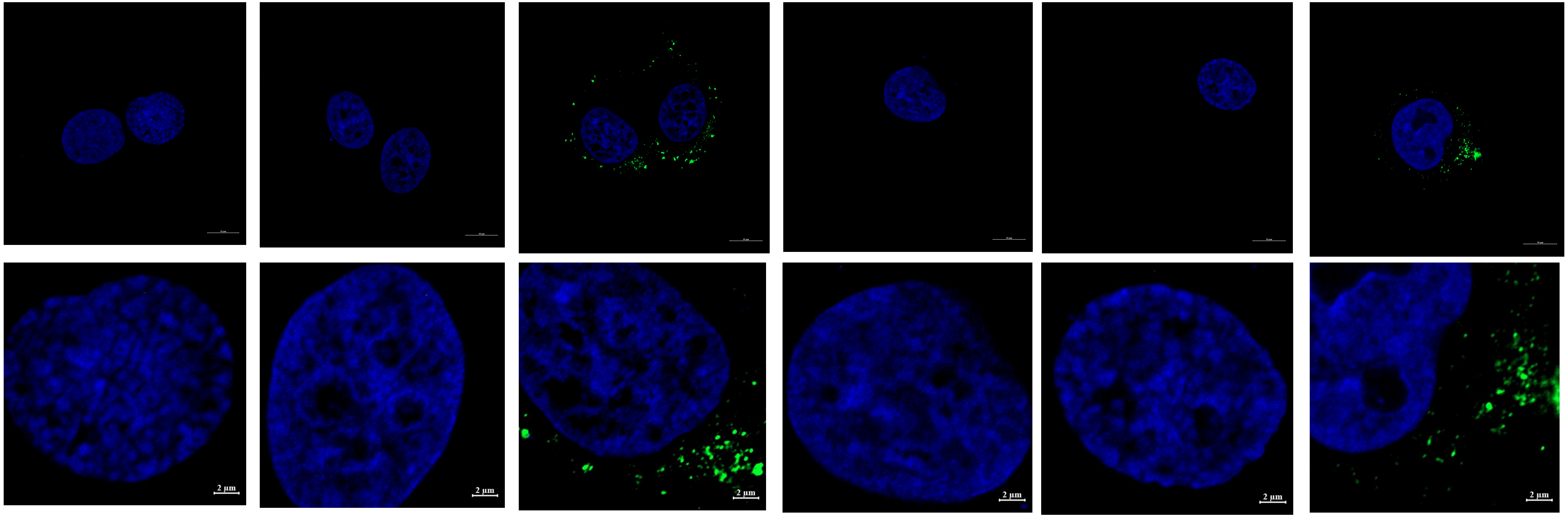

Fig6A

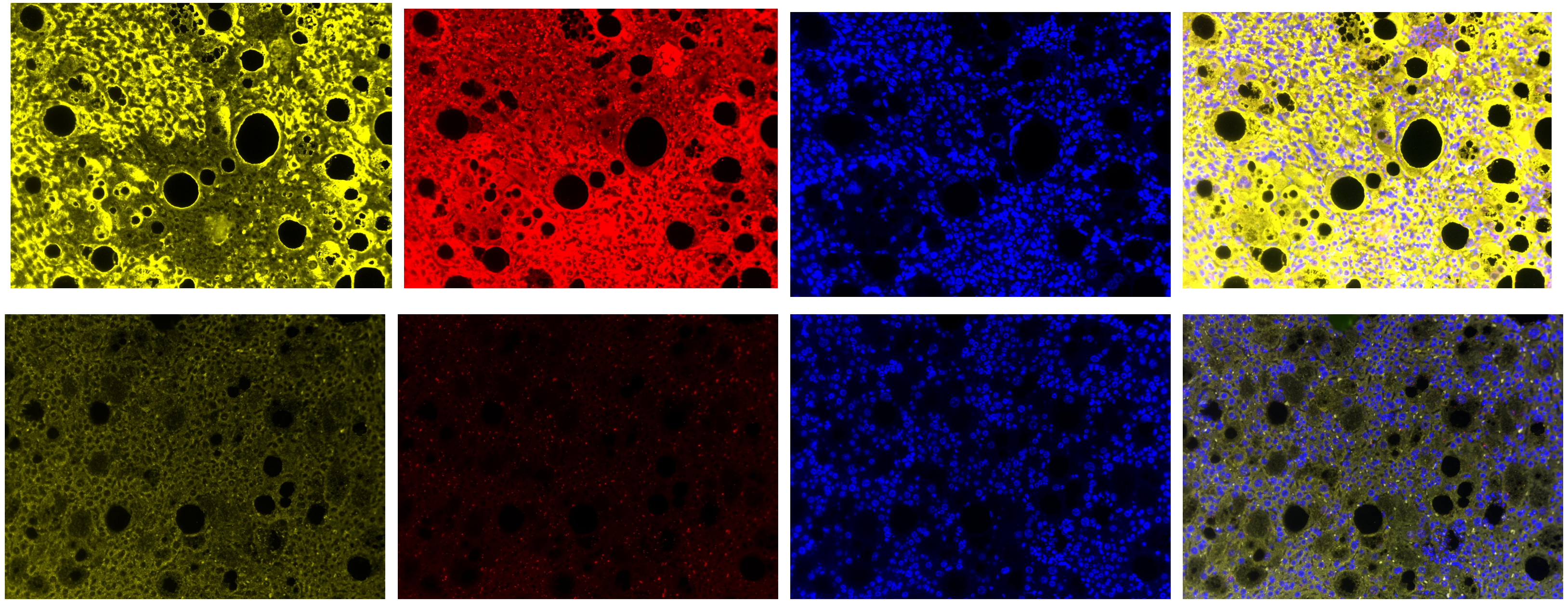

Fig6B

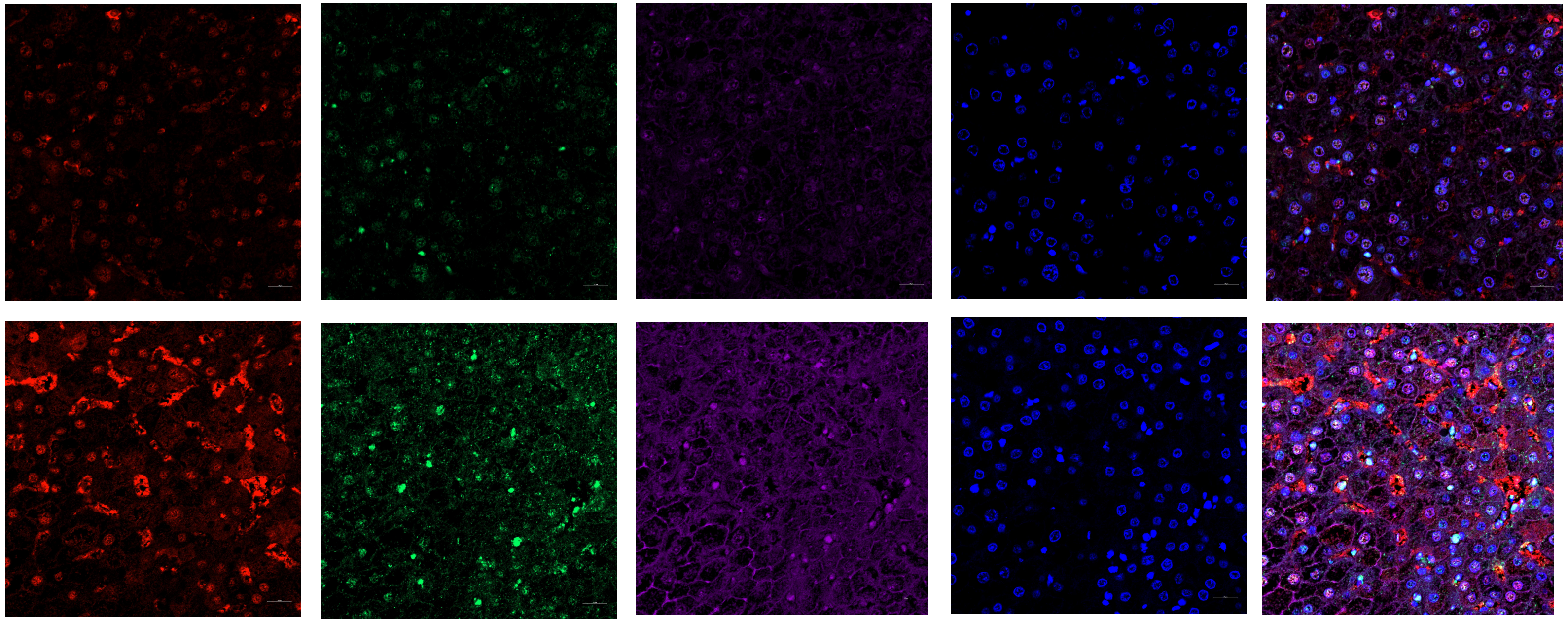

Fig7B

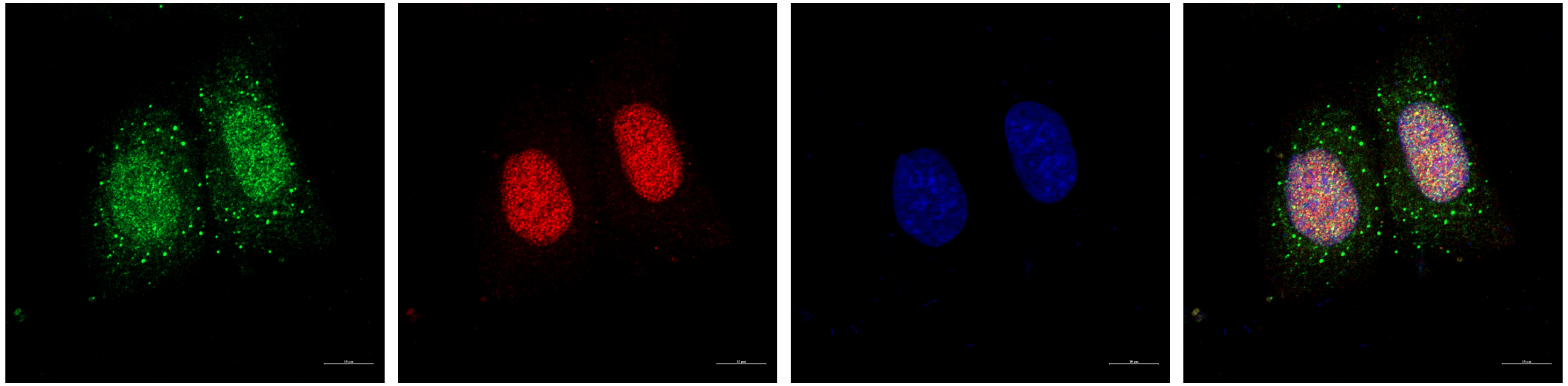

Fig7K

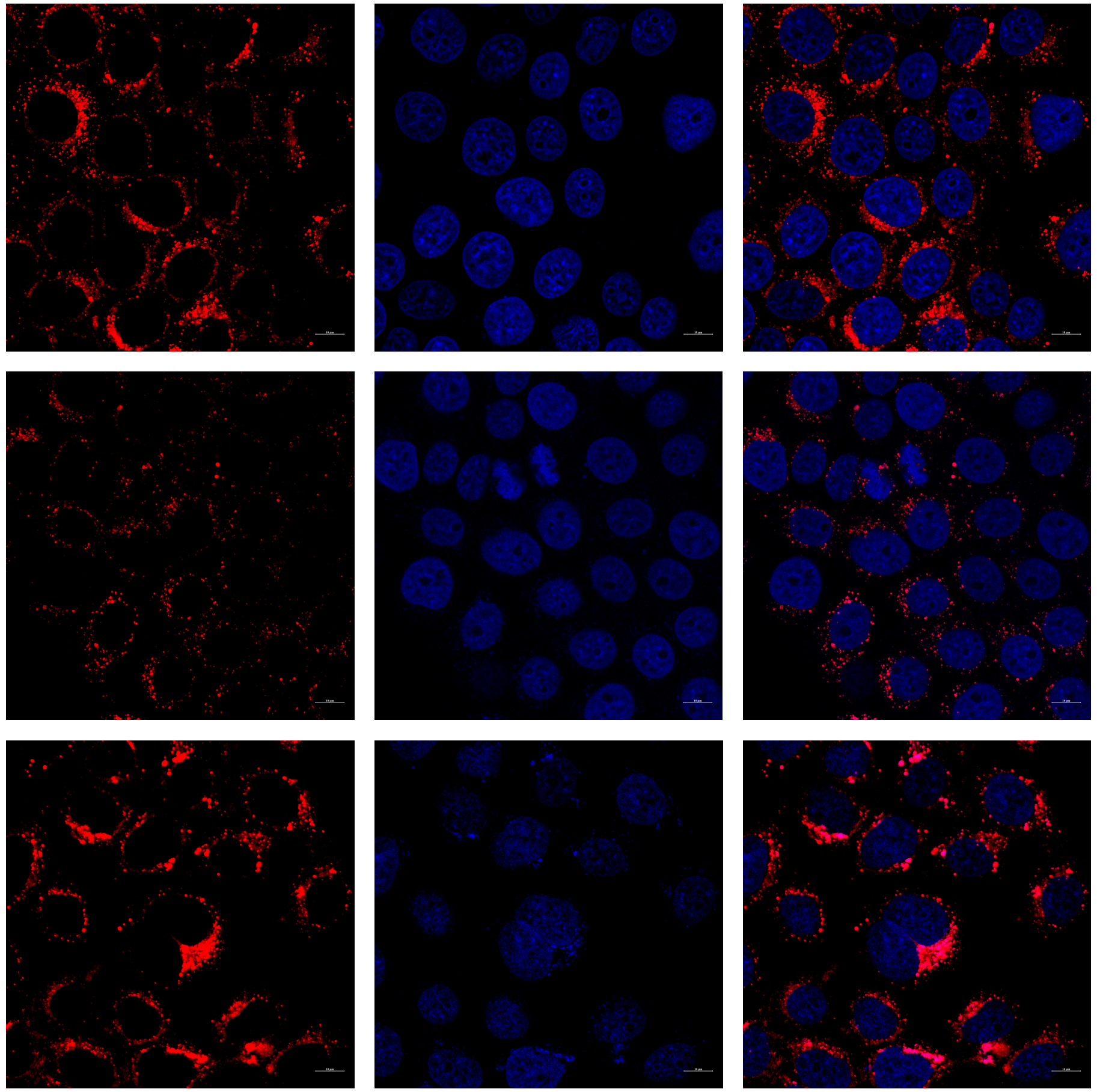

Fig7I

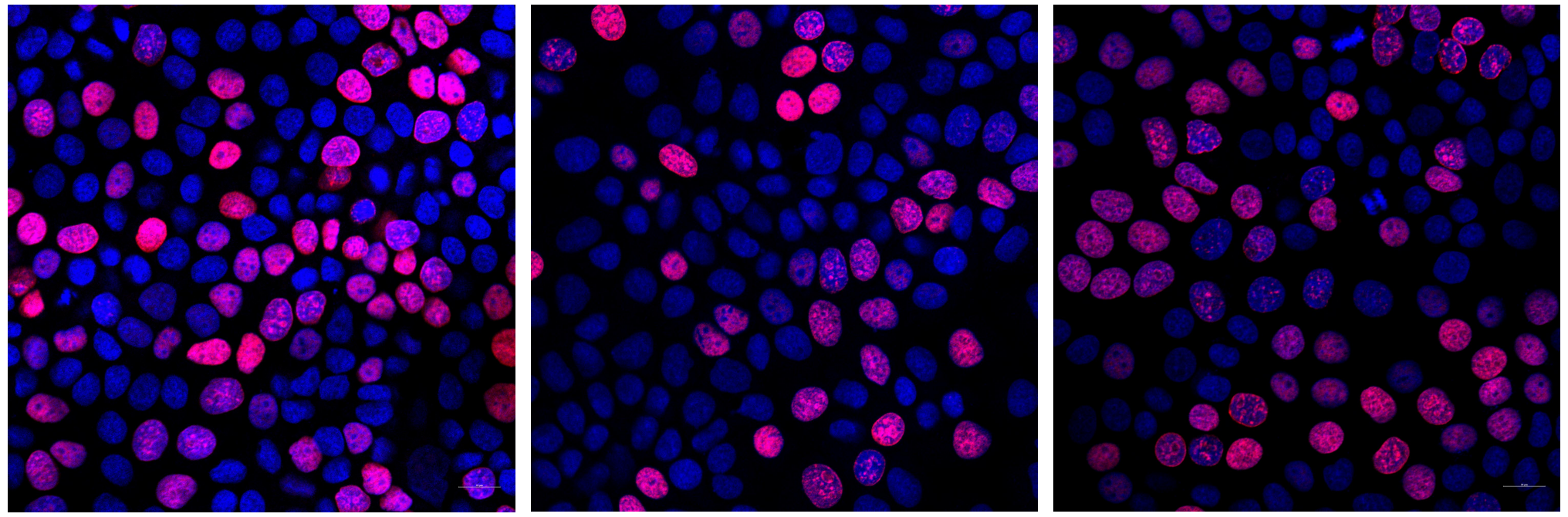

Fig8I

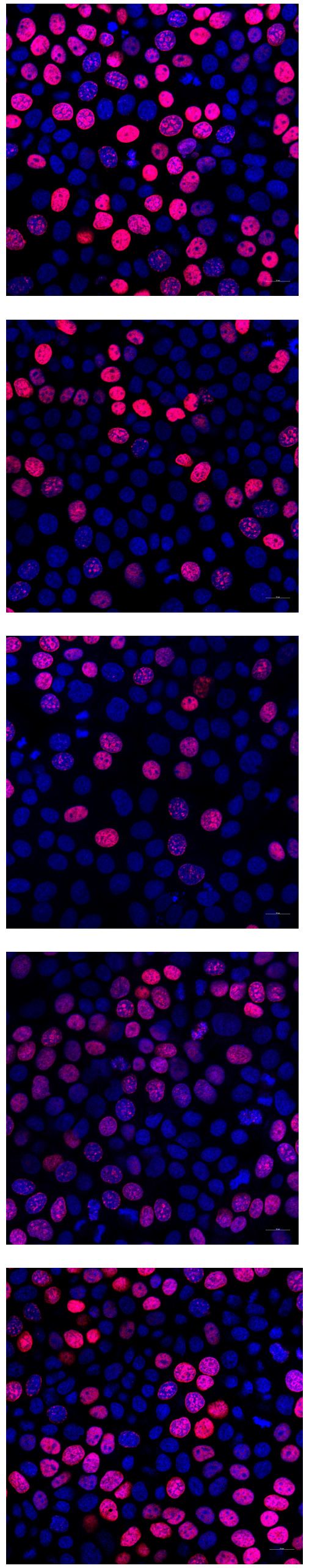

Fig8J

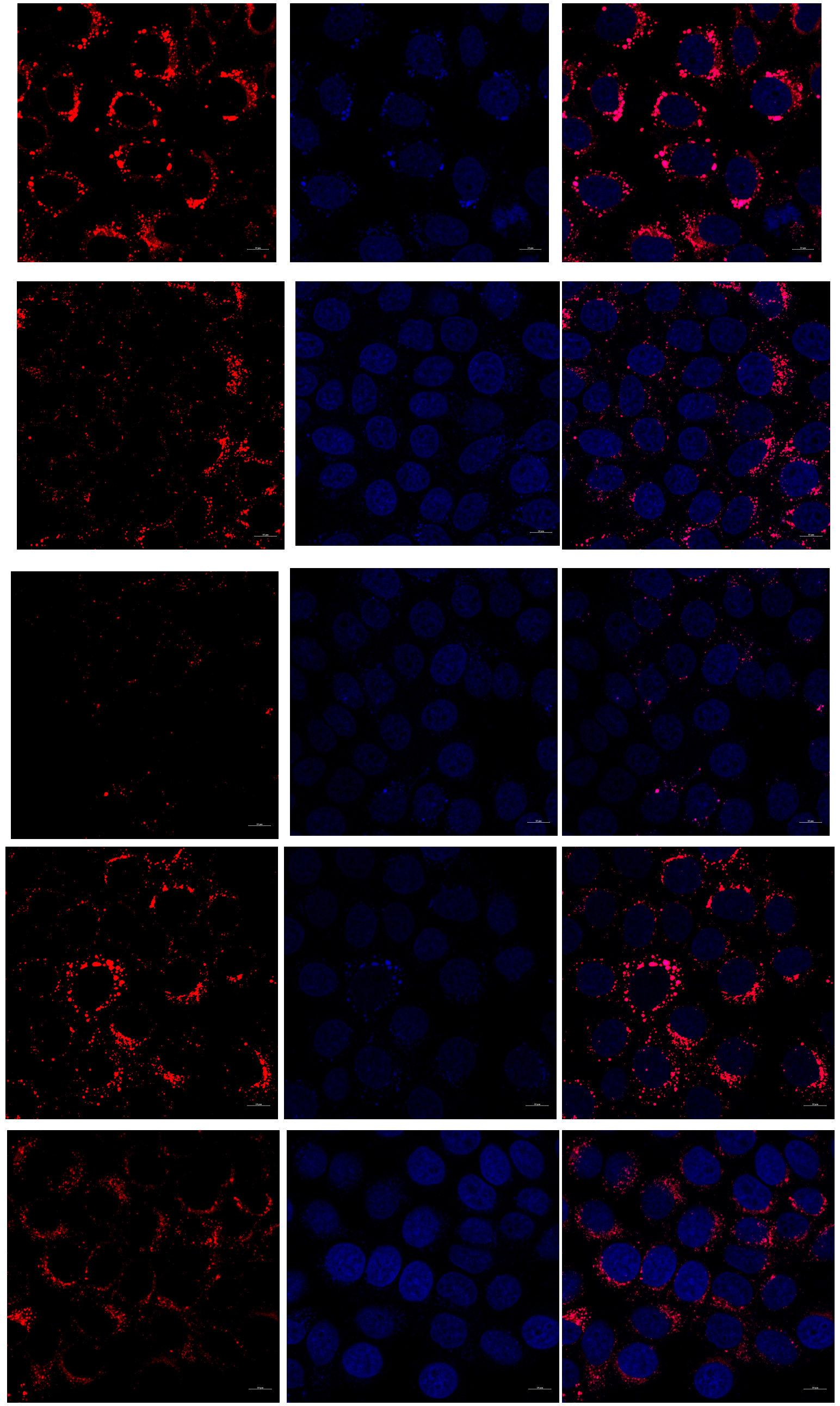

FigS2C

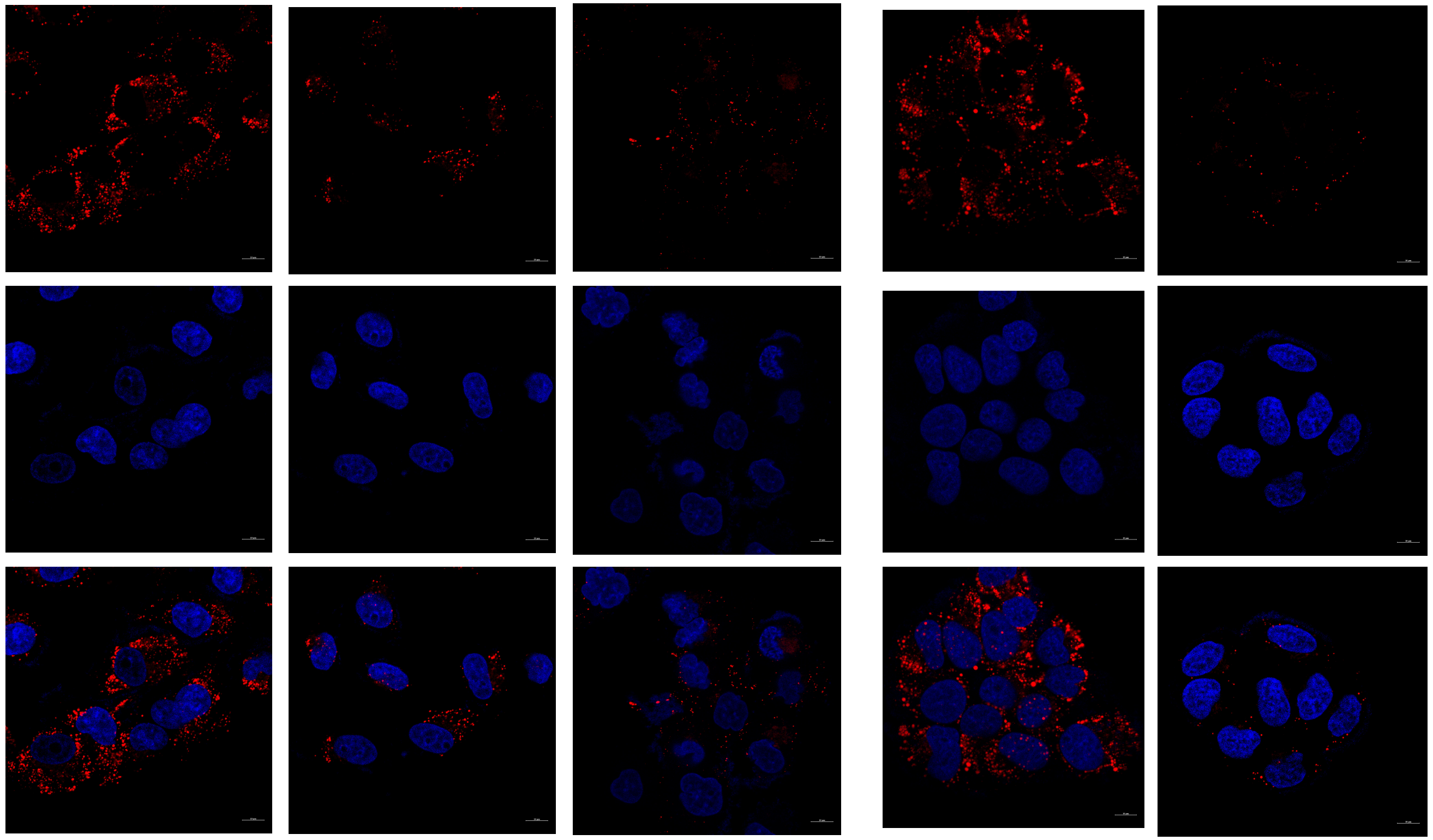

FigS2D

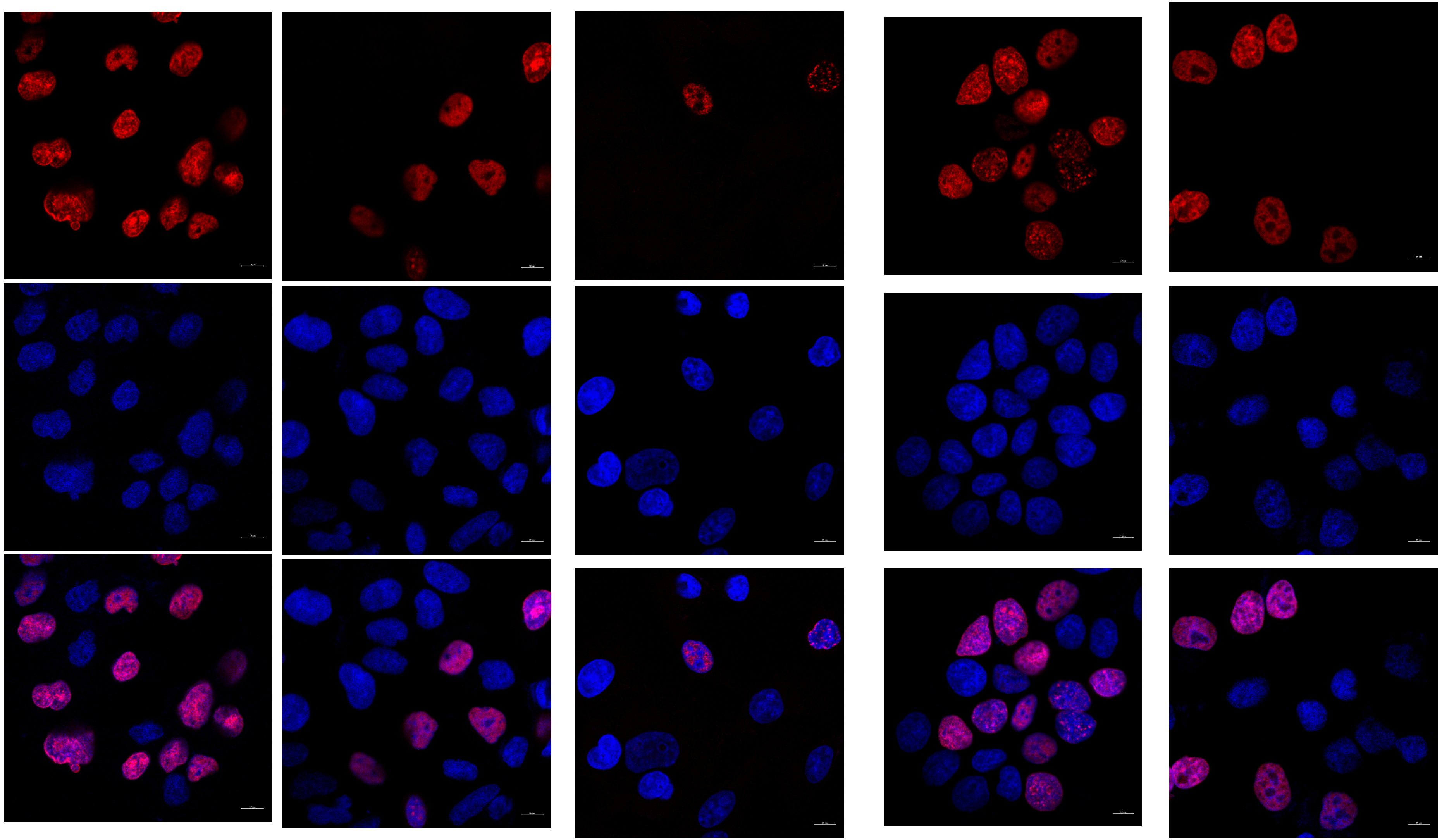

FigS4D

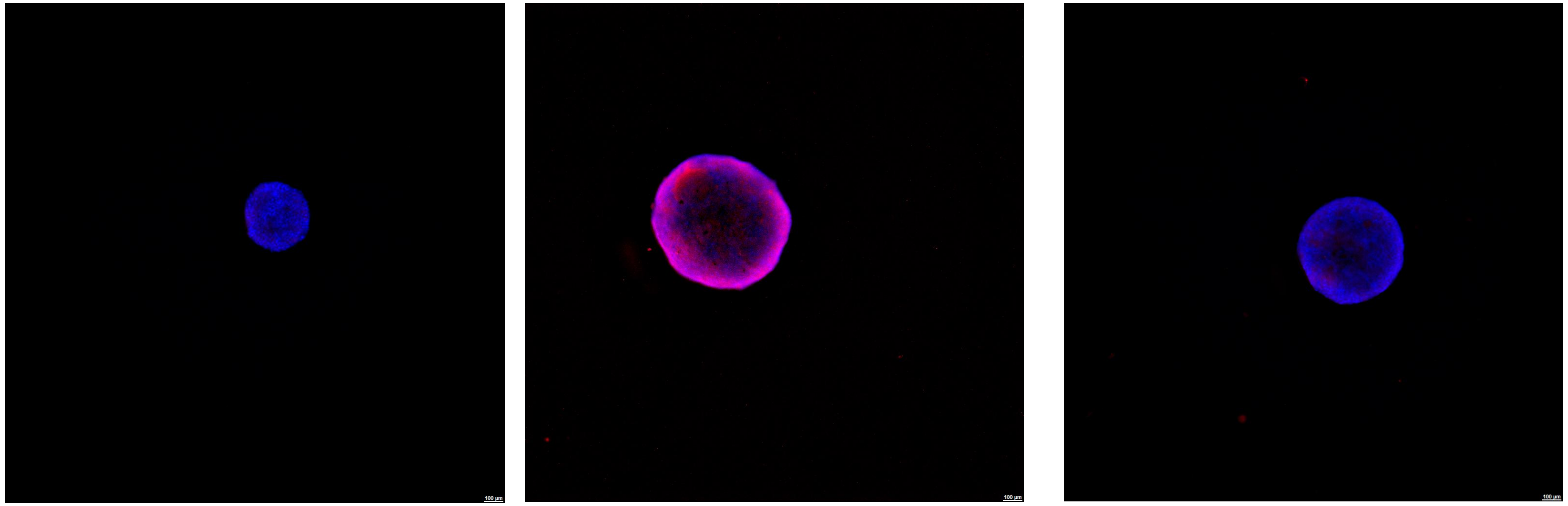

FigS5C

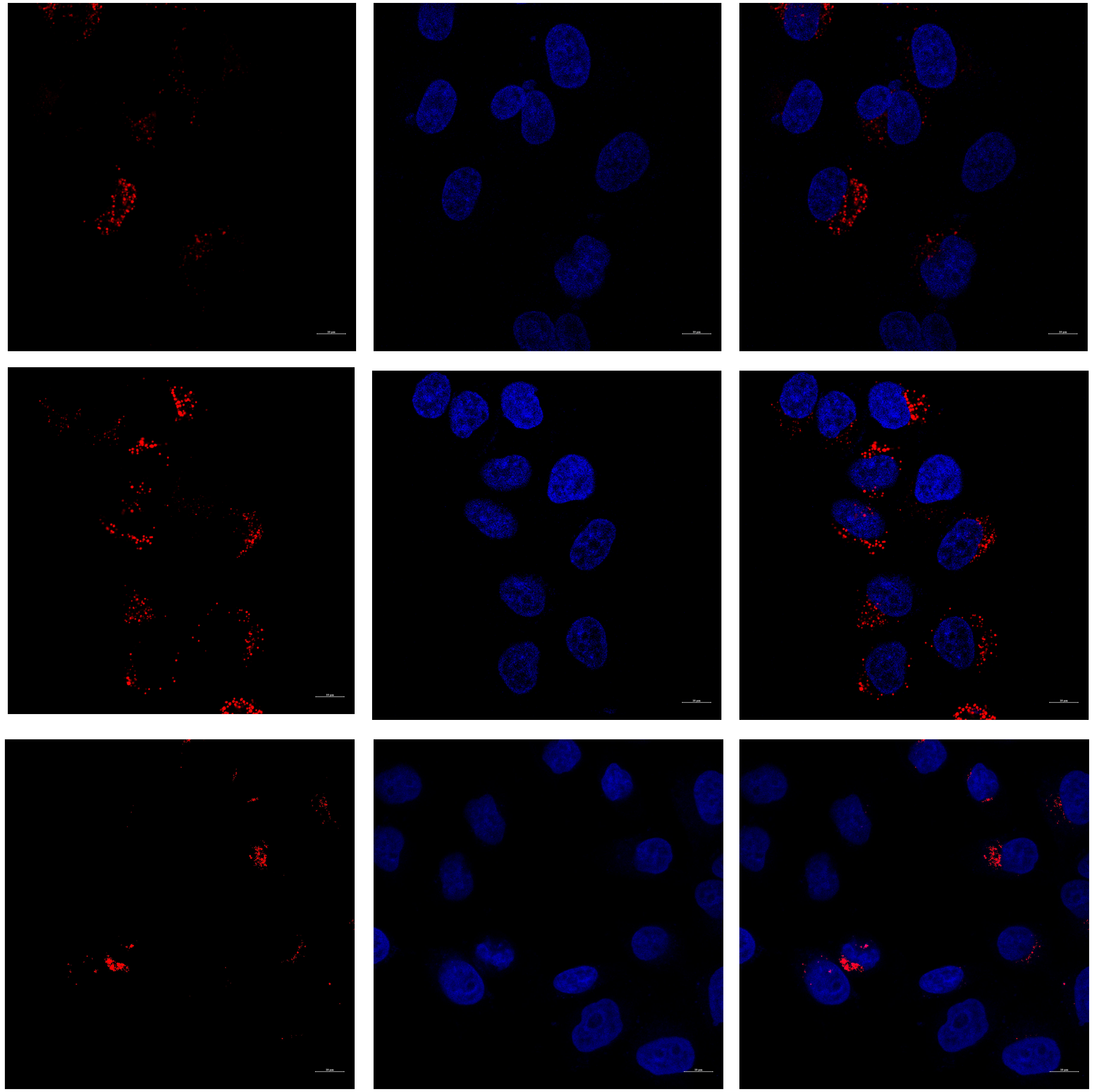

FigS5D

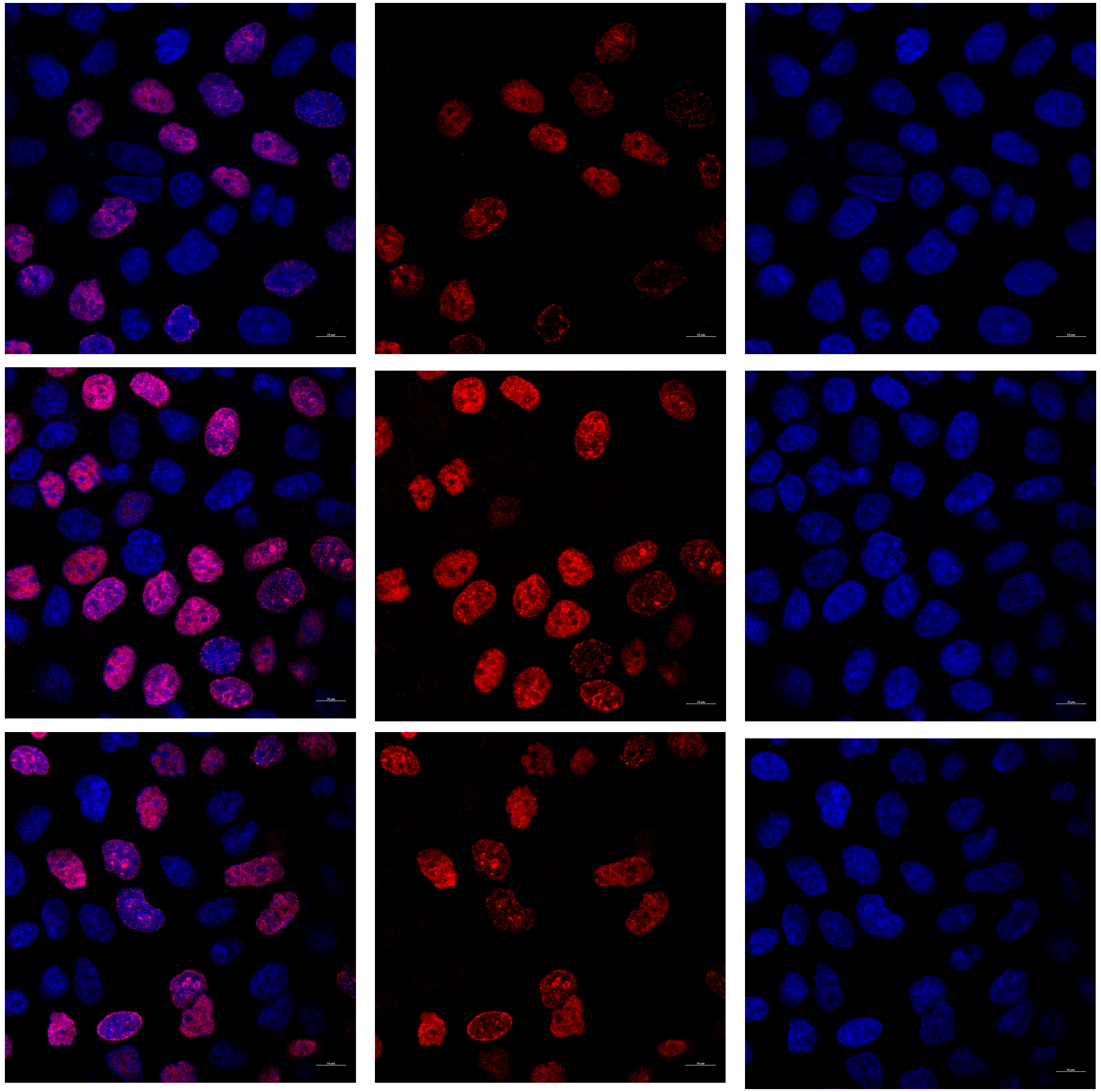

FigS7C

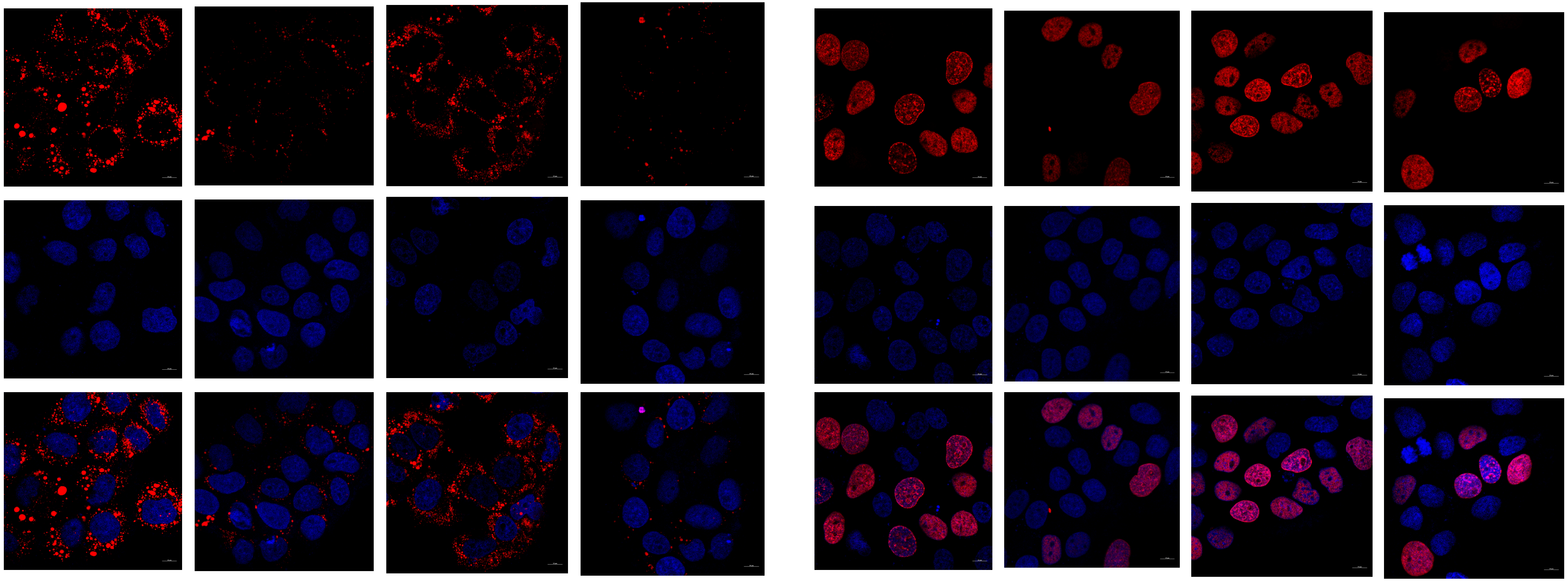

FigS8D

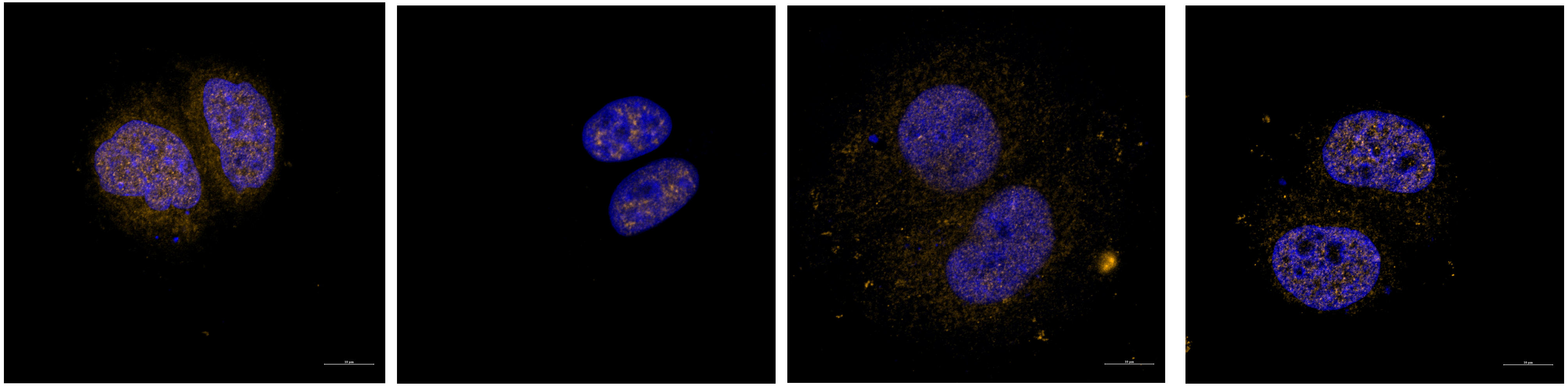

FigS12B

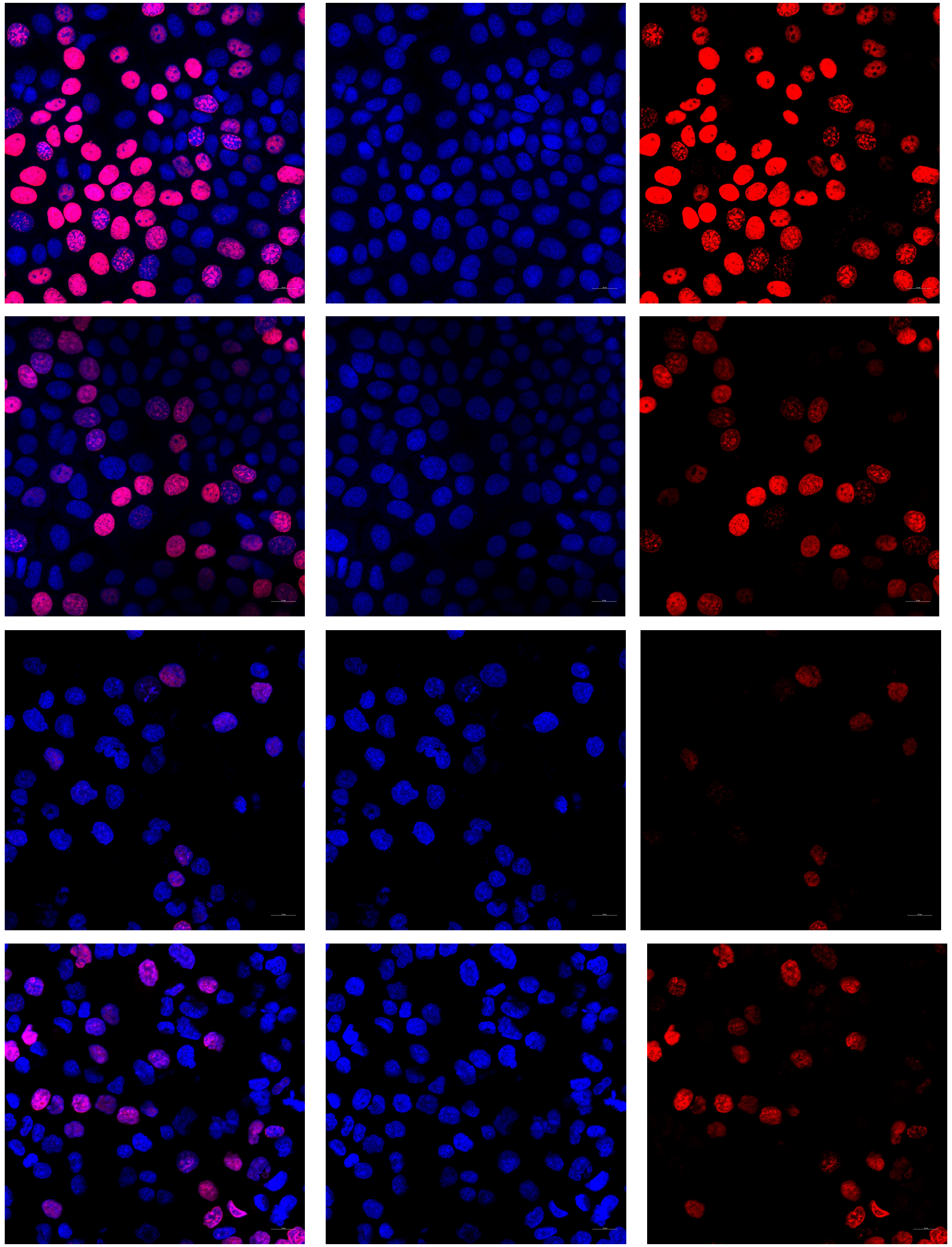

FigS12F

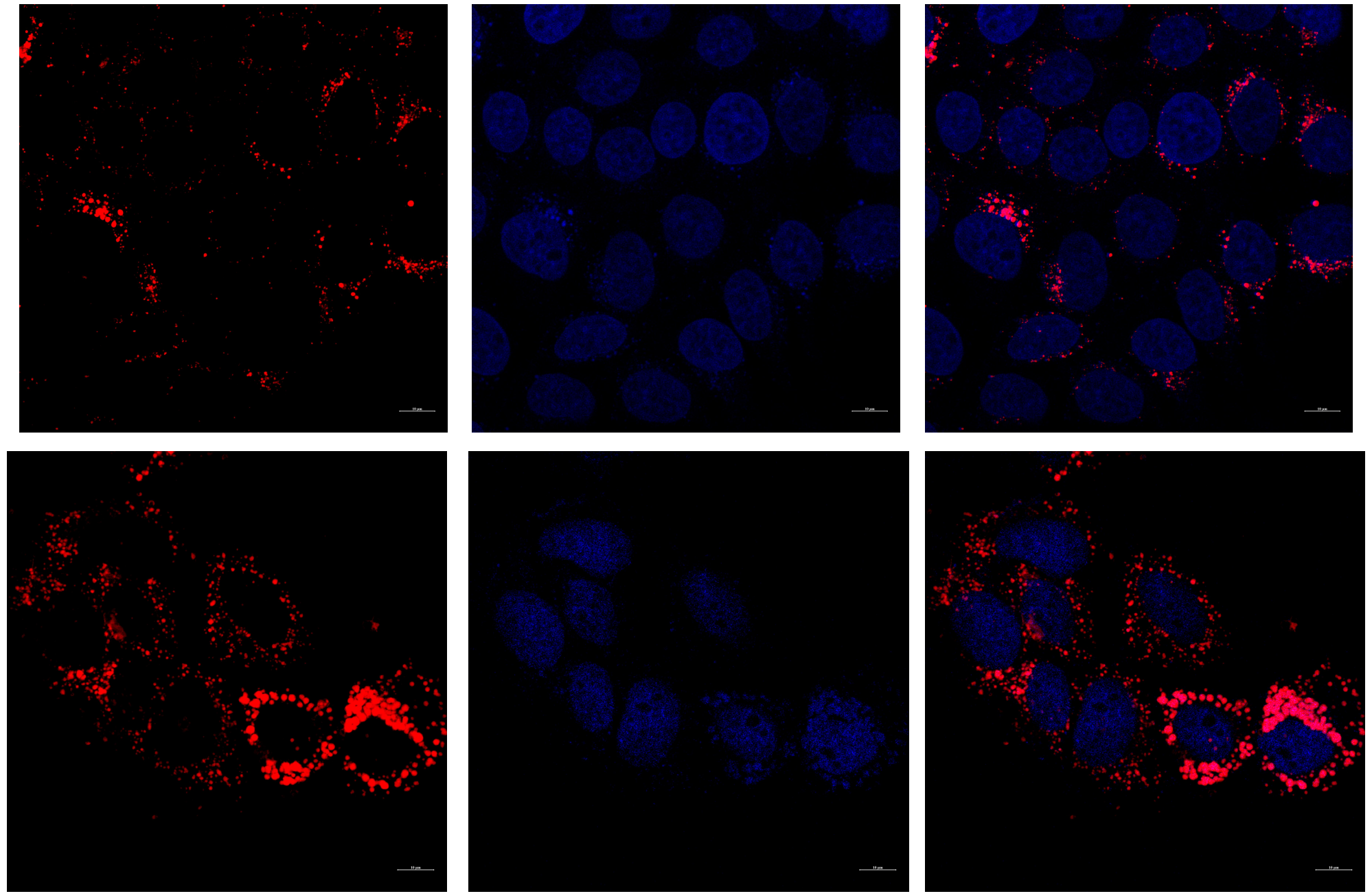

FigS14A

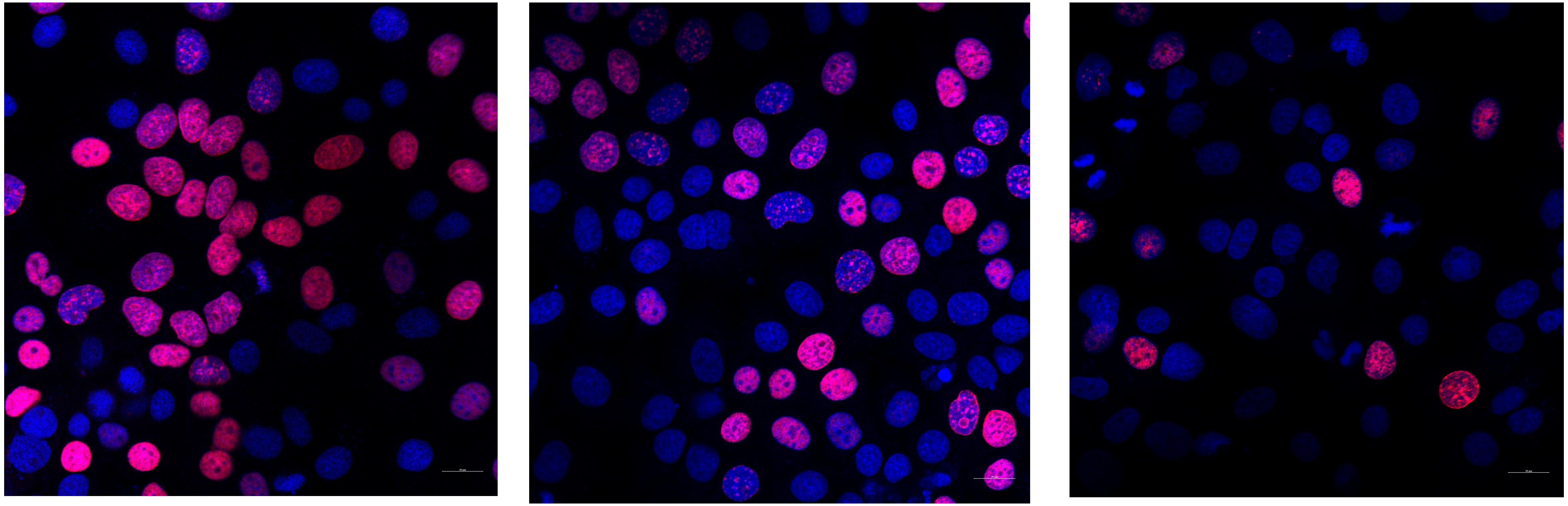

FigS15B

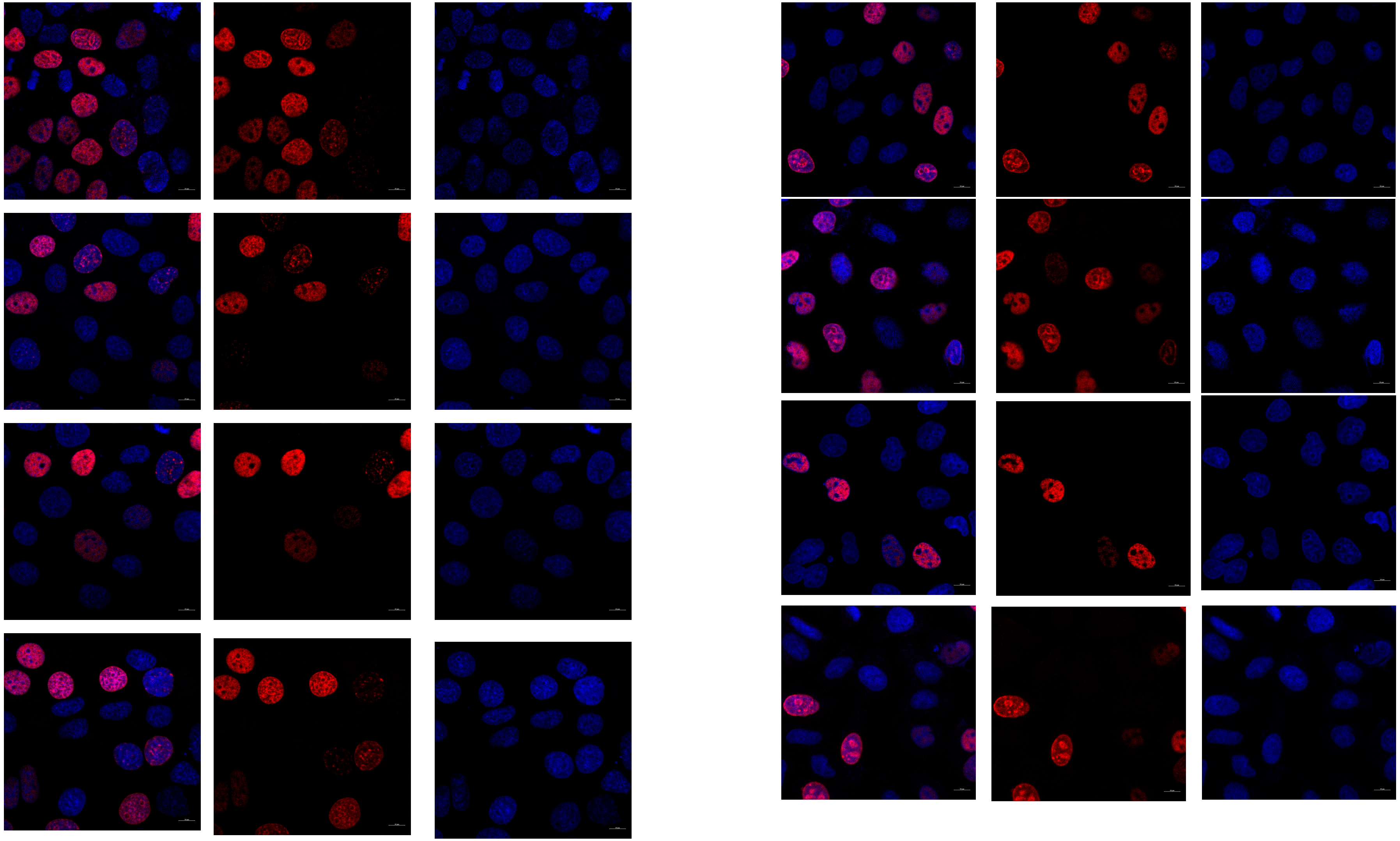

Supplement: Supplementary file 1 [file ADVS-9999-e78039-s002.pdf]
