# Supplementary material for: USP4‐Dependent CHAF1B Stabilization Regulates Distinct SETDB1 Ubiquitin States Linked to AKT T308 Signaling and Lipogenic Remodeling in HCC
Source: Adv Sci (Weinh). 2026 Sep 29:e78039. Online ahead of print. doi: 10.1002/advs.78039 (PMC13624420; doi:10.1002/advs.78039)
Supplement: Supplementary file 2 [file ADVS-9999-e78039-s003.pdf]

Fig. 1H

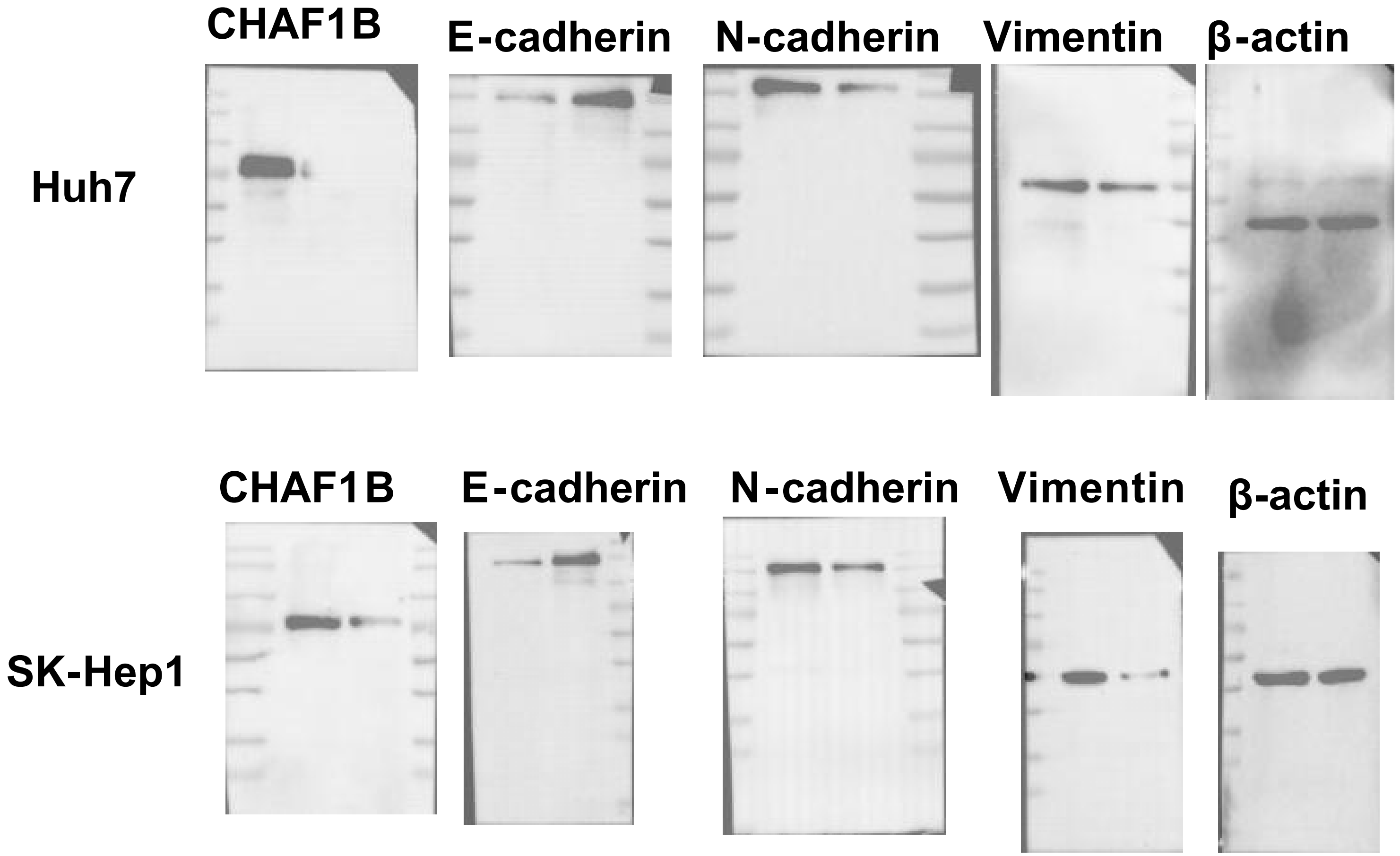

CHAF1B
E-cadherin
N-cadherin
Vimentin
β-actin
Huh7
CHAF1B
E-cadherin
N-cadherin
Vimentin
β-actin
SK-Hep1

Fig. 1H

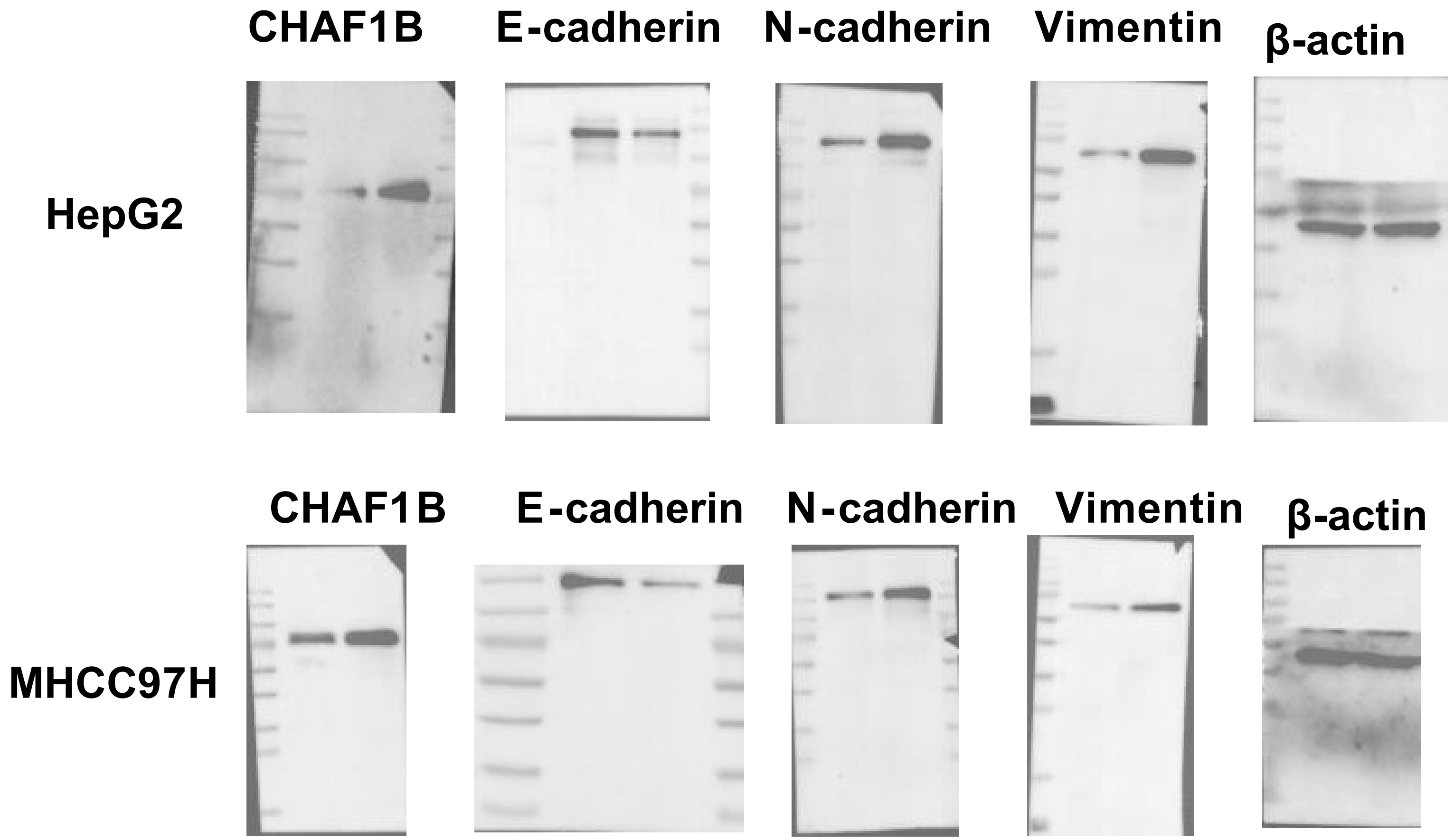
CHAF1B
E-cadherin
N-cadherin
Vimentin
β-actin
HepG2
CHAF1B
E-cadherin
N-cadherin
Vimentin
β-actin
MHCC97H

Fig.2D Huh7

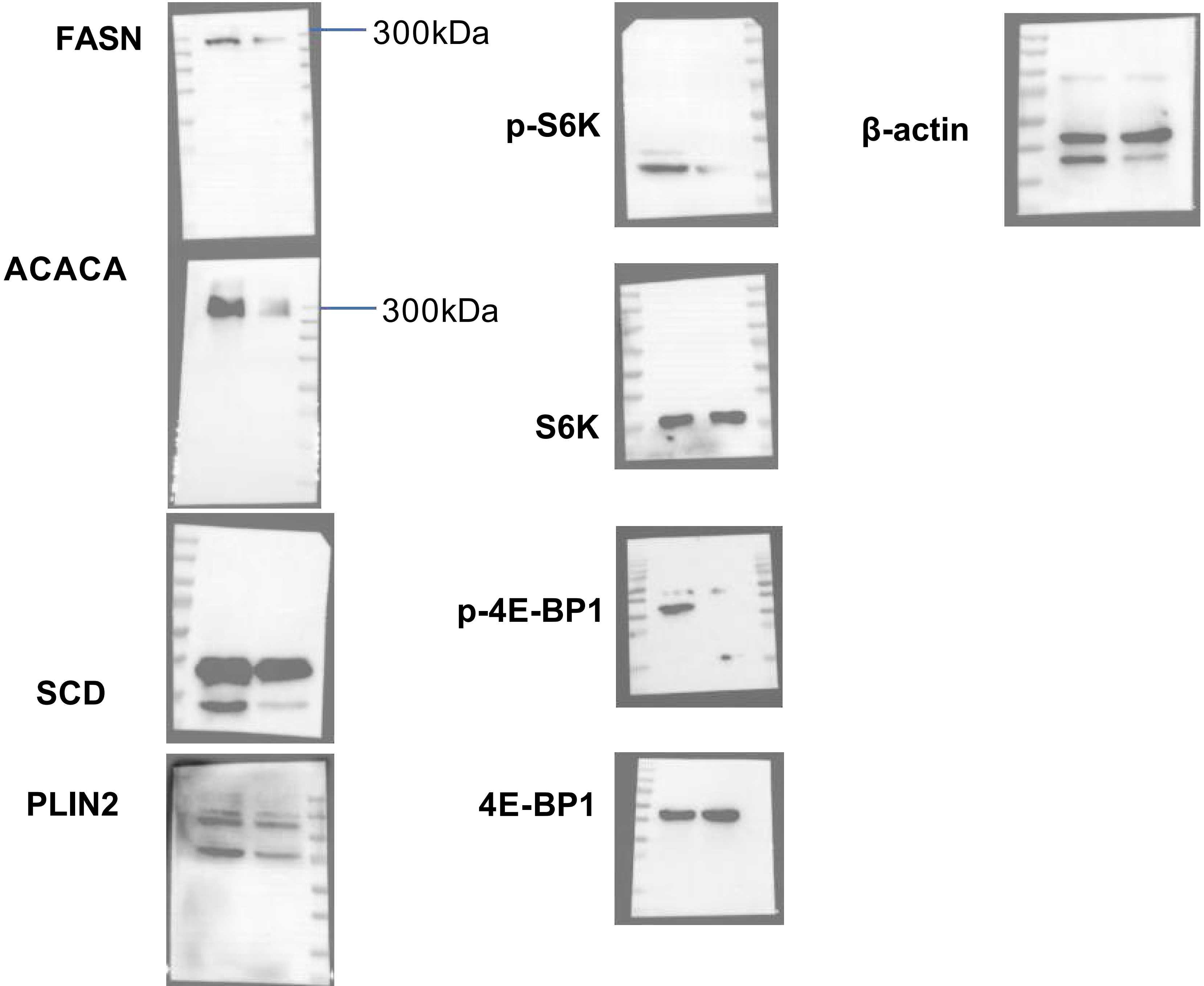
FASN
300kDa
ACACA
300kDa
SCD
PLIN2
p-S6K
S6K
p-4E-BP1
4E-BP1
β-actin

Fig.2D HepG2

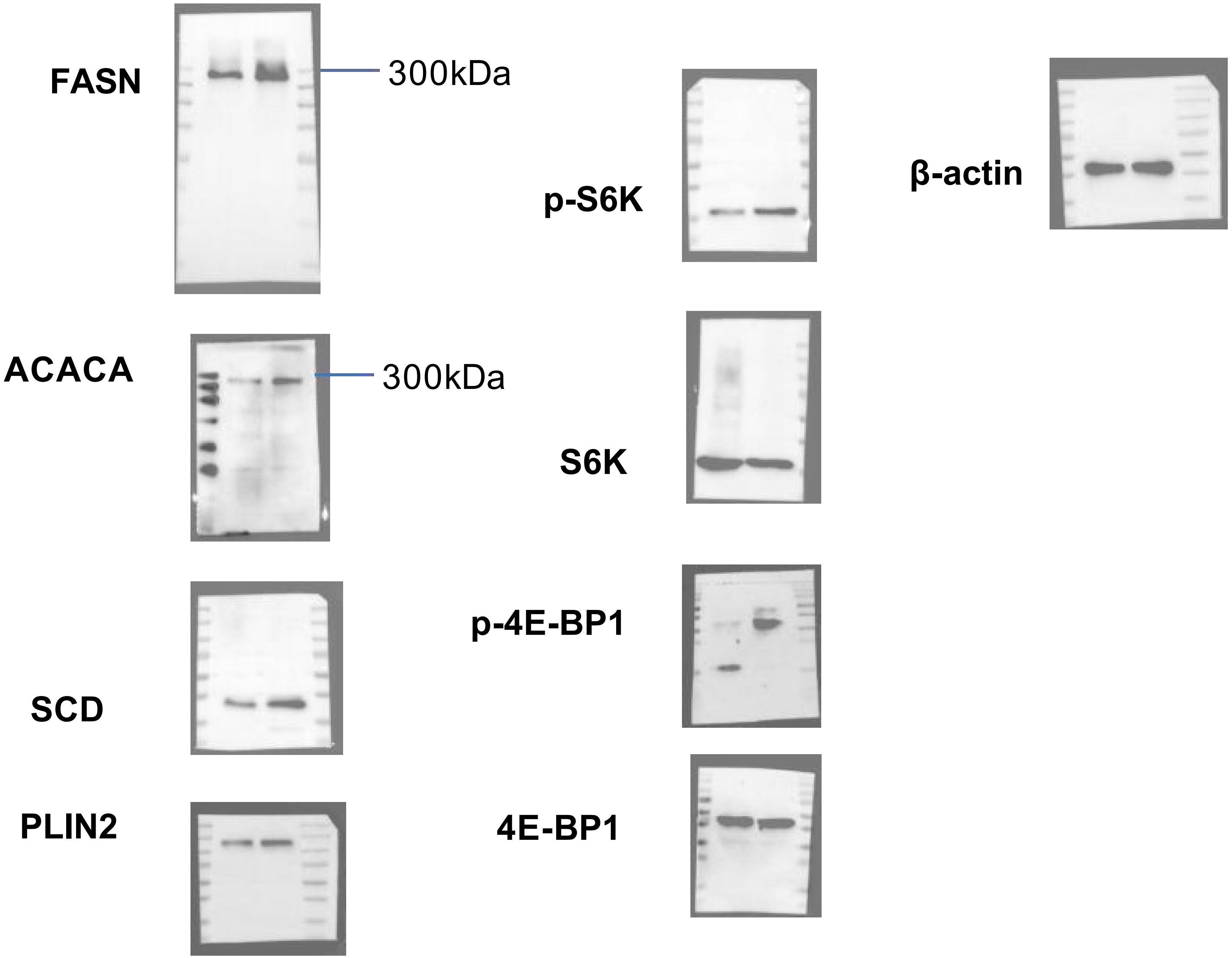
FASN
300kDa
ACACA
300kDa
SCD
PLIN2
p-S6K
S6K
p-4E-BP1
4E-BP1
β-actin

Fig.2J Huh7

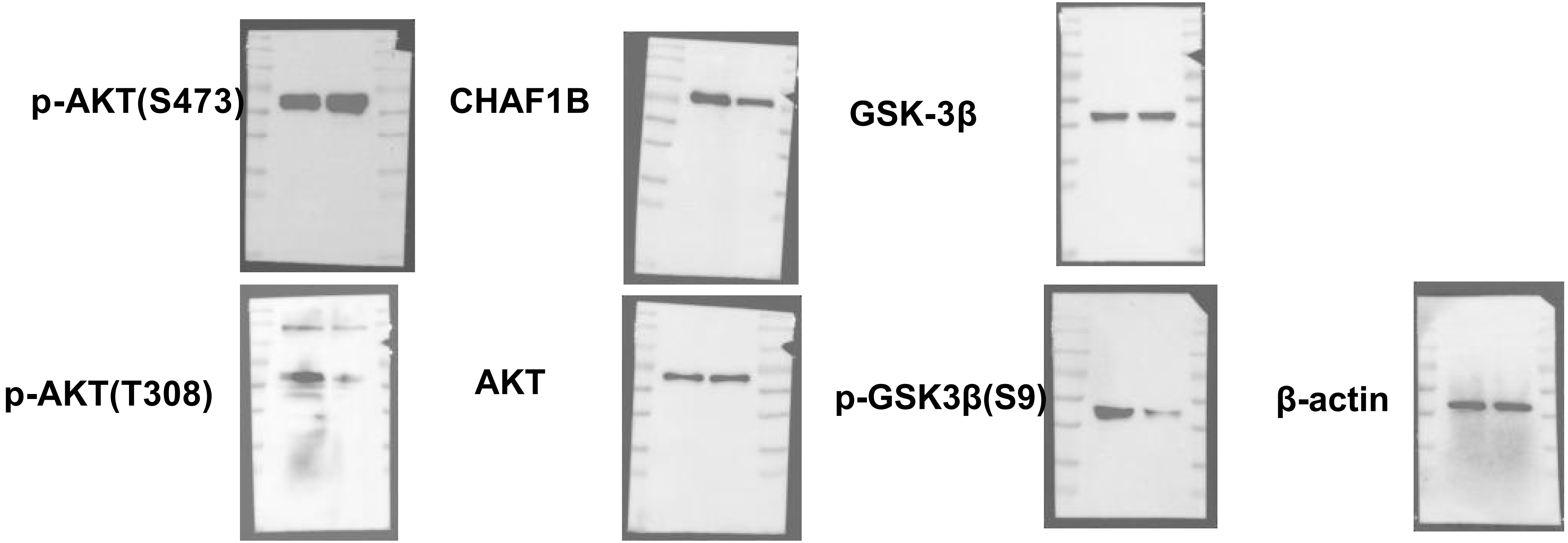

Fig.2J SK-Hep1

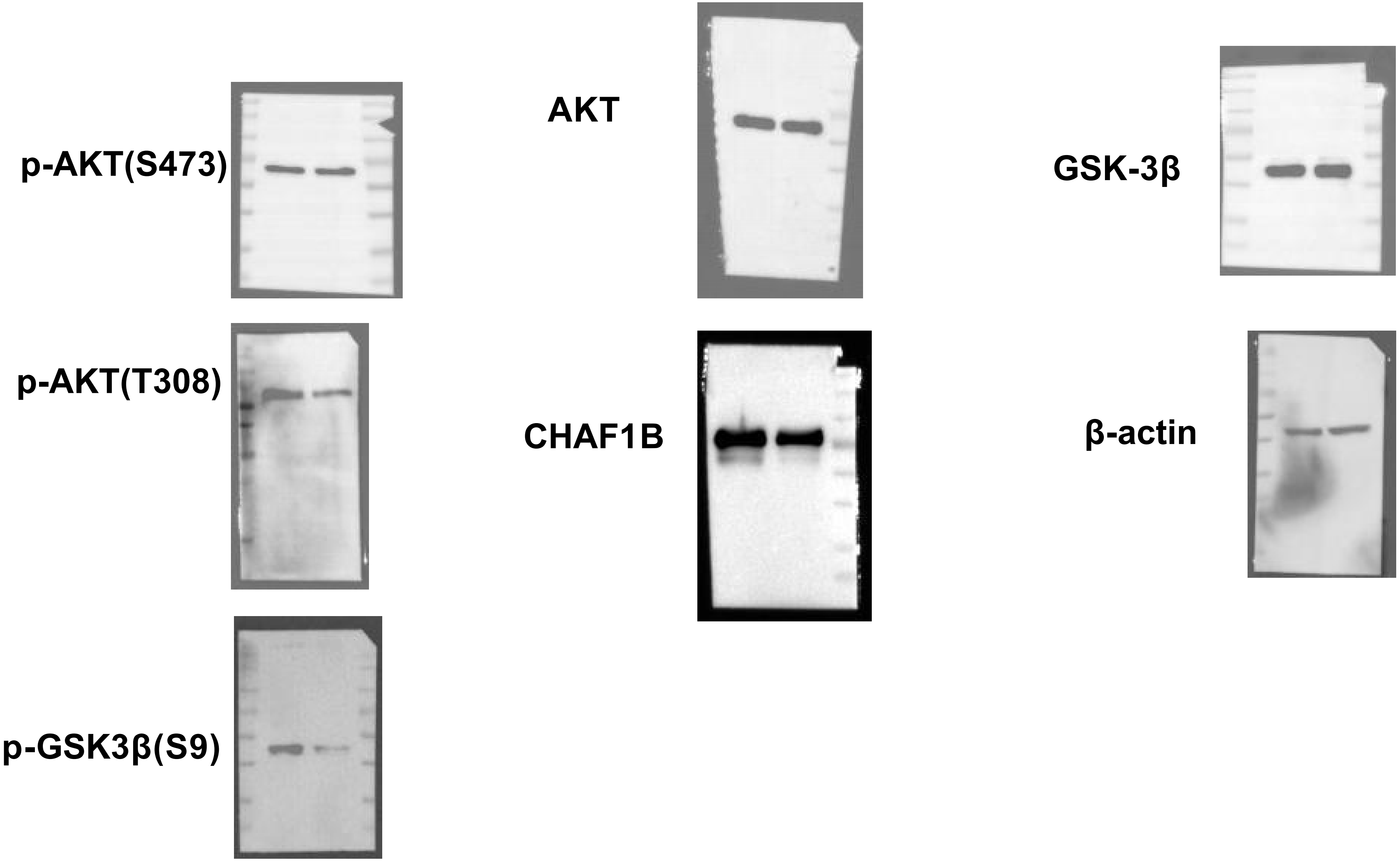

Fig.2J HepG2

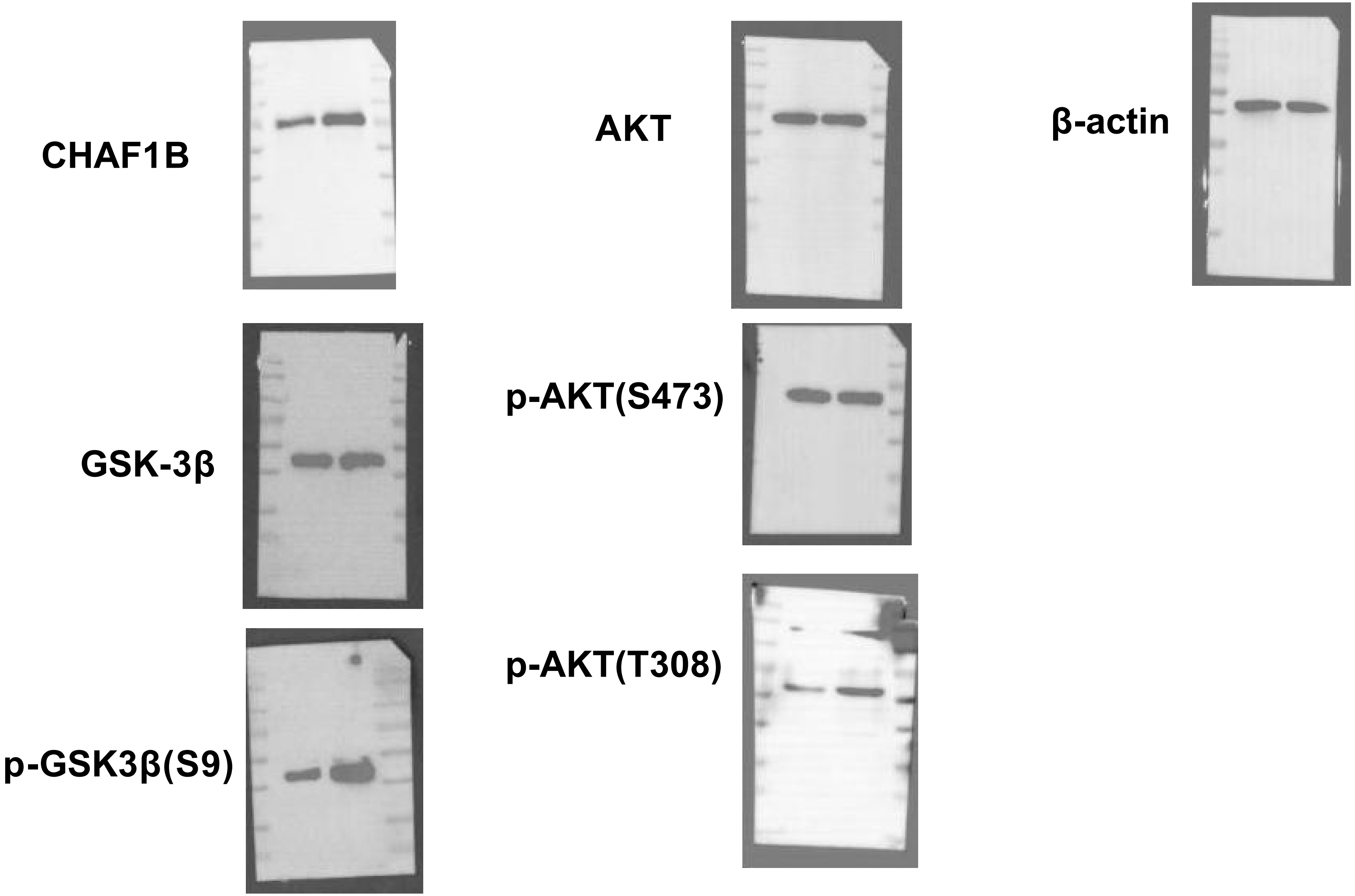

Fig.2J
MHCC97H

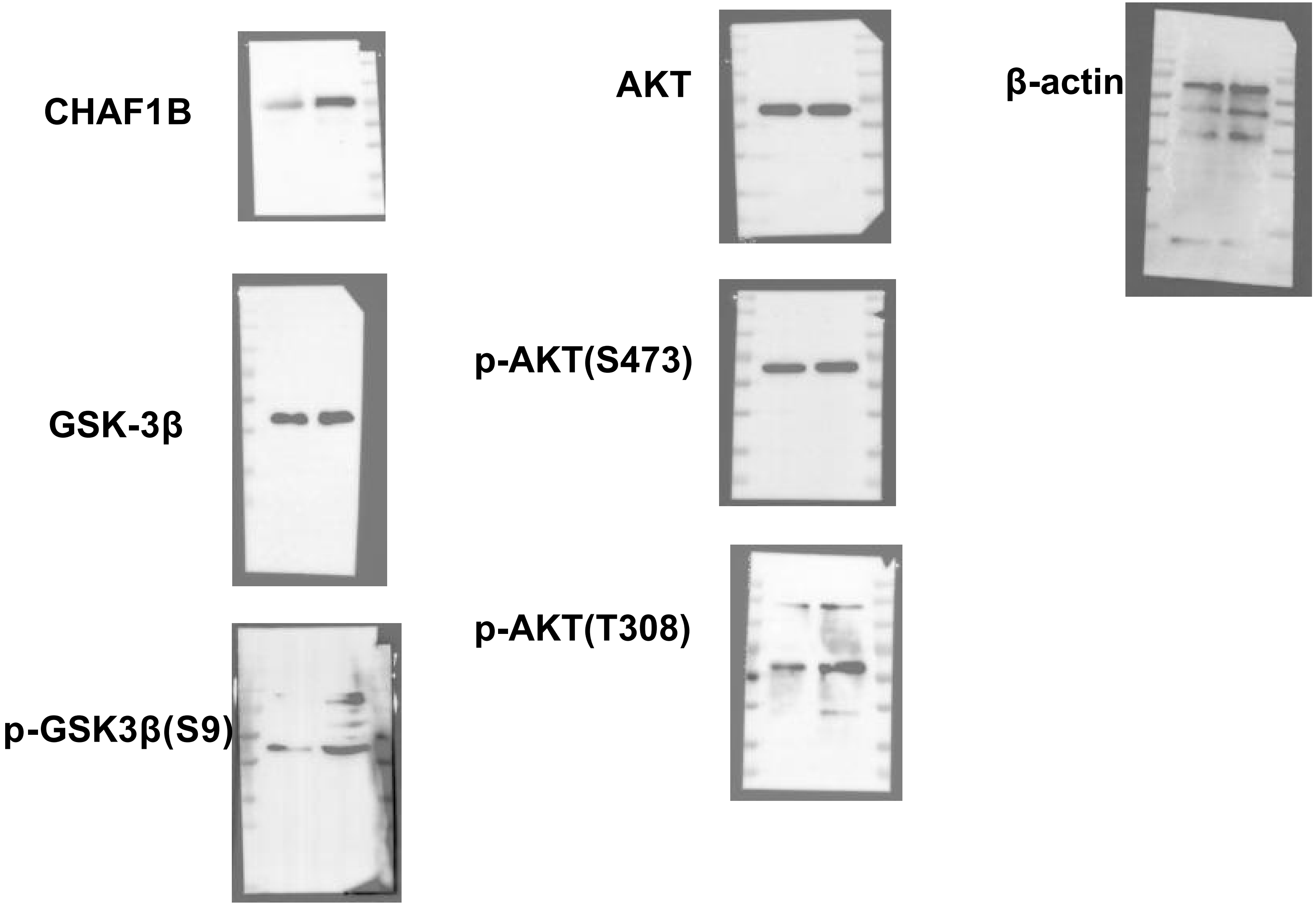

Fig.2K
HepG2

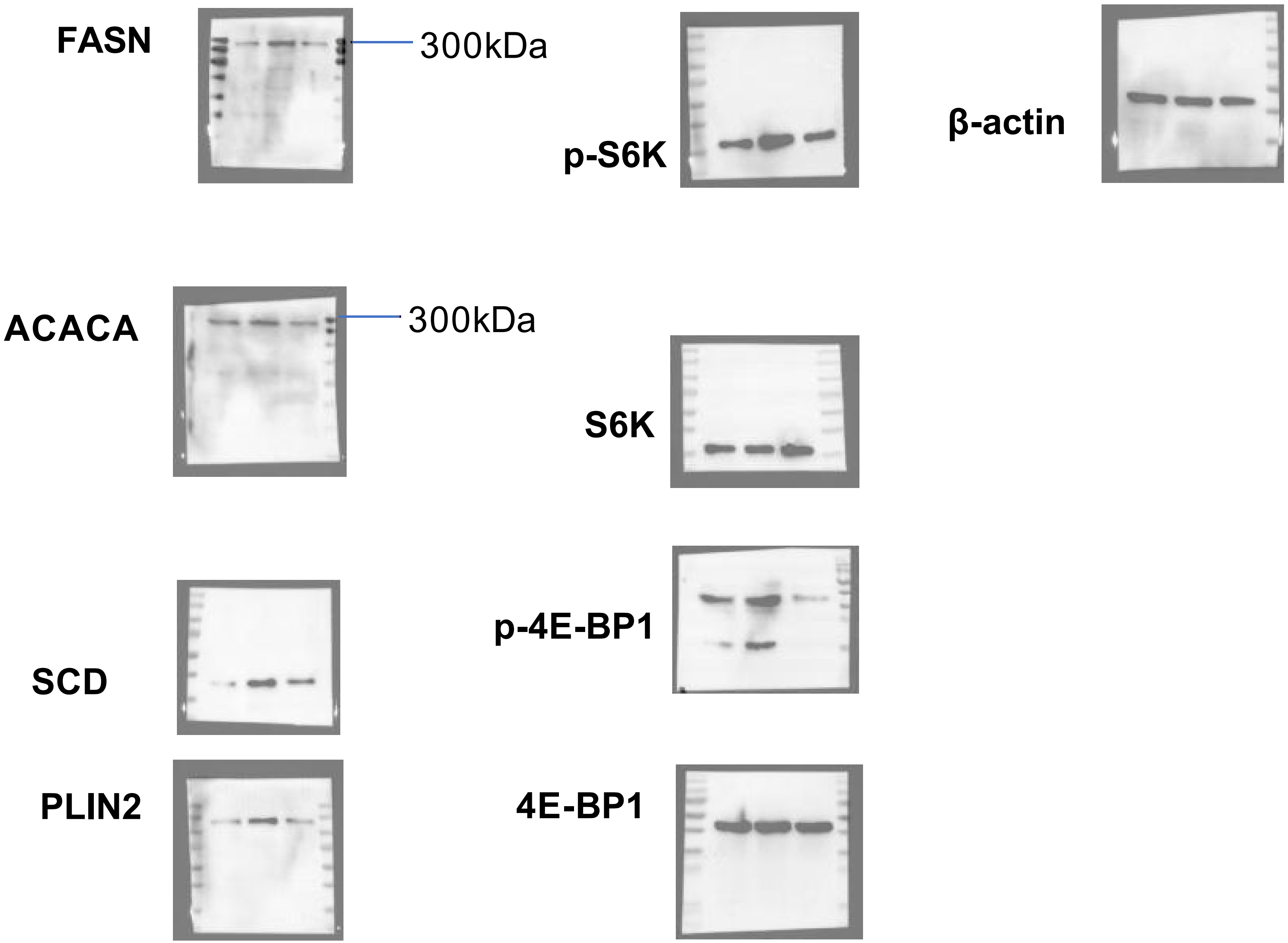
FASN
300kDa
p-S6K
β-actin
ACACA
300kDa
S6K
SCD
p-4E-BP1
PLIN2
4E-BP1

Fig.2T

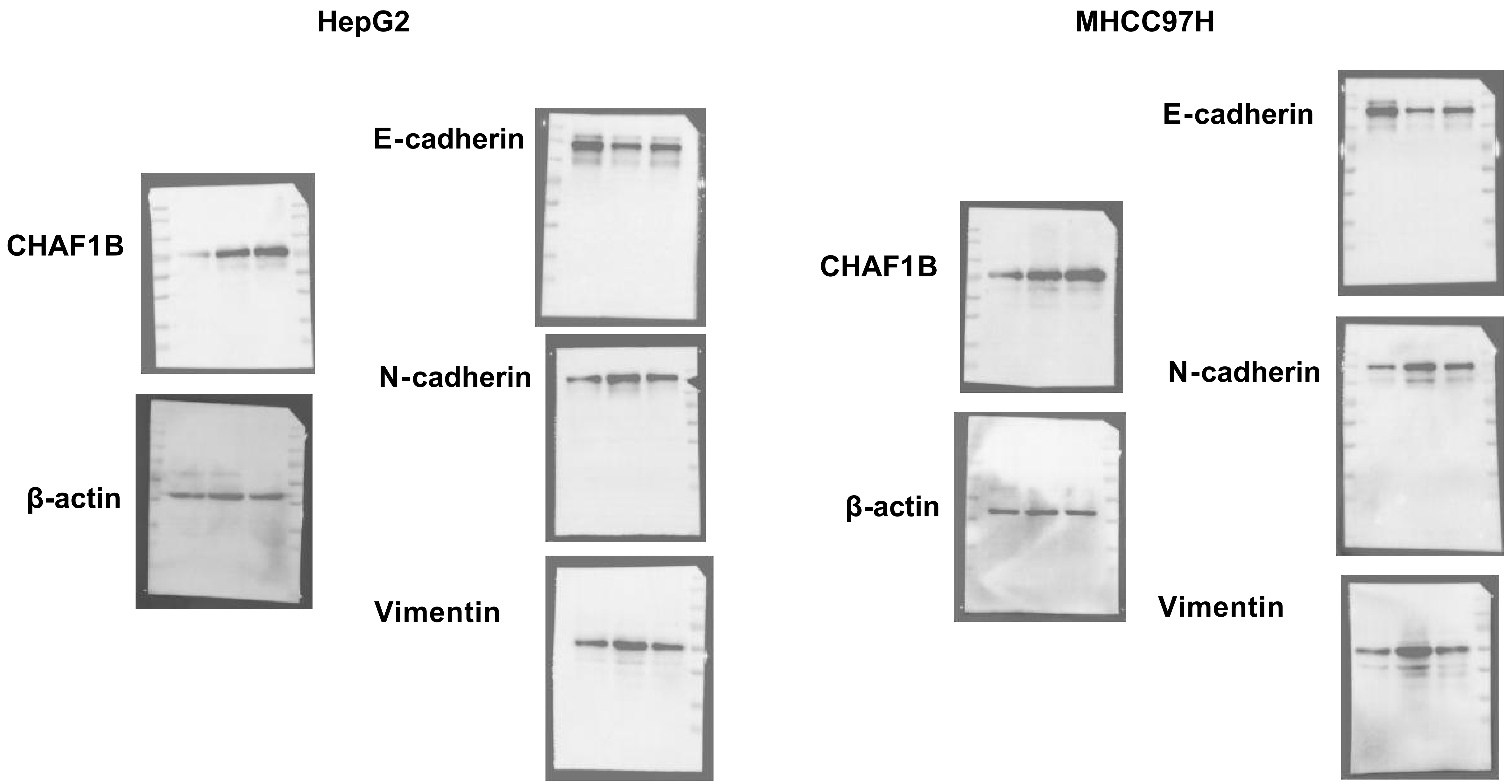

HepG2
MHCC97H
CHAF1B
β-actin
E-cadherin
N-cadherin
Vimentin
CHAF1B
β-actin
E-cadherin
N-cadherin
Vimentin

Fig.3E

Huh7

IB: CHAF1B

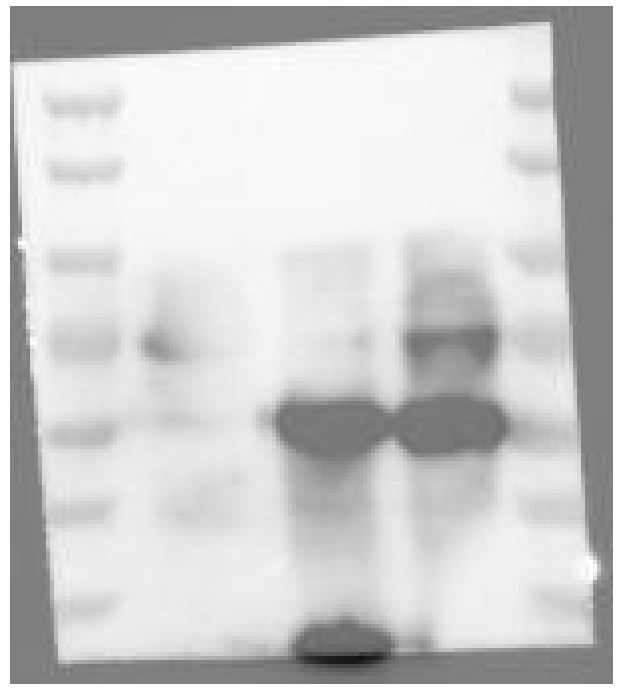

IB: CHAF1B

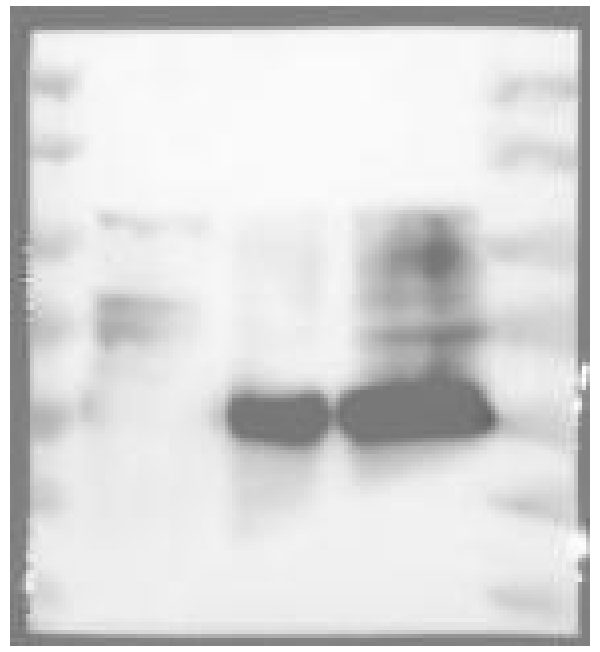

IB: SETDB1

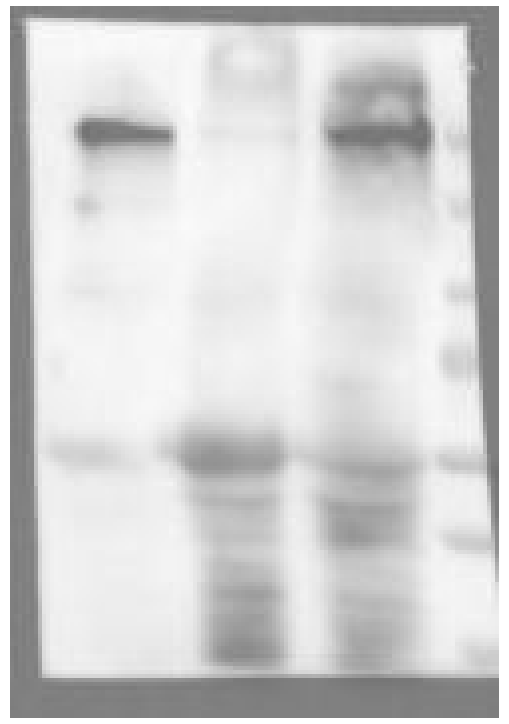

IB: SETDB1

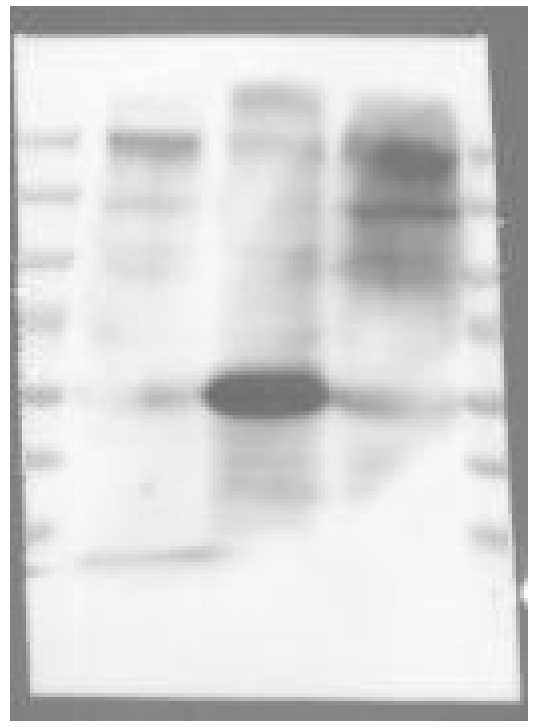

Fig.3E

SK-Hep1

IB: CHAF1B

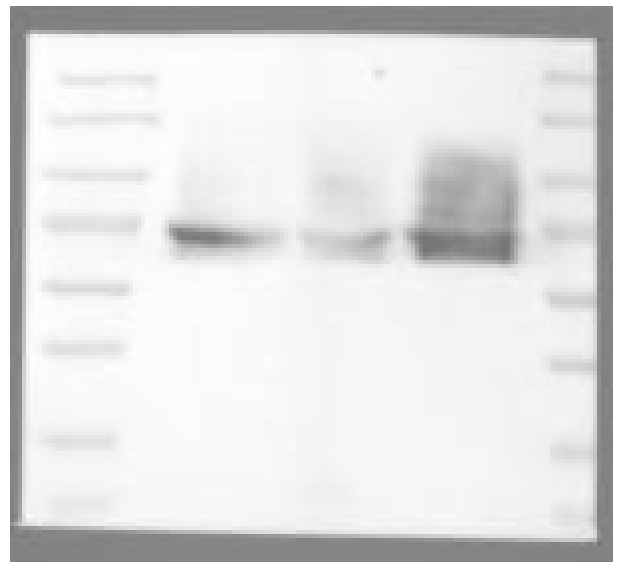

IB: CHAF1B

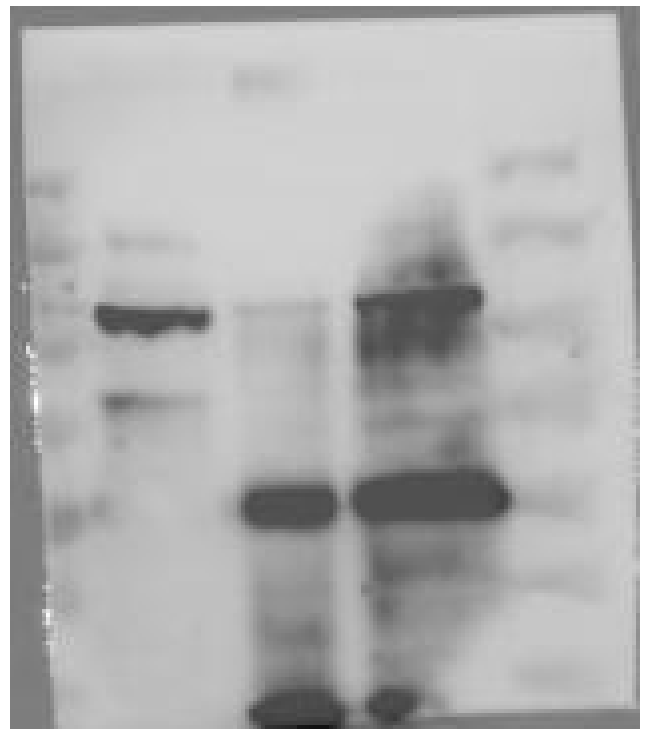

IB: SETDB1

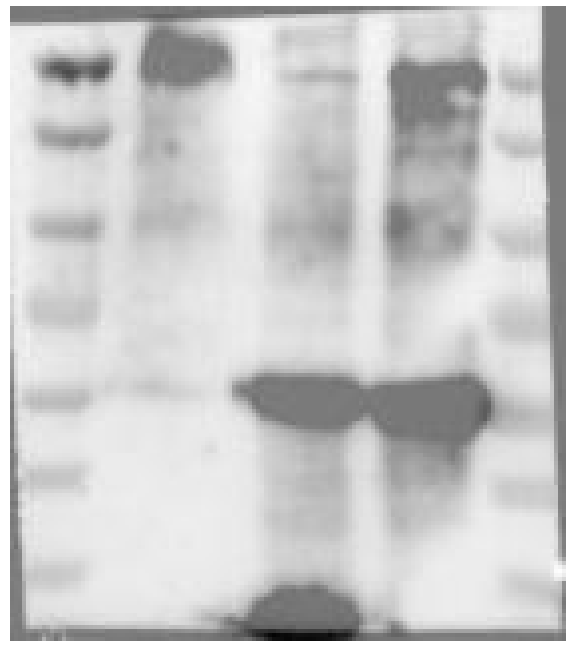

IB: SETDB1

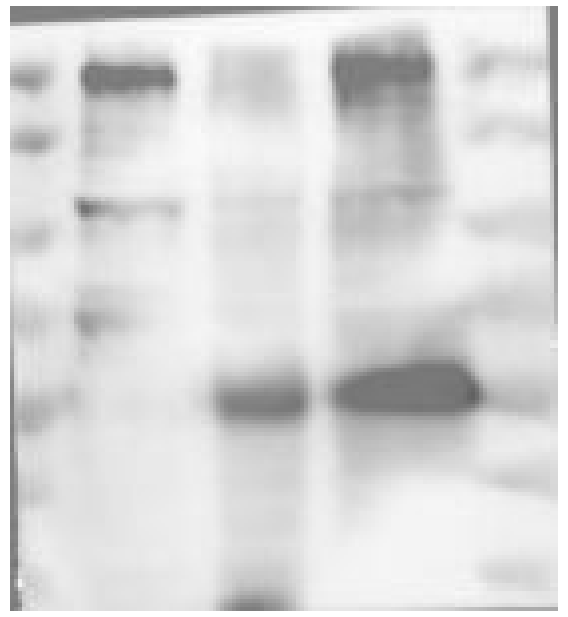

Fig.3F

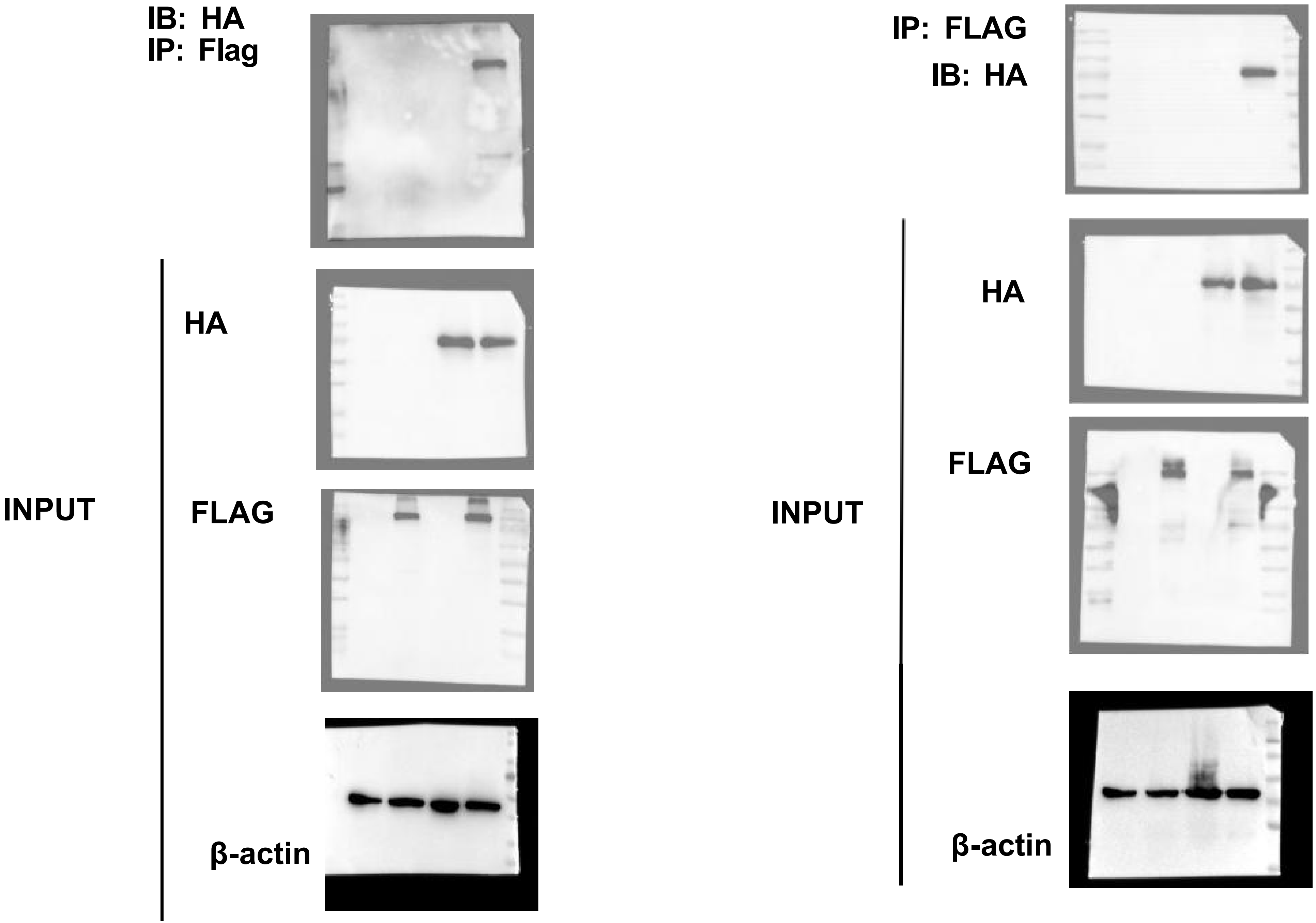
IB: HA
IP: Flag
HA
FLAG
INPUT
β-actin
IP: FLAG
IB: HA
HA
FLAG
INPUT
β-actin

Fig.3G

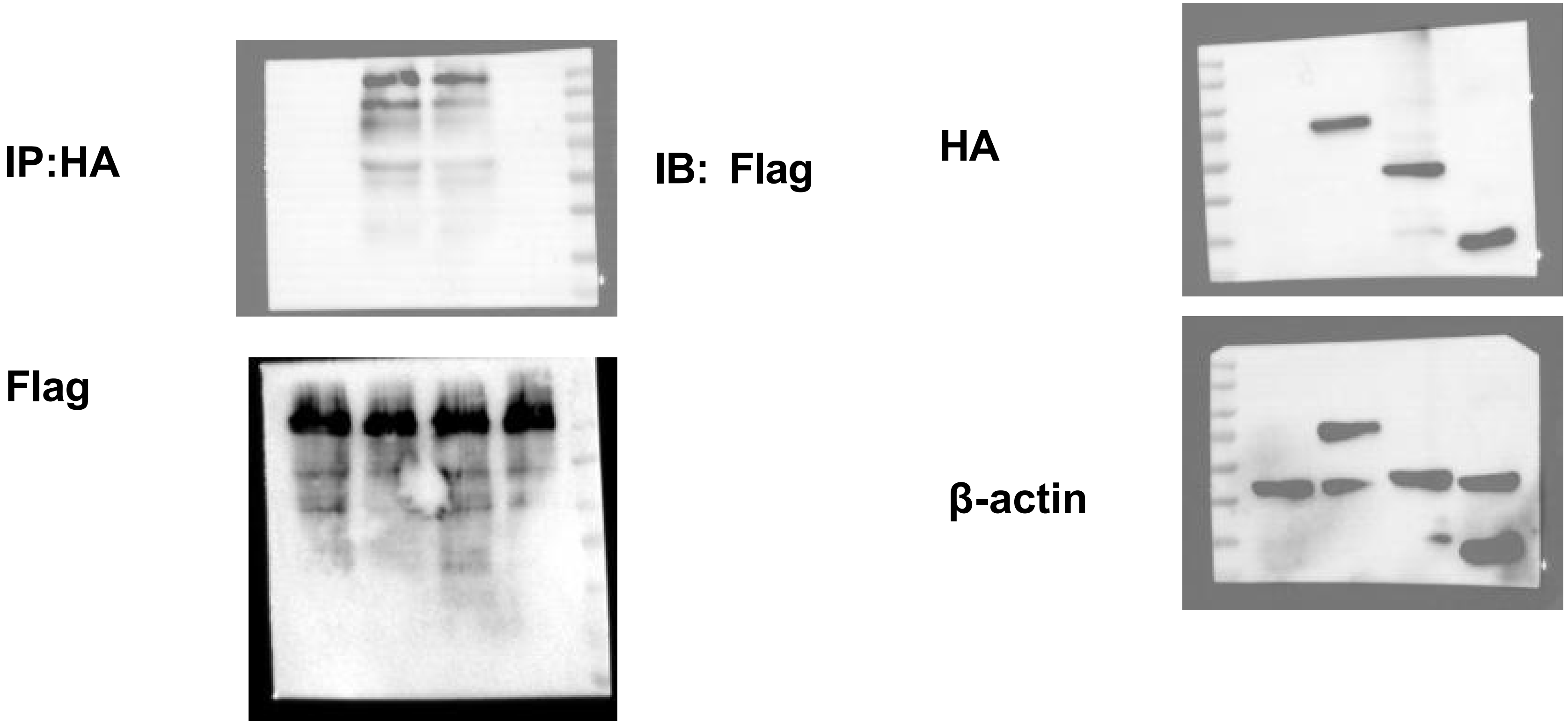

Fig.3H

Huh7

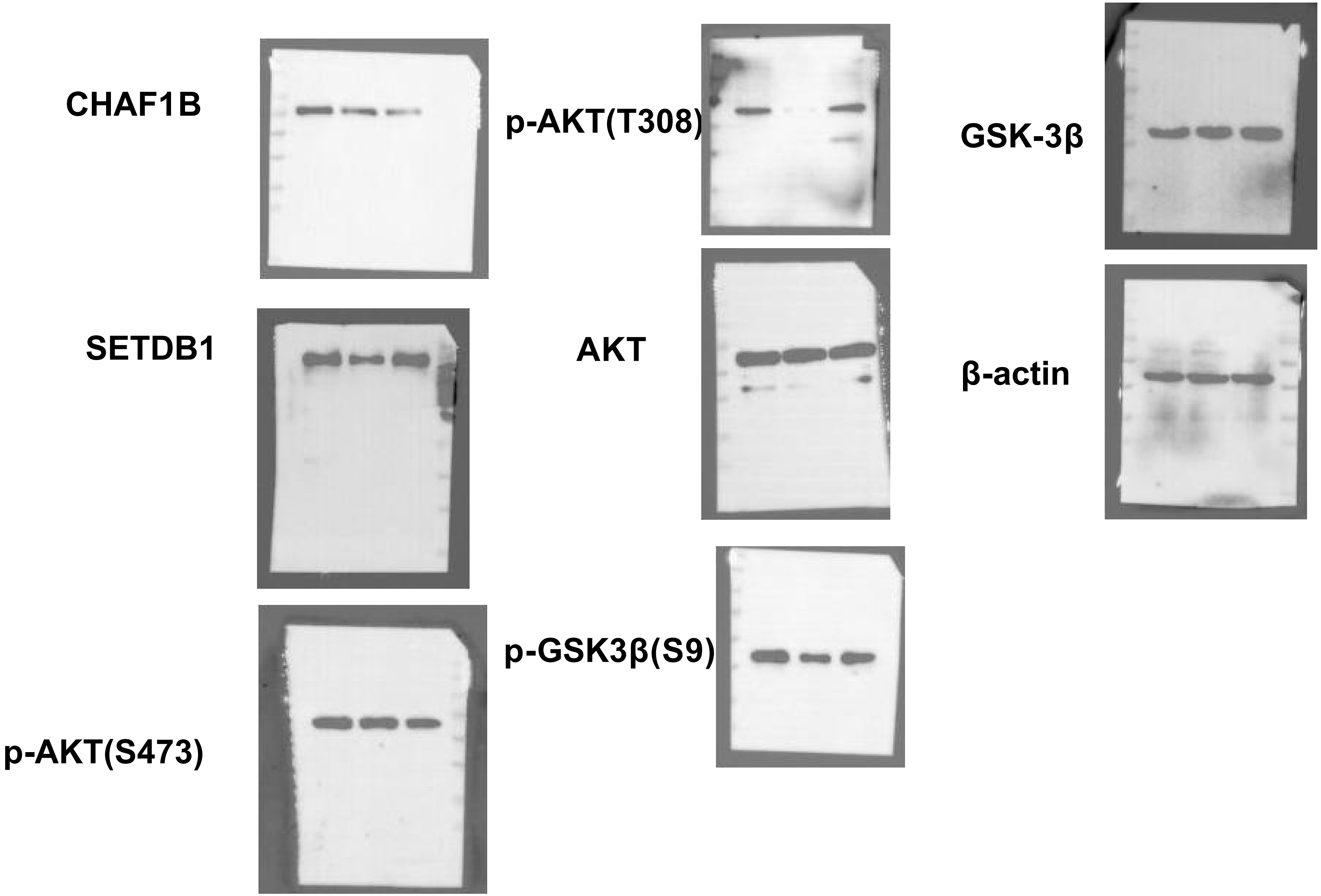
CHAF1B
p-AKT(T308)
GSK-3β
SETDB1
AKT
β-actin
p-GSK3β(S9)
p-AKT(S473)

Fig.3H

SK-Hep1

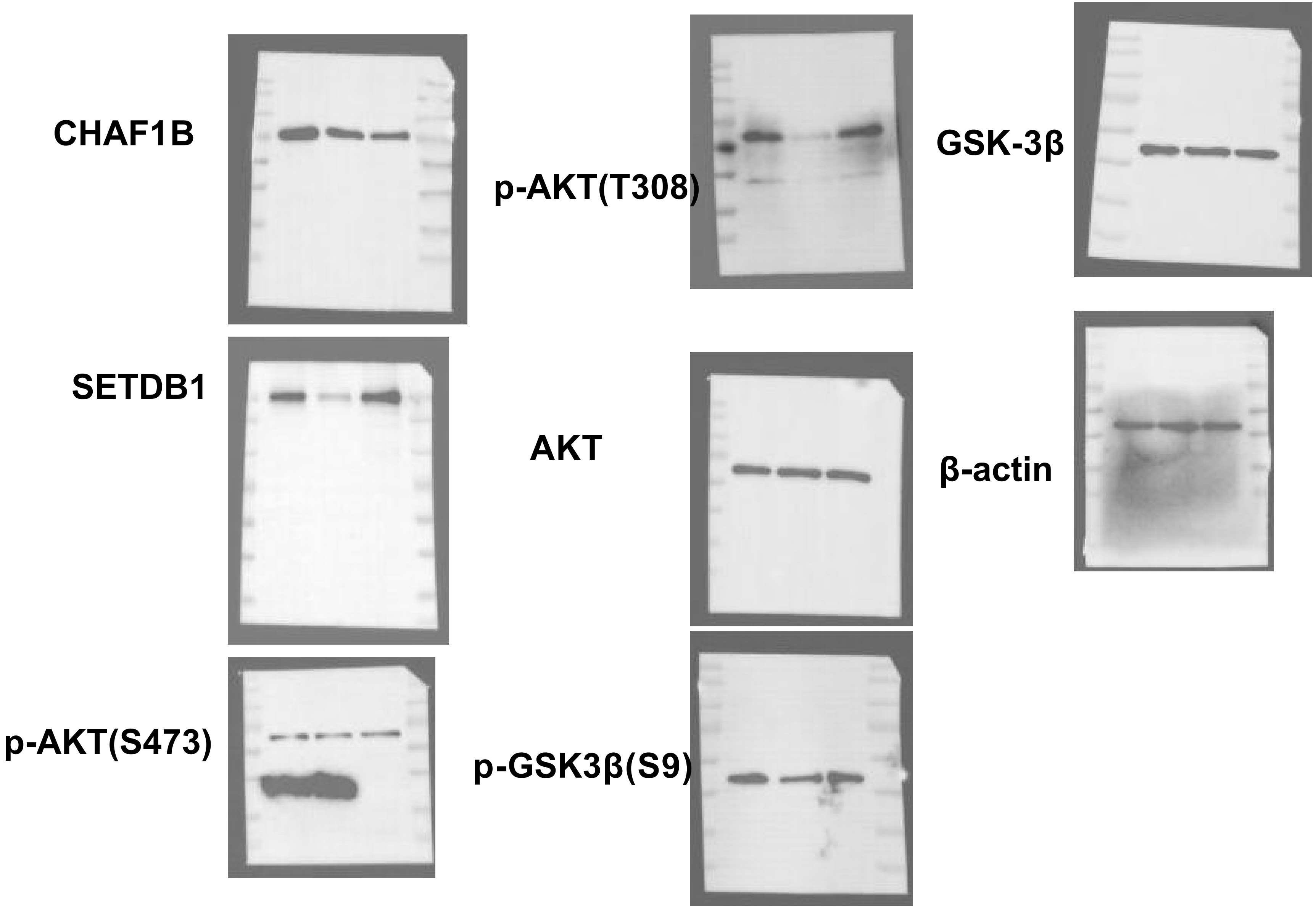

Fig.3I

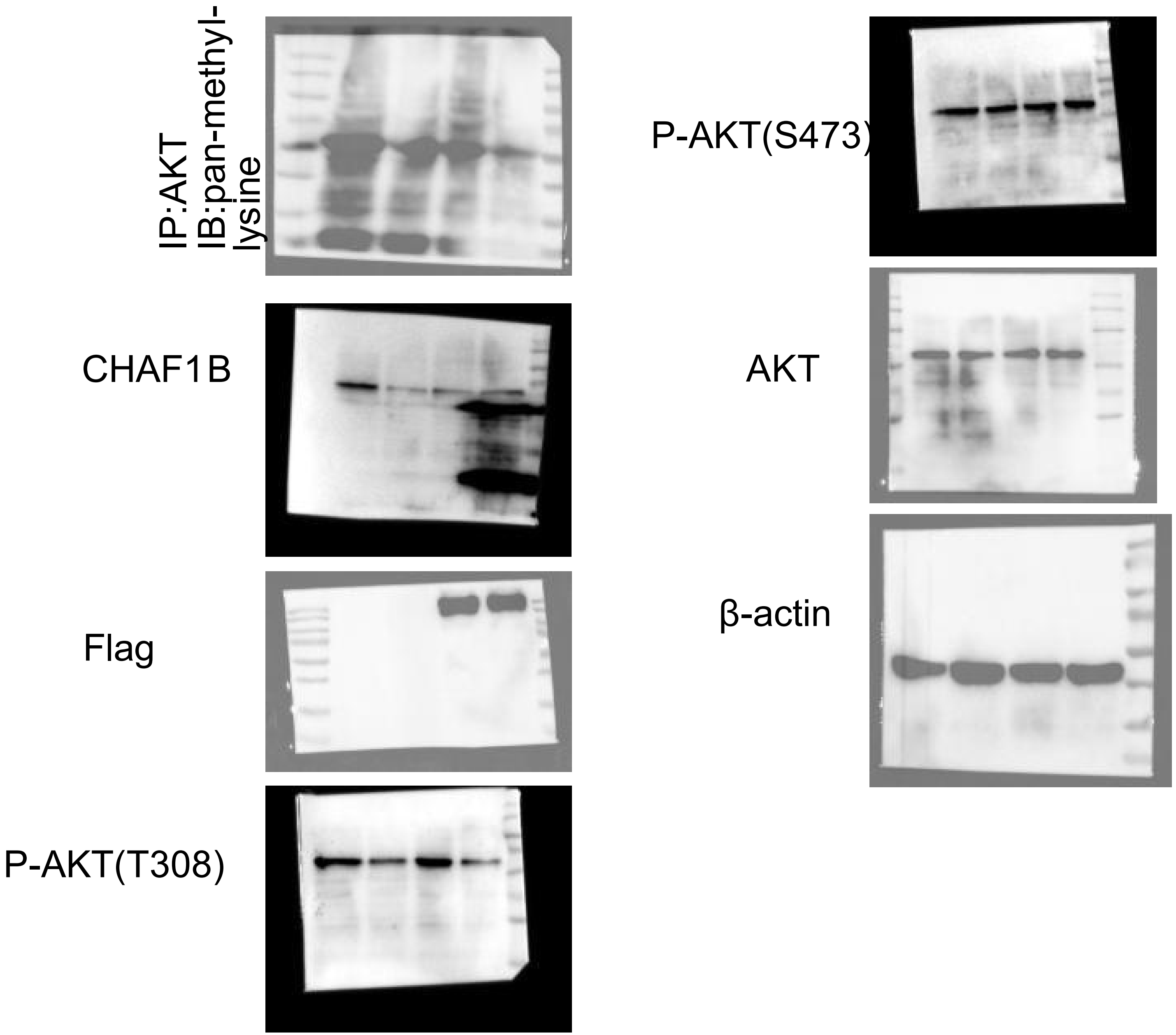
IP:AKT
IB:pan-methyl-
lysine
CHAF1B
Flag
P-AKT(T308)
P-AKT(S473)
AKT
β-actin

Fig.3O

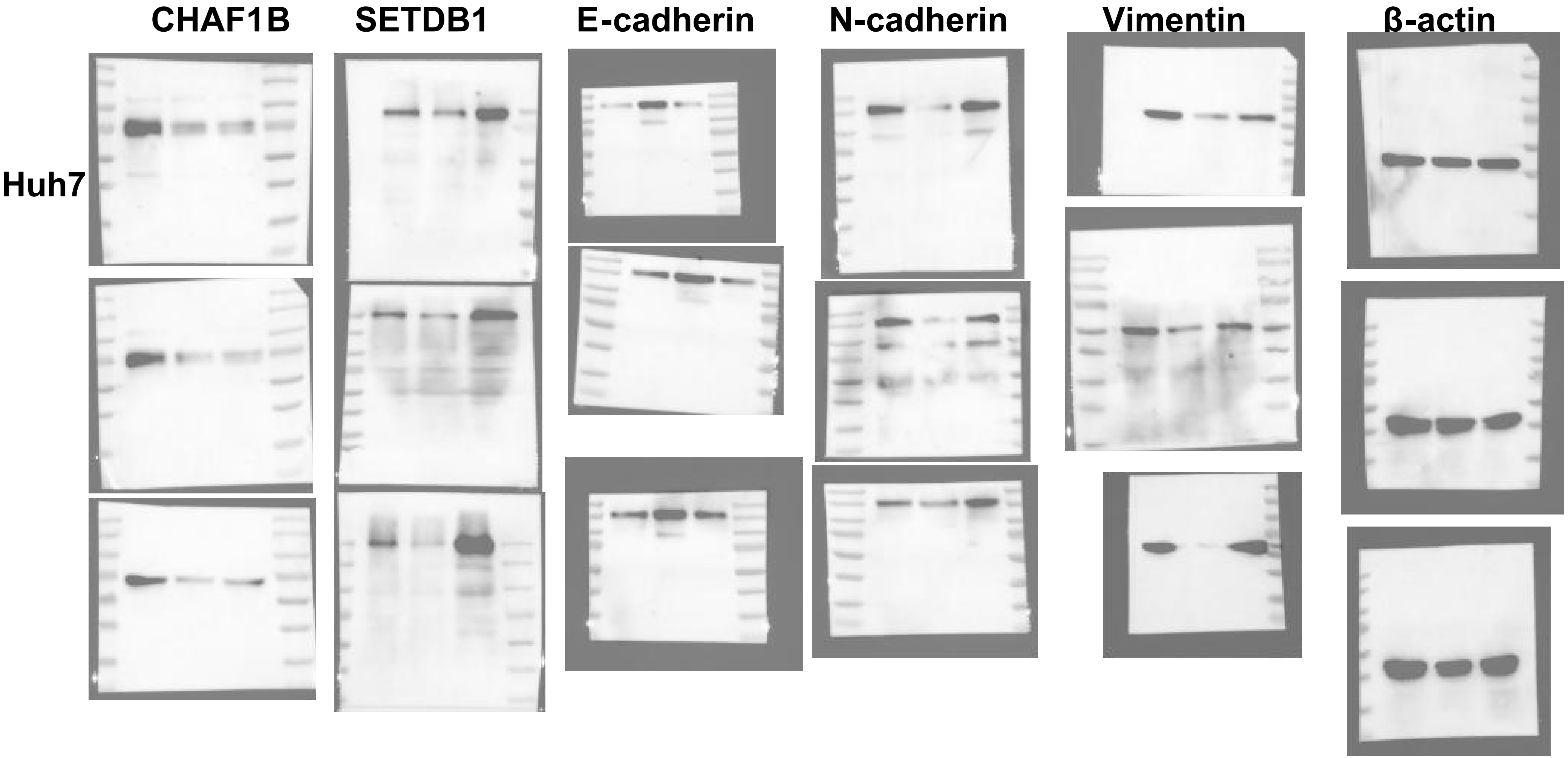

Fig.3O

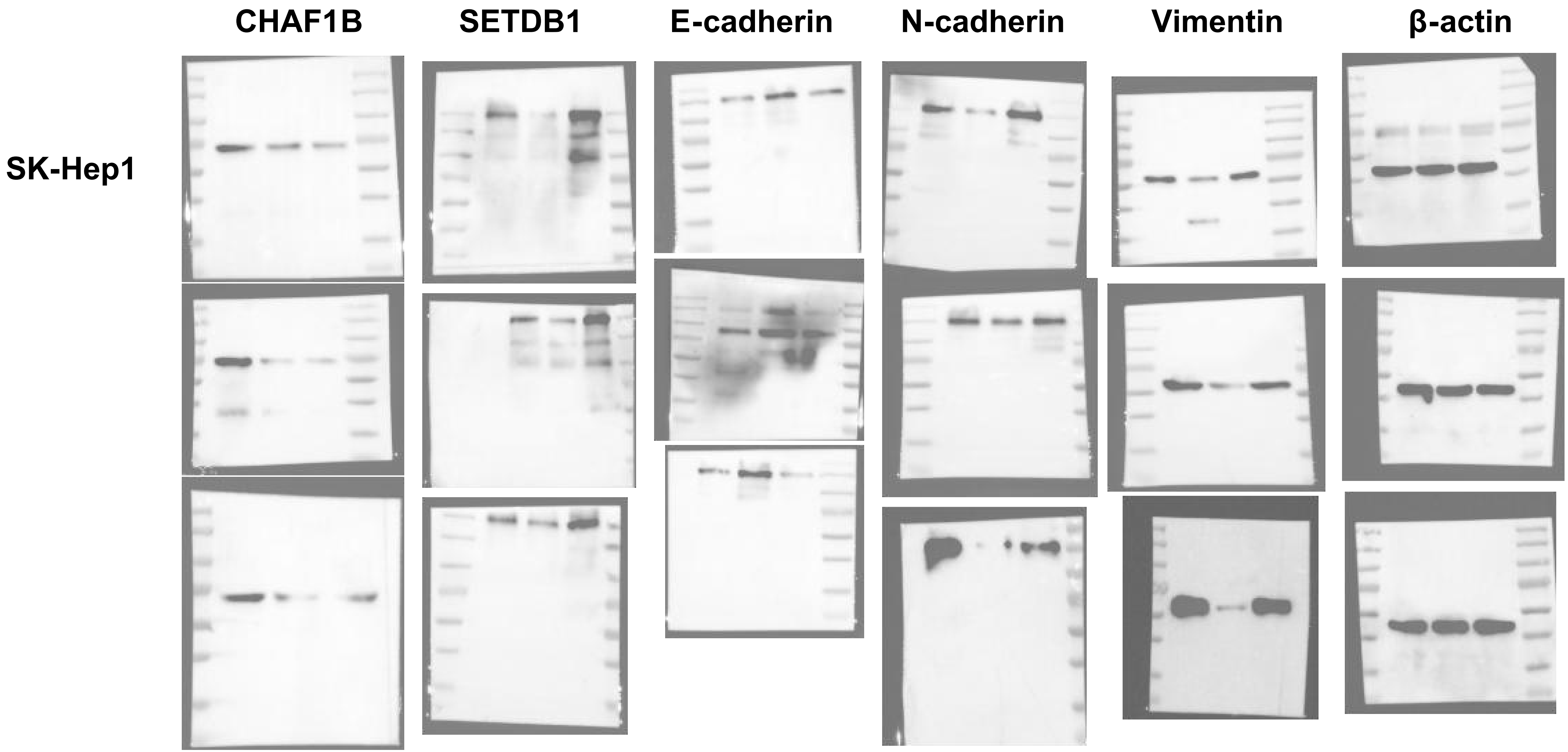
CHAF1B
SETDB1
E-cadherin
N-cadherin
Vimentin
β-actin
SK-Hep1

Fig. 4A

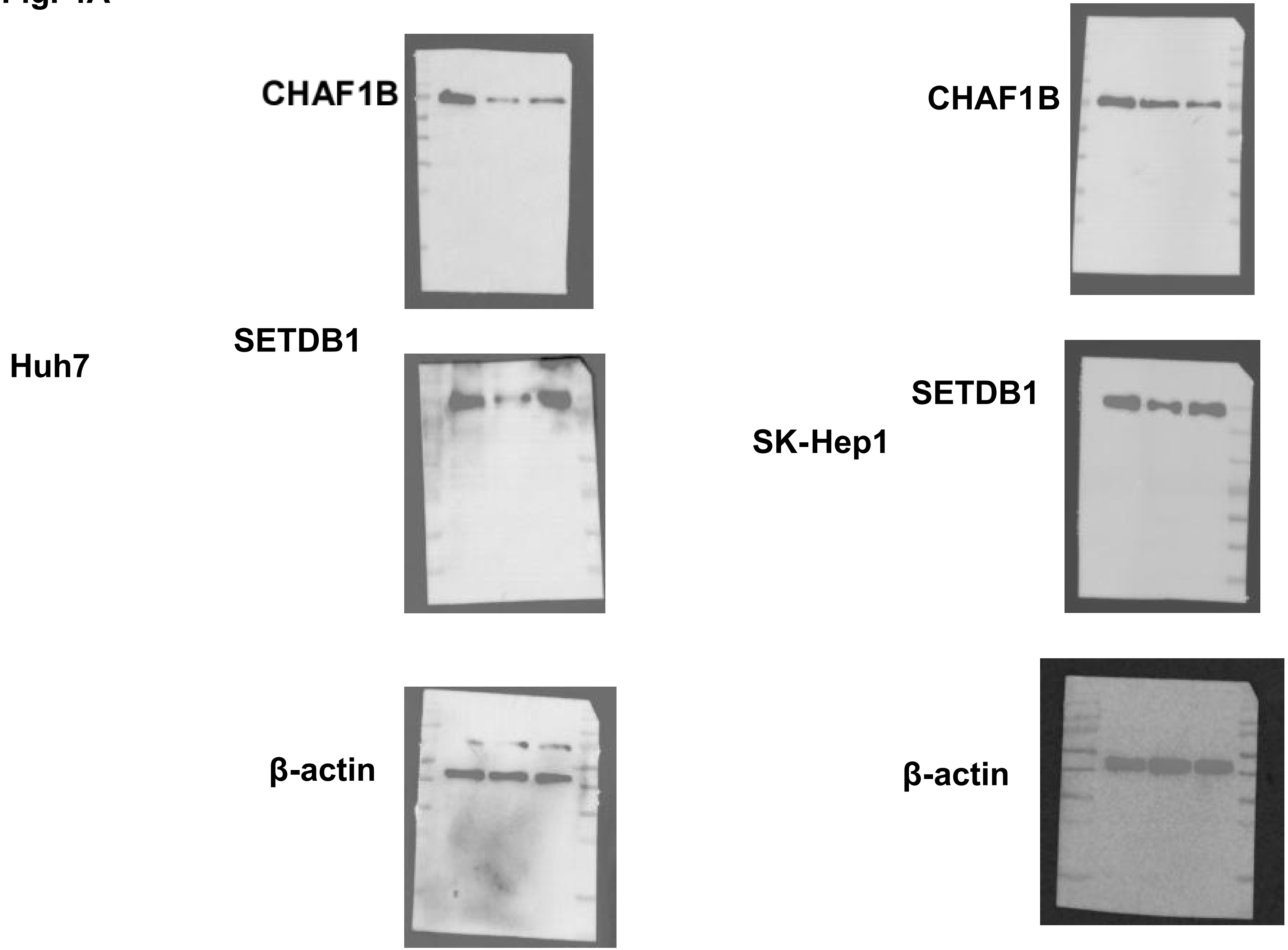

Fig. 4A

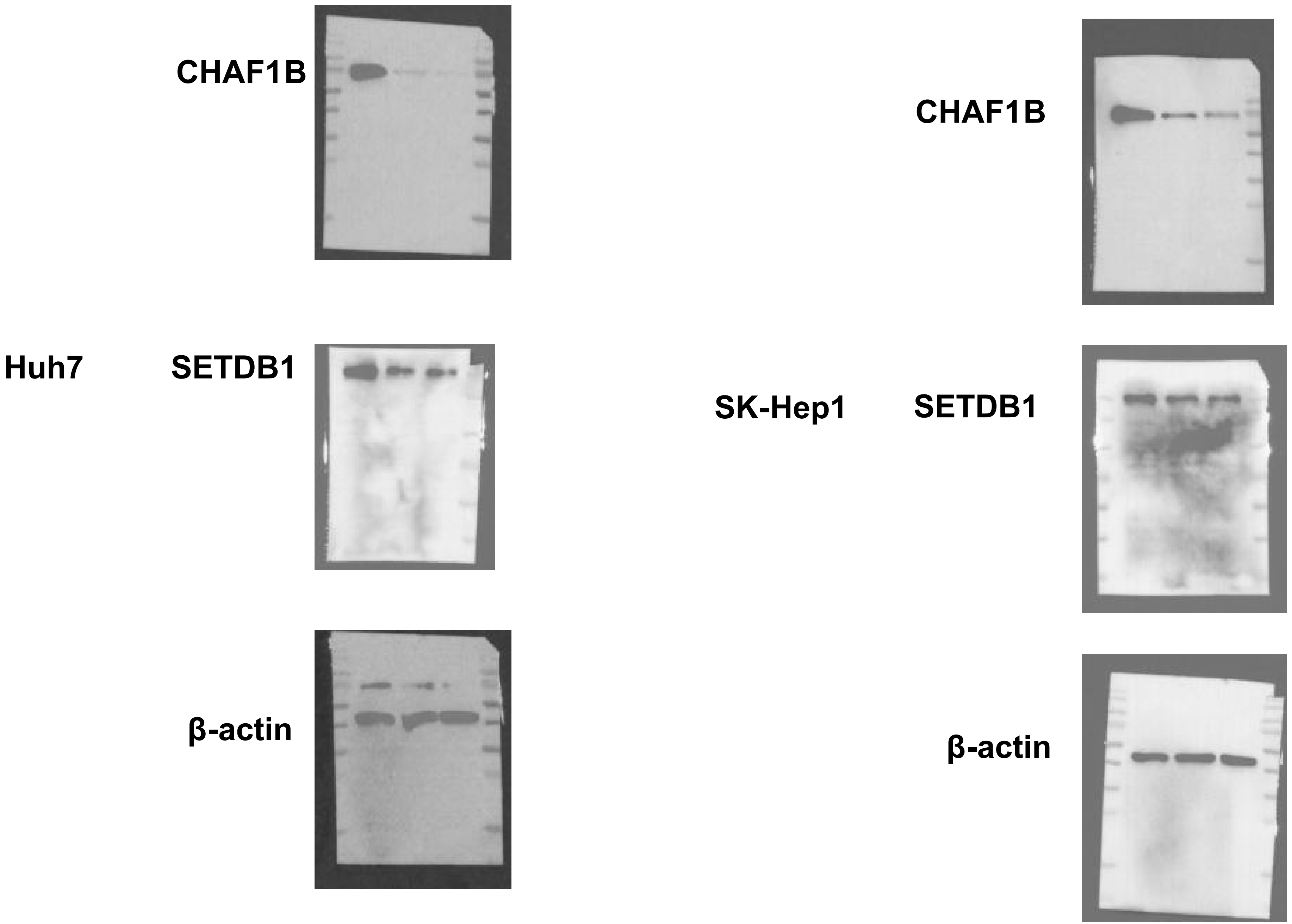

Fig. 4B

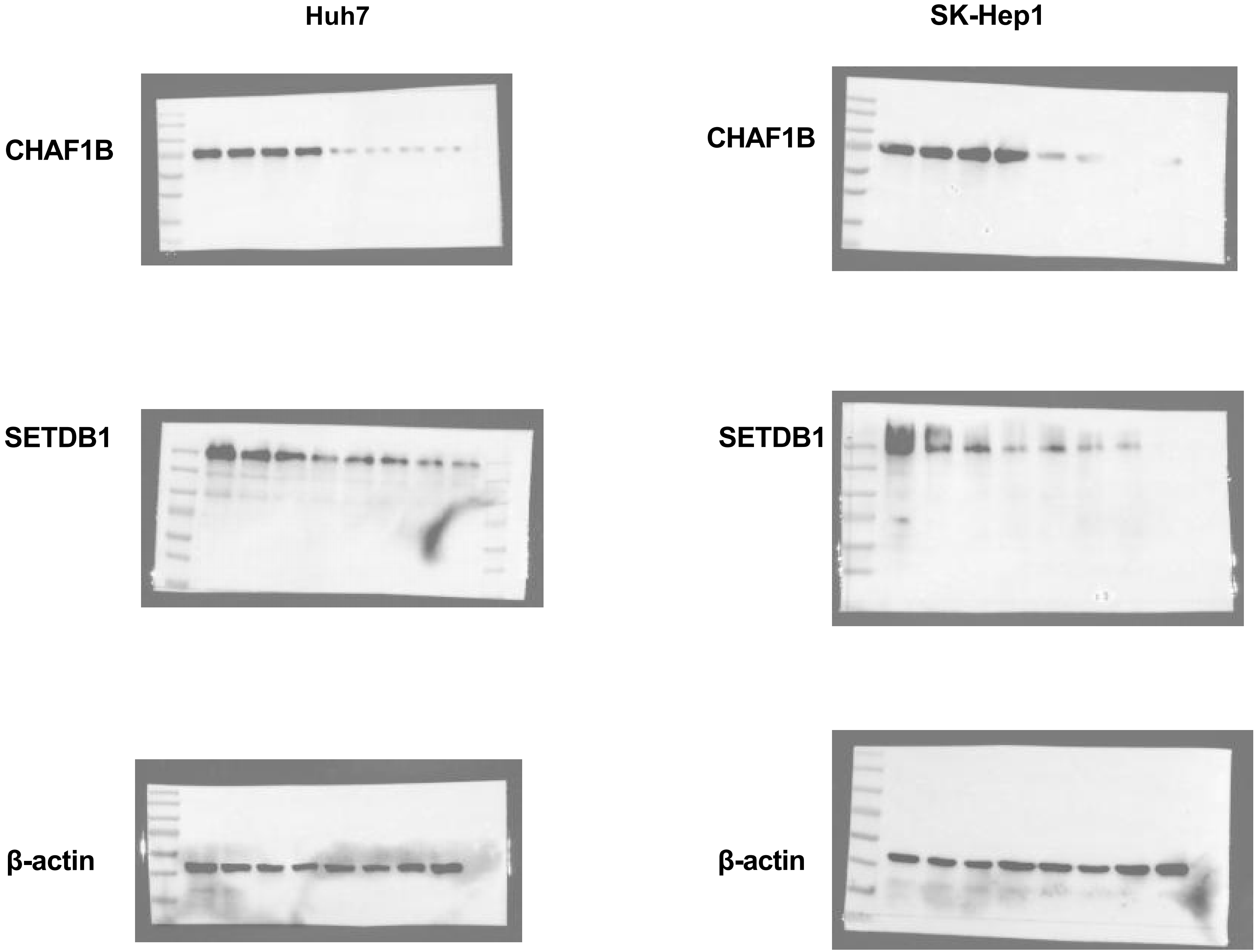

Fig. 4C

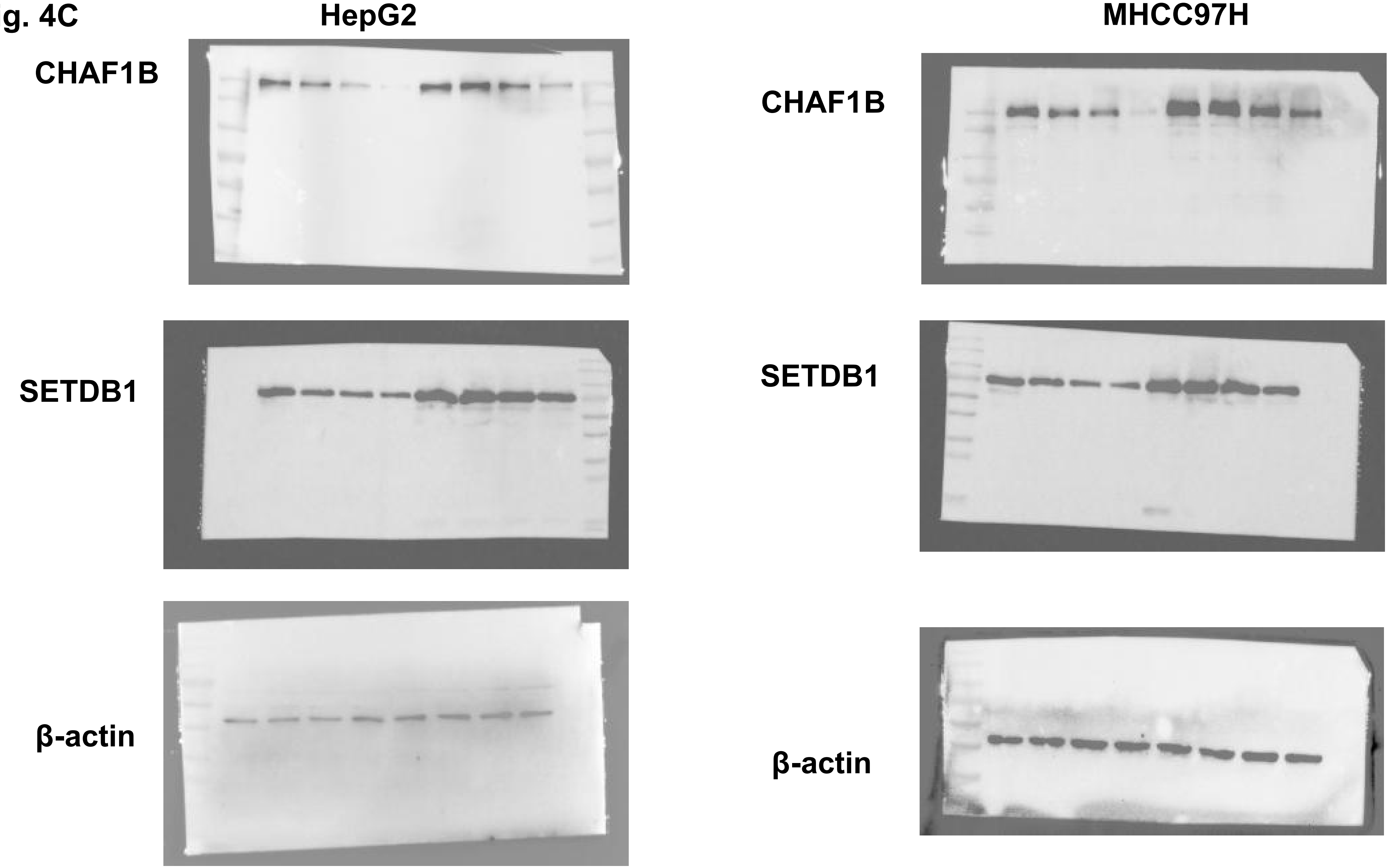

Fig. 4D HEK293T

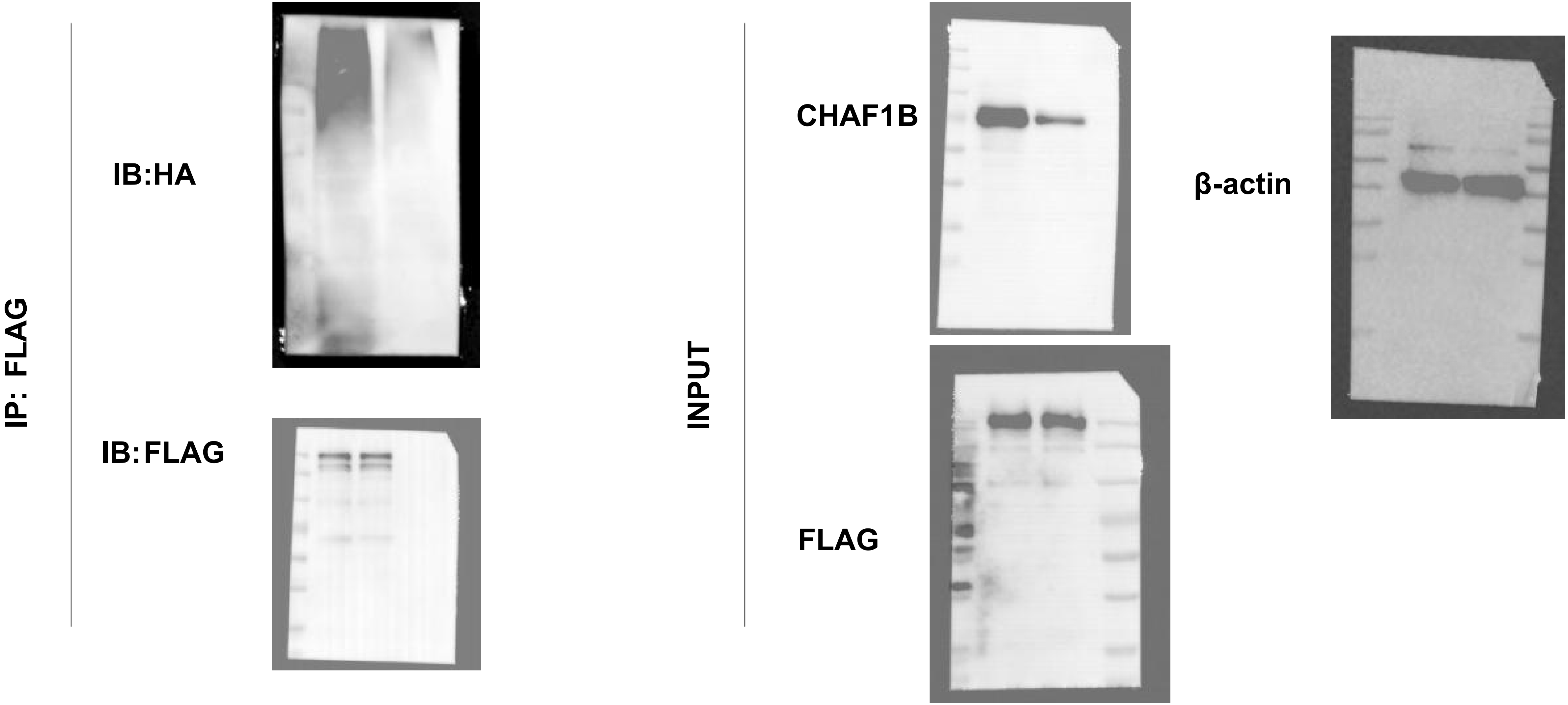

Fig. 4D

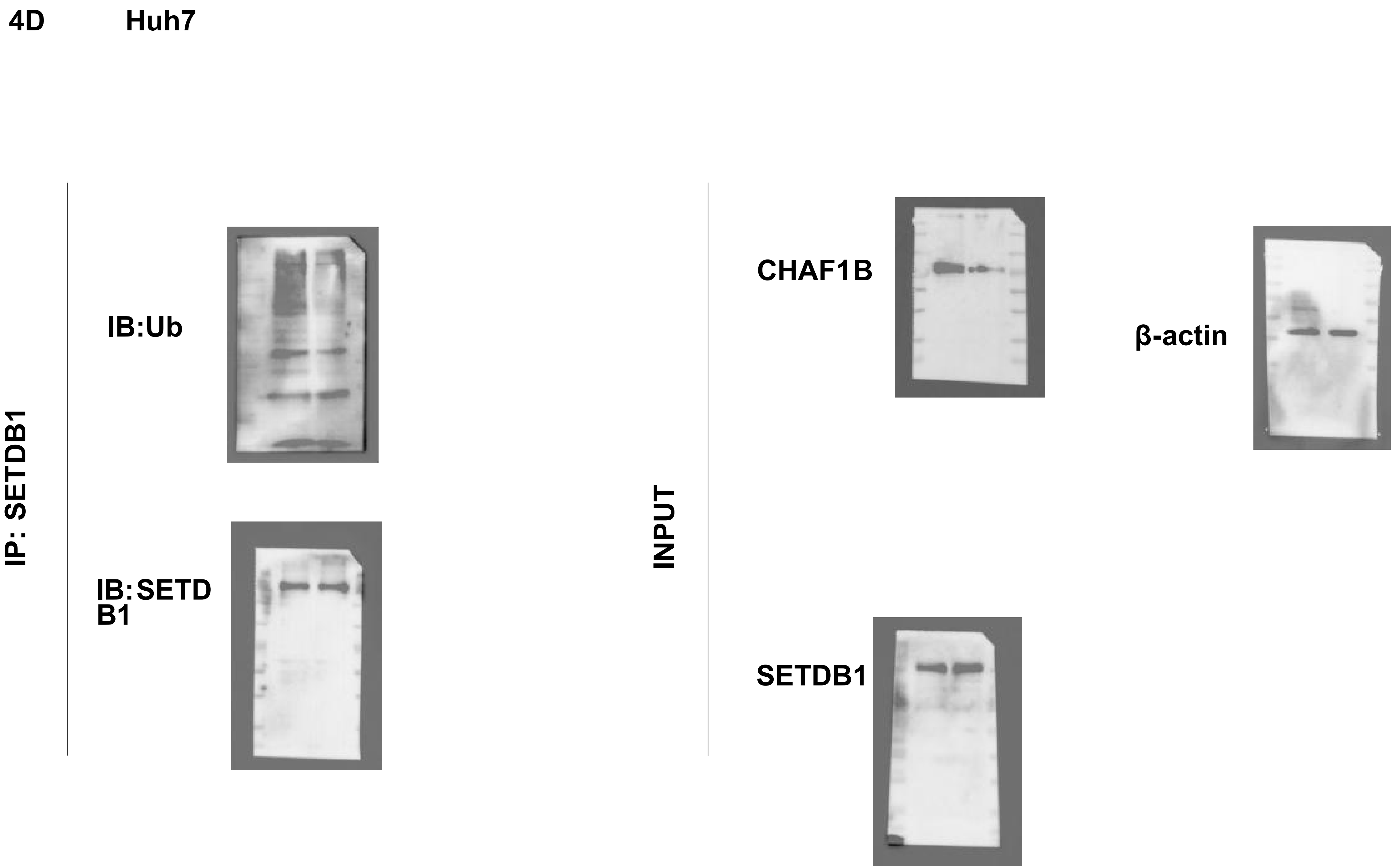

Fig. 4D

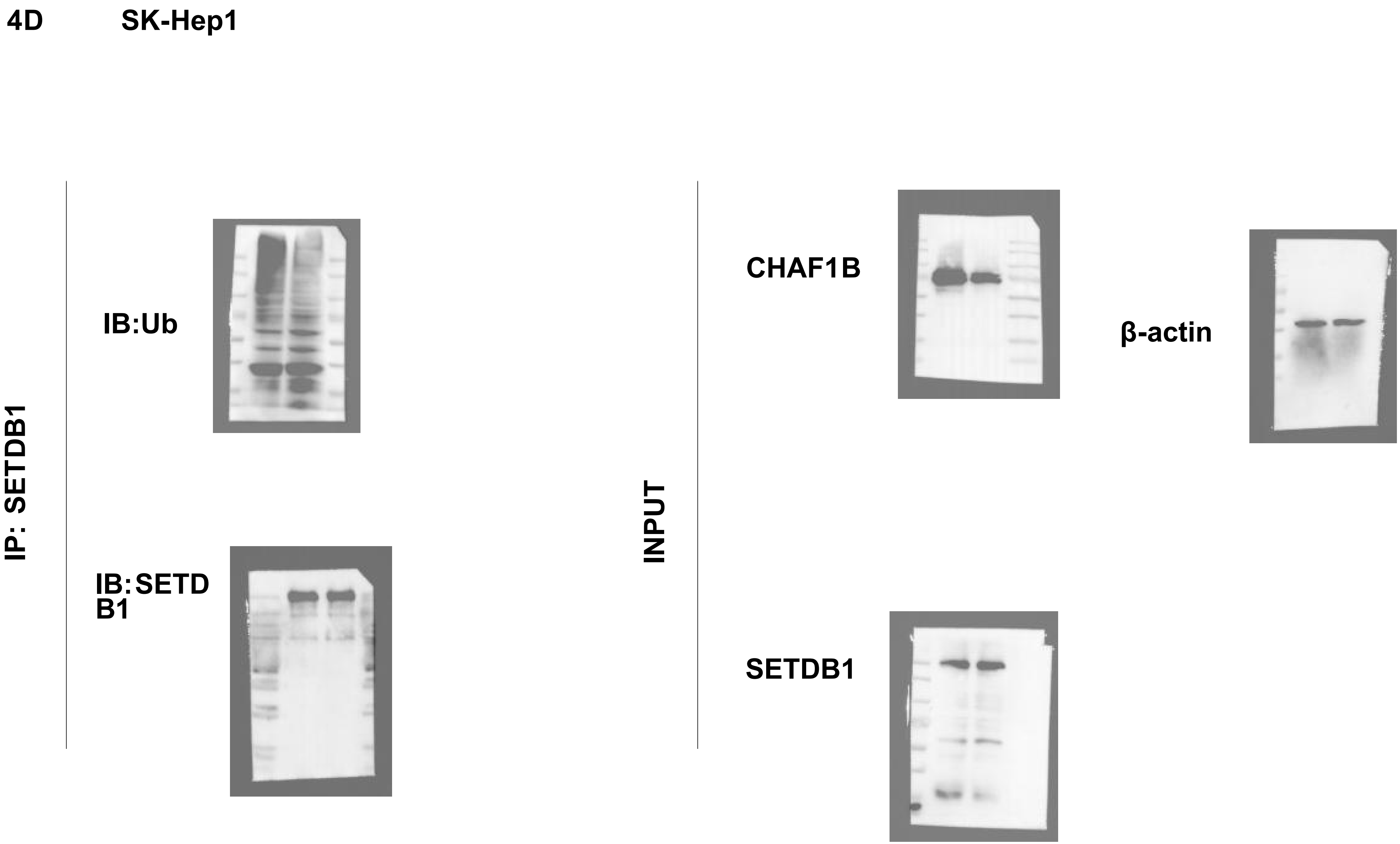

Fig. 4E **HEK293T**

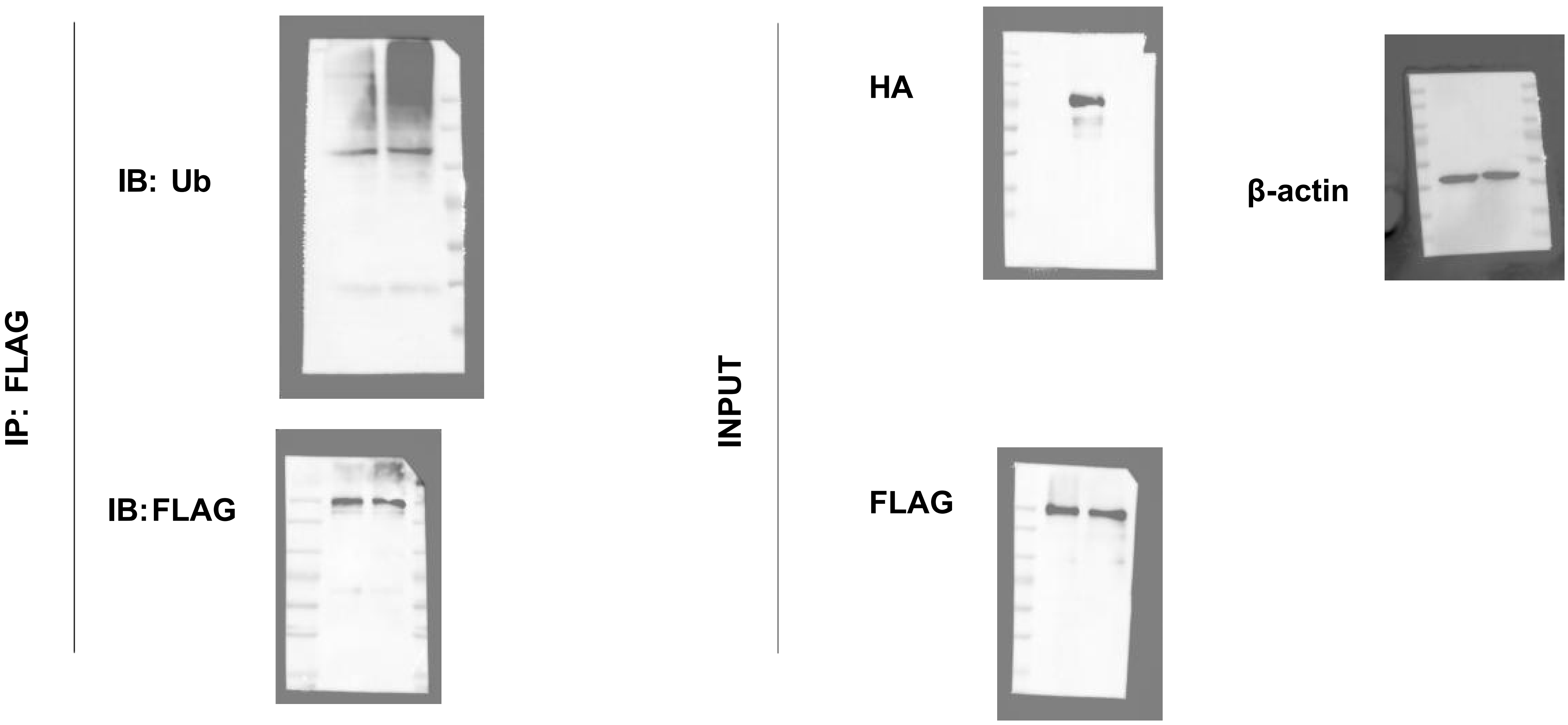

Fig. 4E HepG2

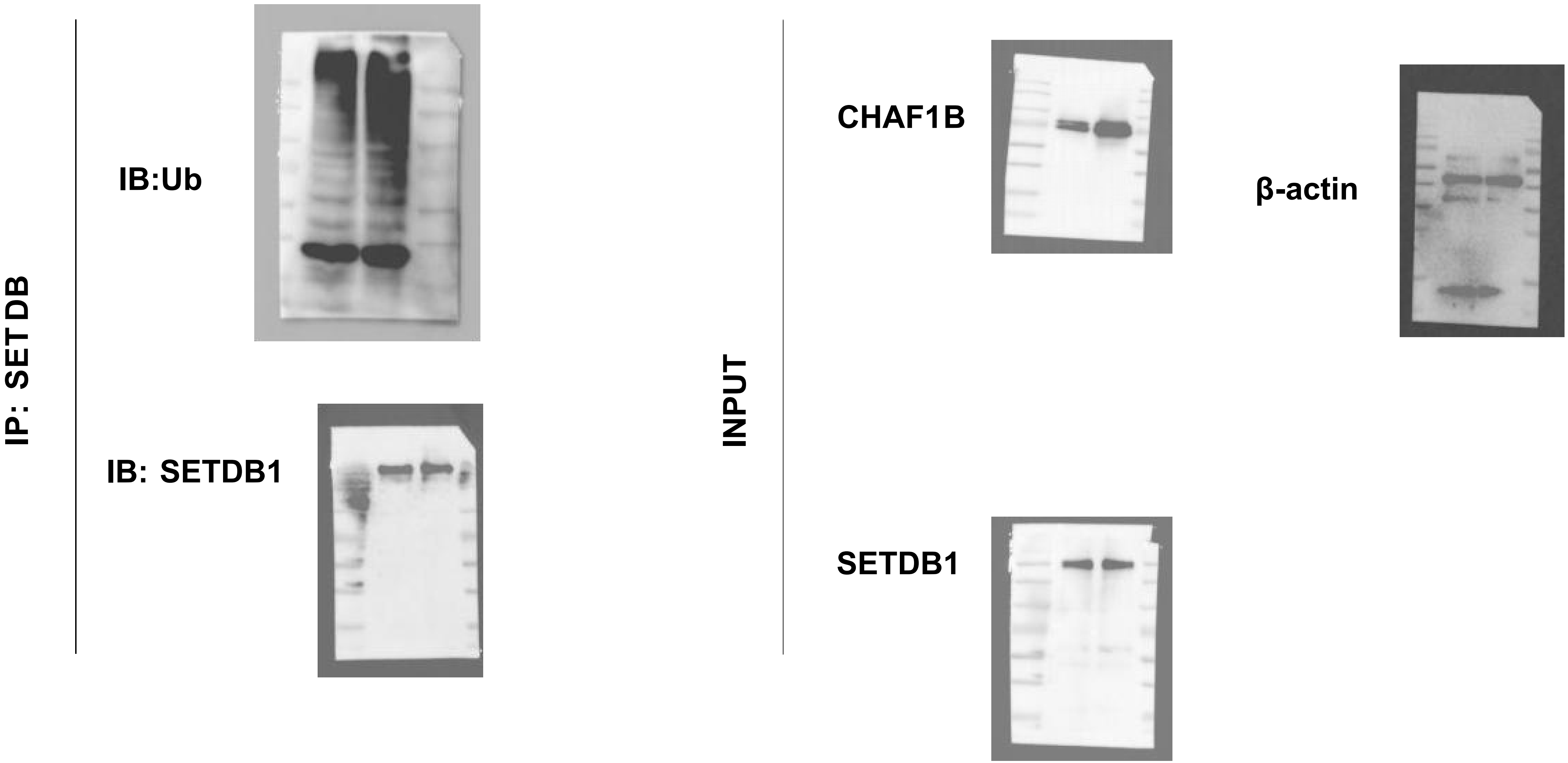

Fig. 4E

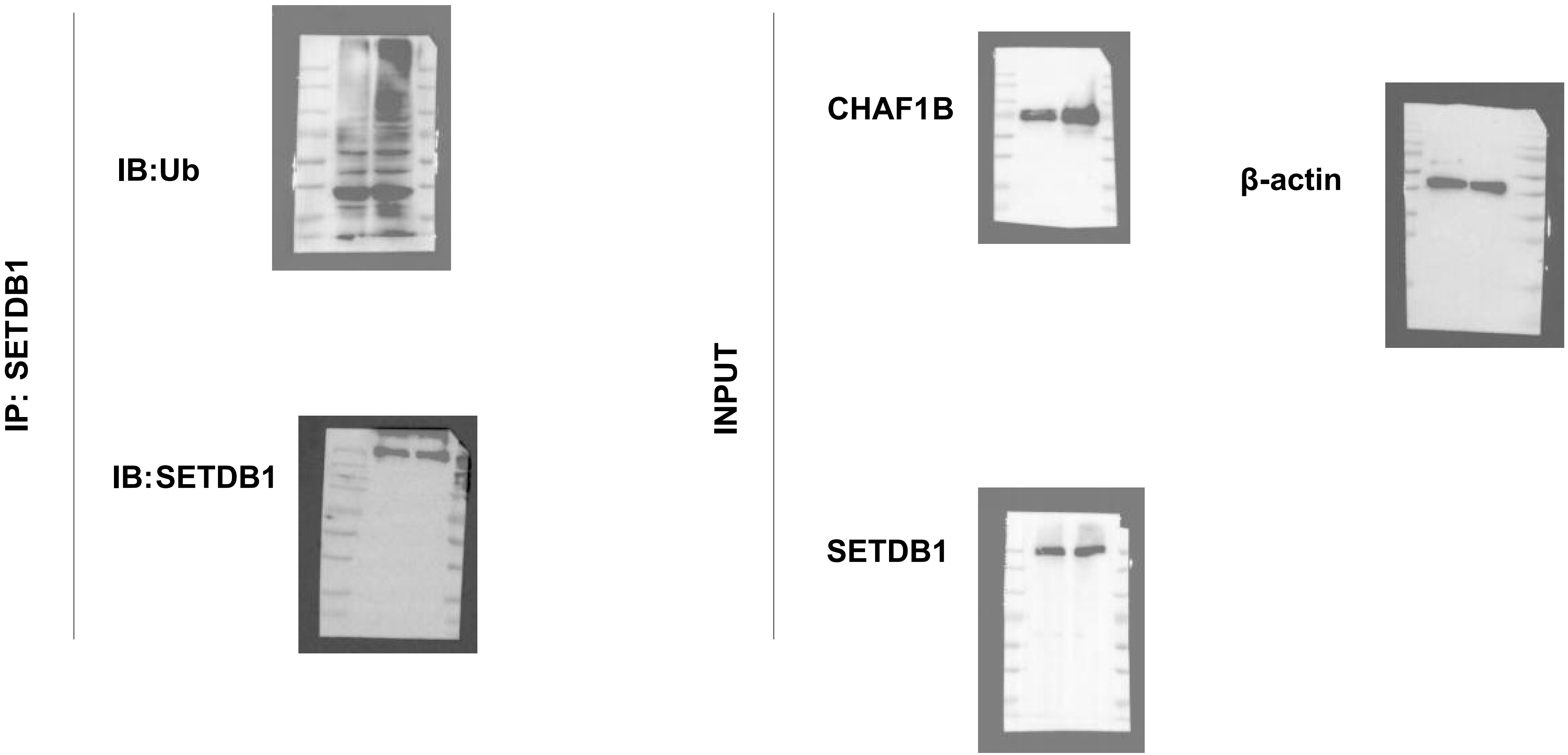
MHCC97H
IP: SETDB1
IB:Ub
IB:SETDB1
INPUT
CHAF1B
β-actin
SETDB1

Fig. 4F

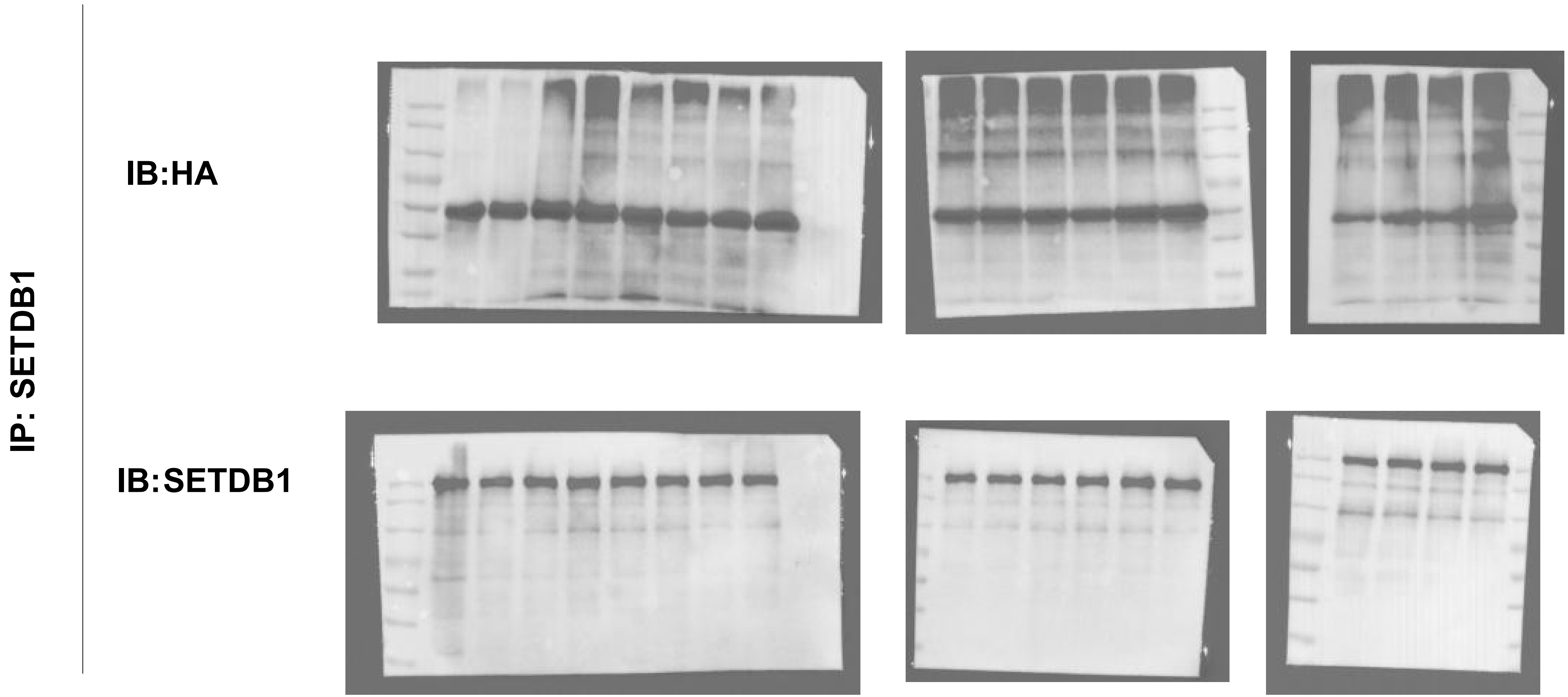

Fig. 4F

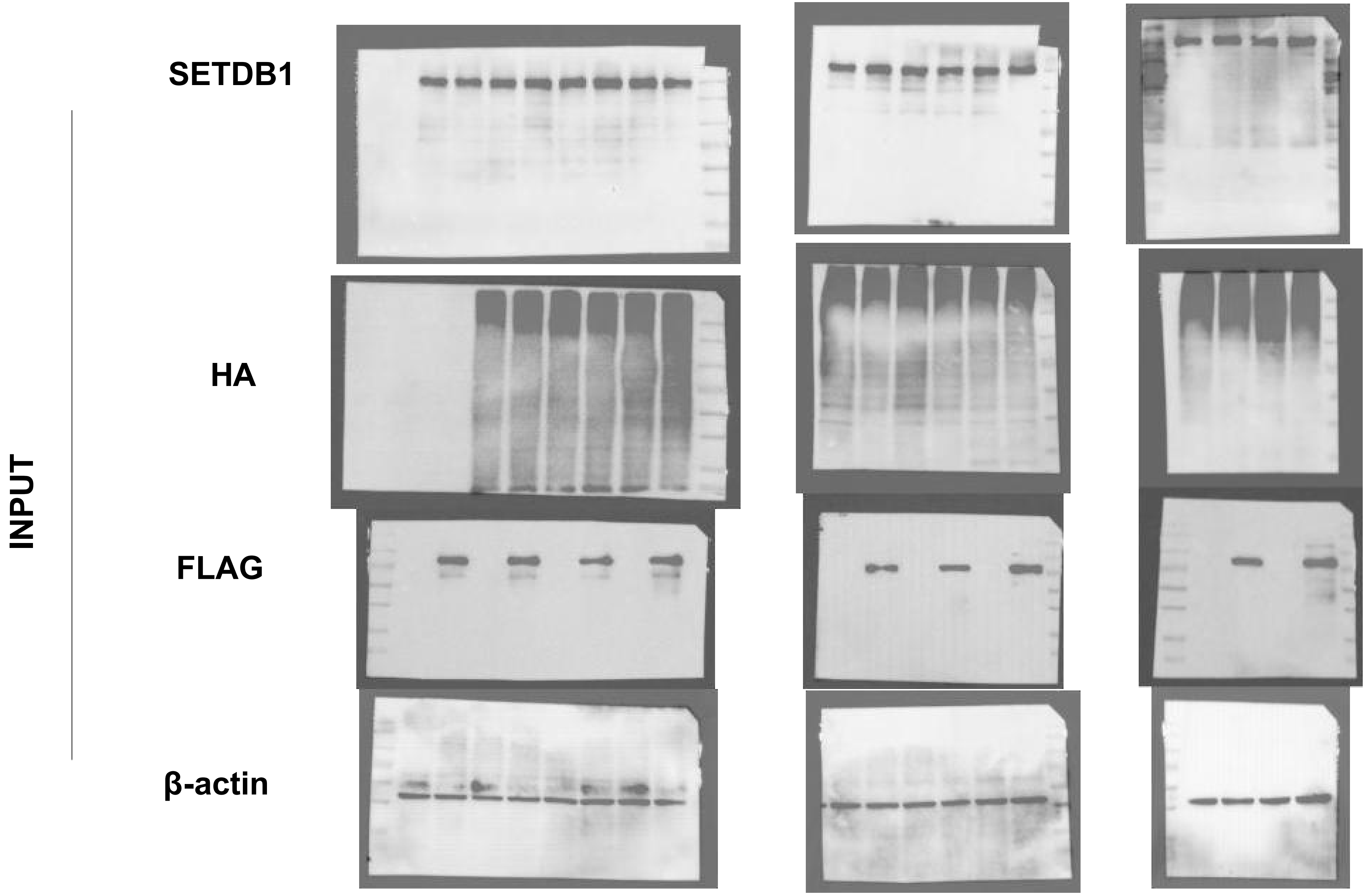

Fig. 4G

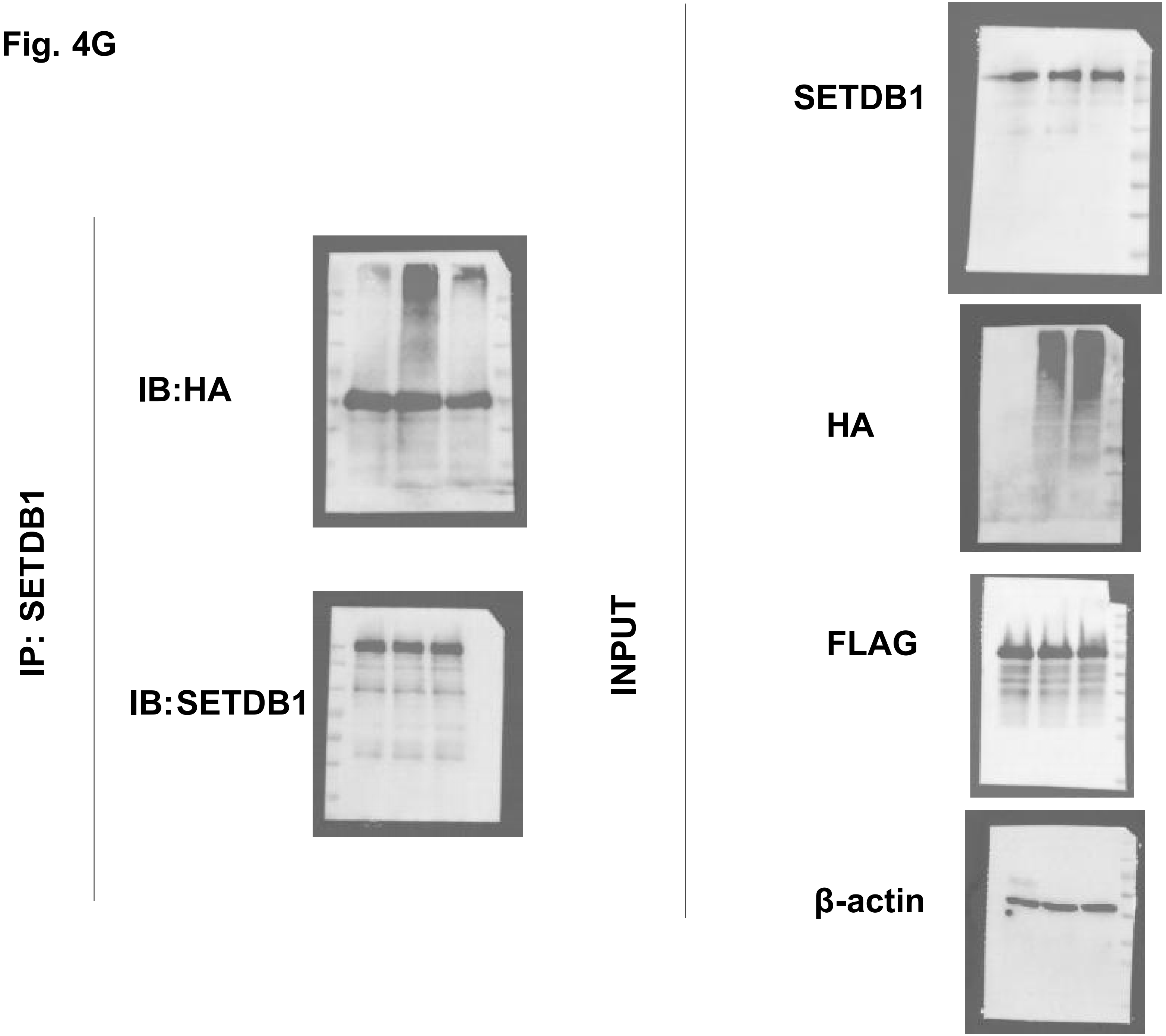

Fig. 4J

HepG2

CHAF1B

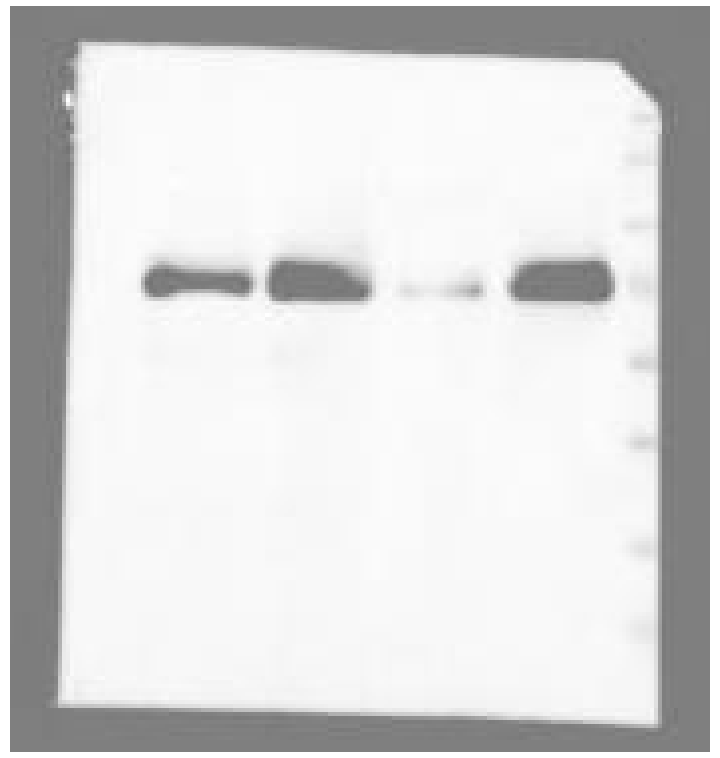

α-tubulin

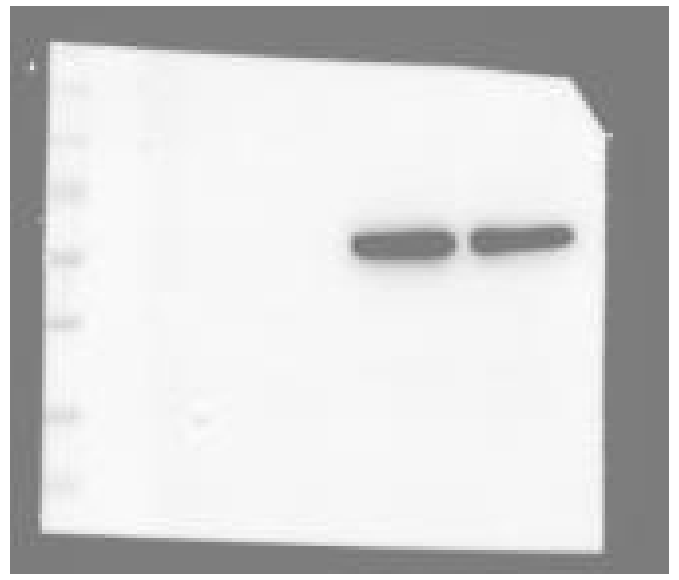

SETDB1

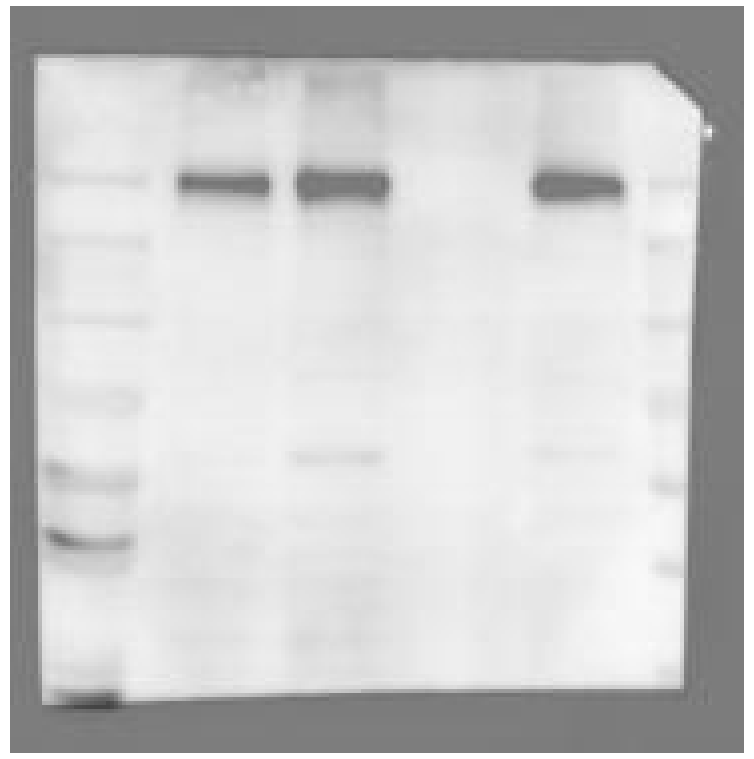

Laminb1

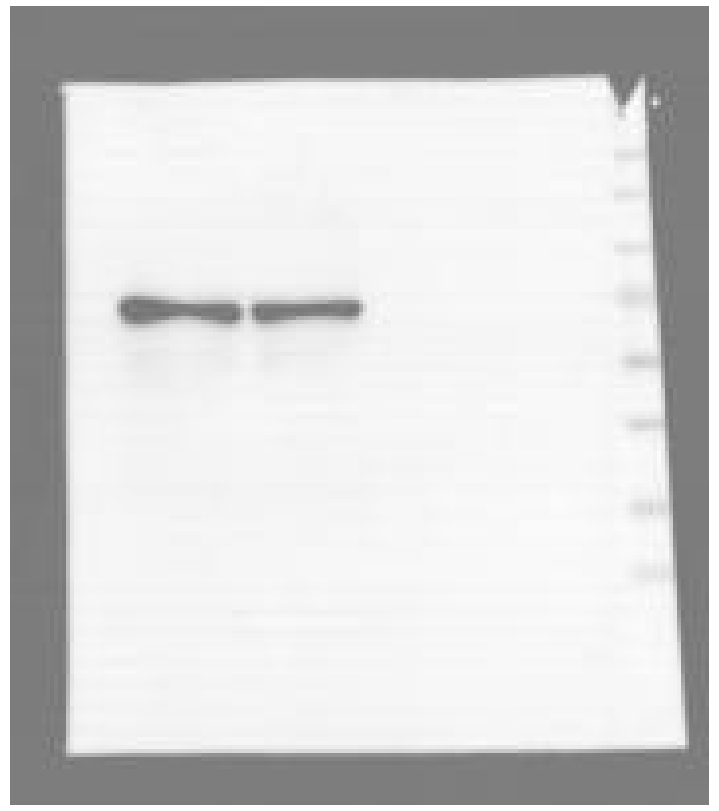

Fig. 4J

MHCC97H

CHAF1B

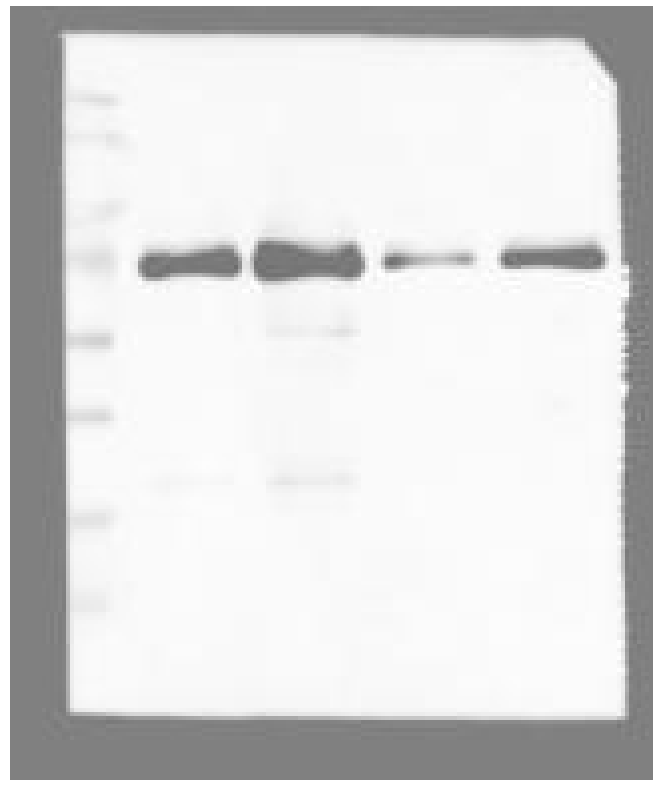

α-tubulin

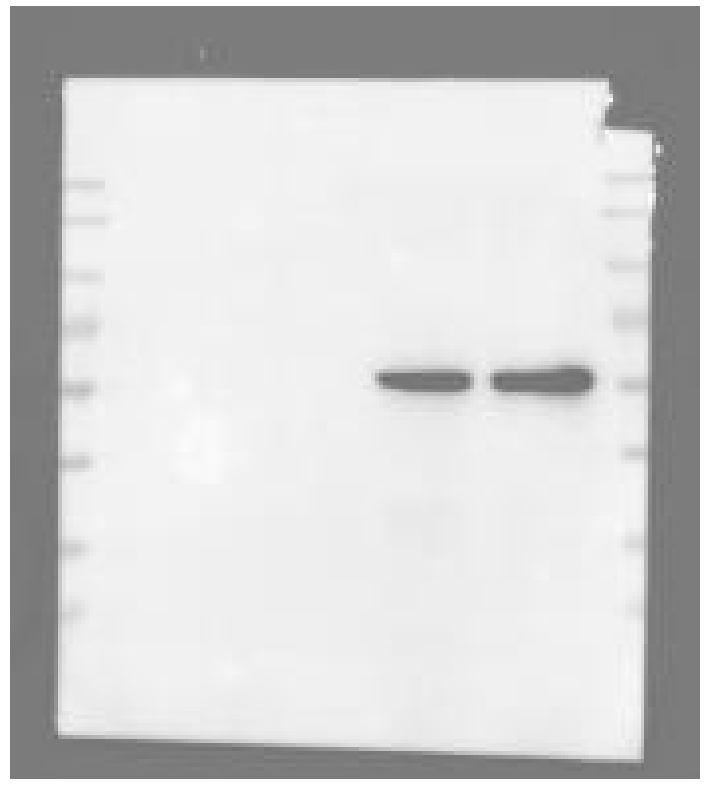

SETDB1

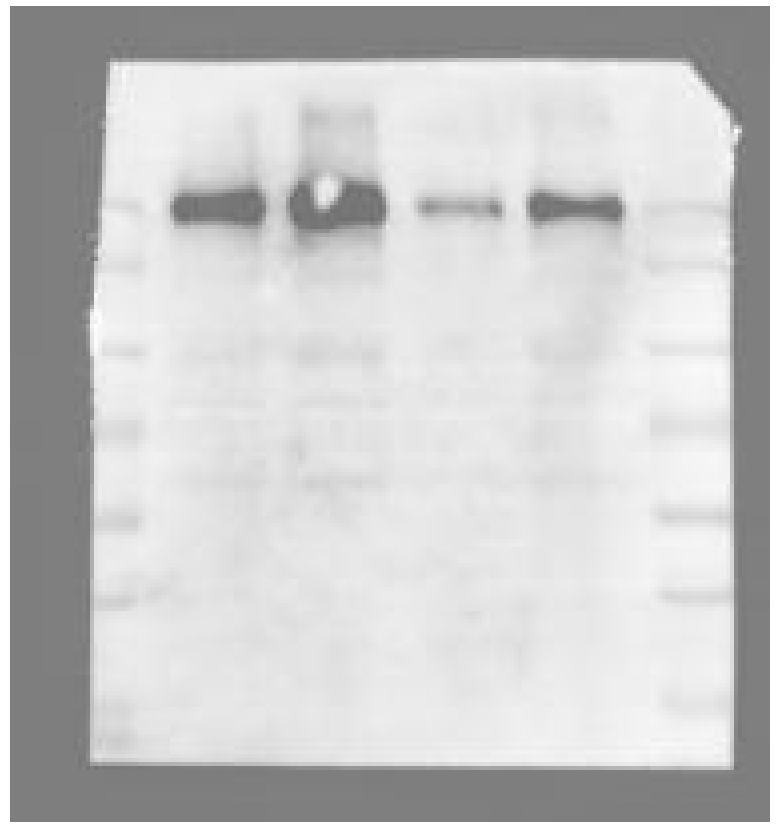

Laminb1

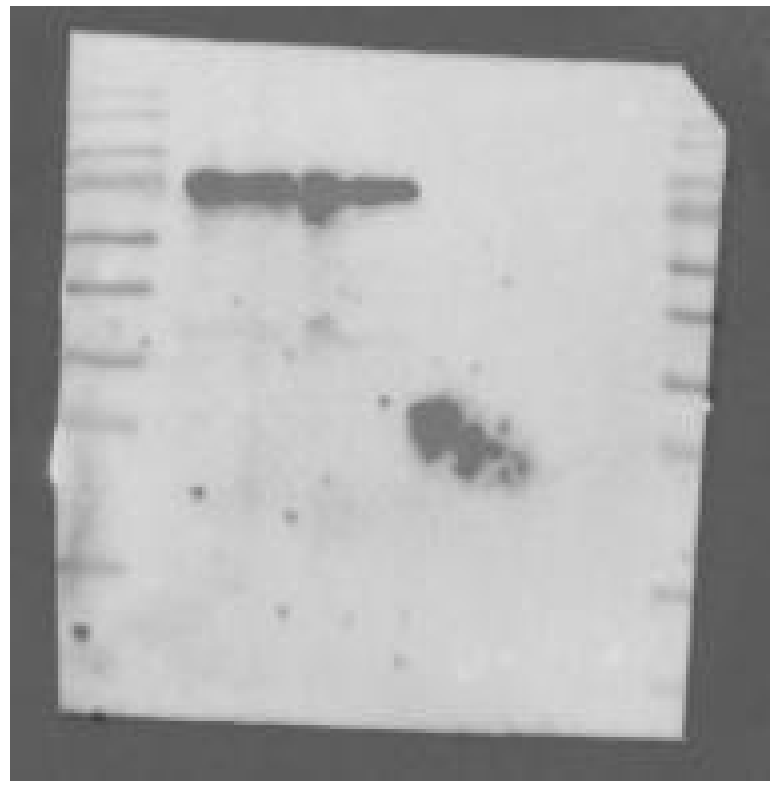

Fig. 4J

HEK293T

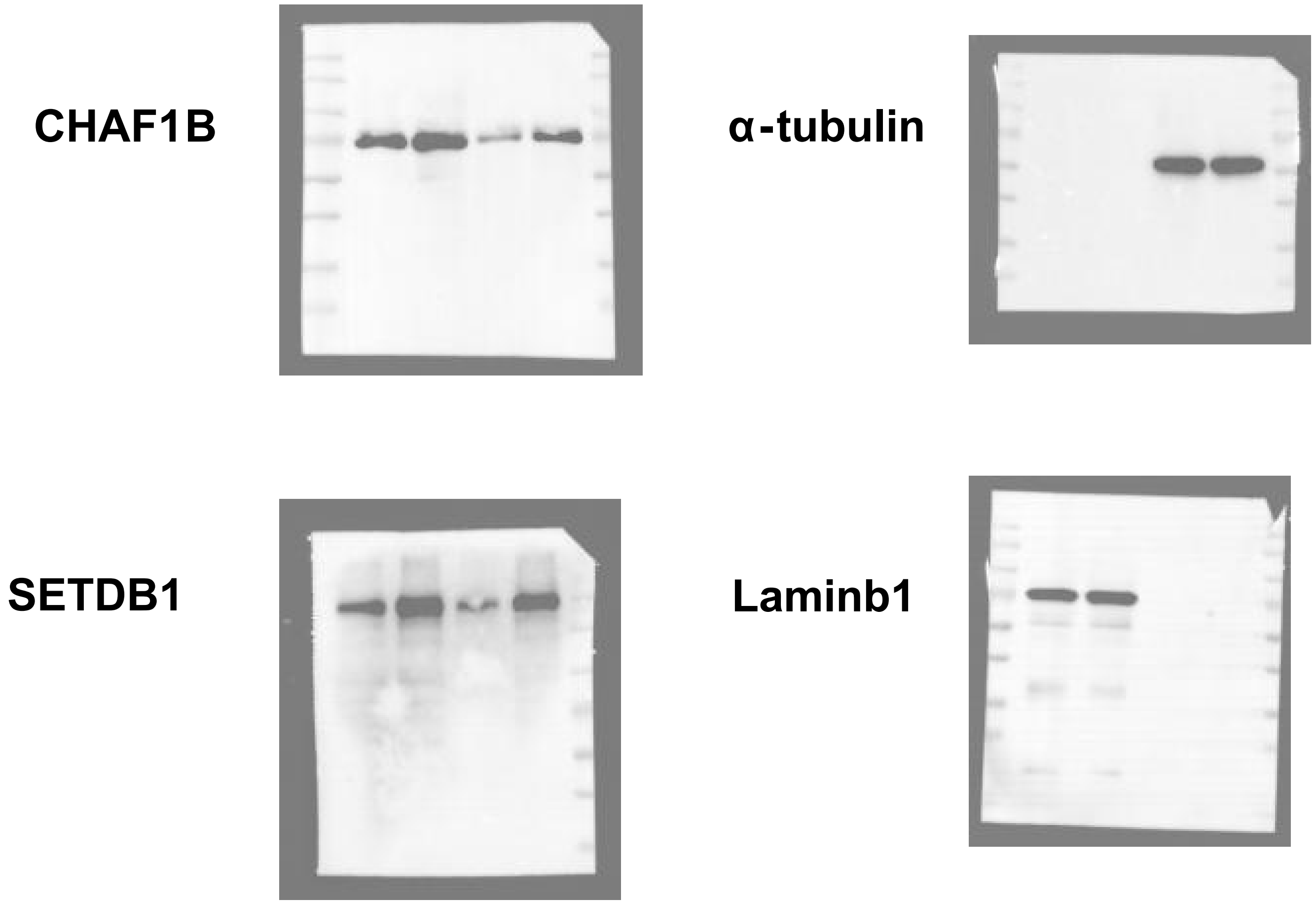

Fig. 4J

HepG2

CHAF1B

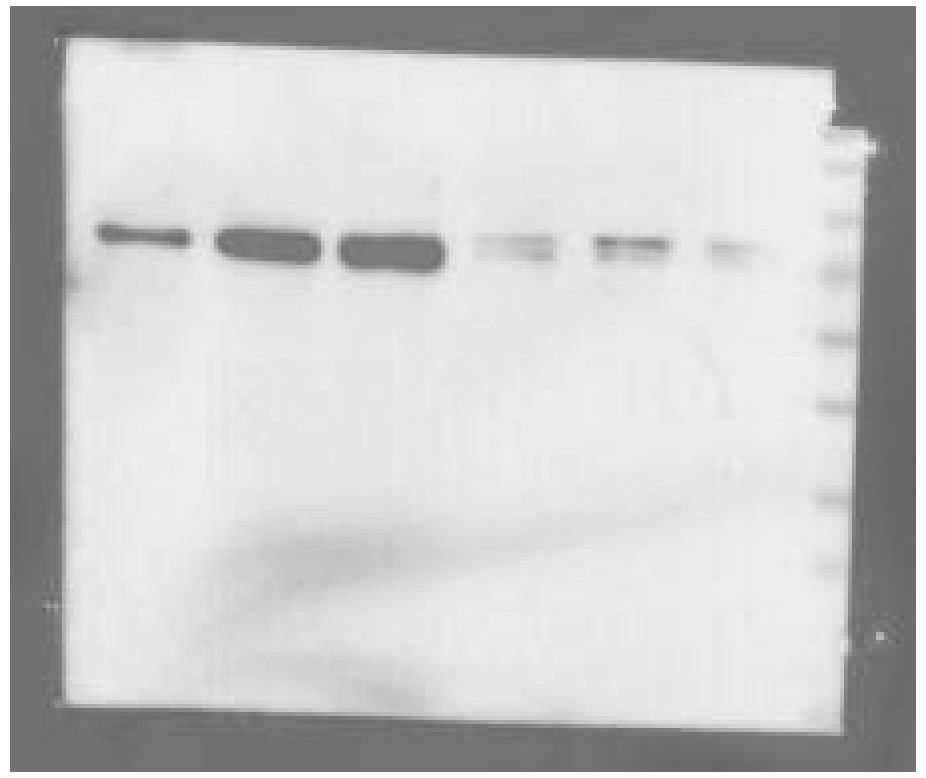

α-tubulin

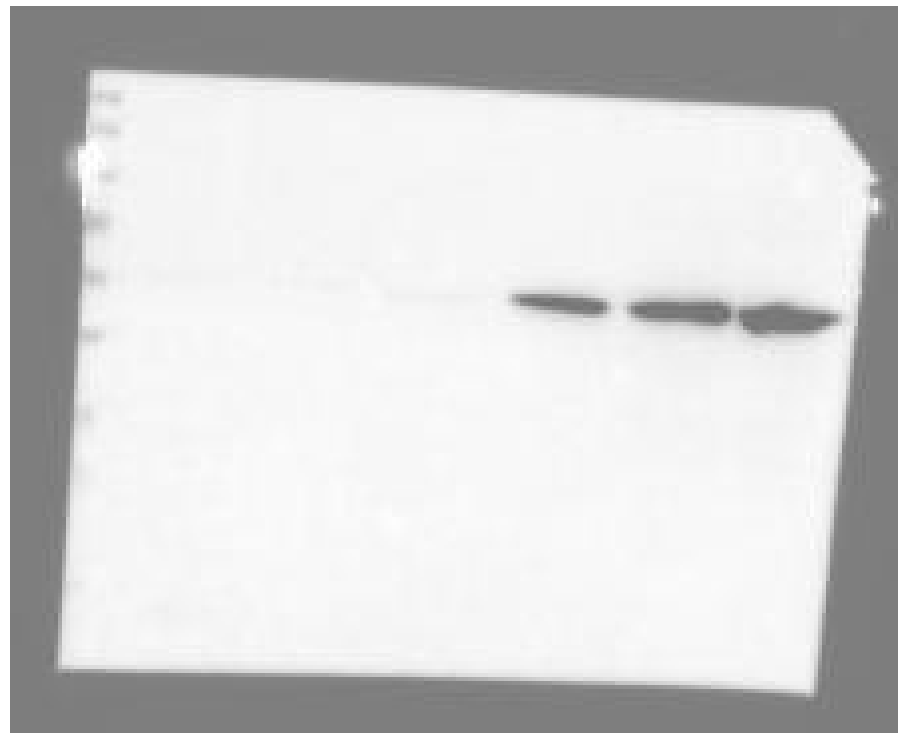

SETDB1

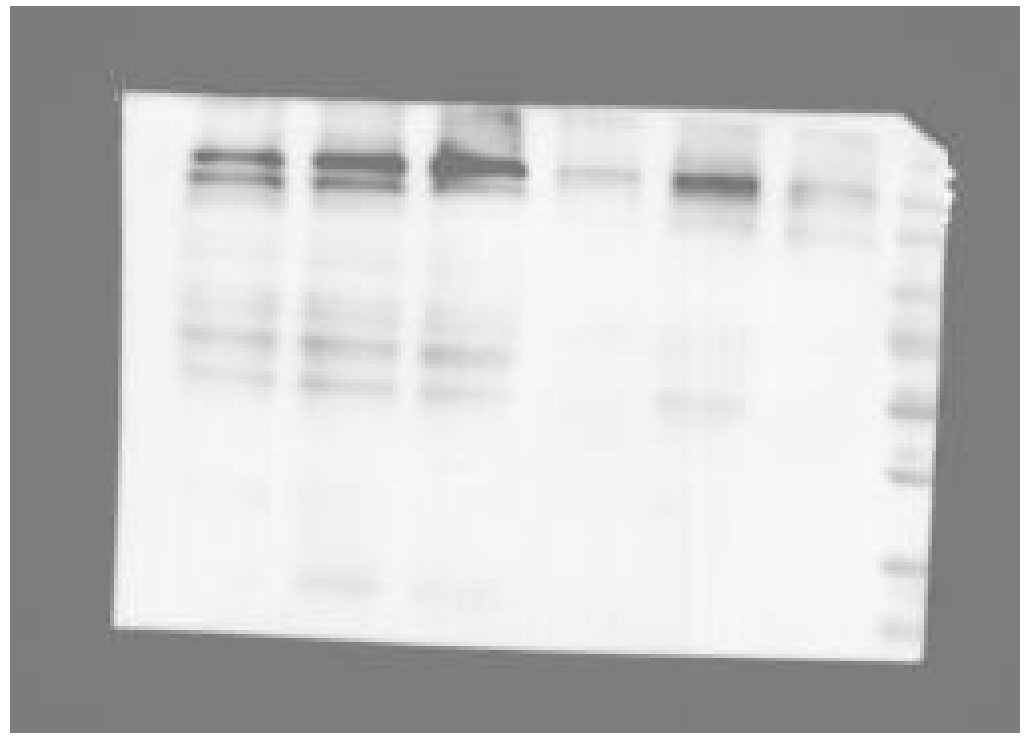

Laminb1

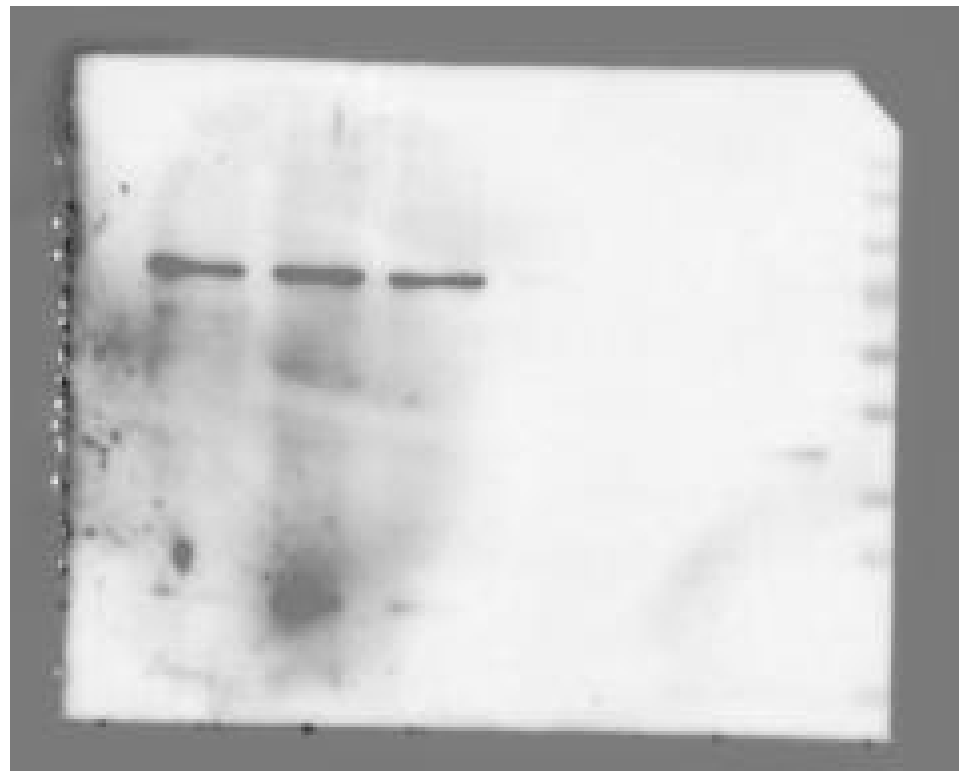

Fig. 4J

MHCC97H

CHAF1B

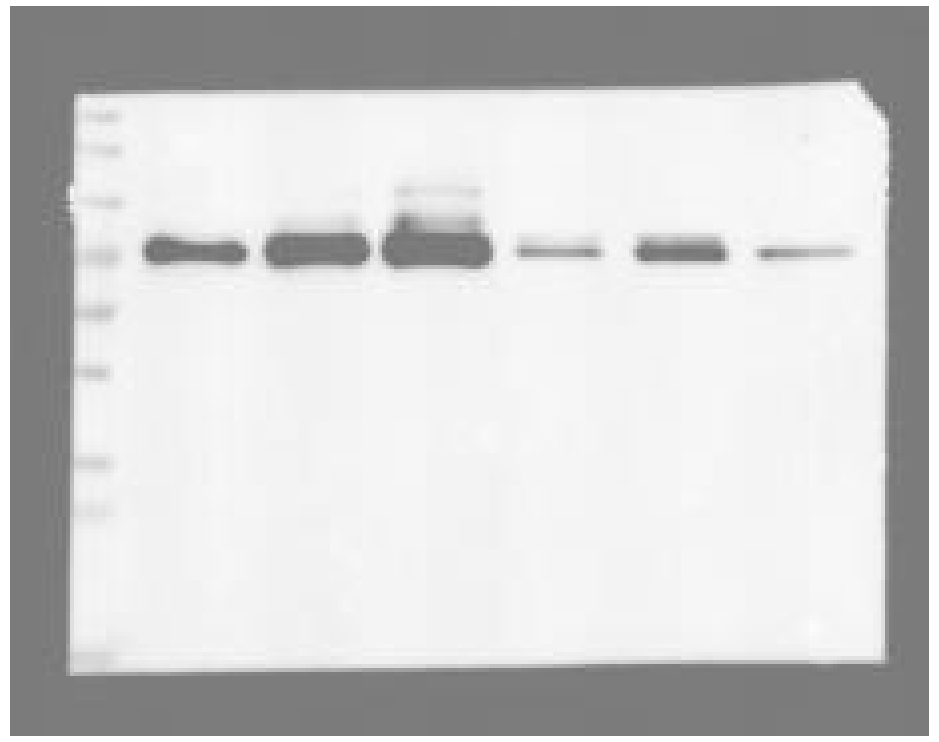

α-tubulin

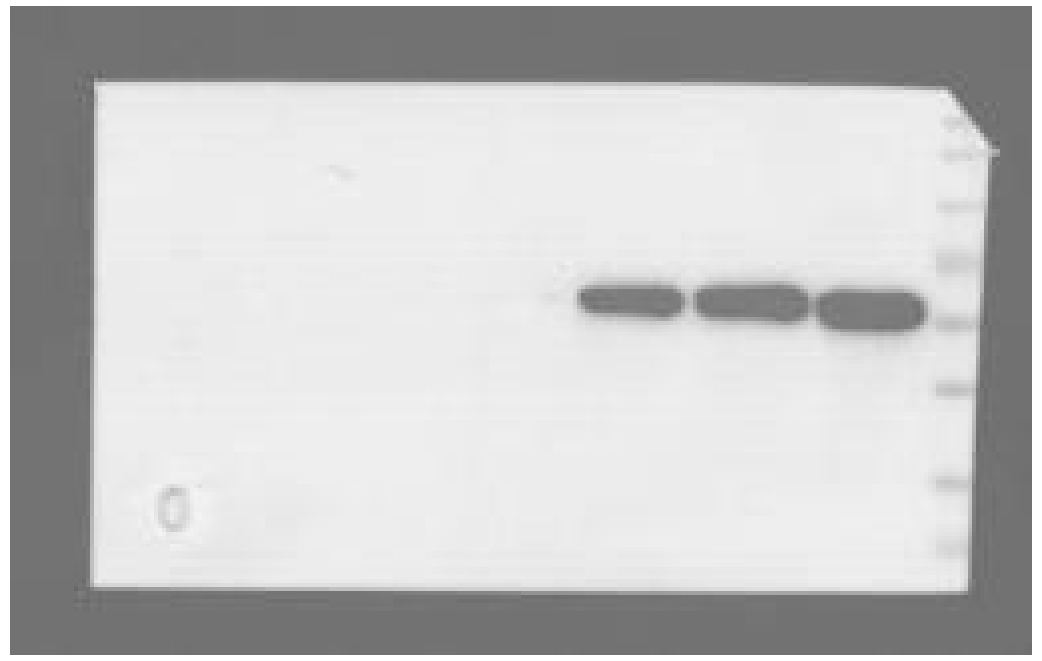

SETDB1

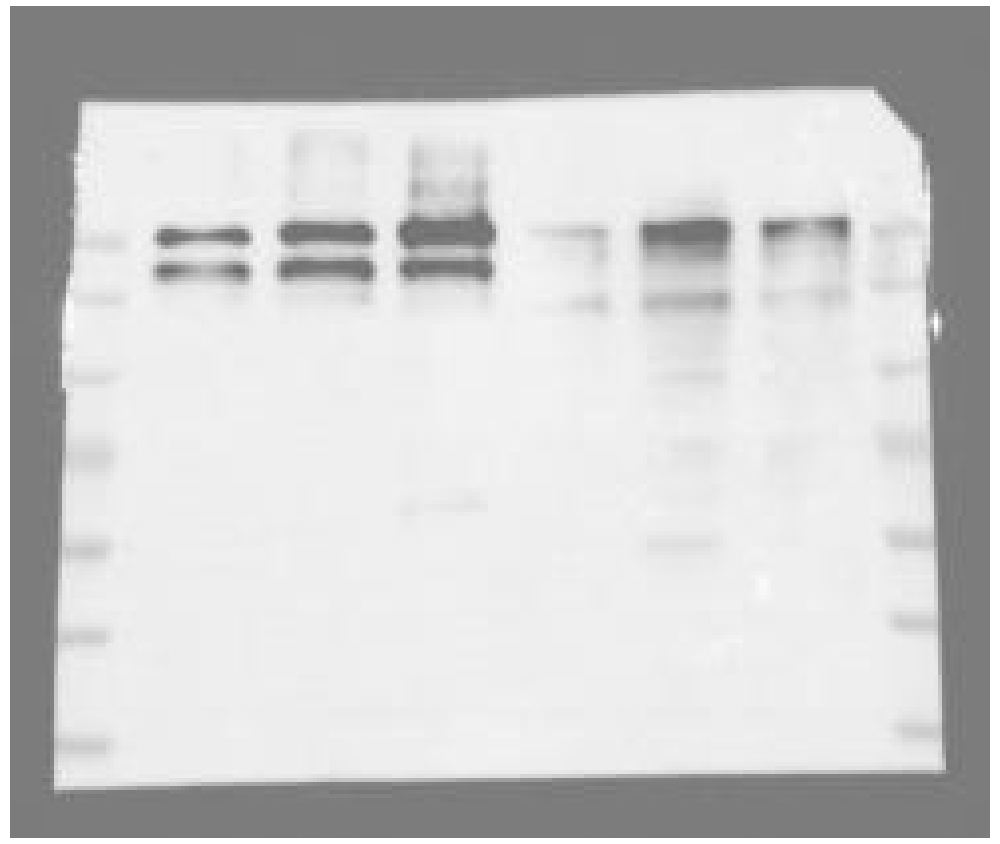

Laminb1

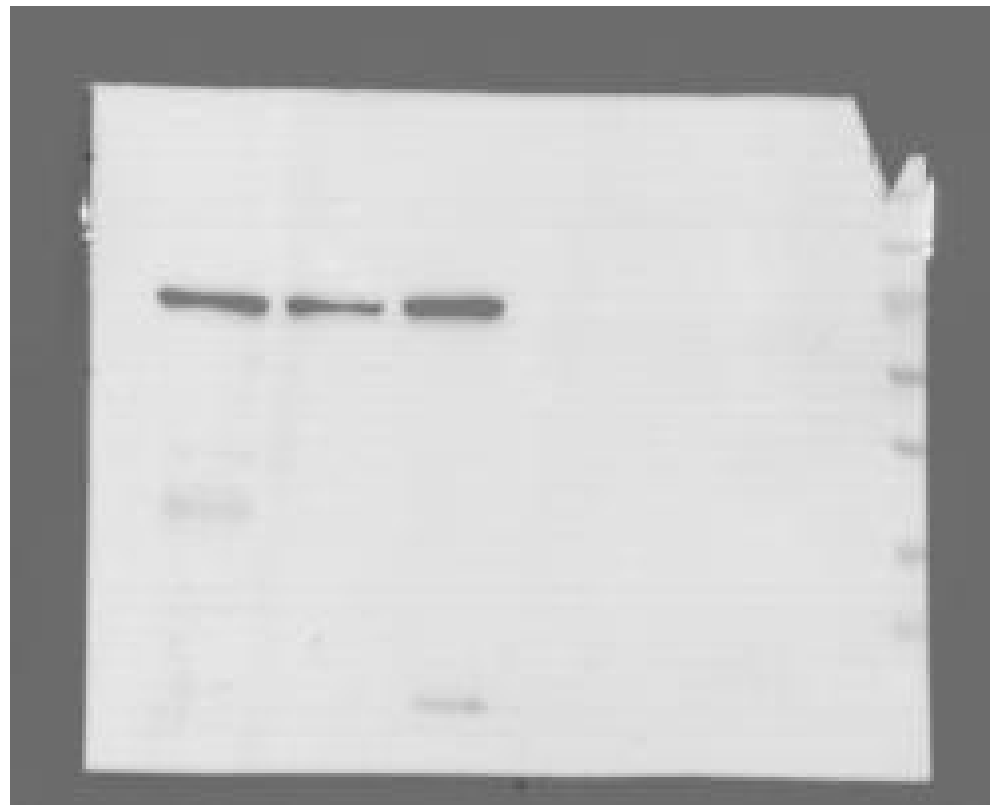

Fig. 4J

HEK293T

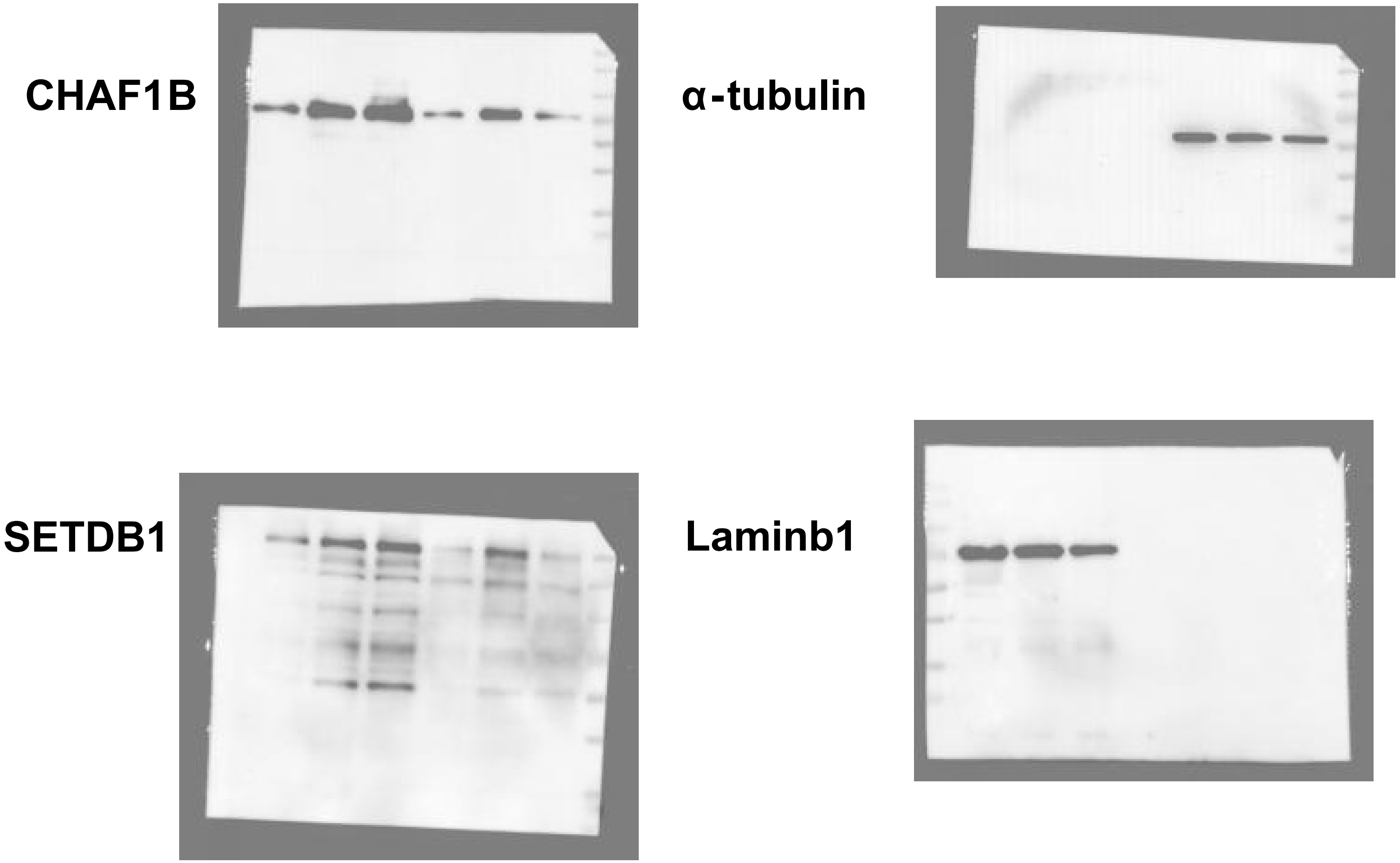

Fig. 4K

HepG2

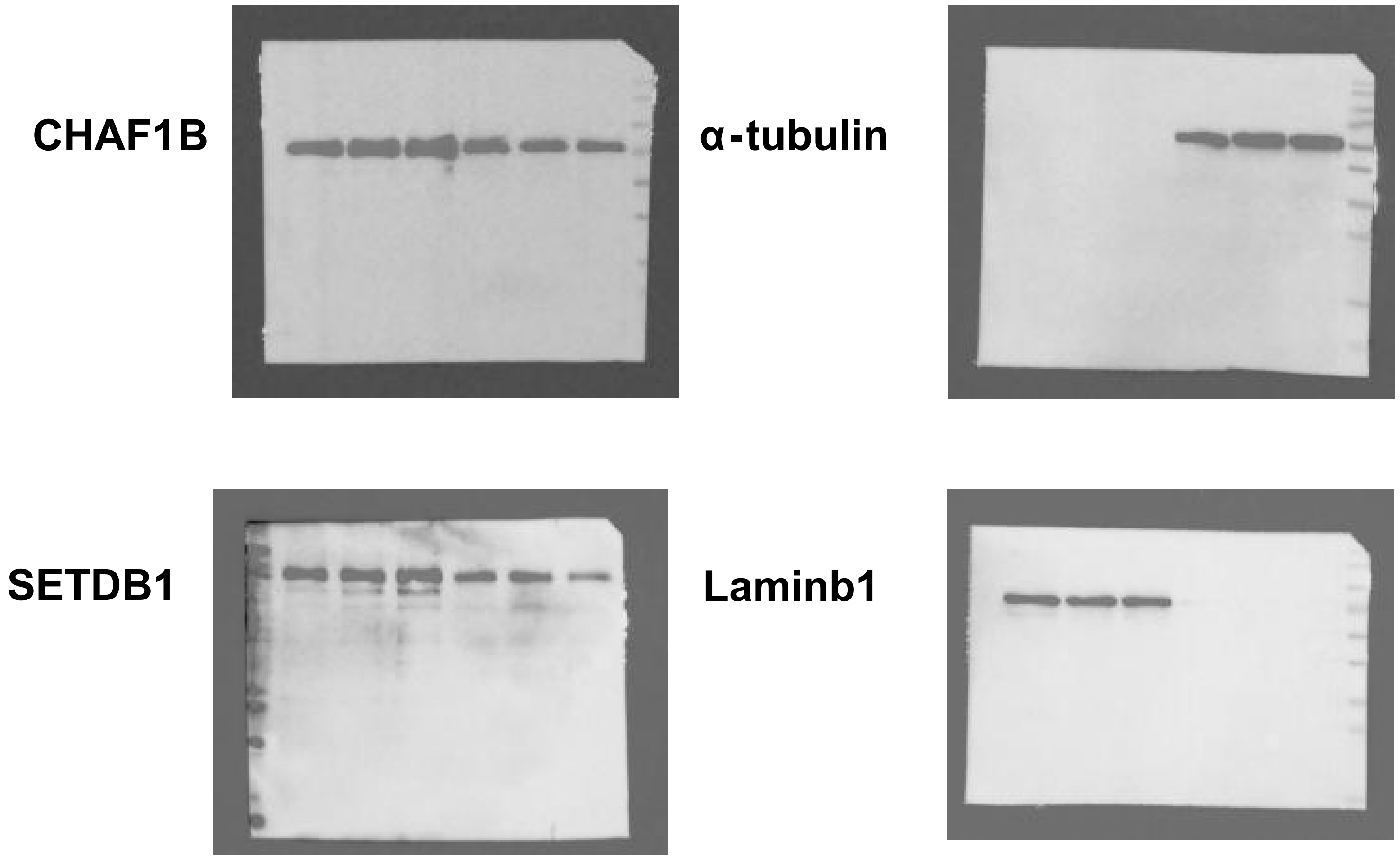

Fig. 4K

MHCC97H

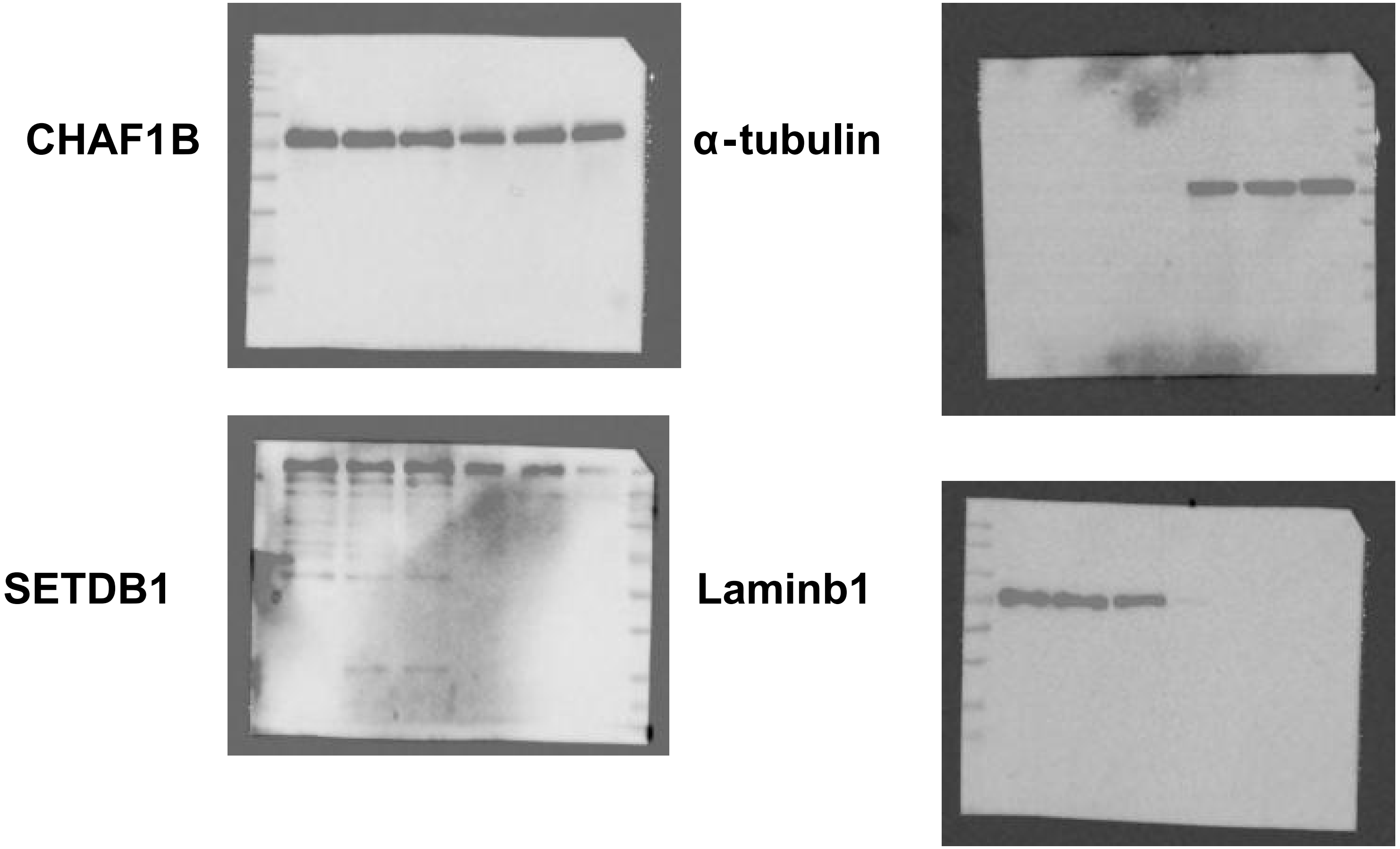

Fig. 4L

HepG2

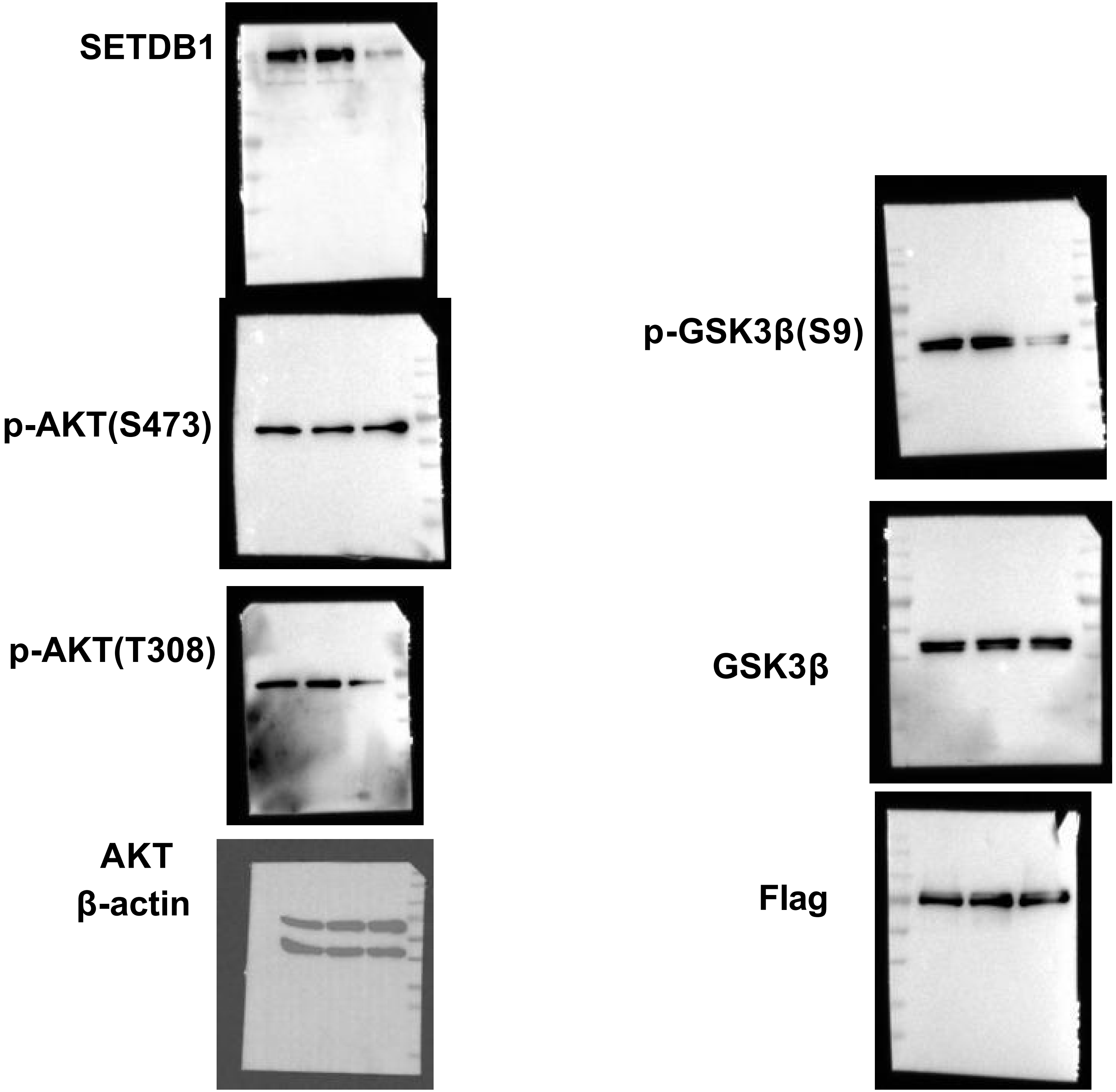

Fig. 4L

MHCC97H

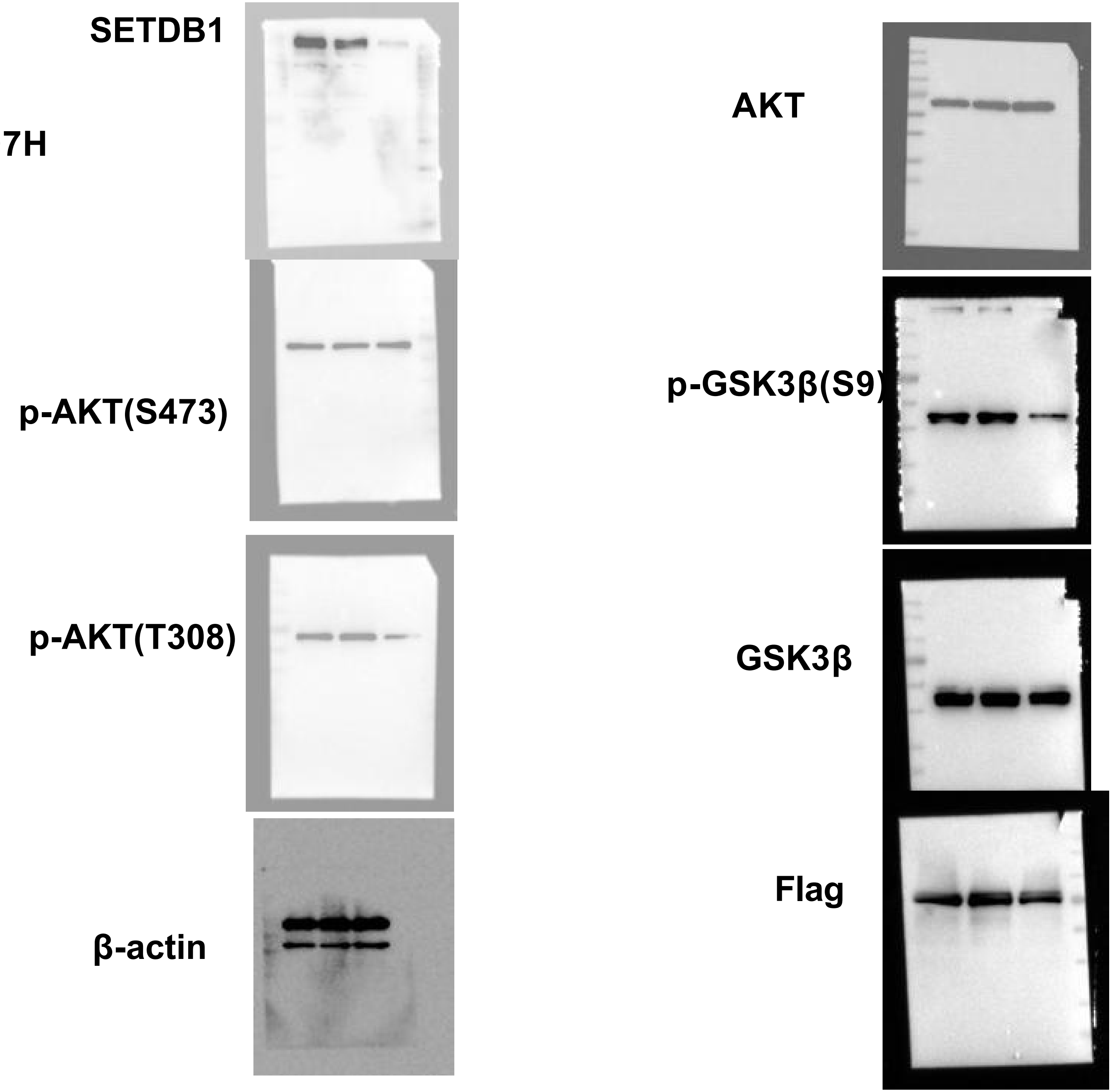

Fig. 4L

HepG2

SETDB1

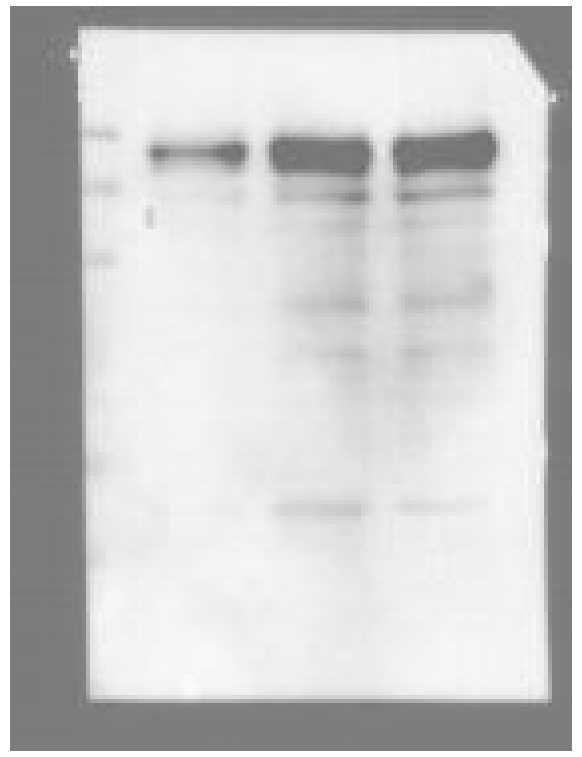

CHAF1B

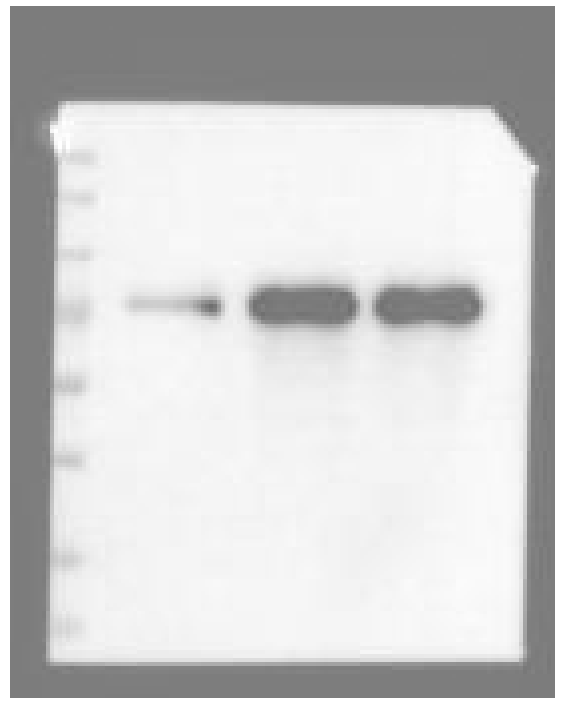

β-actin

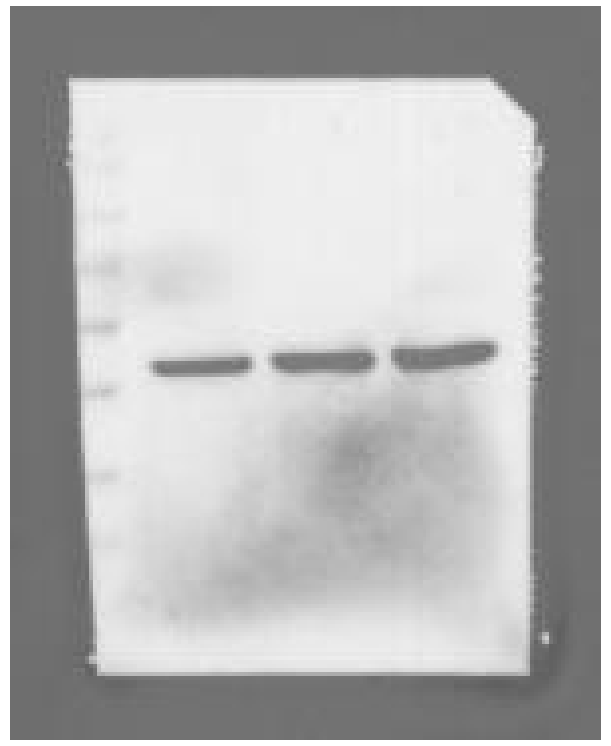

AKT

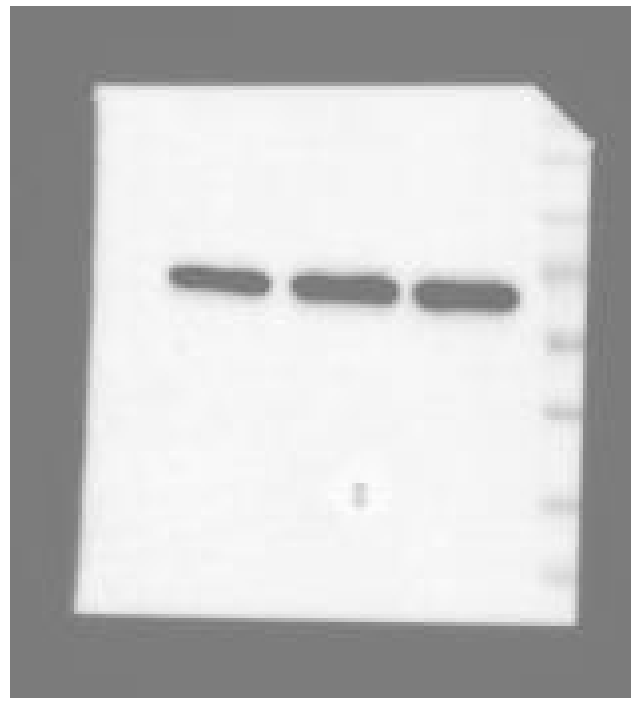

p-AKT(S473)

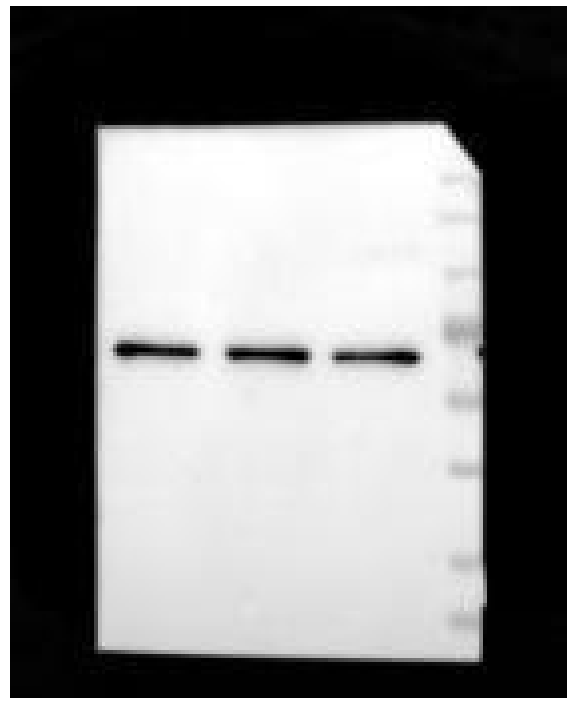

p-AKT(T308)

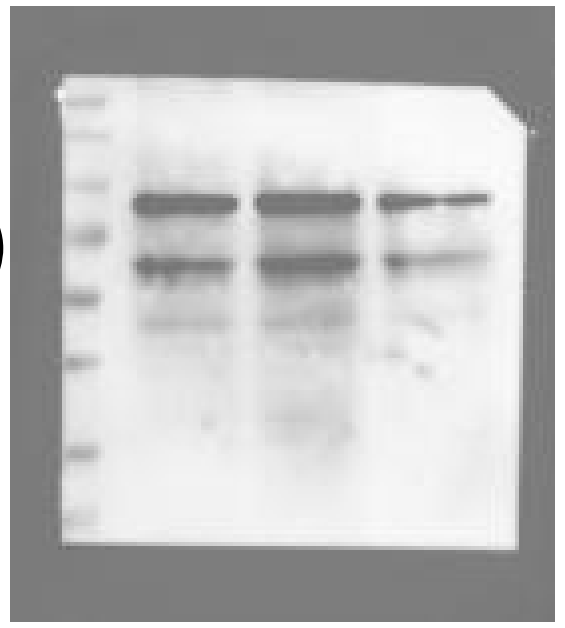

GSK3β

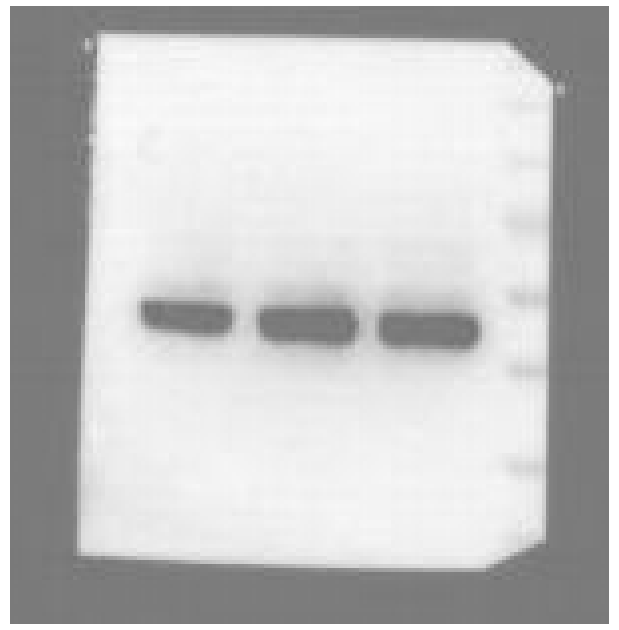

p-GSK3β(S9)

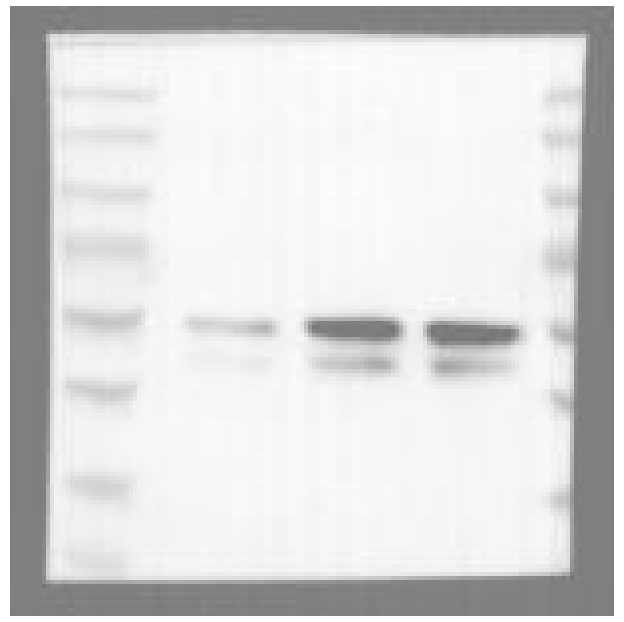

Fig. 4L

MHCC97H

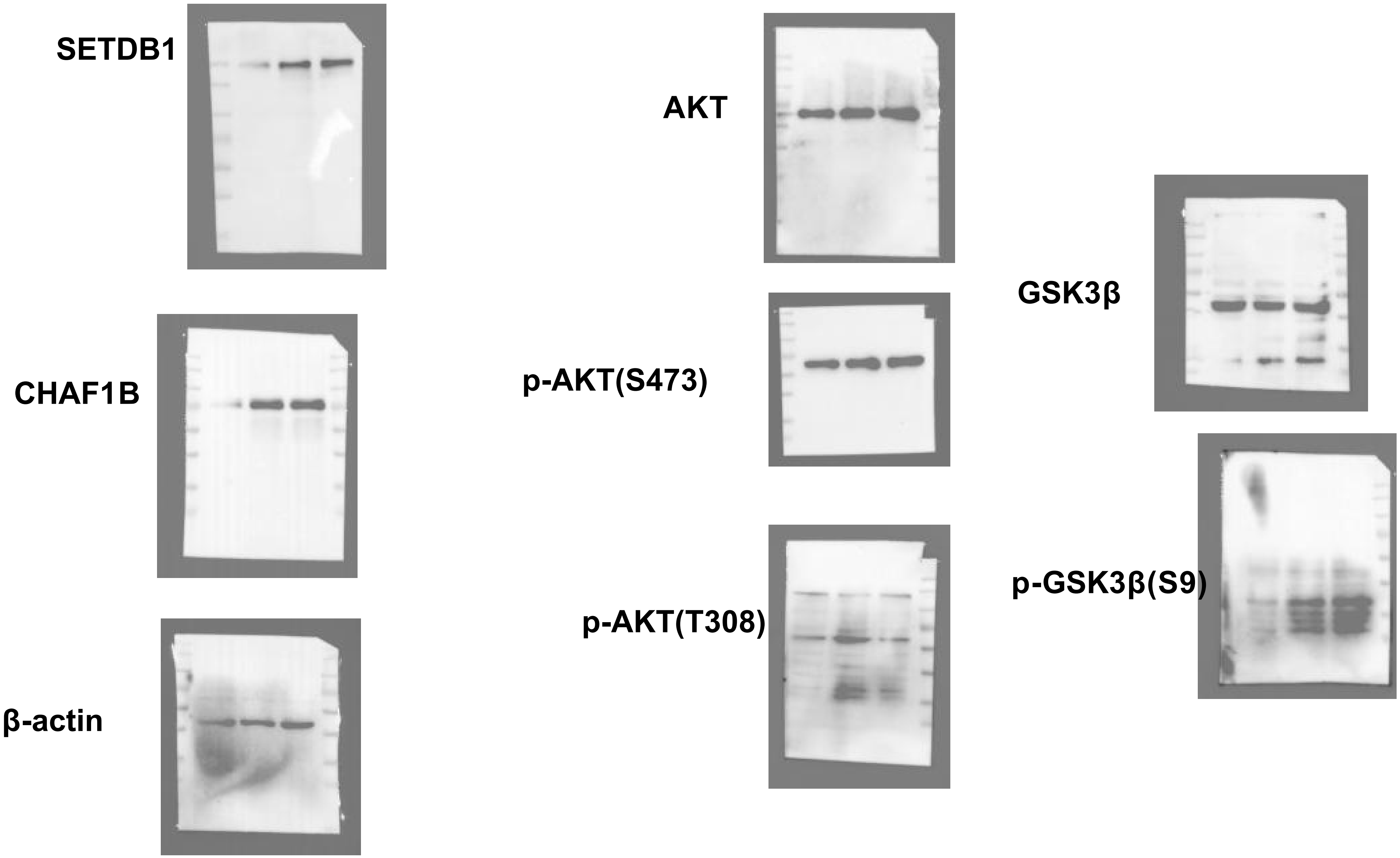

Fig. 4N Huh7

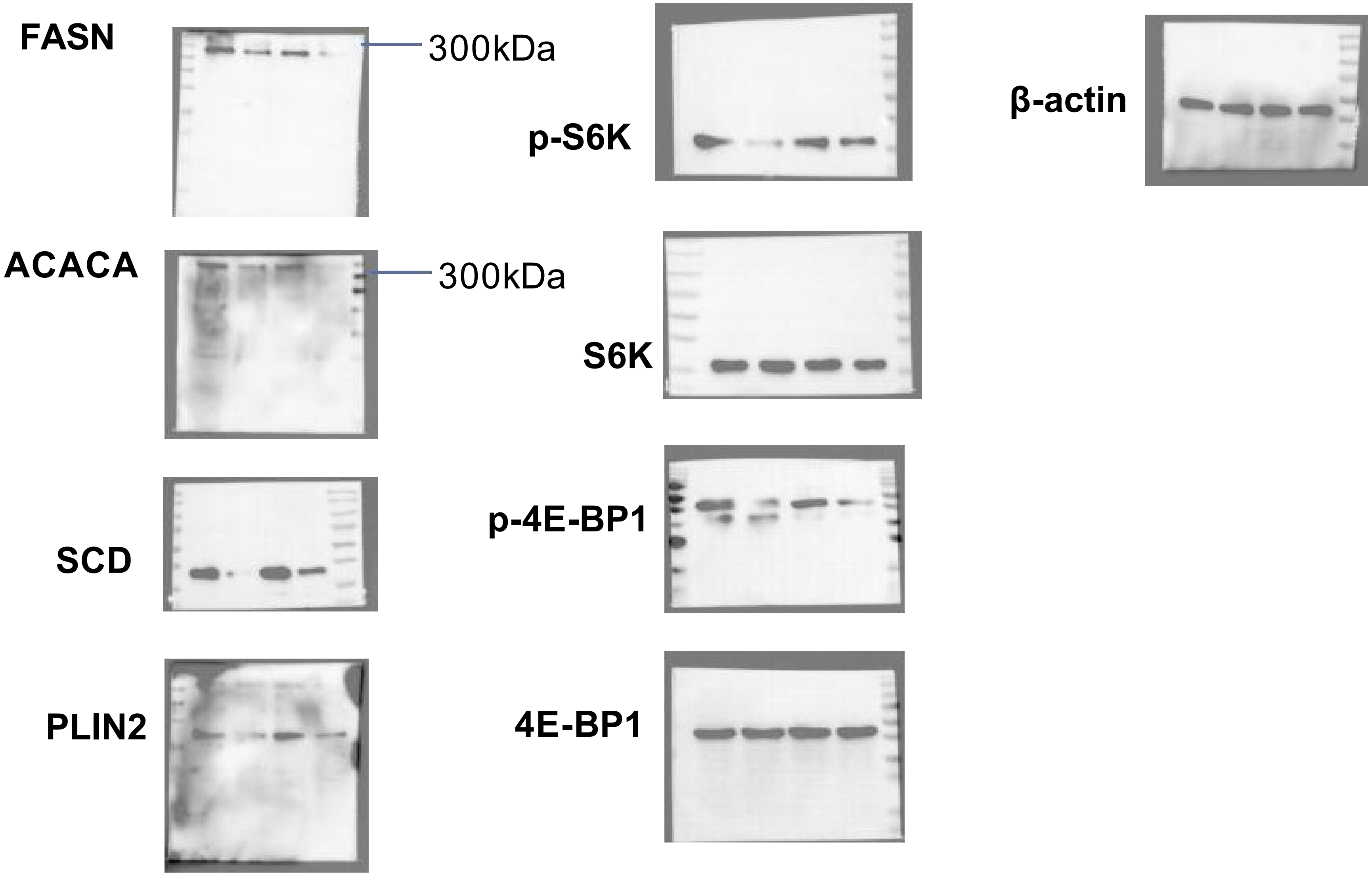
FASN
300kDa
ACACA
300kDa
SCD
PLIN2
p-S6K
S6K
p-4E-BP1
4E-BP1
β-actin

Fig. 5C

HEK293T

CHAF1B

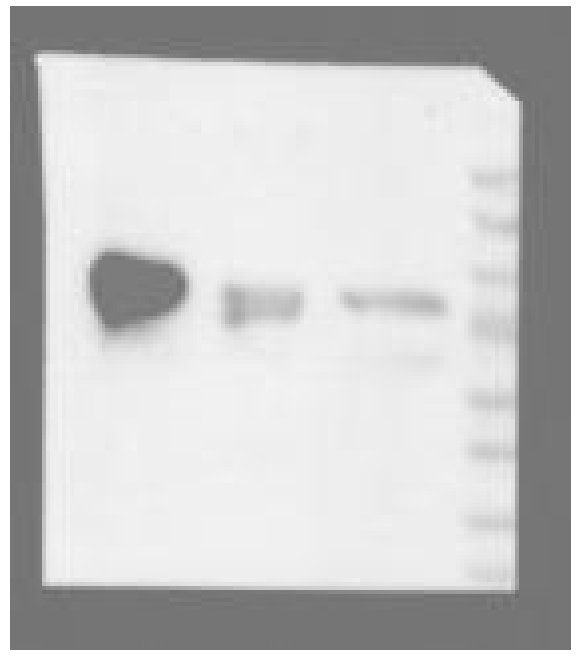

SETDB1

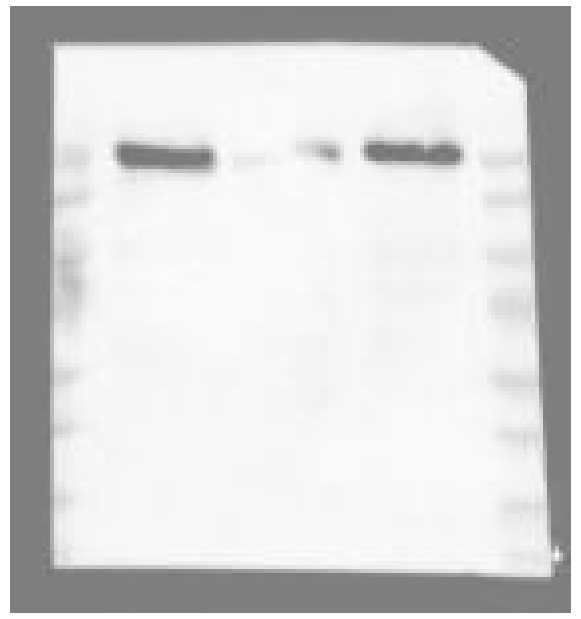

UHRF1

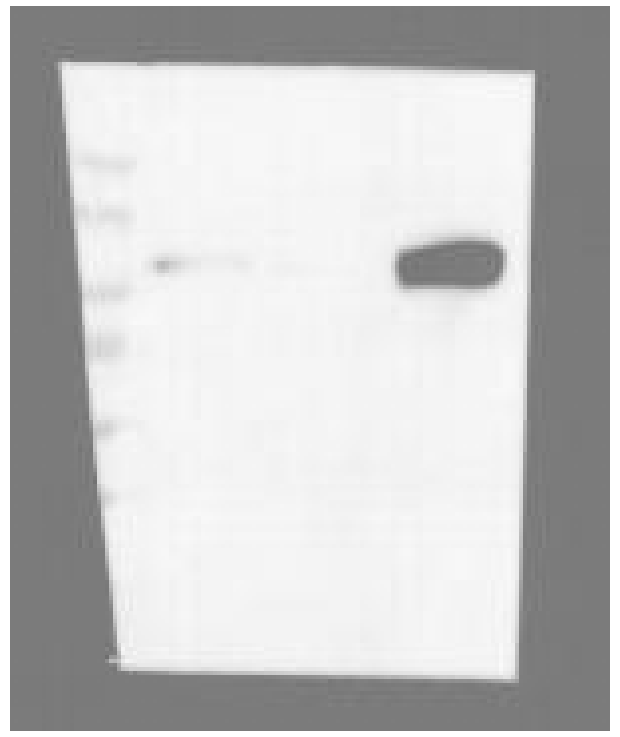

β-actin

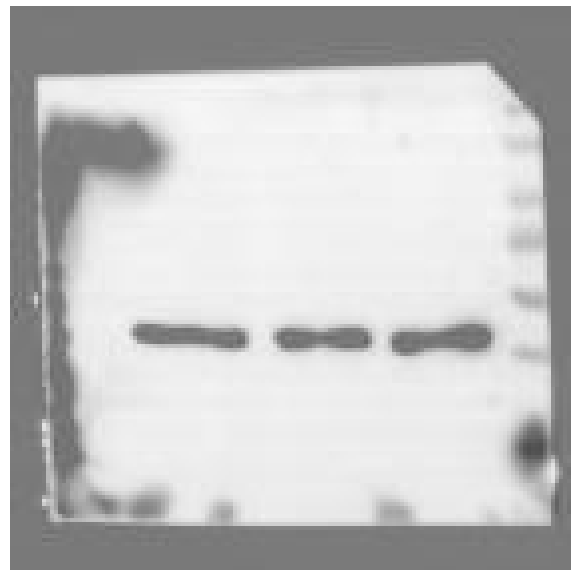

Fig. 5F

Huh7

IB: UHRF1

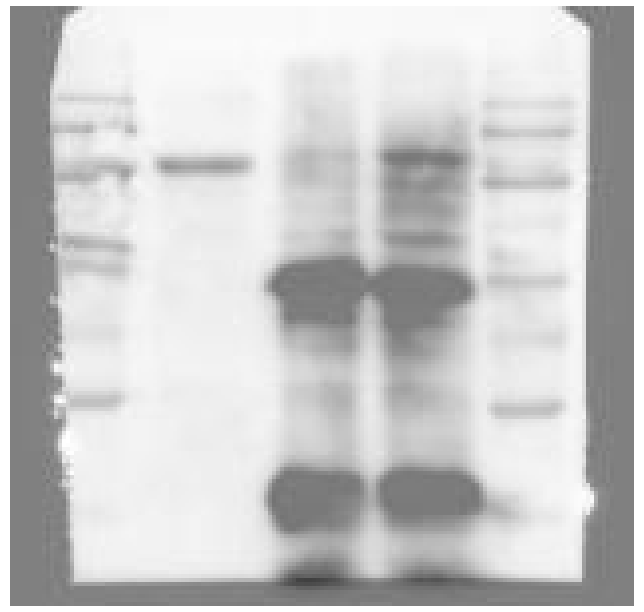

IB: CHAF1B

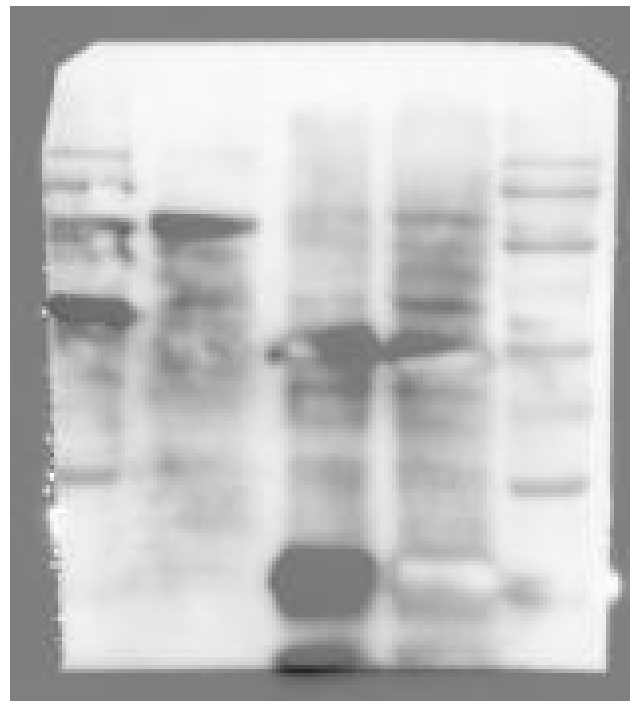

IB: CHAF1B

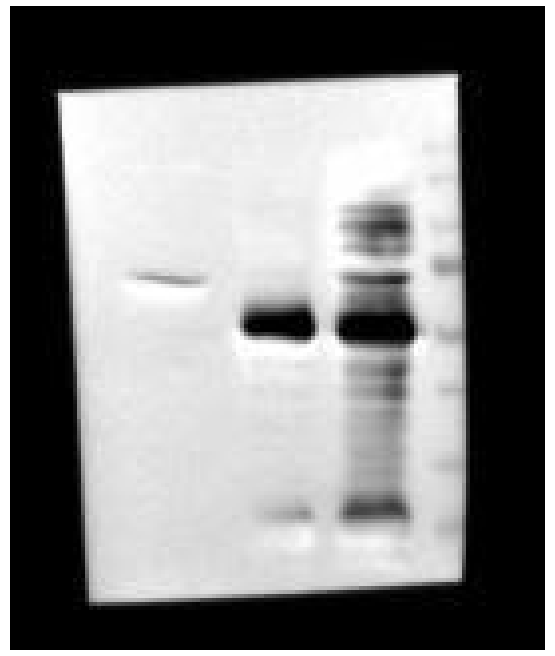

IB: UHRF1

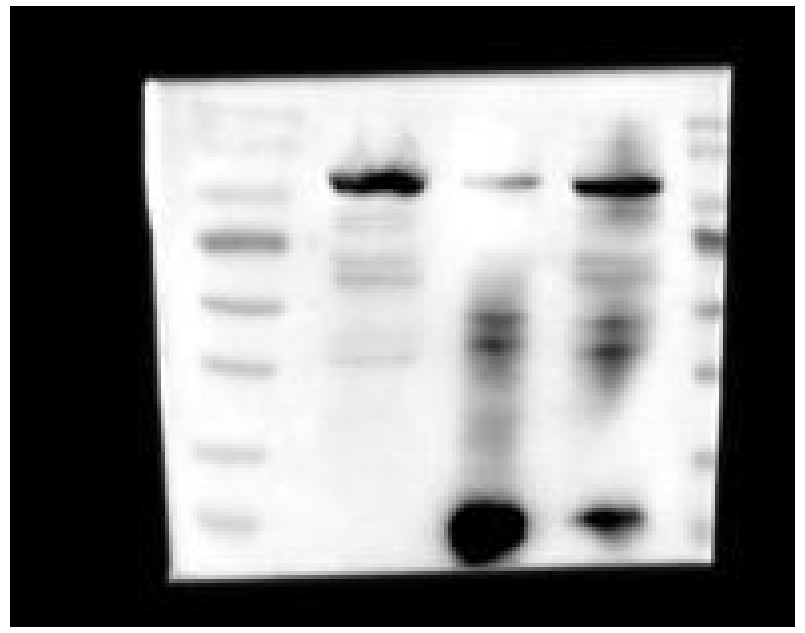

SK-Hep1

IB: UHRF1

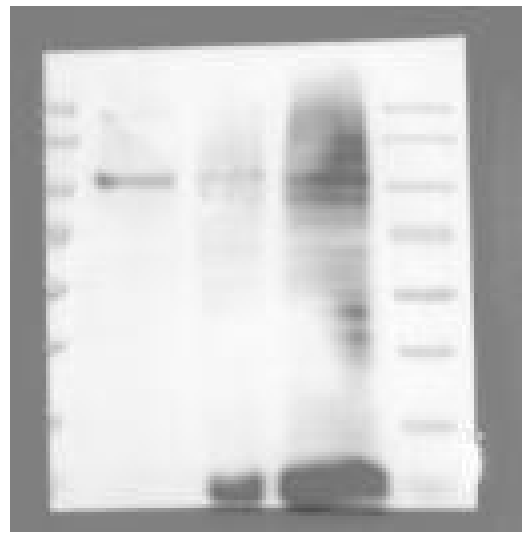

IB: CHAF1B

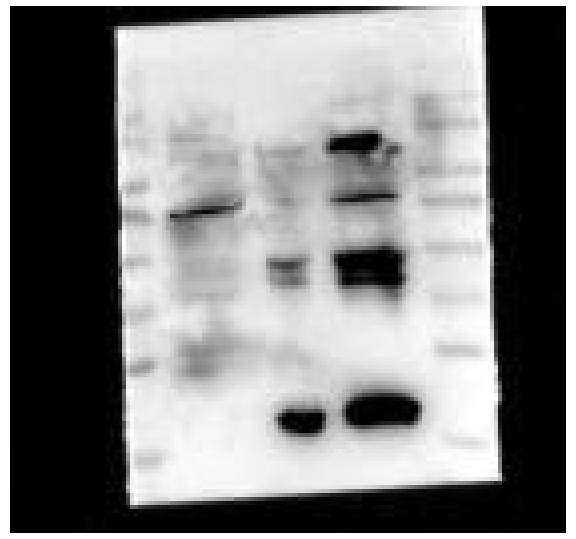

IB: CHAF1B

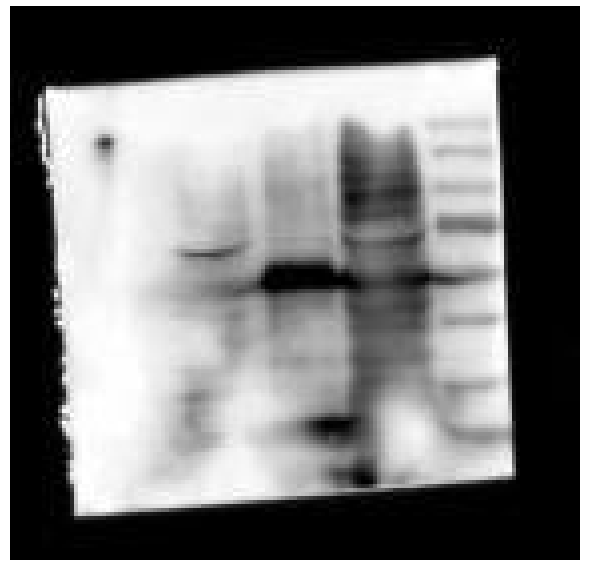

IB: UHRF1

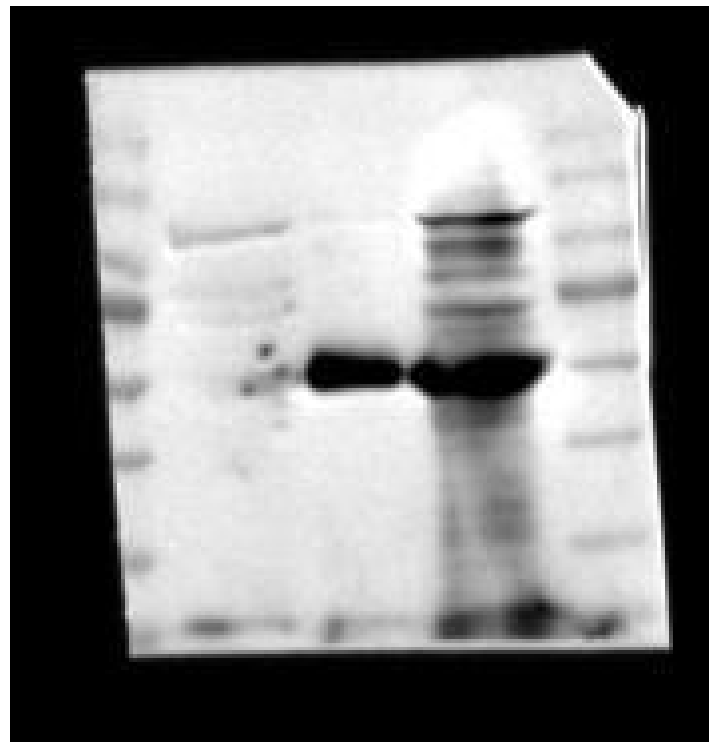

Fig. 5G

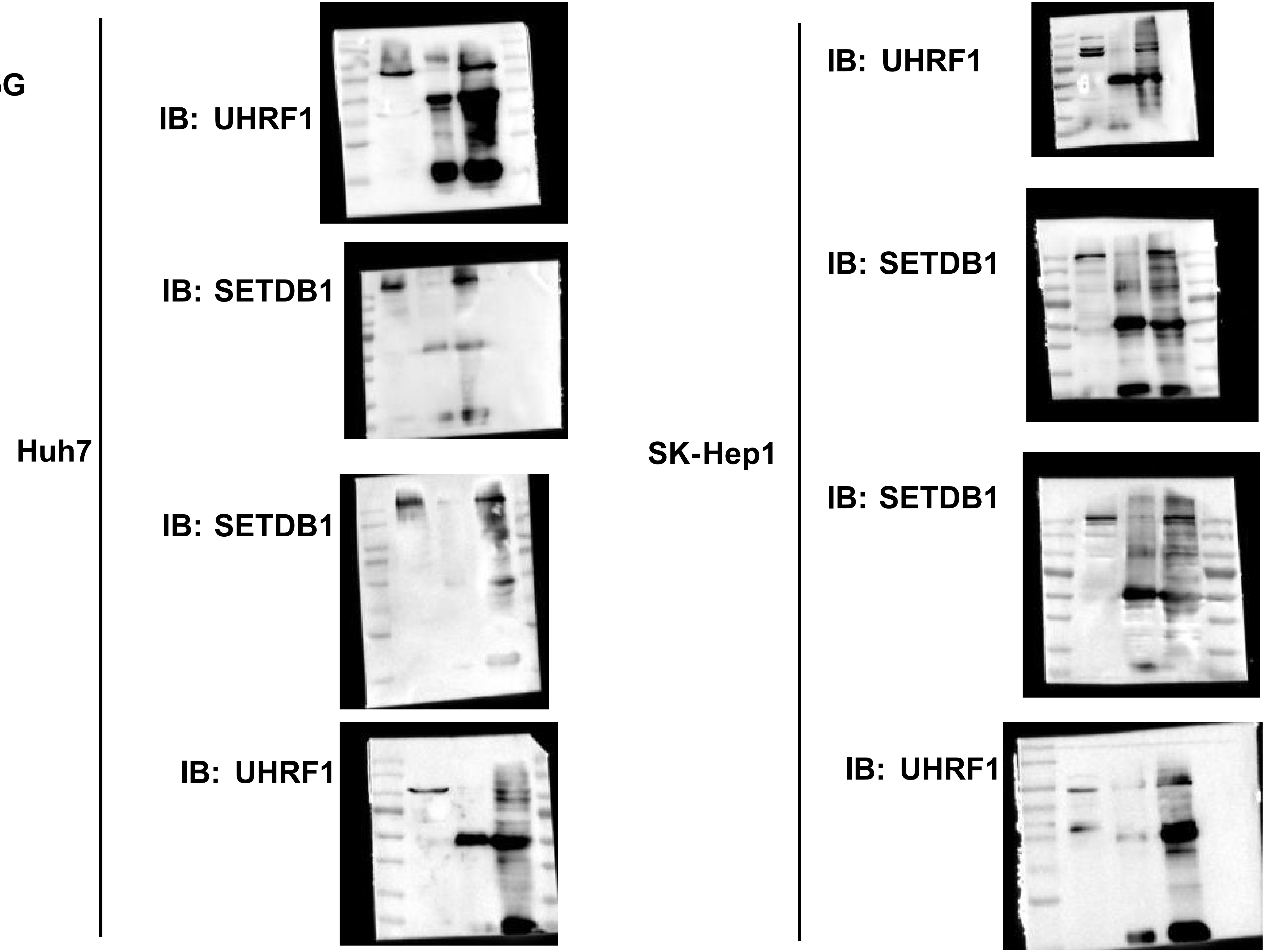
Huh7
IB: UHRF1
IB: SETDB1
IB: SETDB1
IB: UHRF1
SK-Hep1
IB: UHRF1
IB: SETDB1
IB: SETDB1
IB: UHRF1

Fig. 5H HEK293T

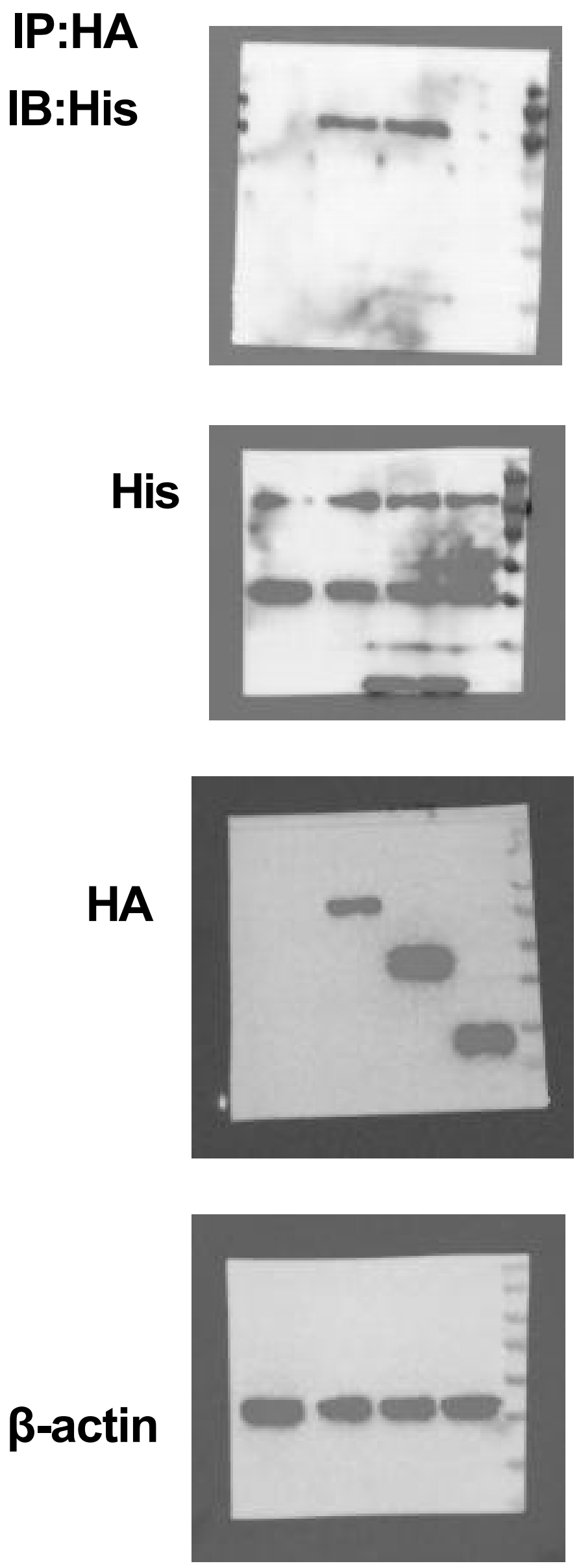

Fig. 5I Huh7

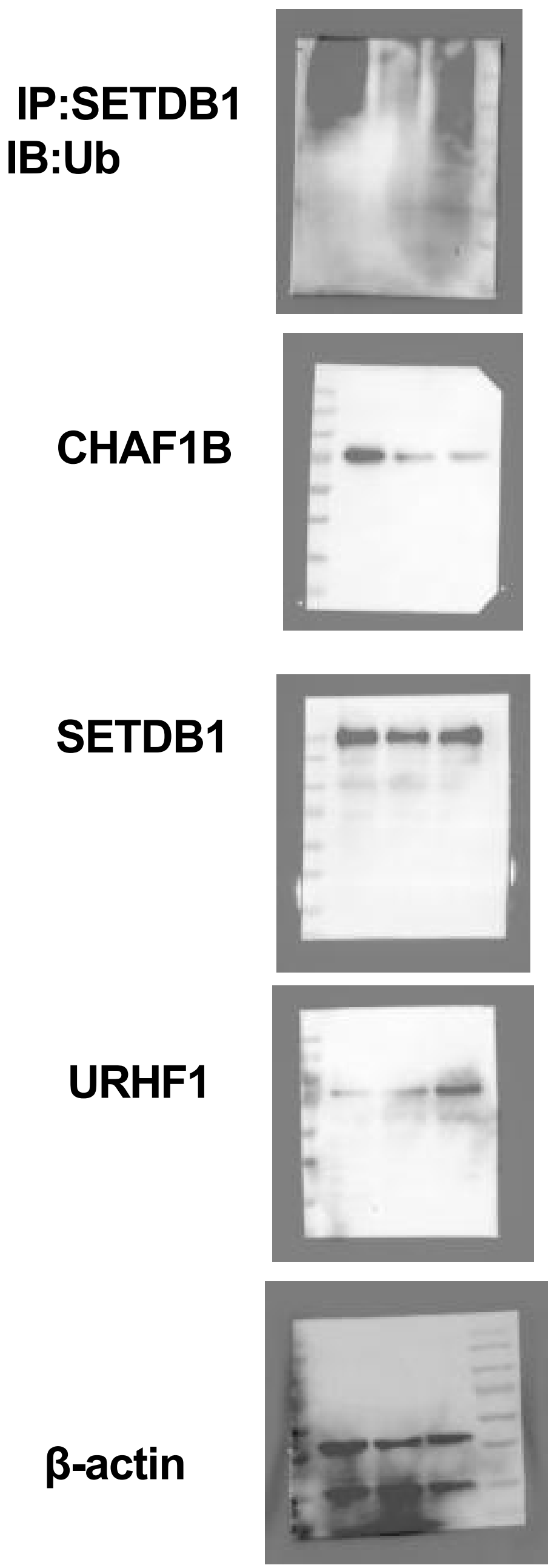

Fig. 5J HEK293T

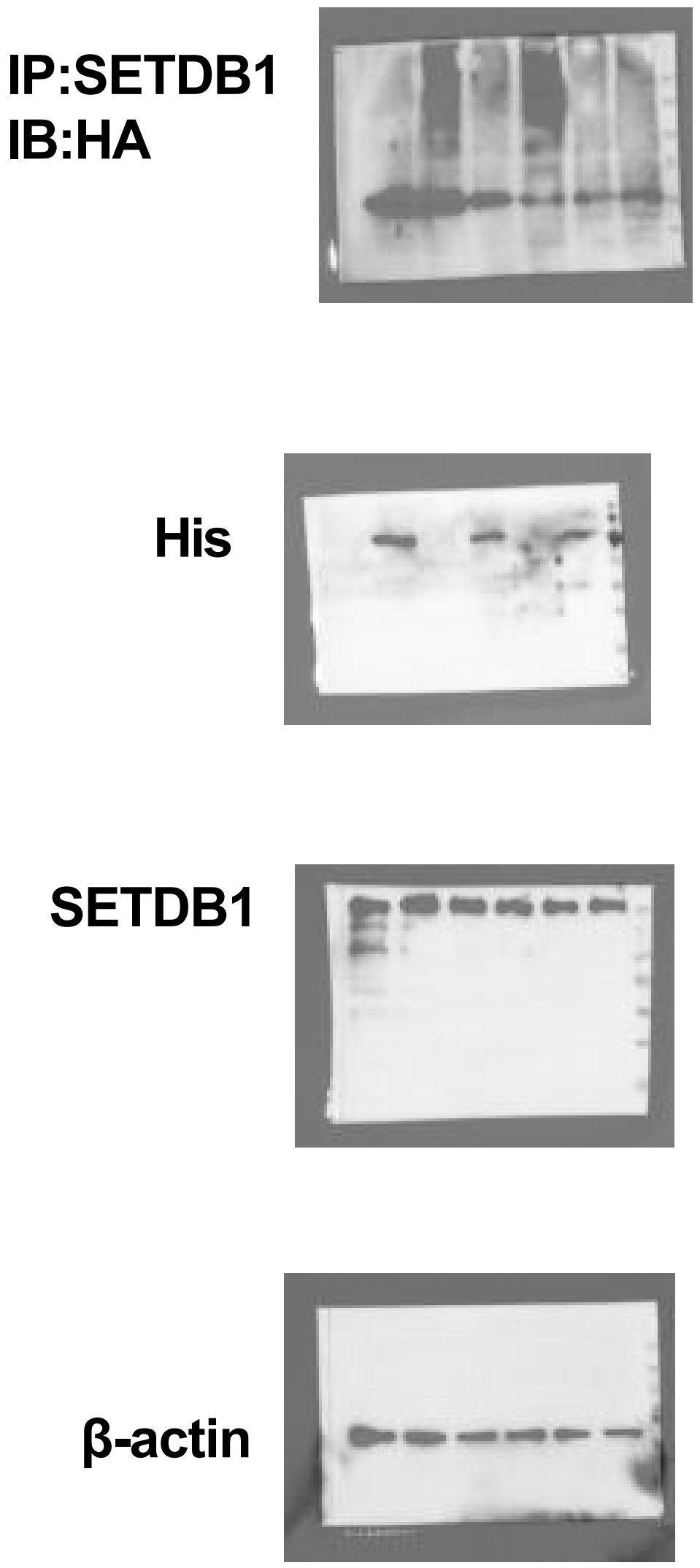

Fig. 5K

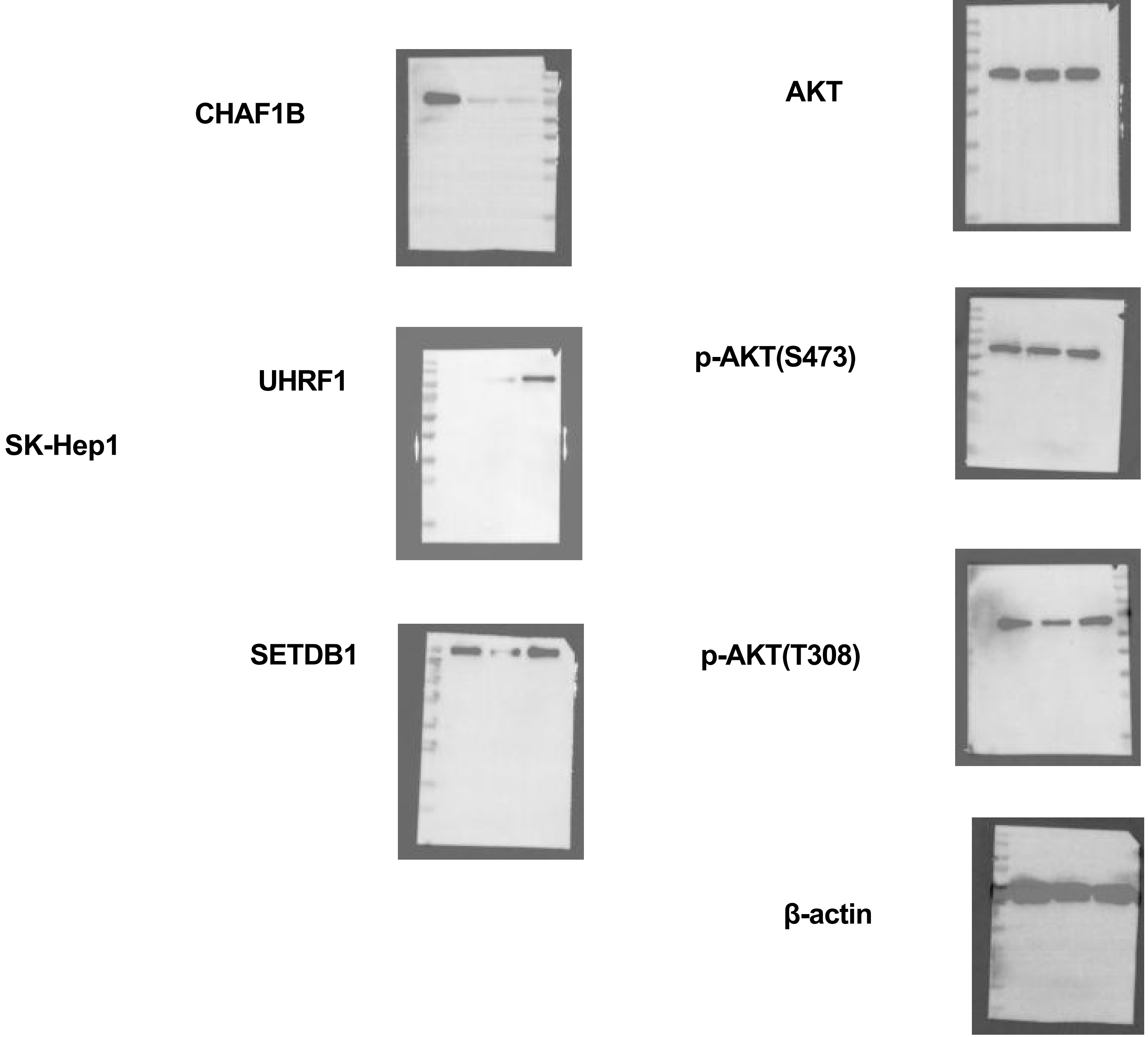

Fig. 7A

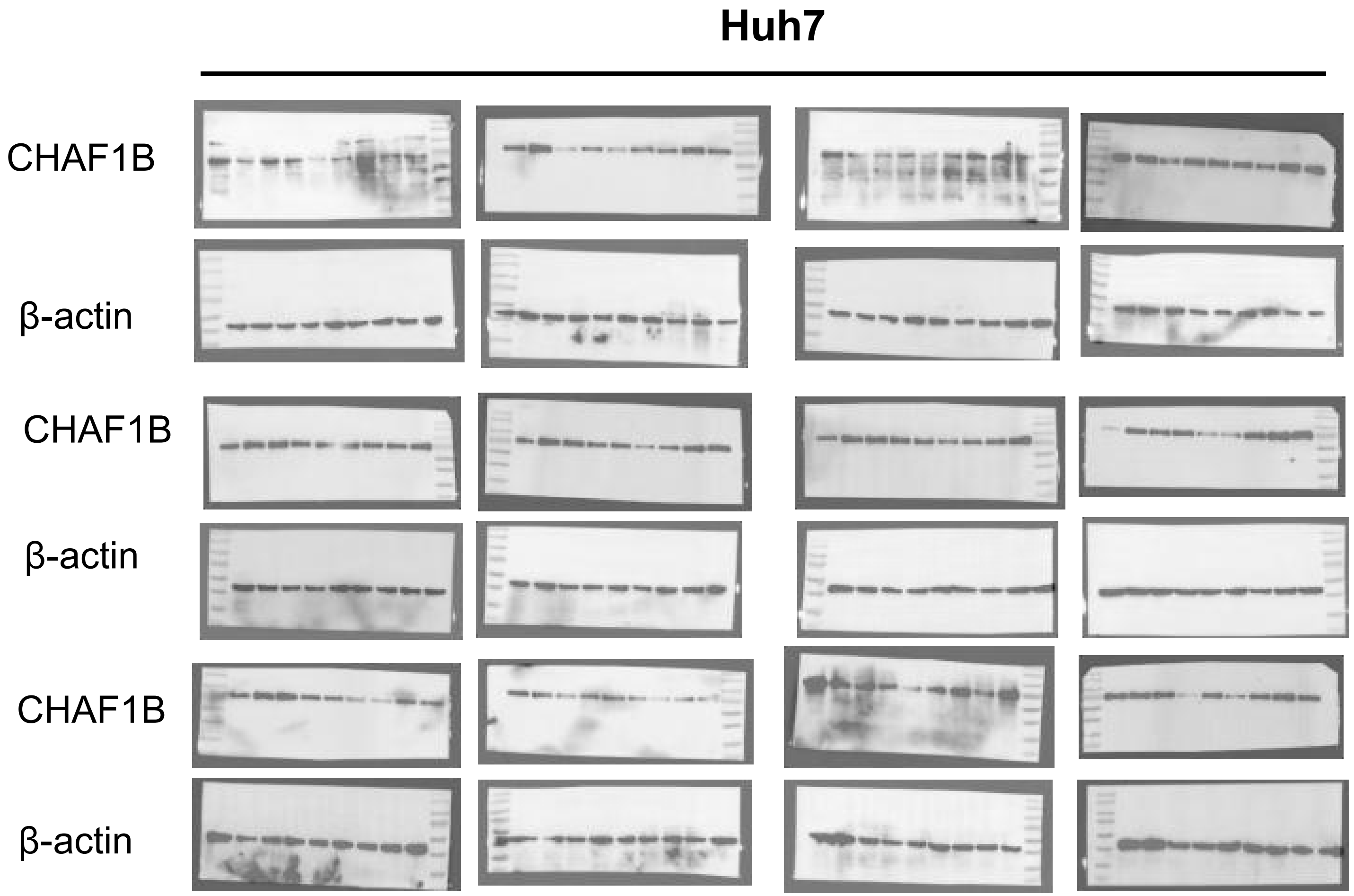

Fig. 7C

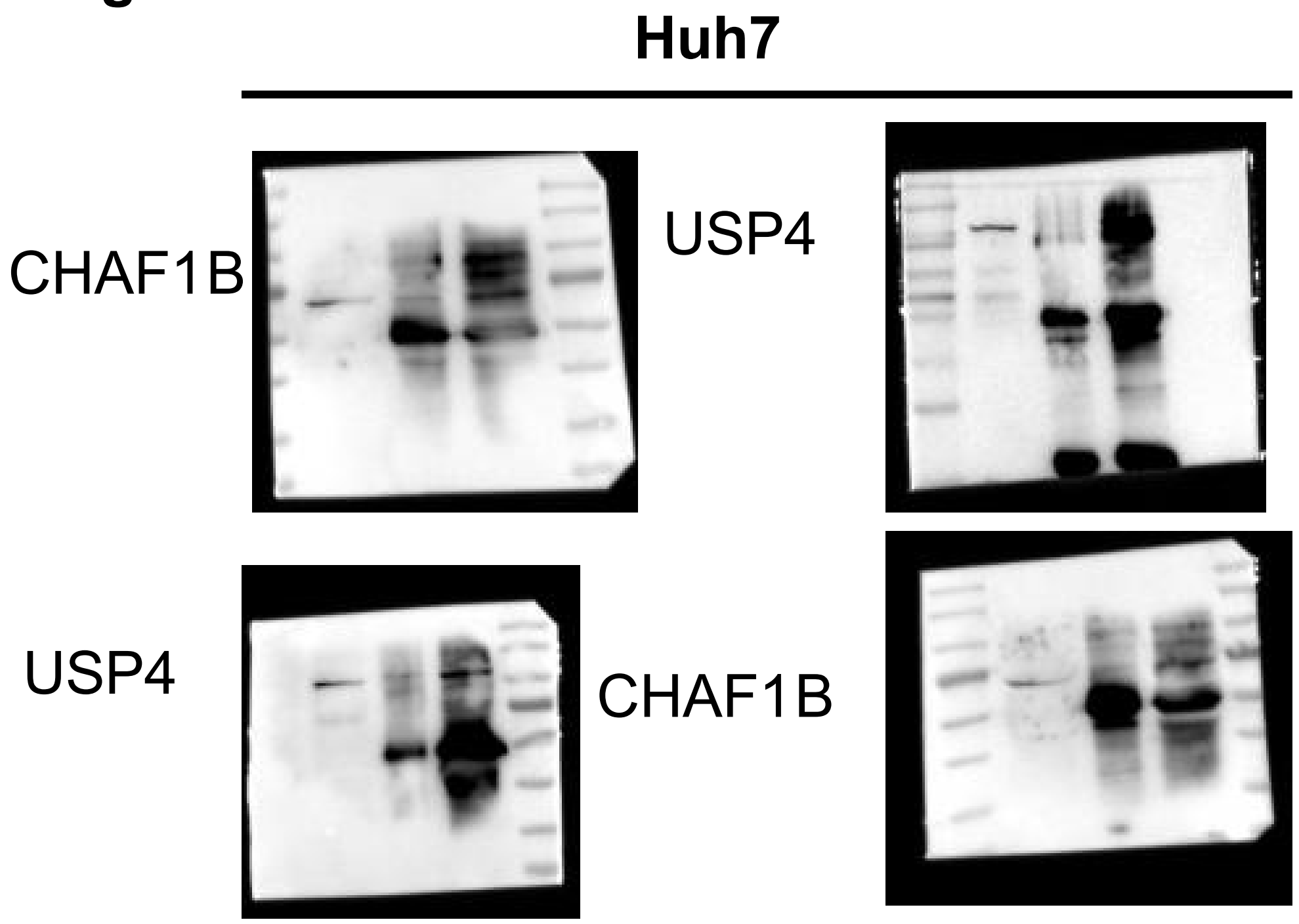
Huh7
CHAF1B
USP4
USP4
CHAF1B

Fig. 7D

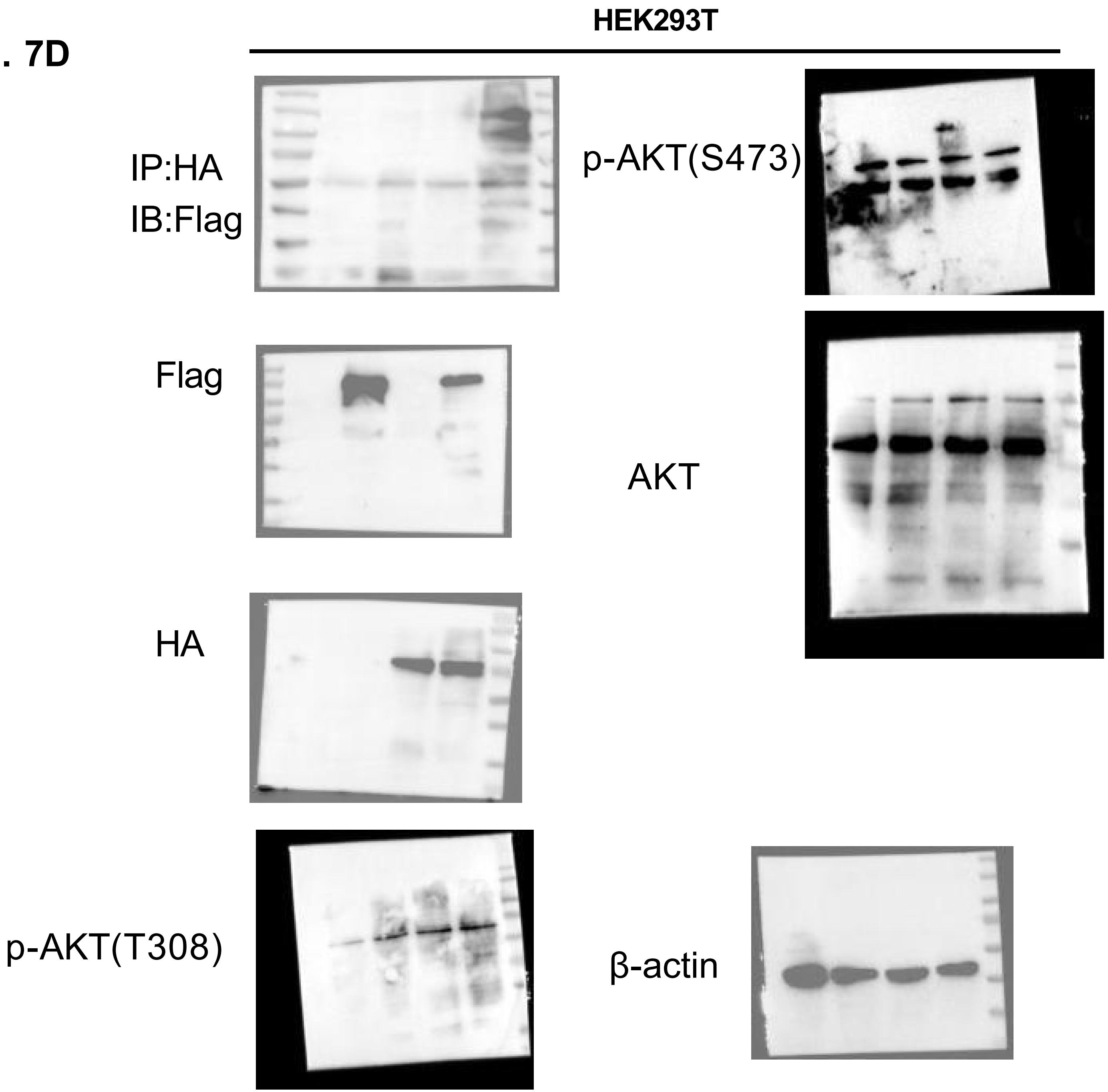
HEK293T
IP:HA
IB:Flag
p-AKT(S473)
Flag
AKT
HA
p-AKT(T308)
β-actin

Fig. 7D

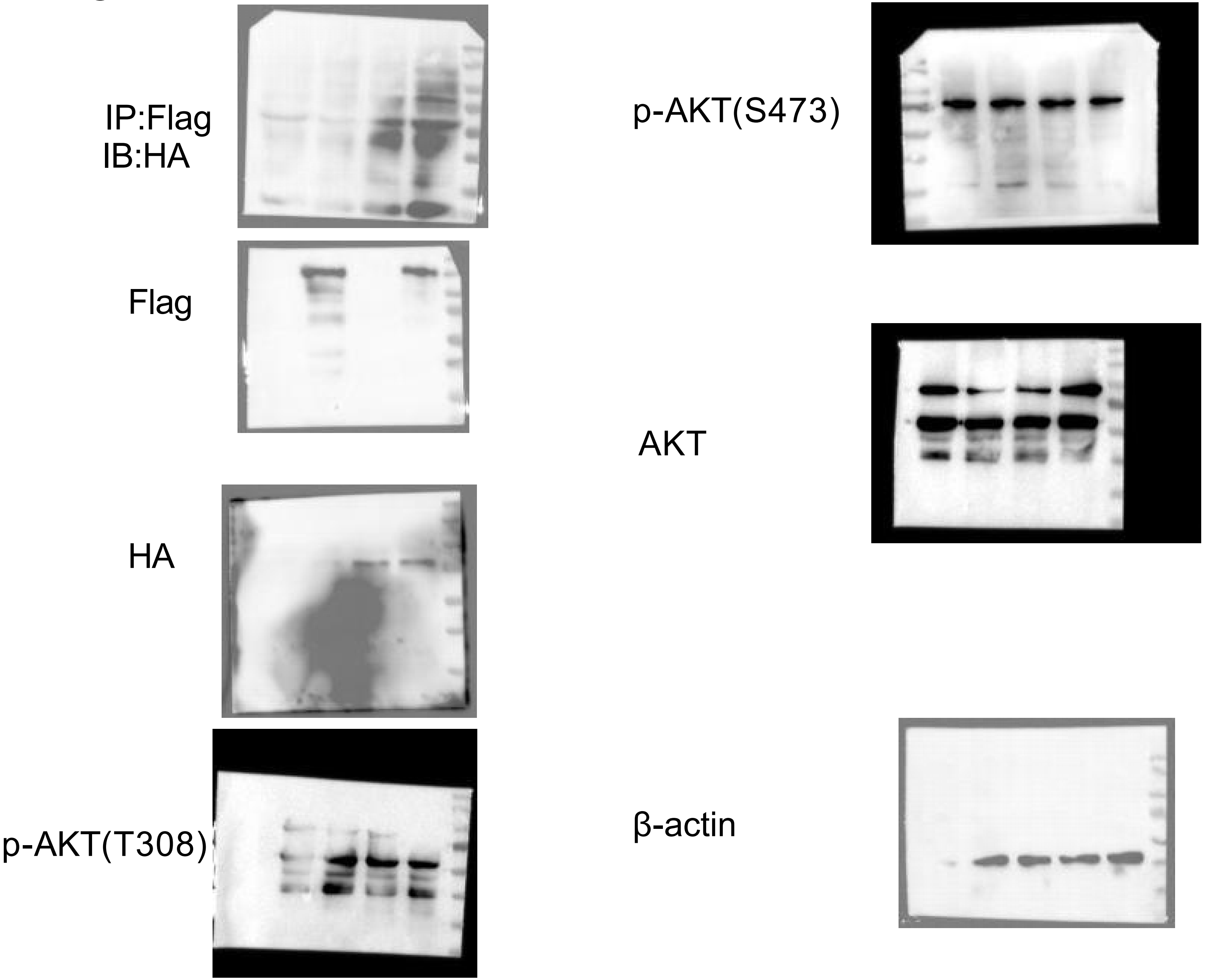

Fig. 7E

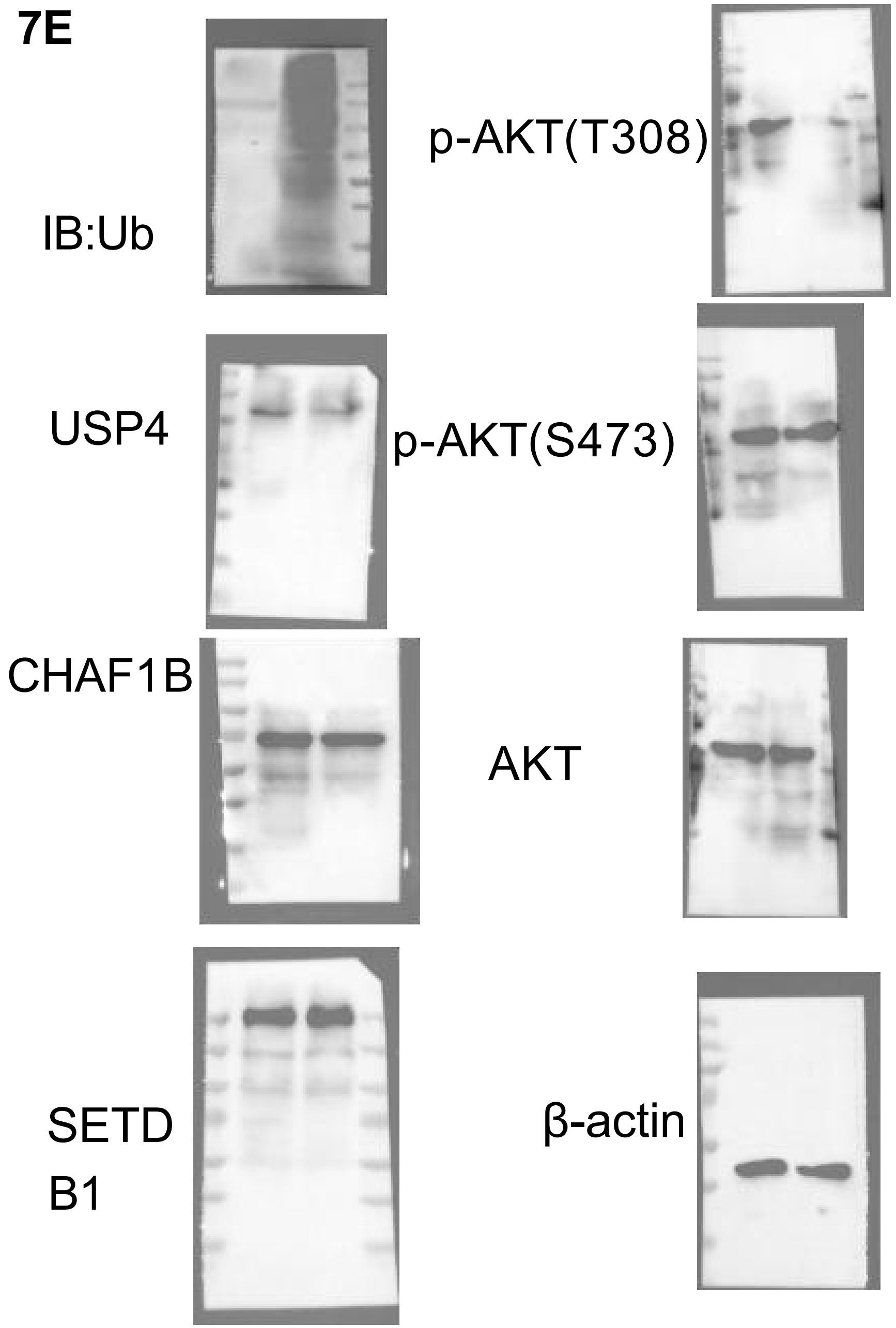

IB:Ub
USP4
CHAF1B
SETD
B1
p-AKT(T308)
p-AKT(S473)
AKT
β-actin

Fig. 7E

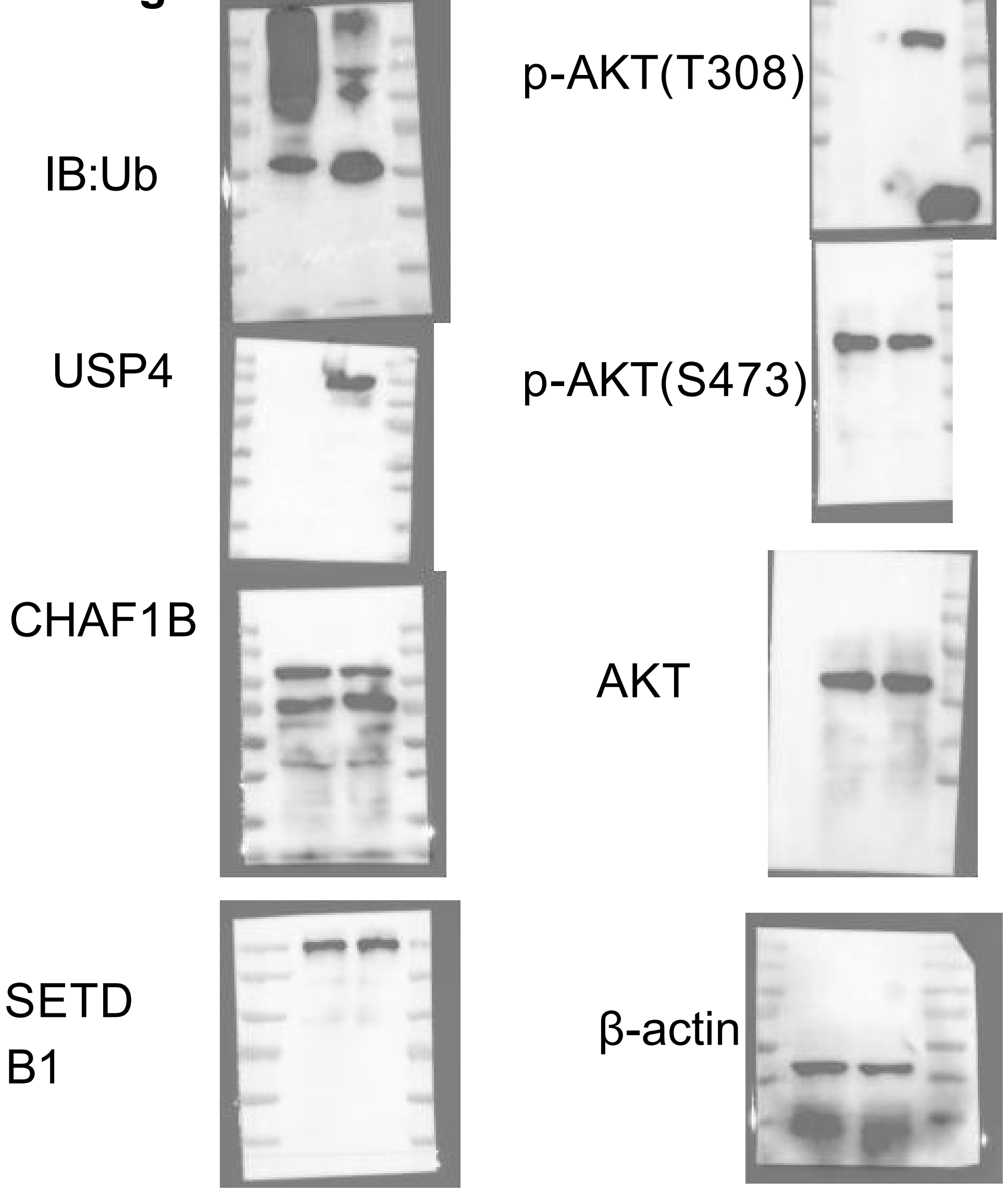

IB:Ub
USP4
CHAF1B
SETD
B1
p-AKT(T308)
p-AKT(S473)
AKT
β-actin

Fig. 7G

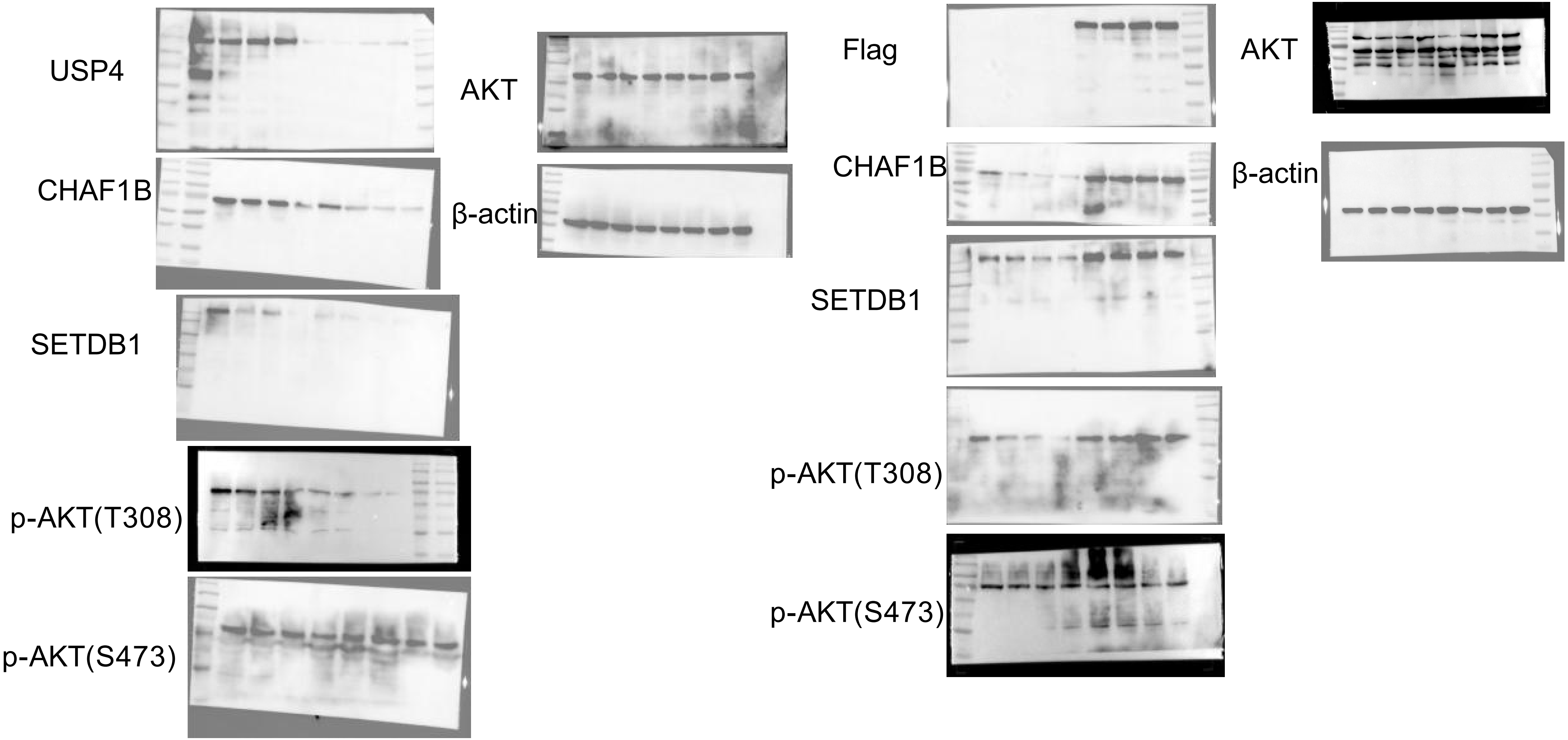

USP4
AKT
CHAF1B
β-actin
SETDB1
p-AKT(T308)
p-AKT(S473)
Flag
CHAF1B
SETDB1
p-AKT(T308)
p-AKT(S473)
AKT
β-actin

Fig. 7H

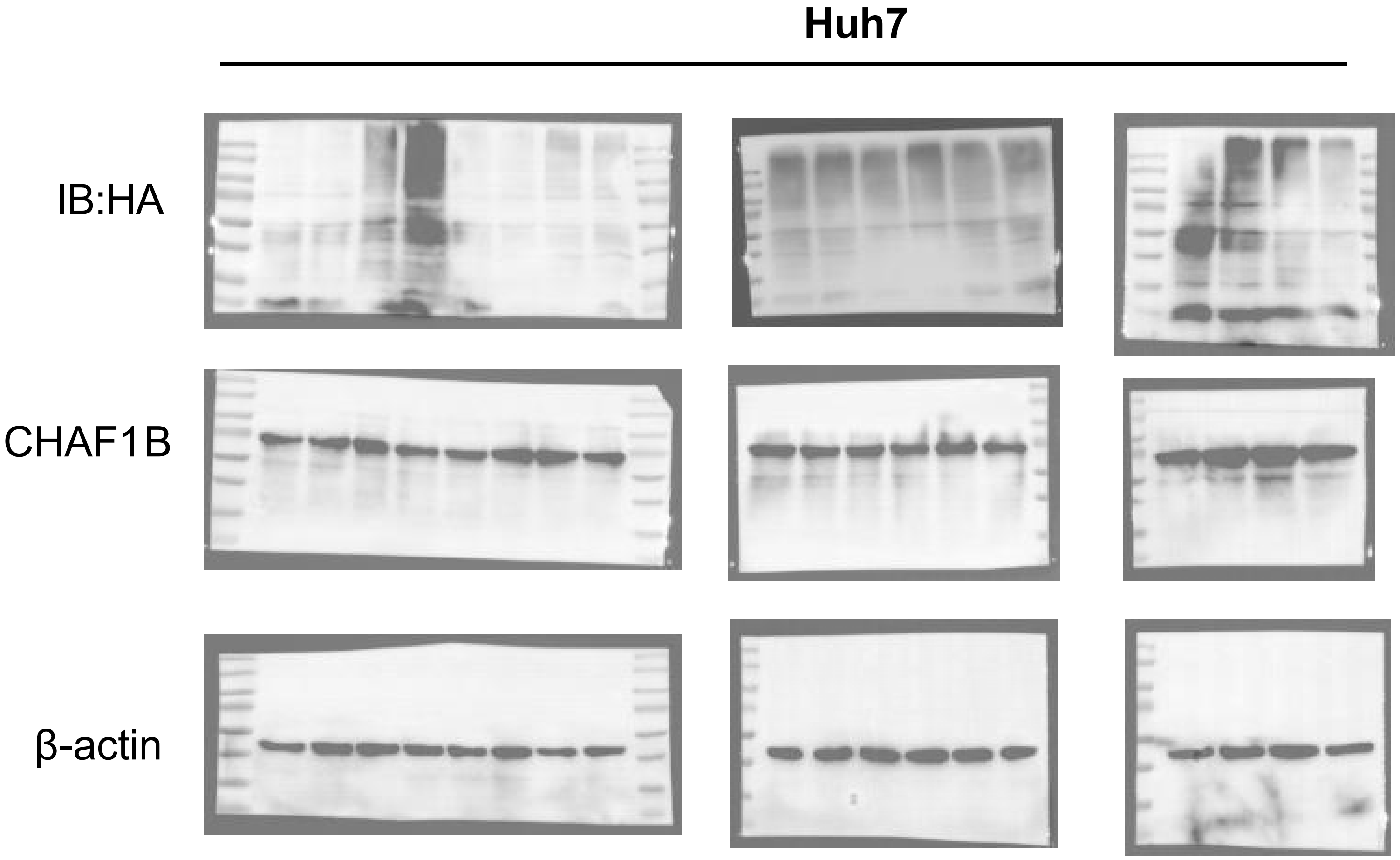

Fig. 8A

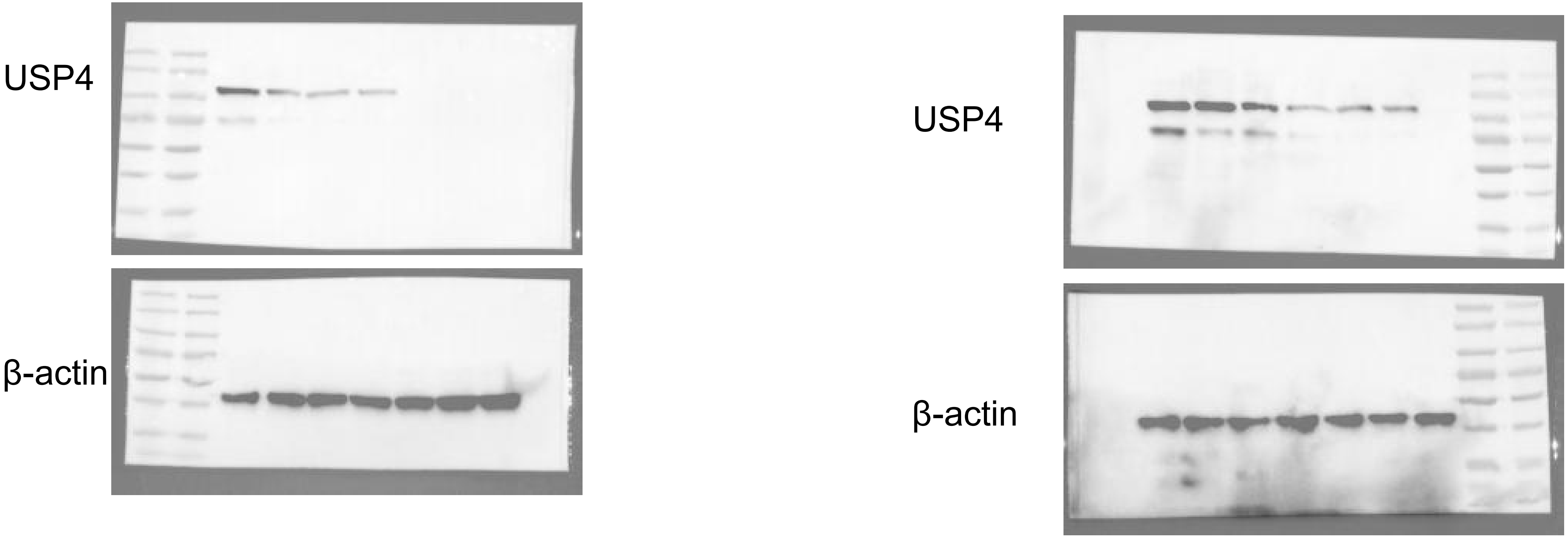

Fig. 8B

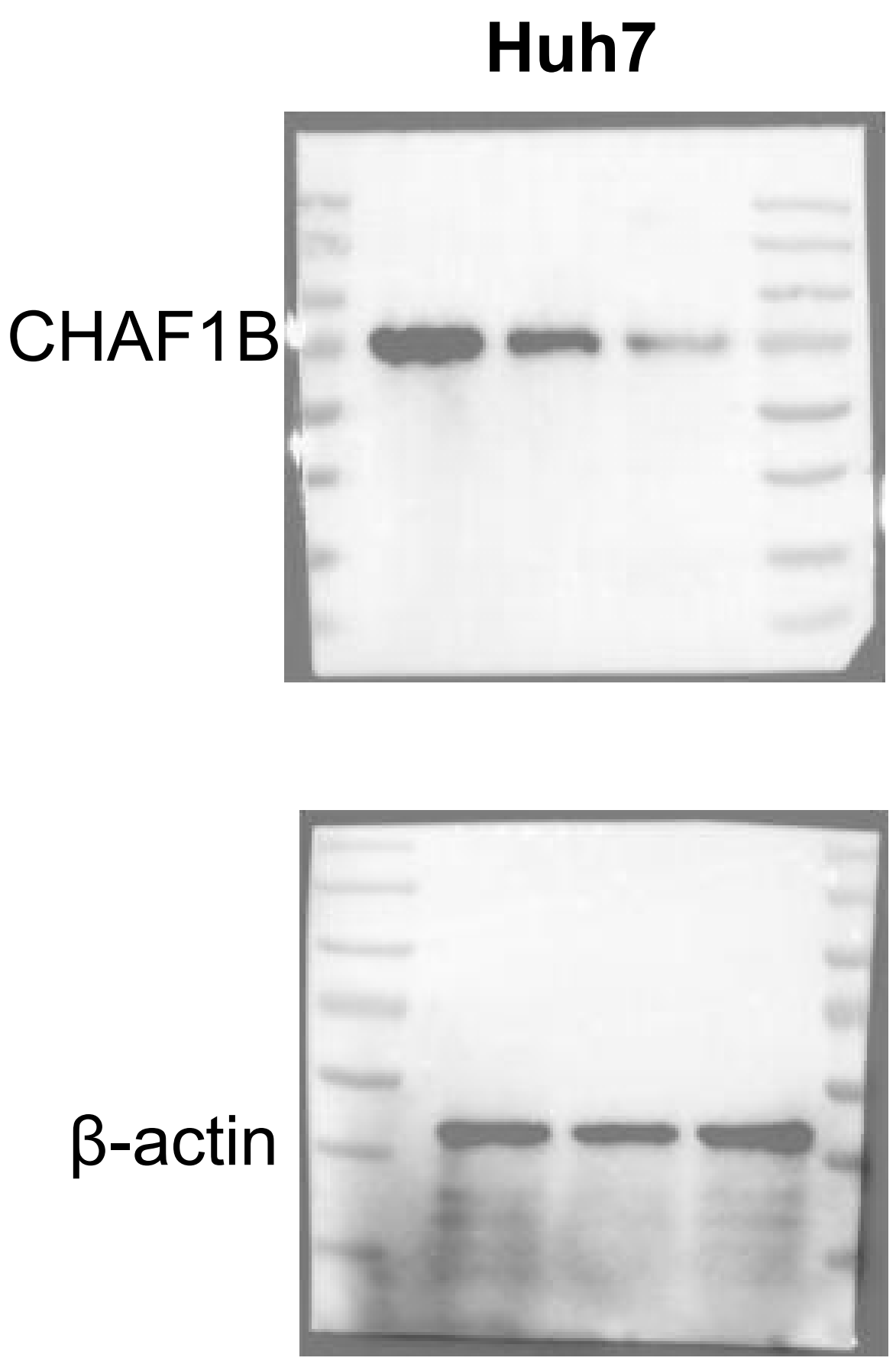

Fig. 8C

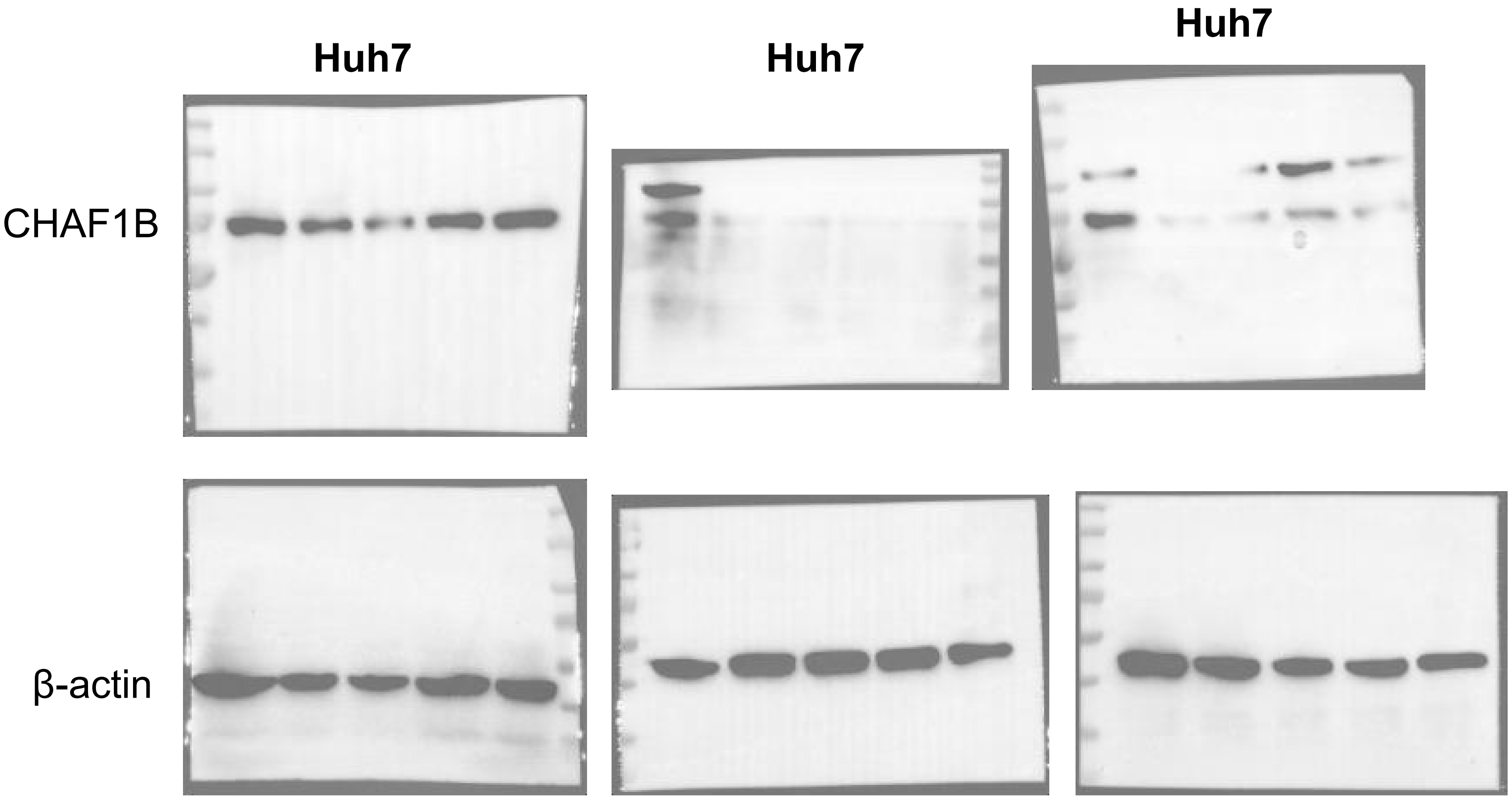

Fig. 8D

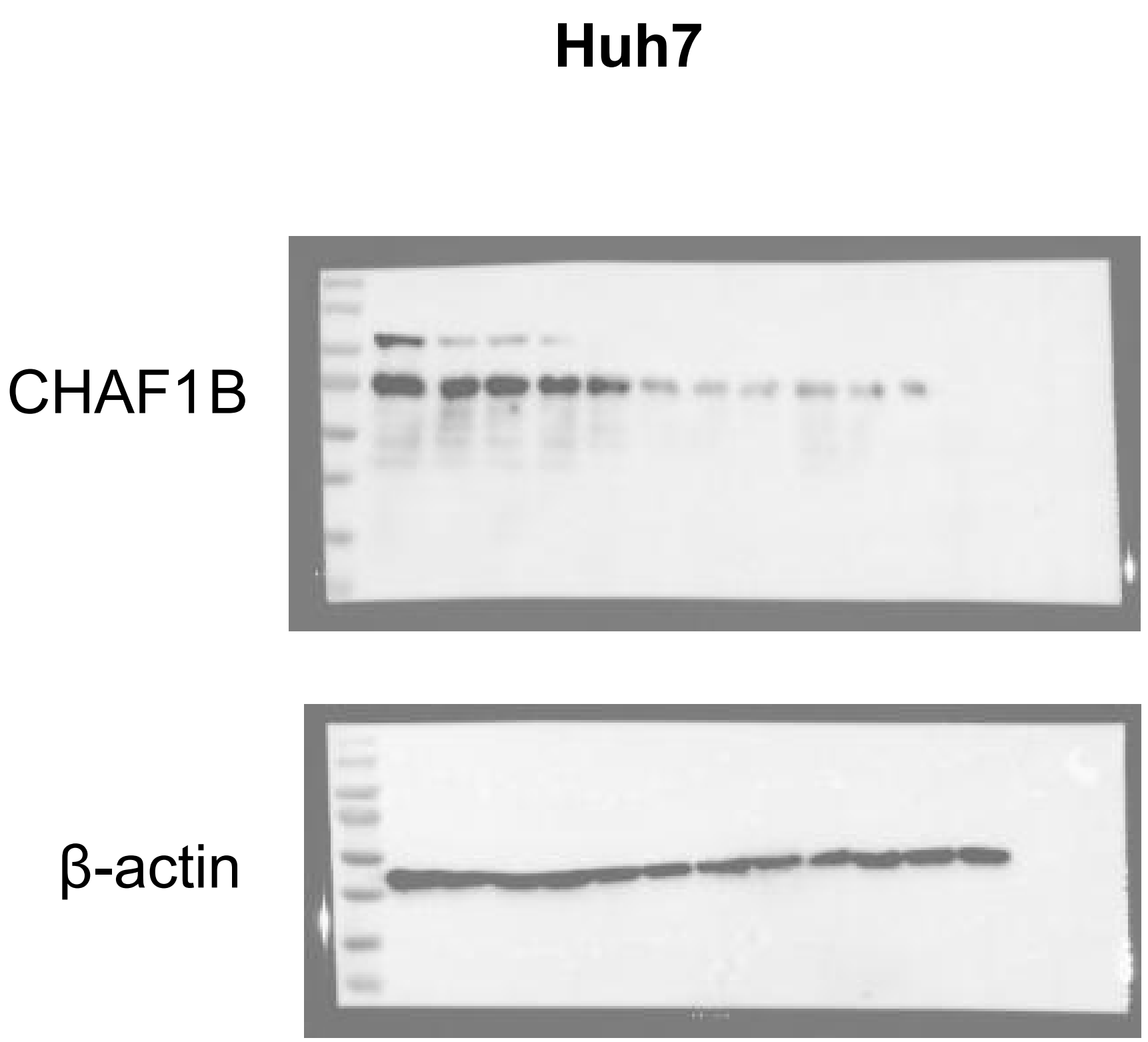

Fig. 8E

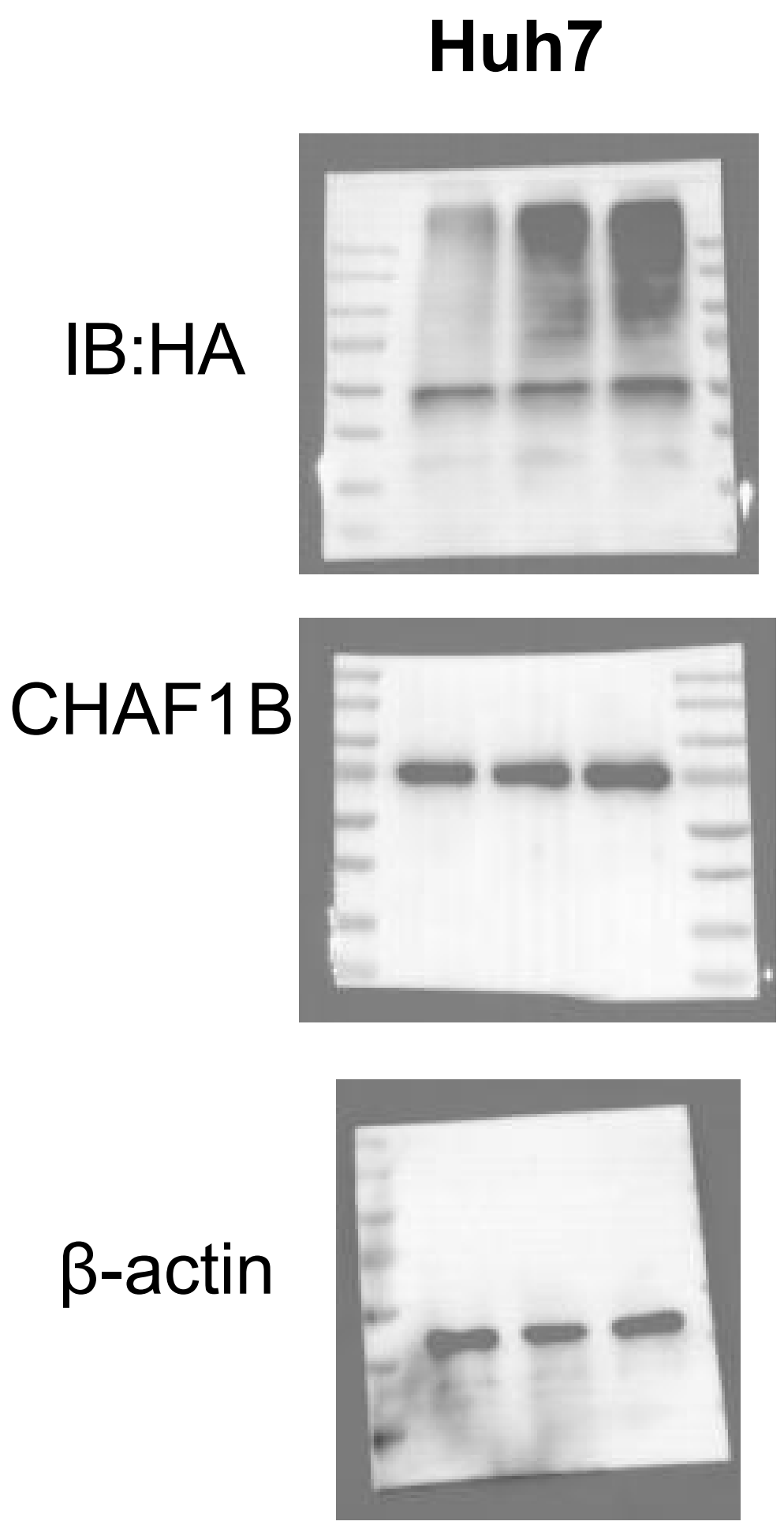

Fig. 8F

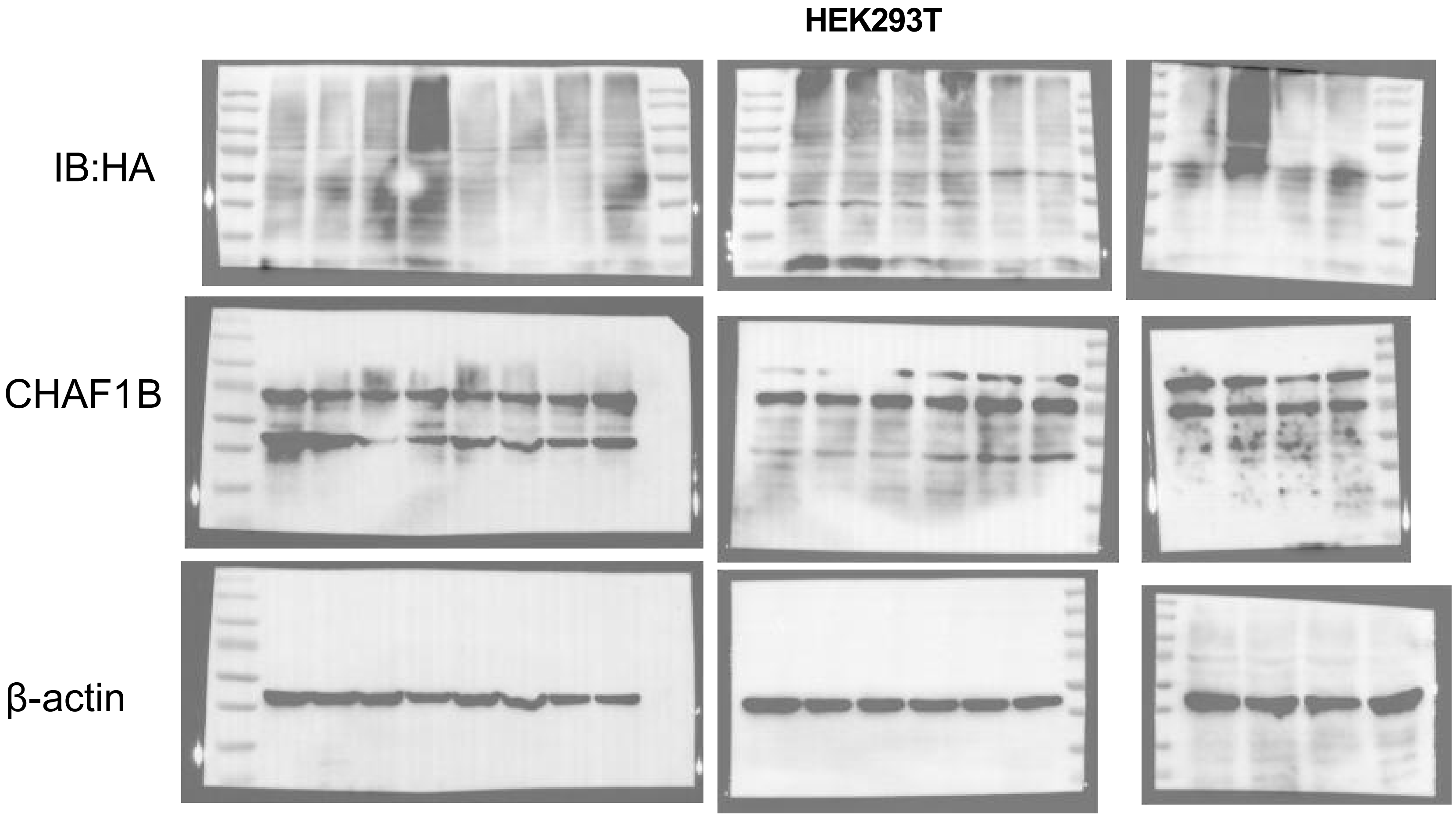

Fig. 8G

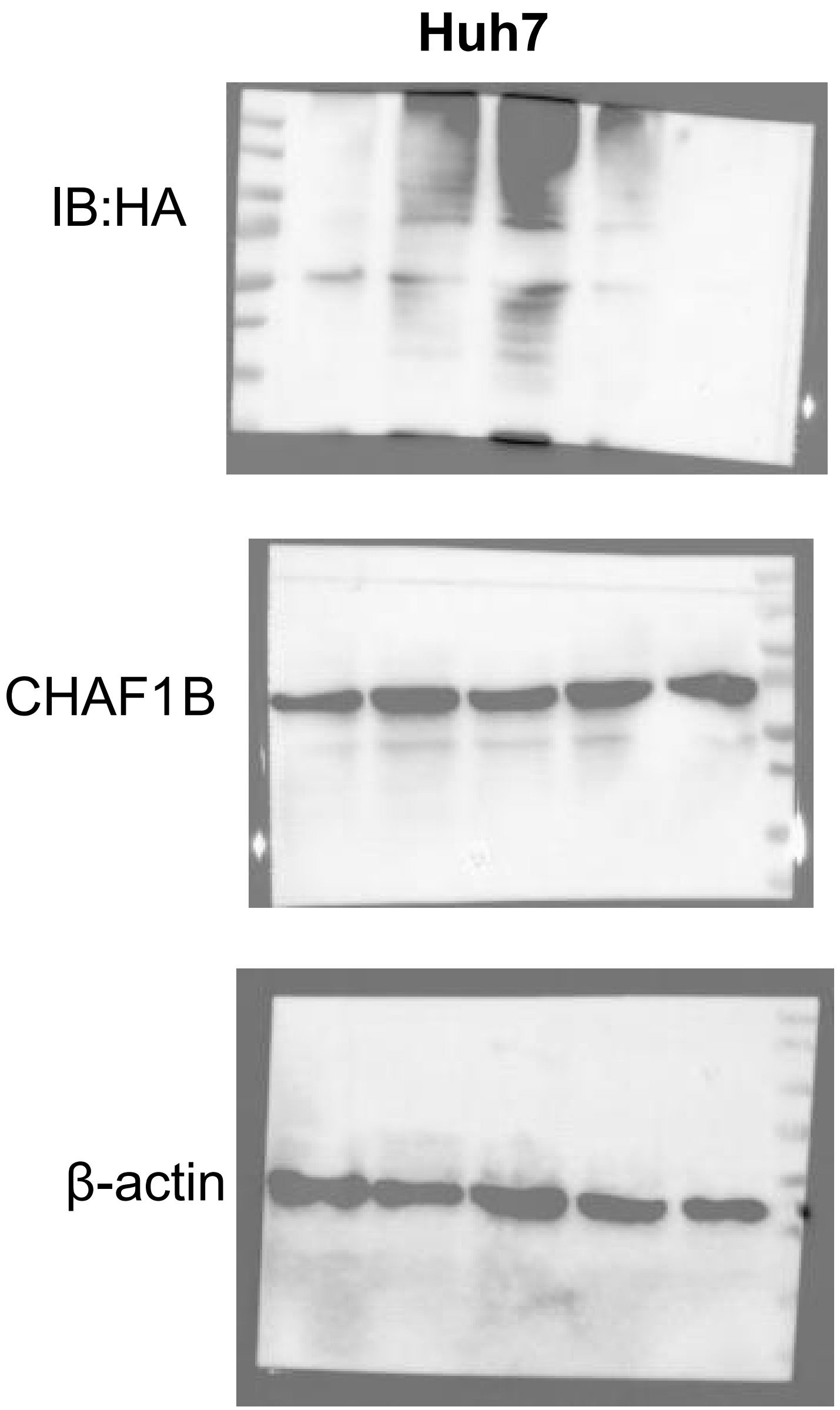

Fig. 8H

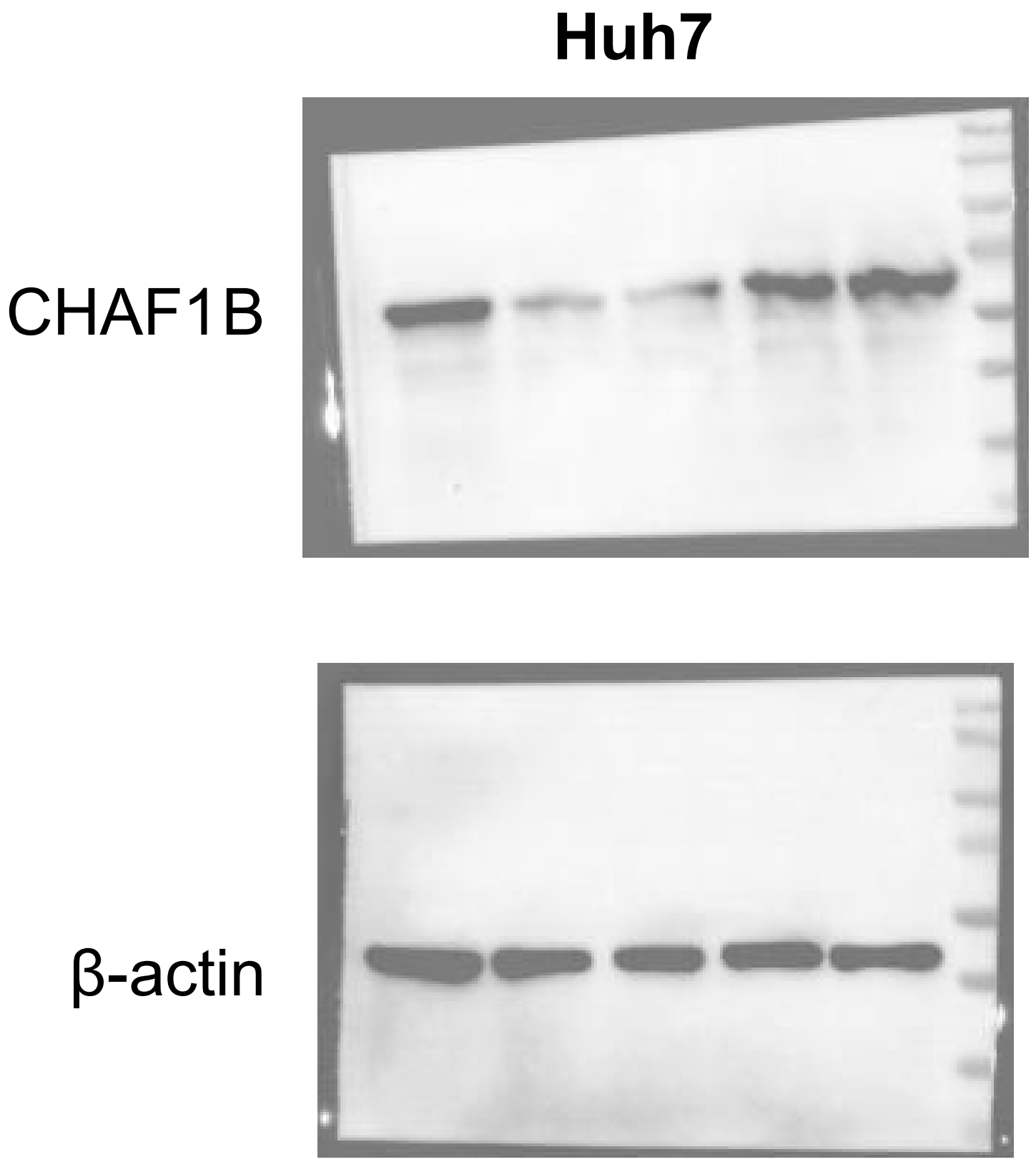

Fig.S1F

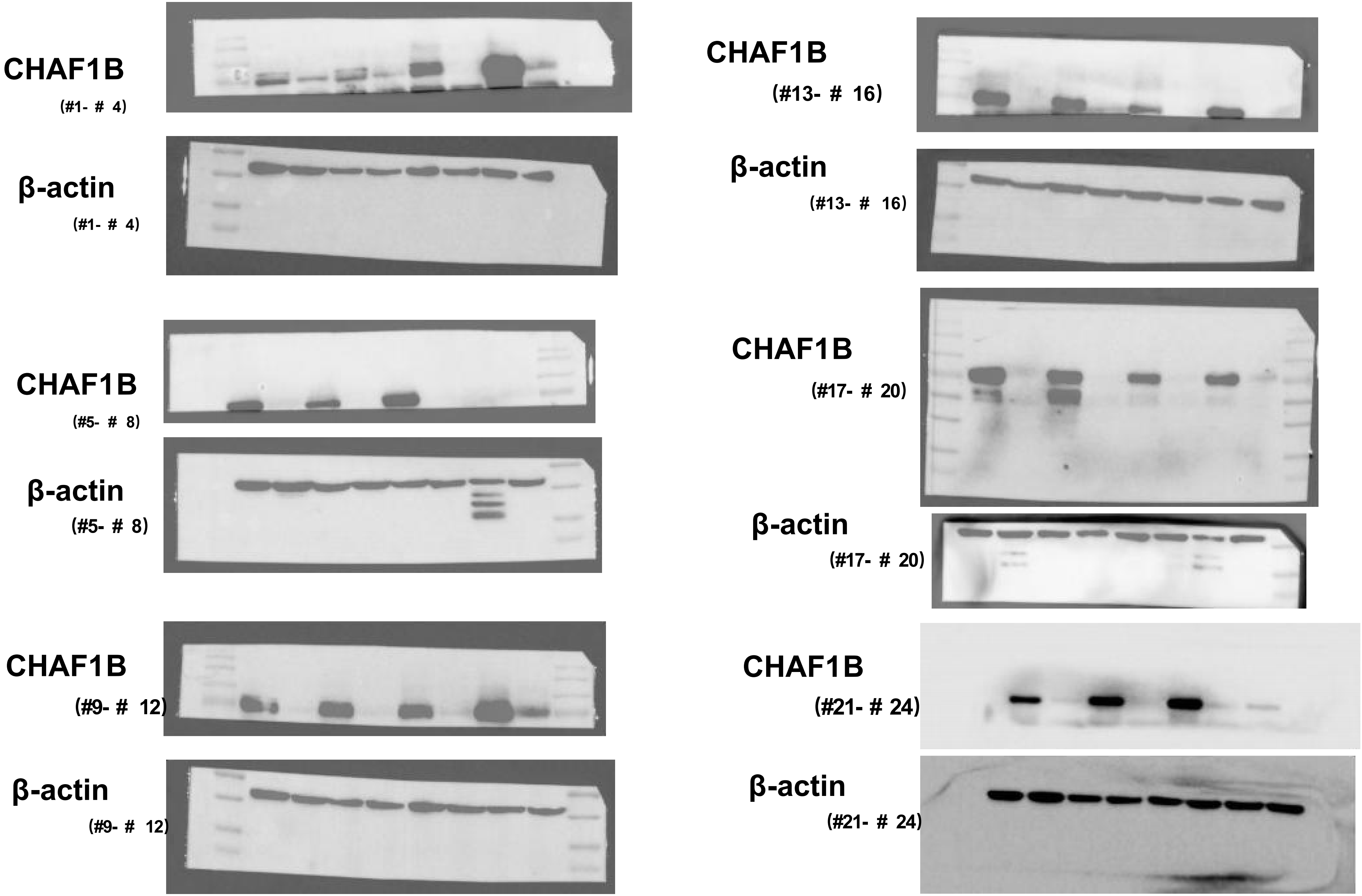
CHAF1B
(#1- # 4)
β-actin
(#1- # 4)
CHAF1B
(#5- # 8)
β-actin
(#5- # 8)
CHAF1B
(#9- # 12)
β-actin
(#9- # 12)
CHAF1B
(#13- # 16)
β-actin
(#13- # 16)
CHAF1B
(#17- # 20)
β-actin
(#17- # 20)
CHAF1B
(#21- # 24)
β-actin
(#21- # 24)

Fig.S2A

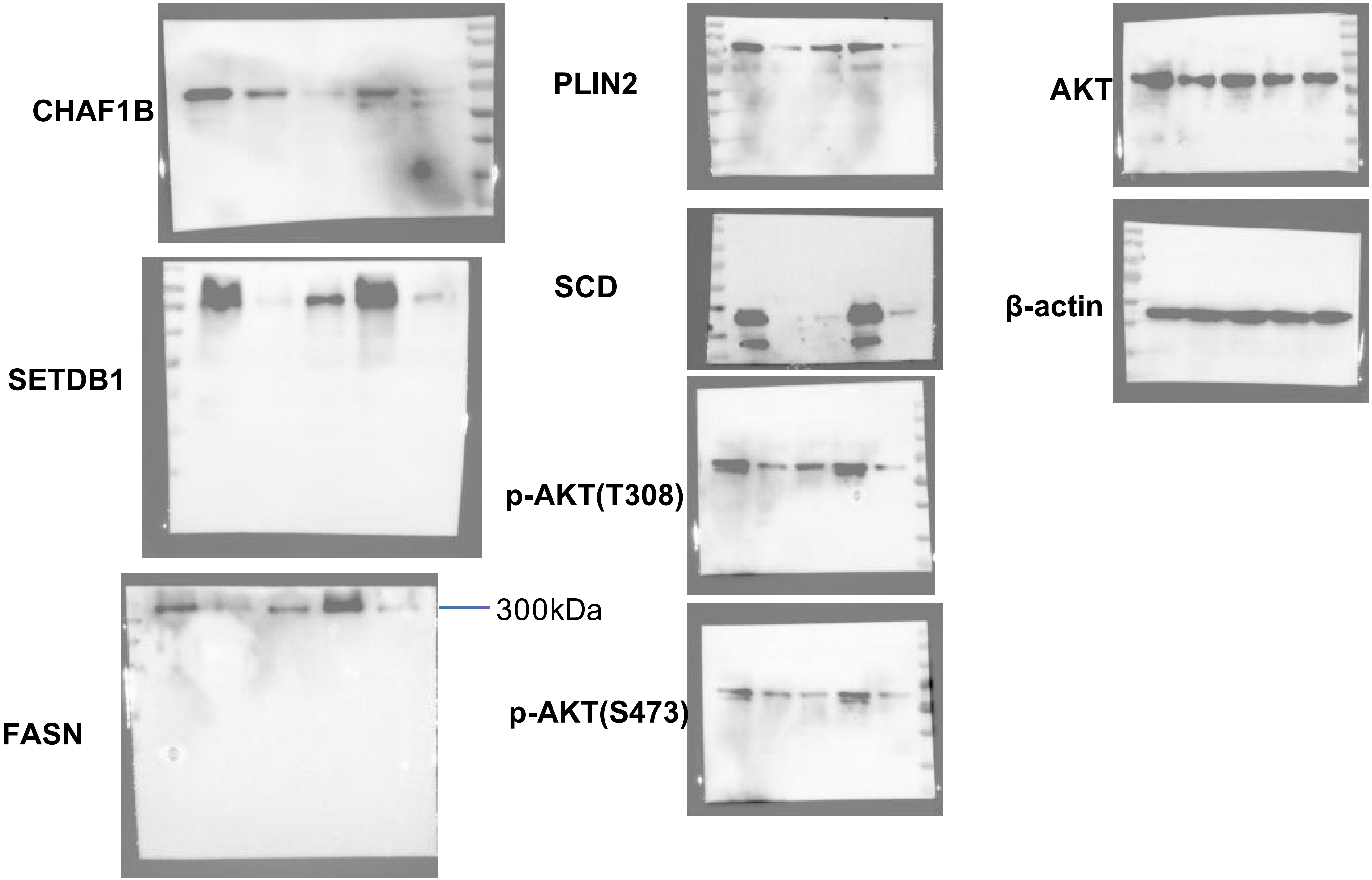
CHAF1B
PLIN2
AKT
SETDB1
SCD
β-actin
p-AKT(T308)
FASN
300kDa
p-AKT(S473)

Fig. S2F

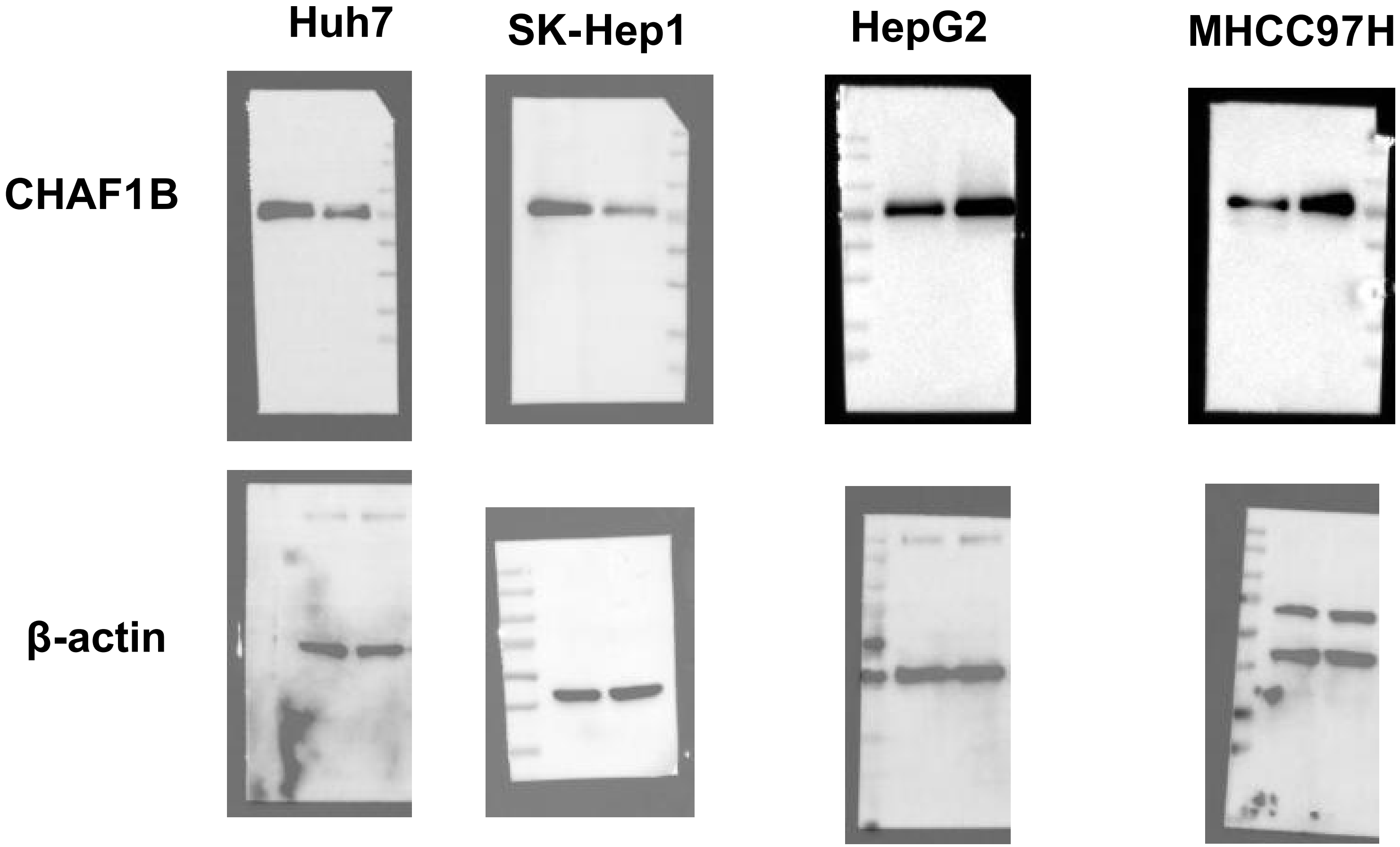

Fig.S4A HepG2

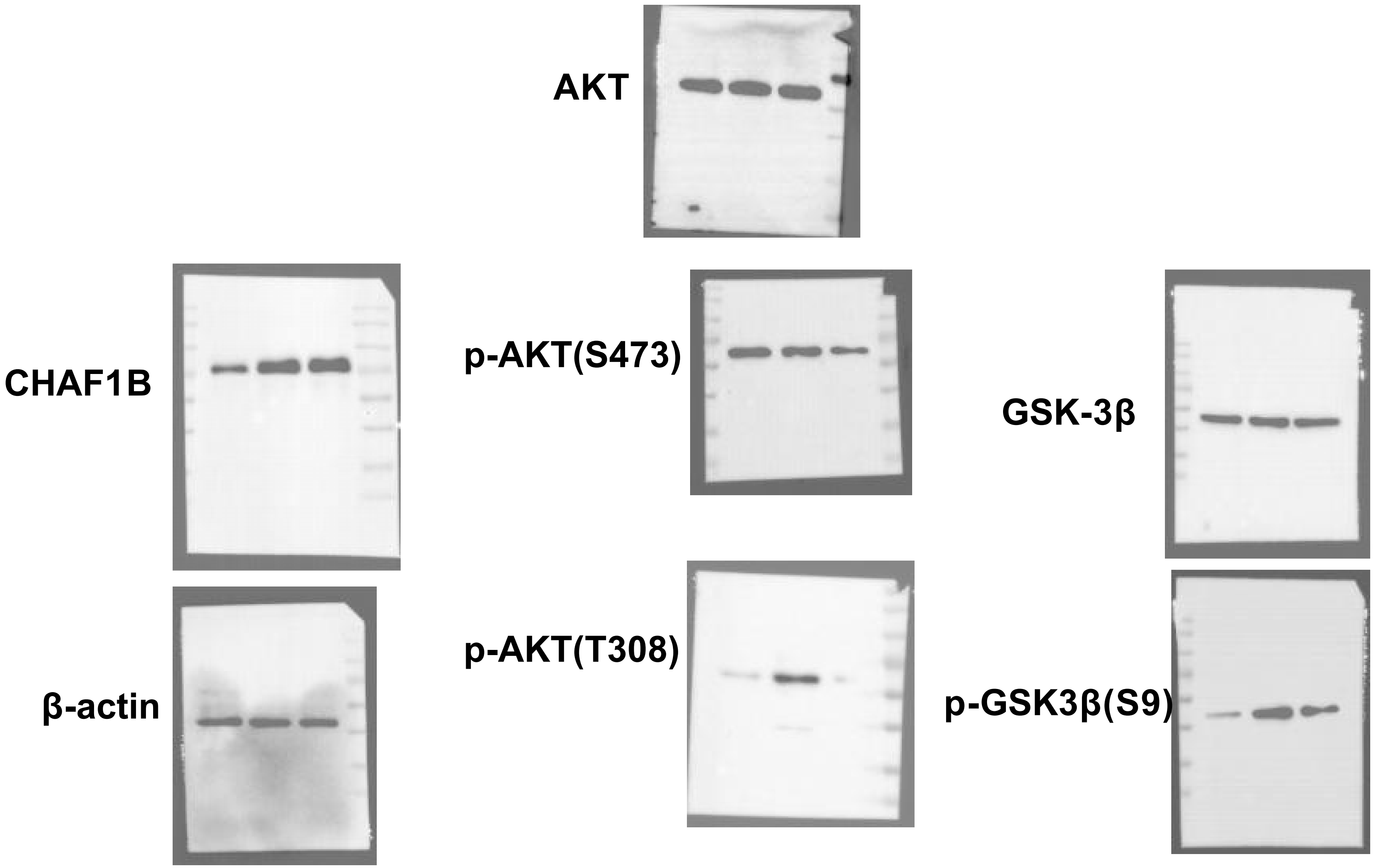

Fig.S4A MHCC97H

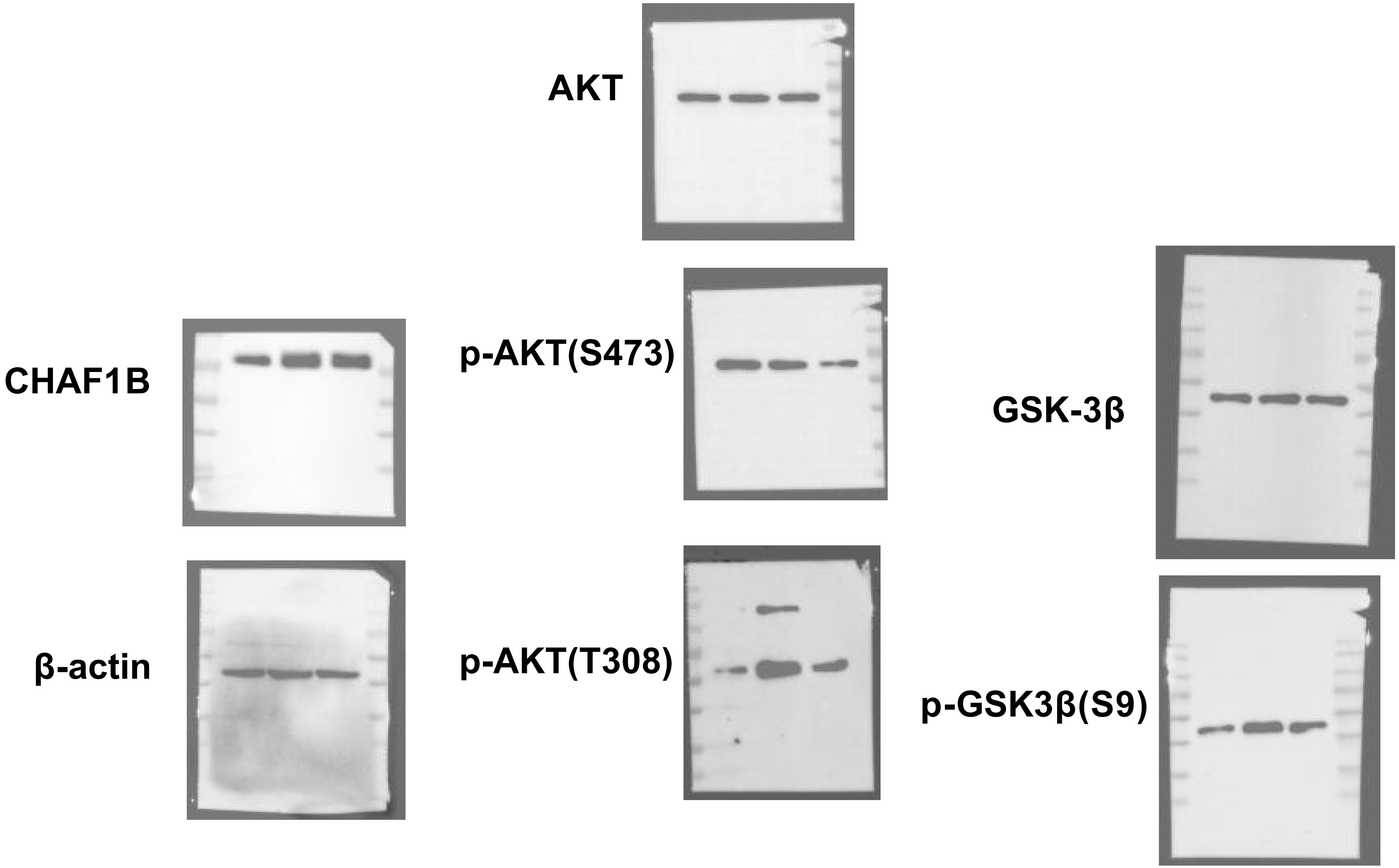

Fig.S5A

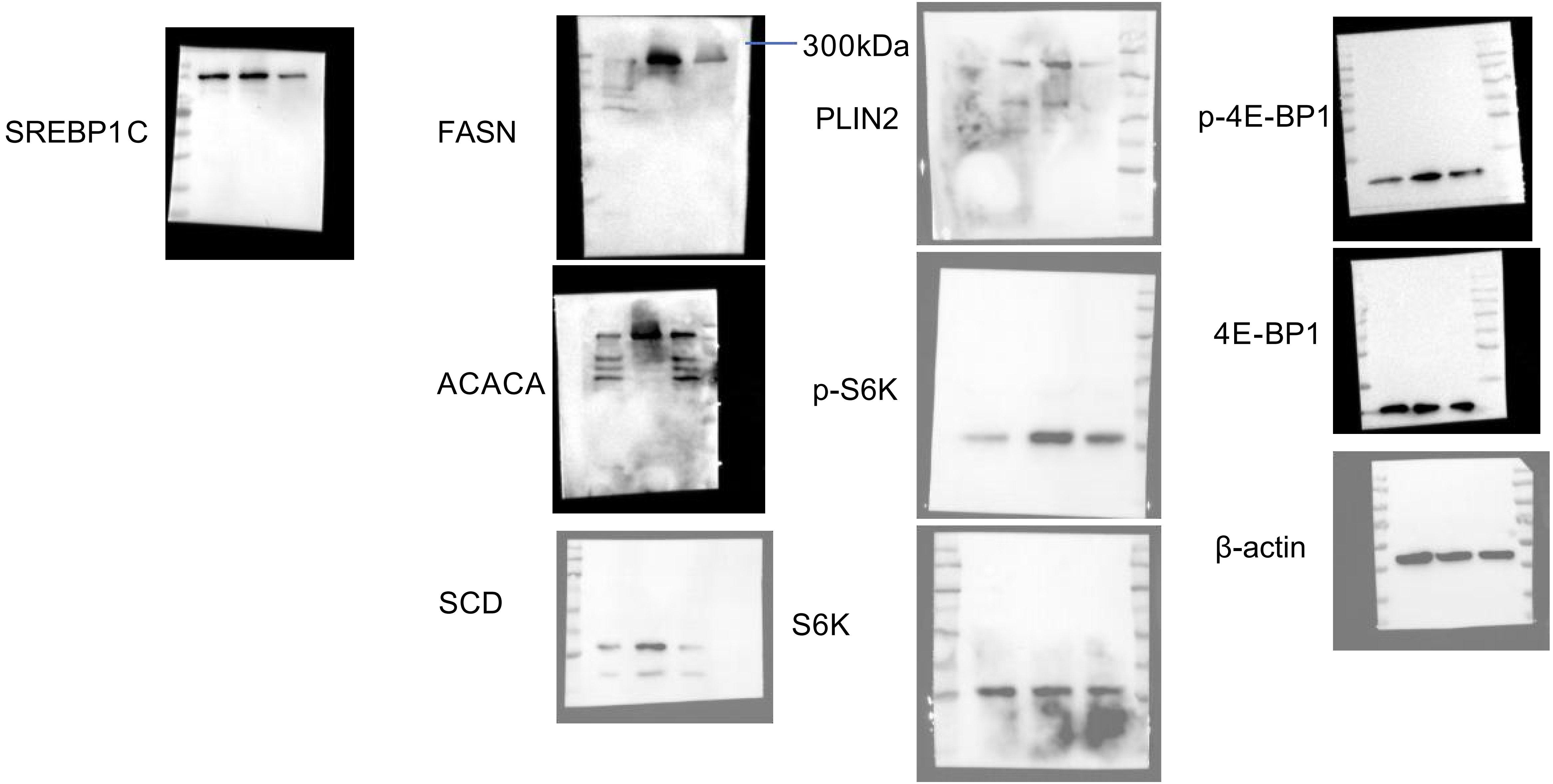
SREBP1C
FASN
300kDa
PLIN2
p-4E-BP1
ACACA
p-S6K
4E-BP1
β-actin
SCD
S6K

Fig.S6B

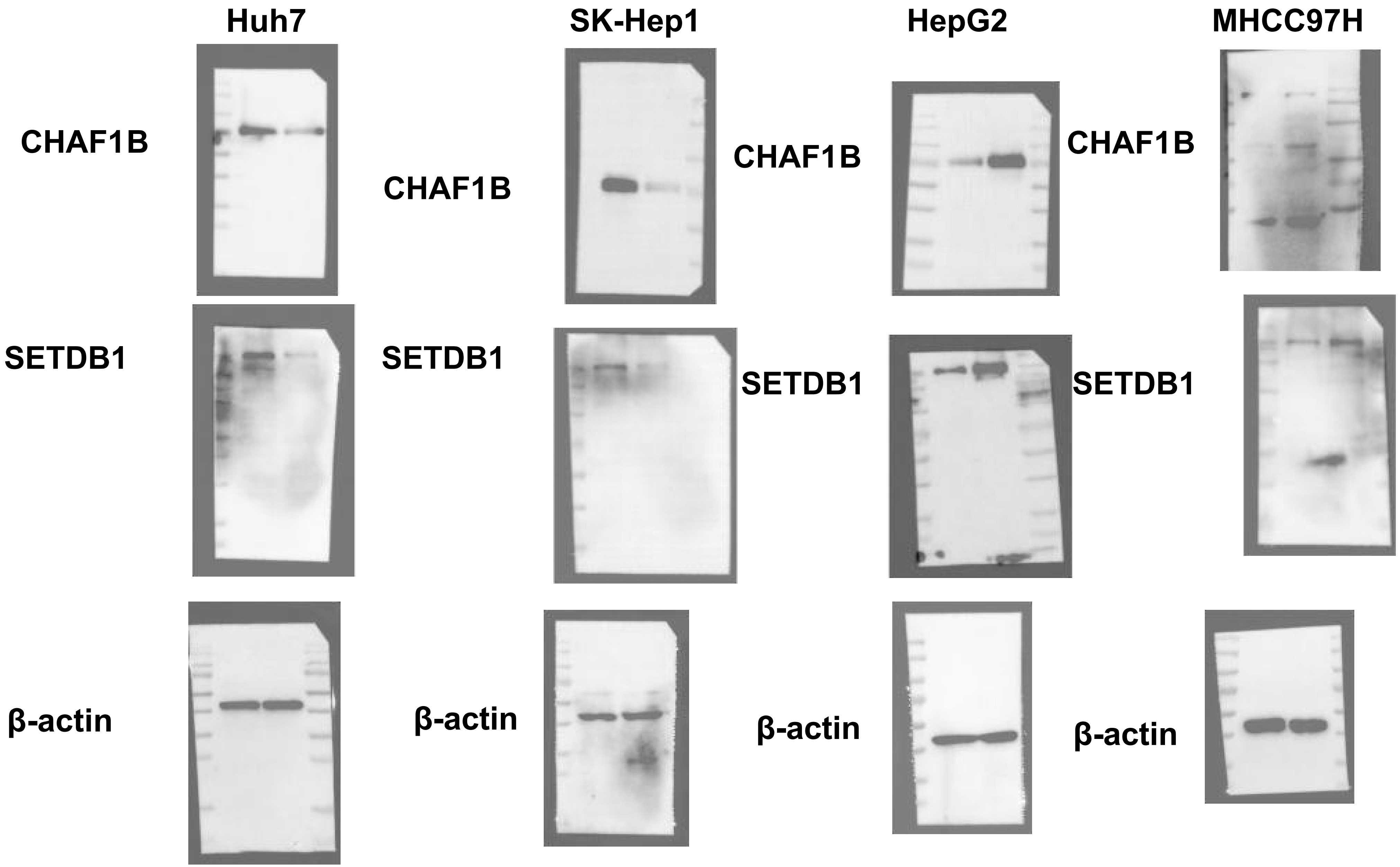
Huh7
SK-Hep1
HepG2
MHCC97H
CHAF1B
CHAF1B
CHAF1B
CHAF1B
SETDB1
SETDB1
SETDB1
SETDB1
β-actin
β-actin
β-actin
β-actin

Fig.S6D

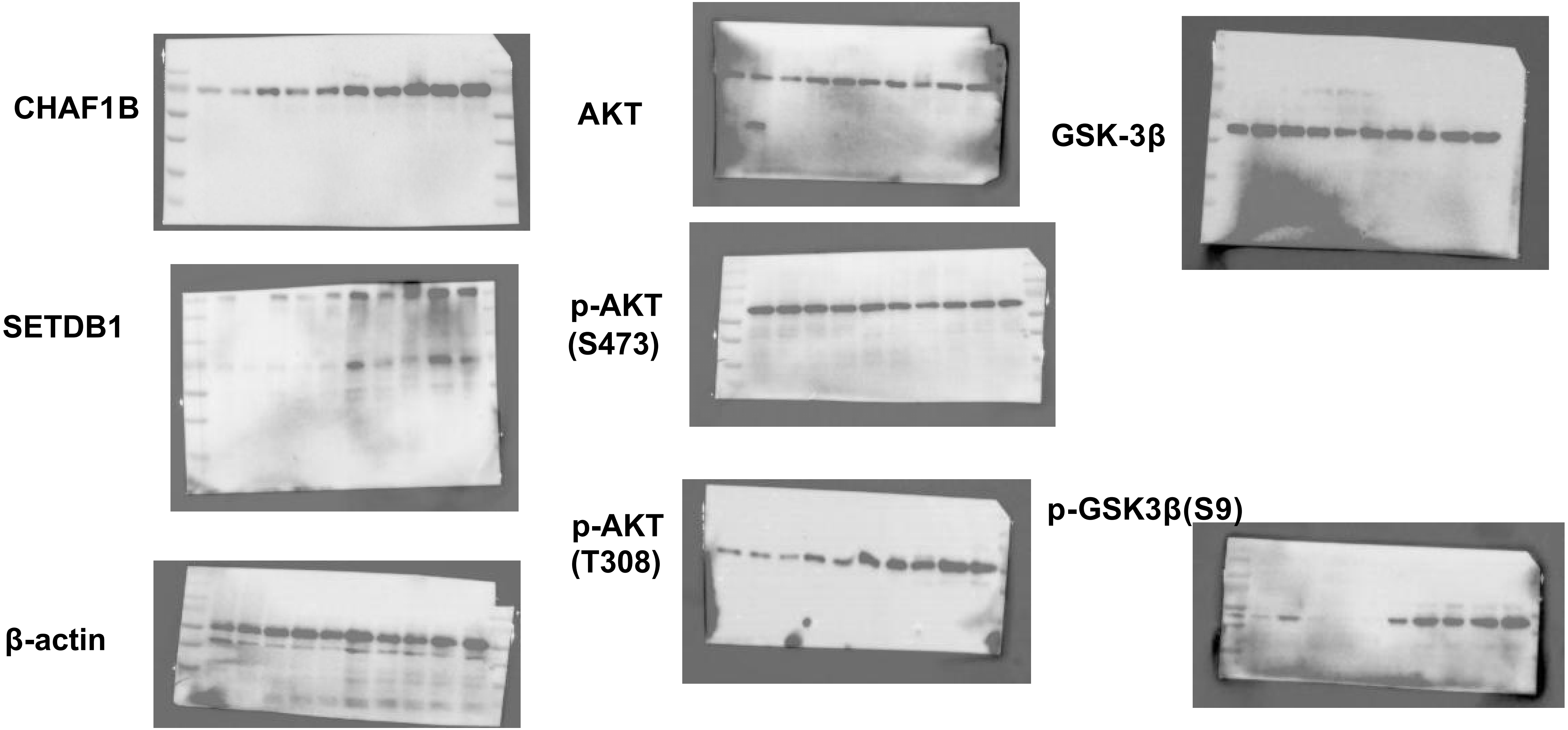
CHAF1B
AKT
GSK-3β
SETDB1
p-AKT
(S473)
p-AKT
(T308)
p-GSK3β(S9)
β-actin

Fig.S7A

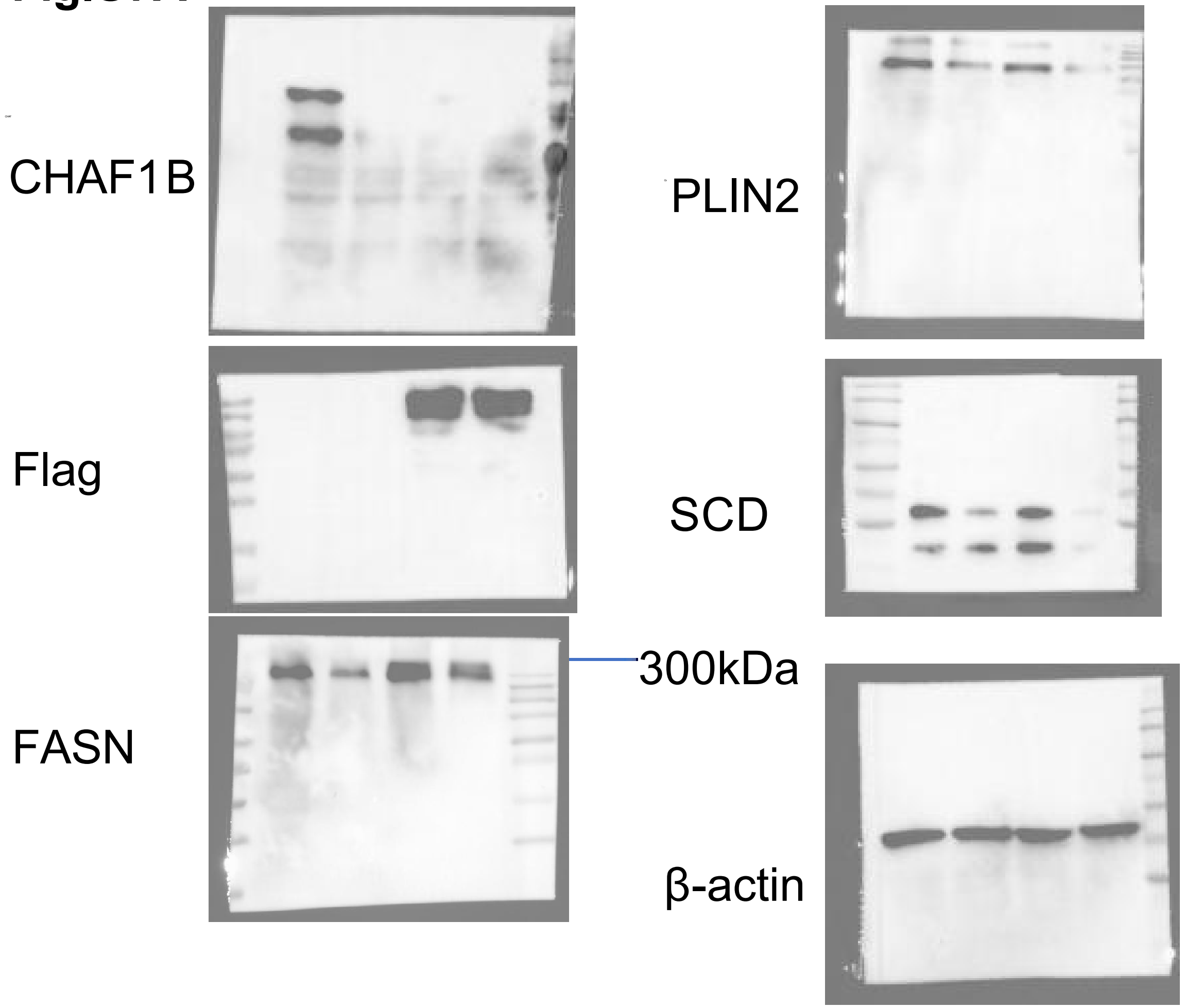

Fig.S8A

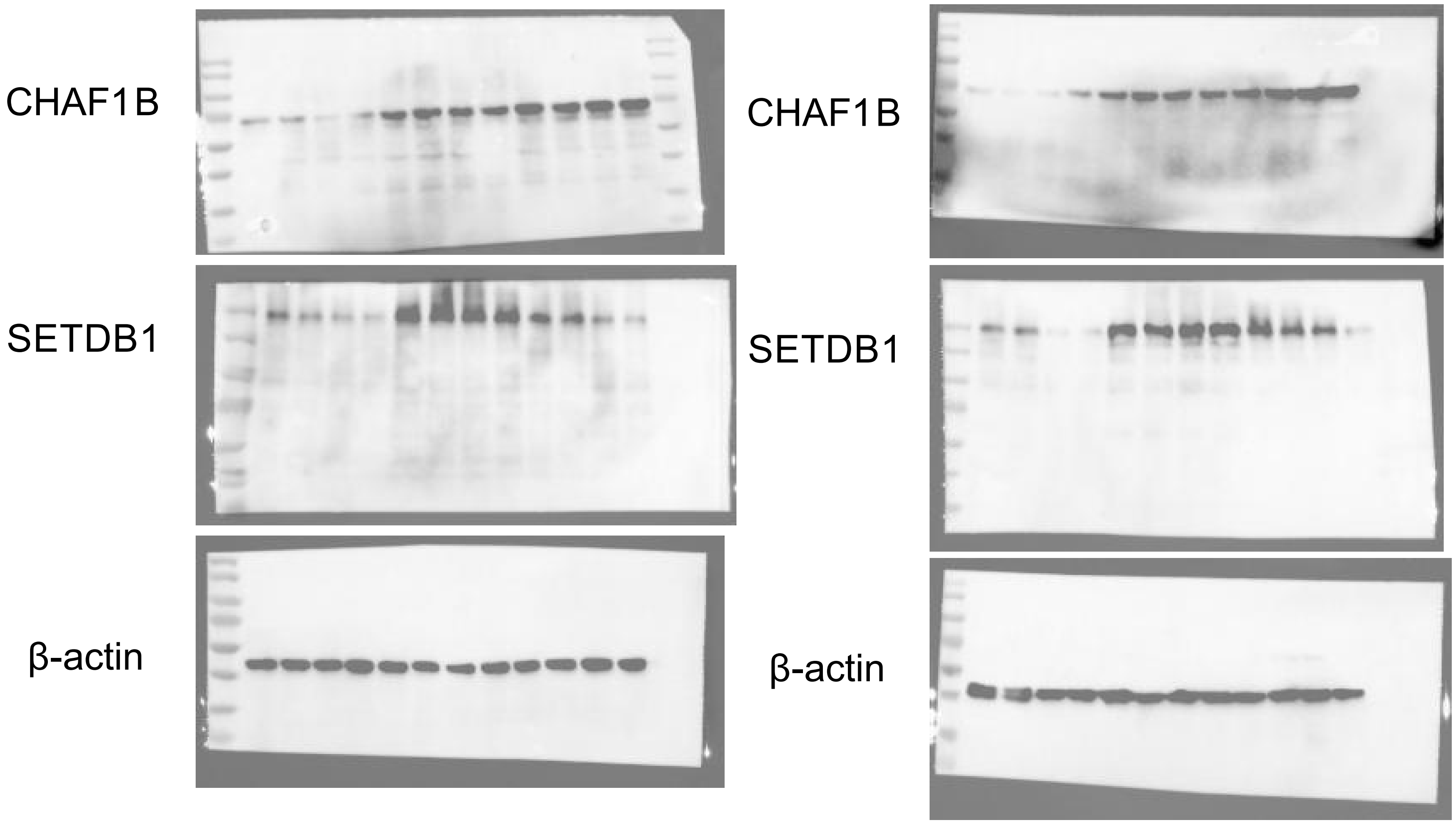

Fig.S8B

IP:SETDB1
IB:HA

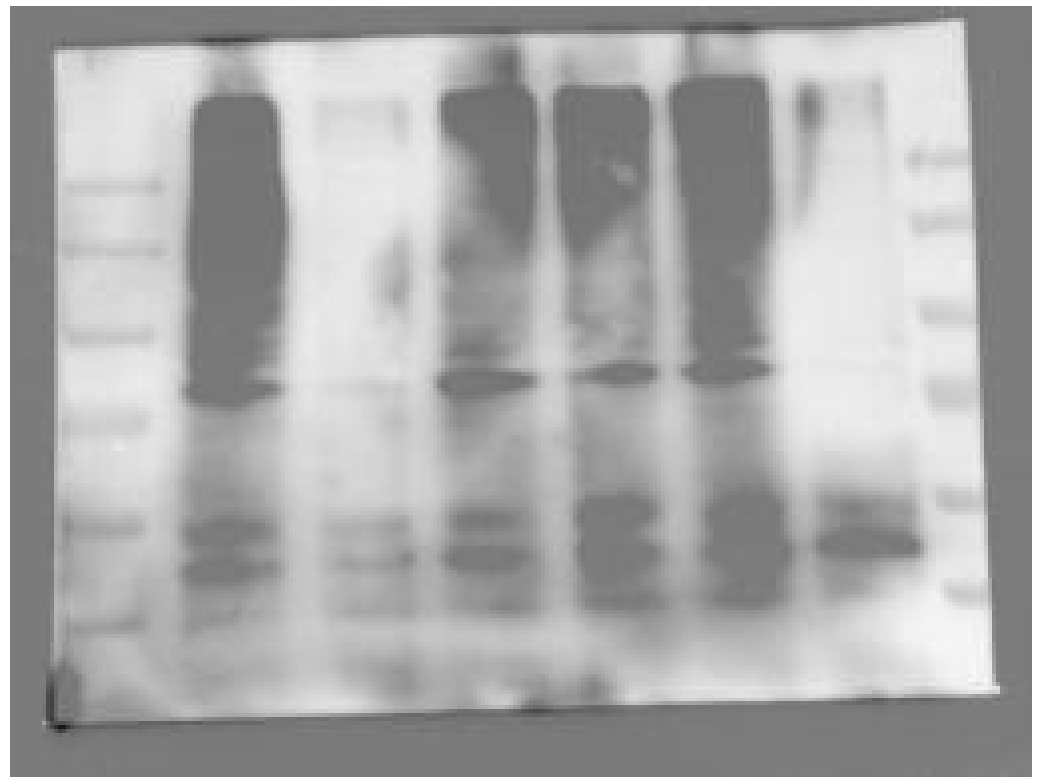

IP:SETDB1
IB:SETDB1

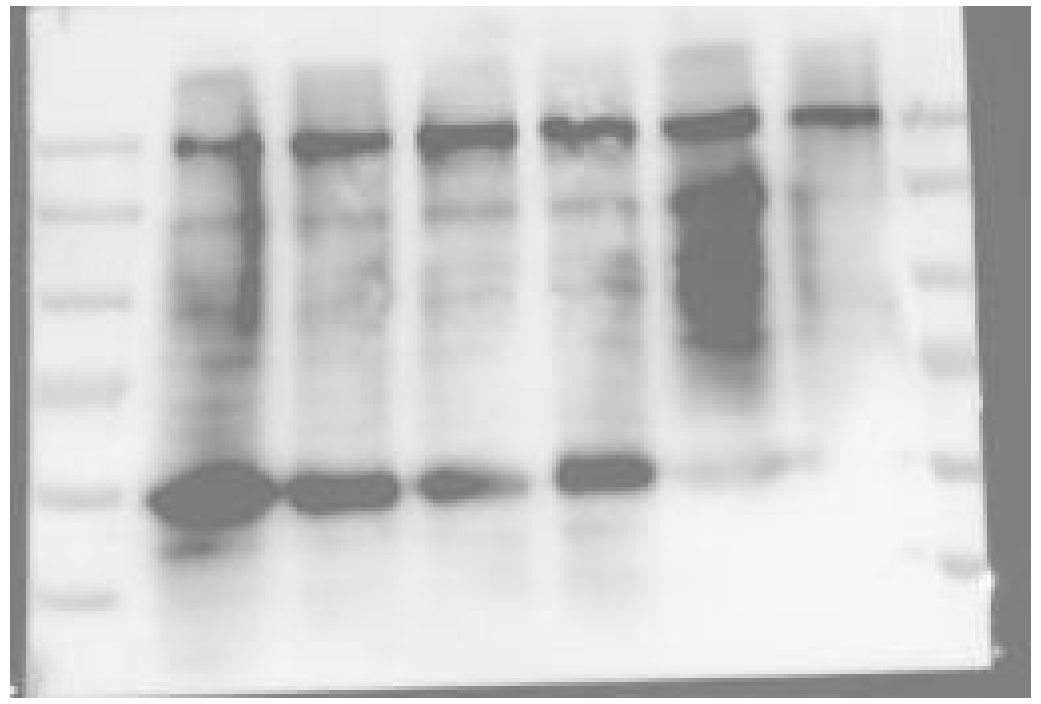

CHAF1B

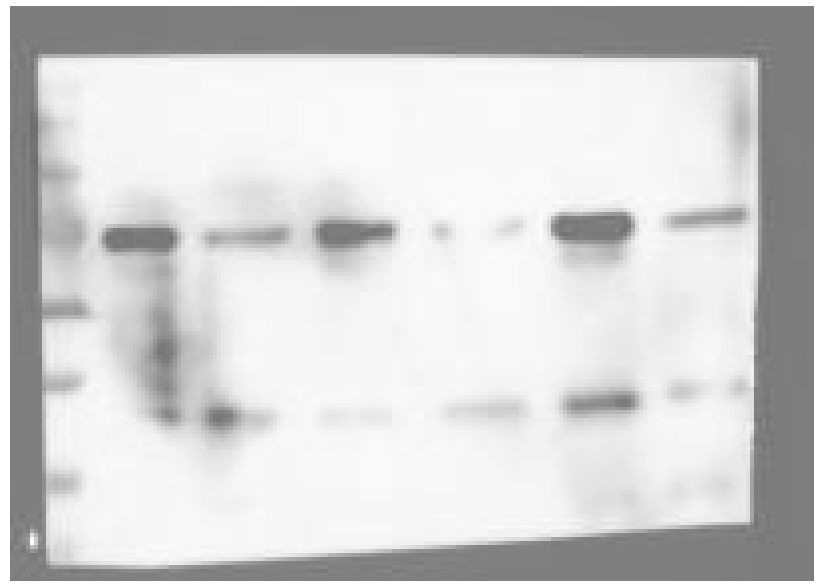

SETDB1

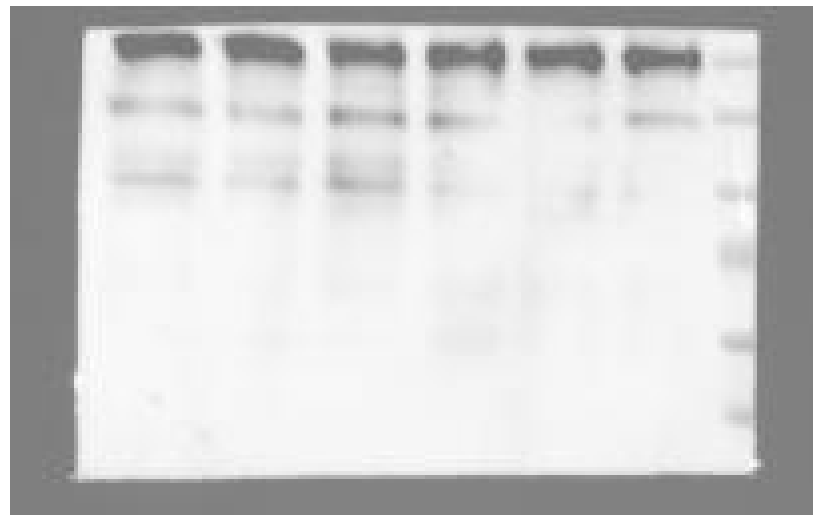

β-actin

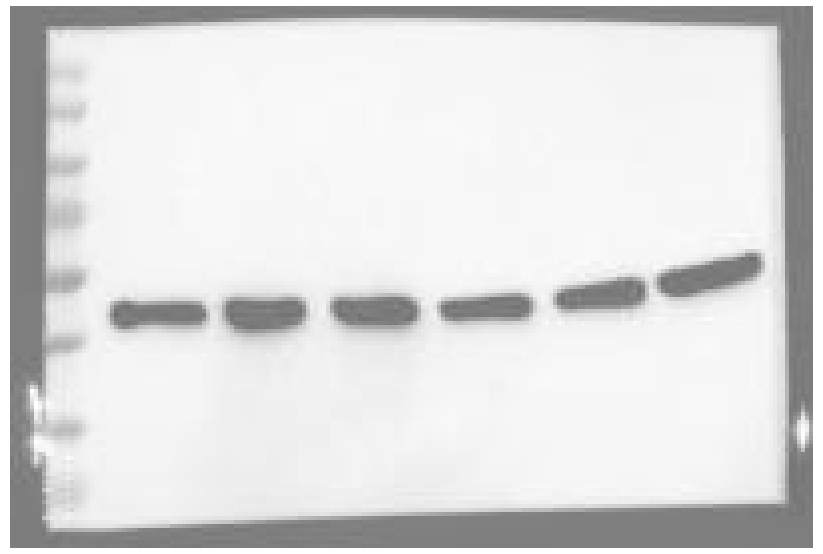

Fig.S8C

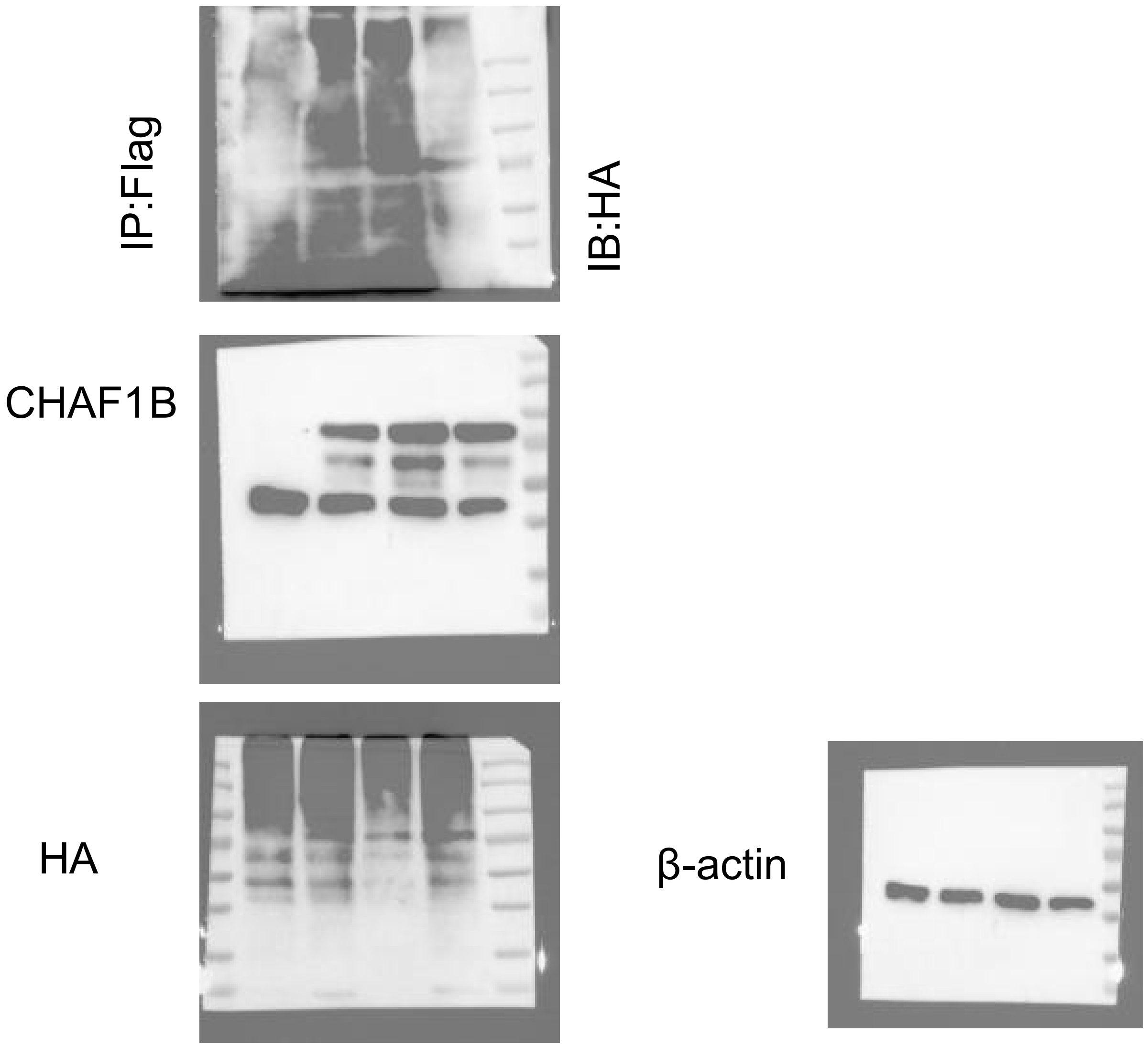

Fig.S8E

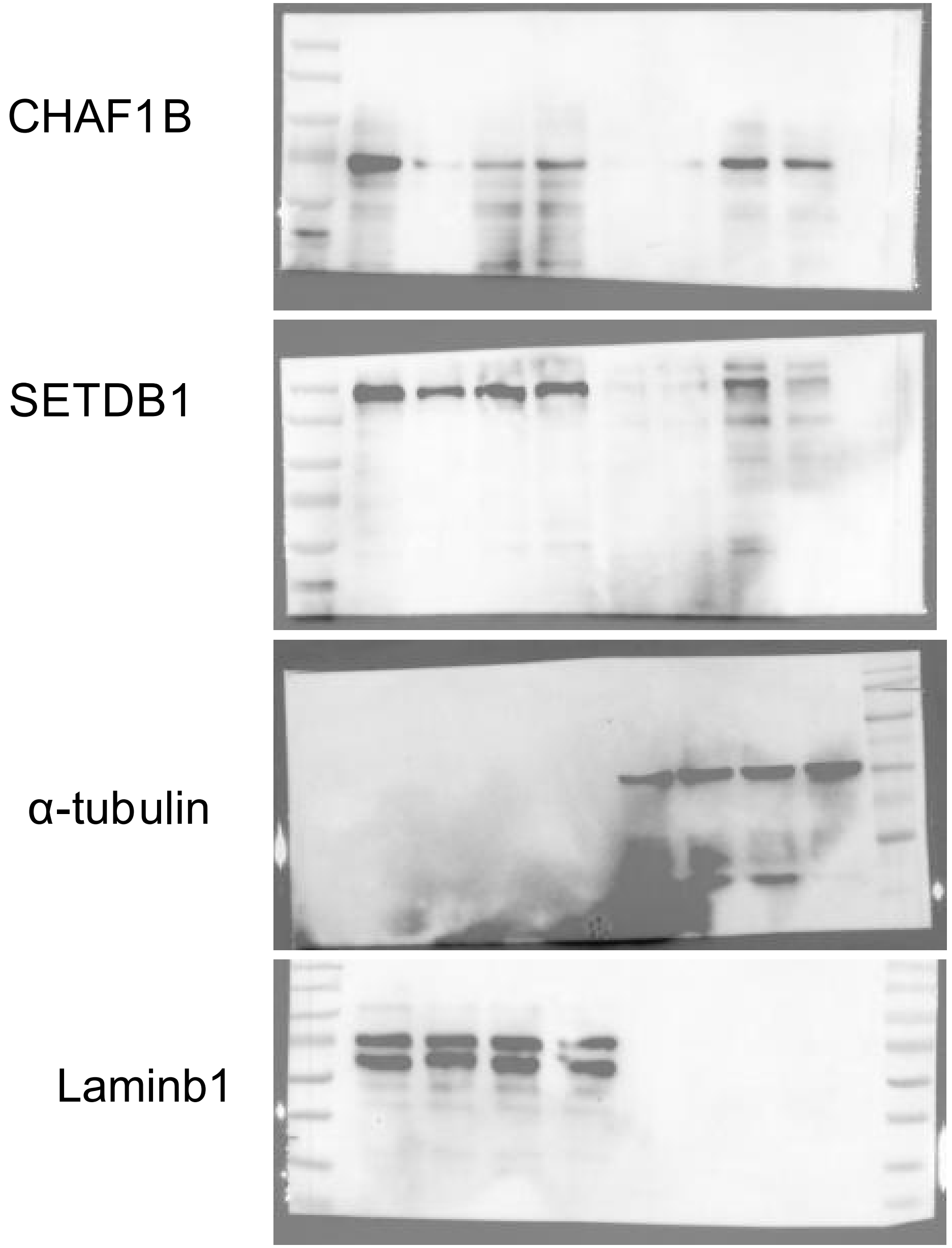

Fig.S8F

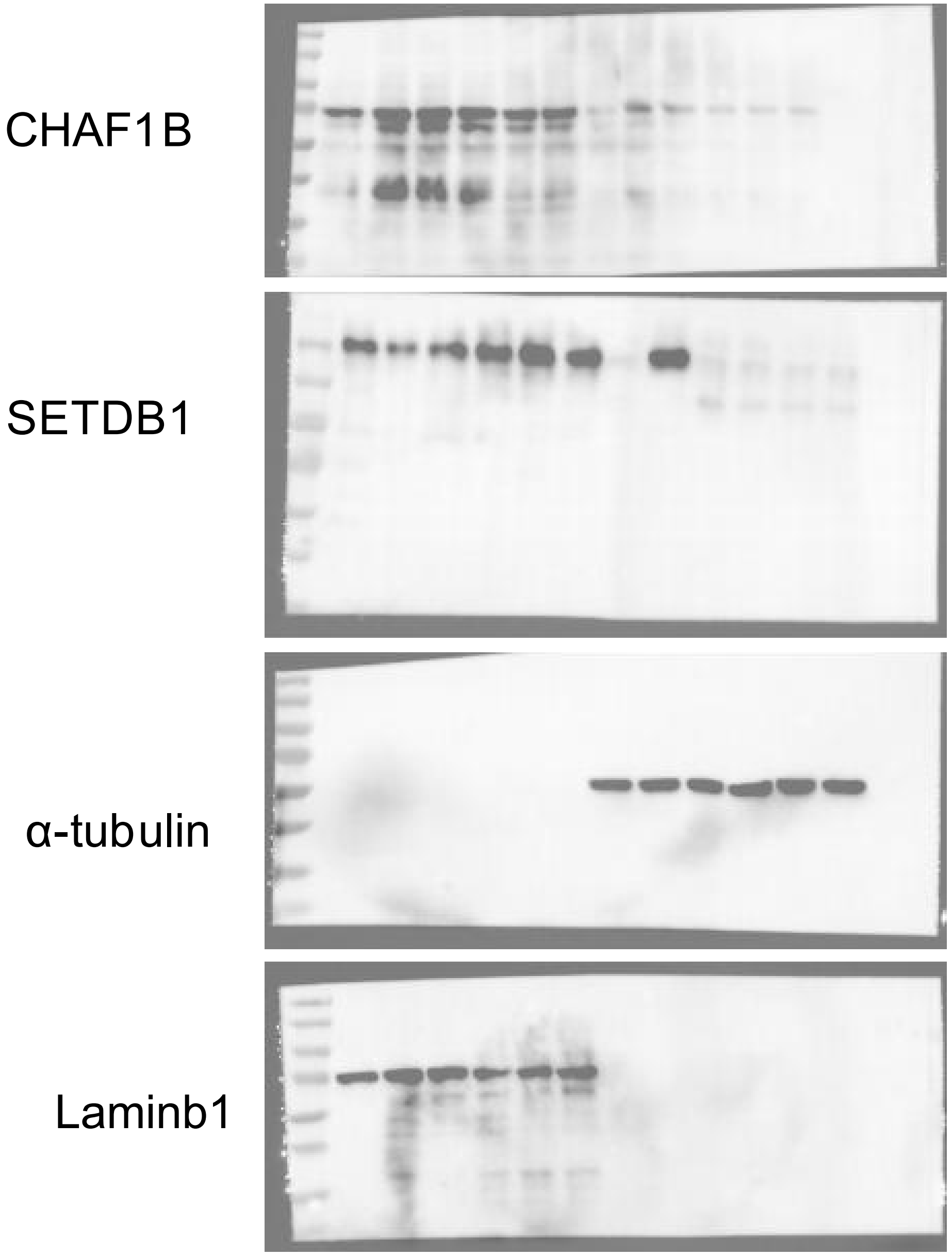

Fig. S9B

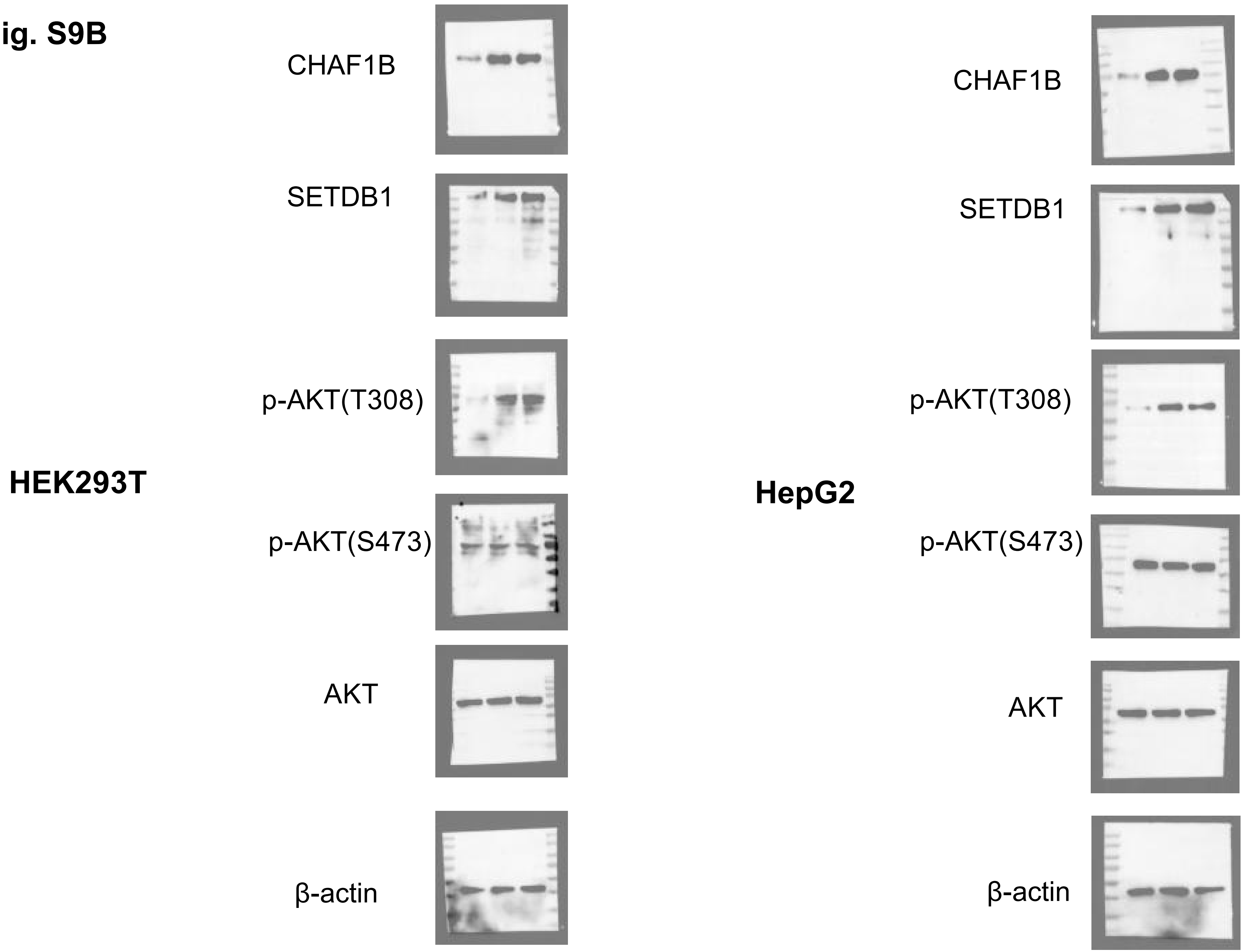
HEK293T
CHAF1B
SETDB1
p-AKT(T308)
p-AKT(S473)
AKT
β-actin
HepG2
CHAF1B
SETDB1
p-AKT(T308)
p-AKT(S473)
AKT
β-actin

Fig. S9C

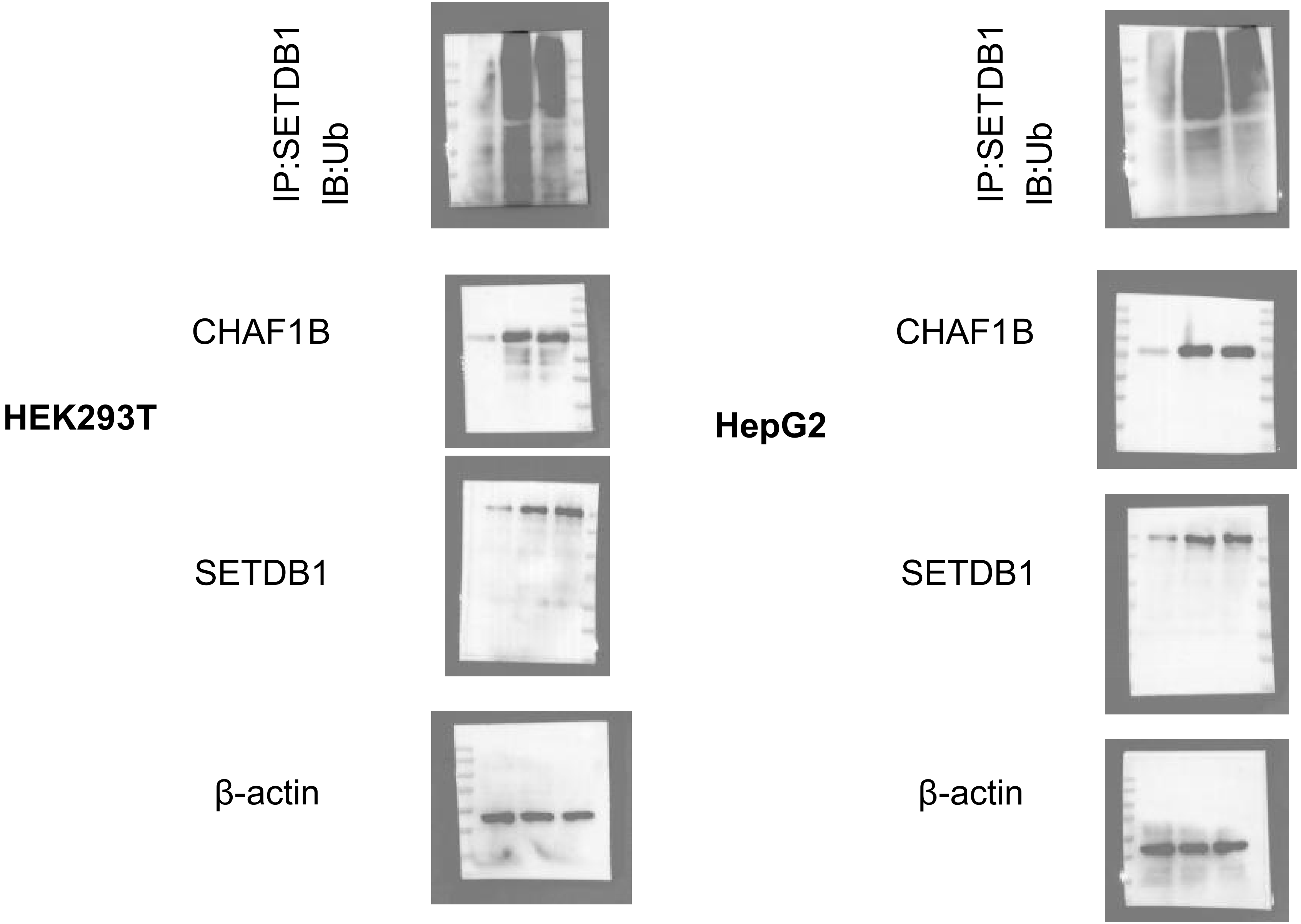
IP:SETDB1
IB:Ub
CHAF1B
SETDB1
β-actin
HEK293T
IP:SETDB1
IB:Ub
CHAF1B
SETDB1
β-actin
HepG2

Fig. S10A

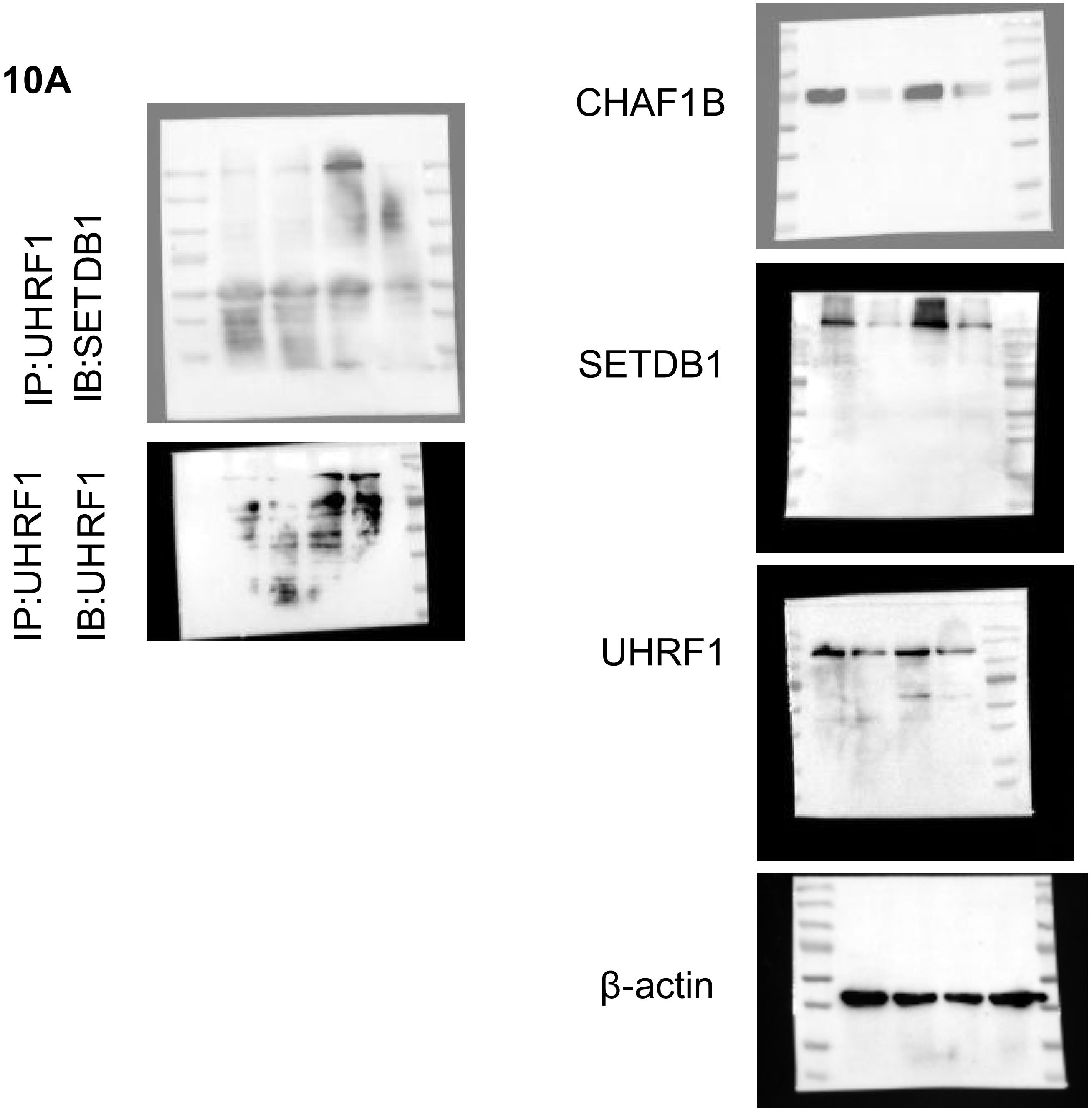

IP:UHRF1
IB:SETDB1
IP:UHRF1
IB:UHRF1
CHAF1B
SETDB1
UHRF1
β-actin

Fig. S10B

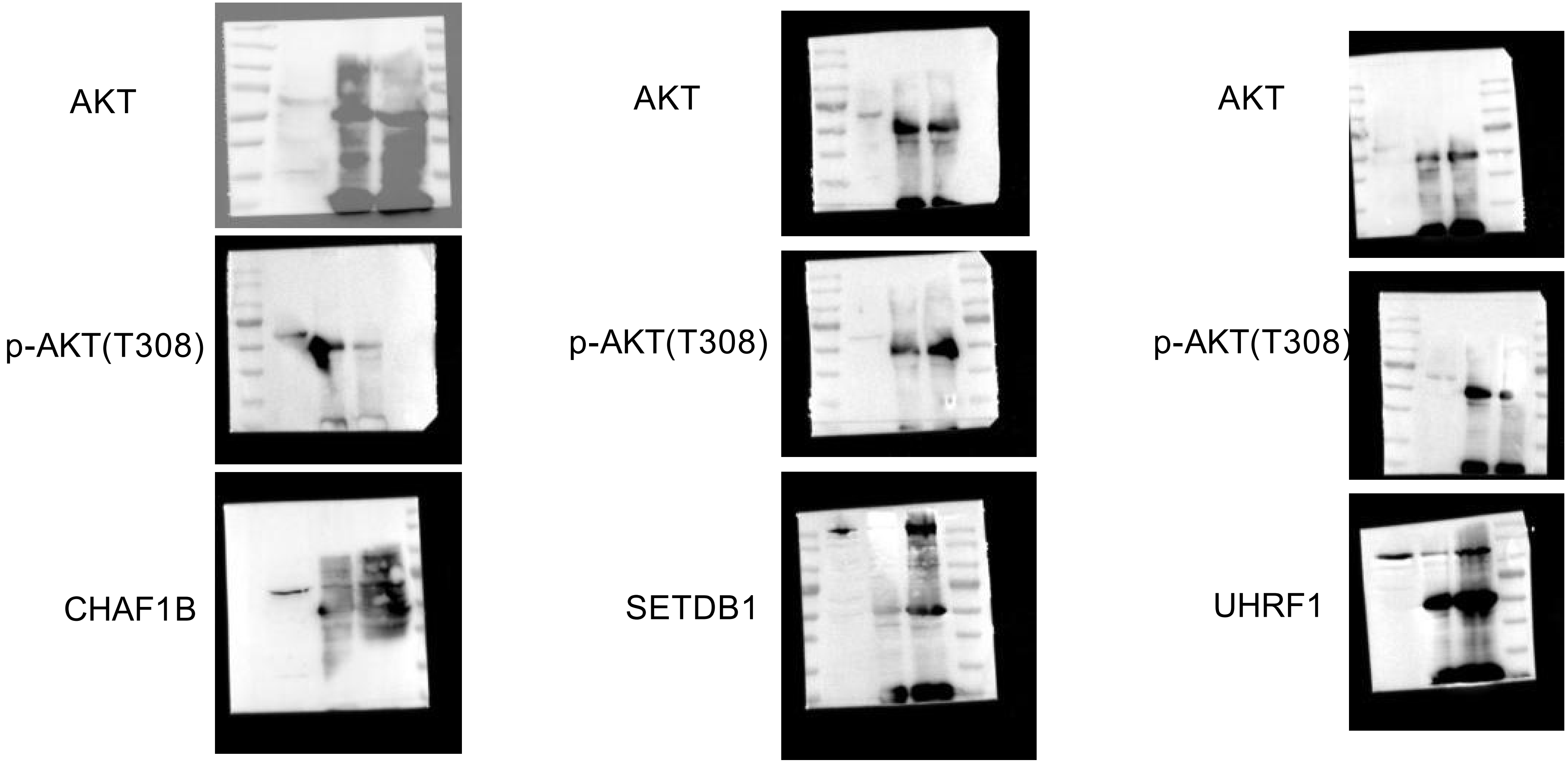
AKT
p-AKT(T308)
CHAF1B
AKT
p-AKT(T308)
SETDB1
AKT
p-AKT(T308)
UHRF1

Fig. S10C

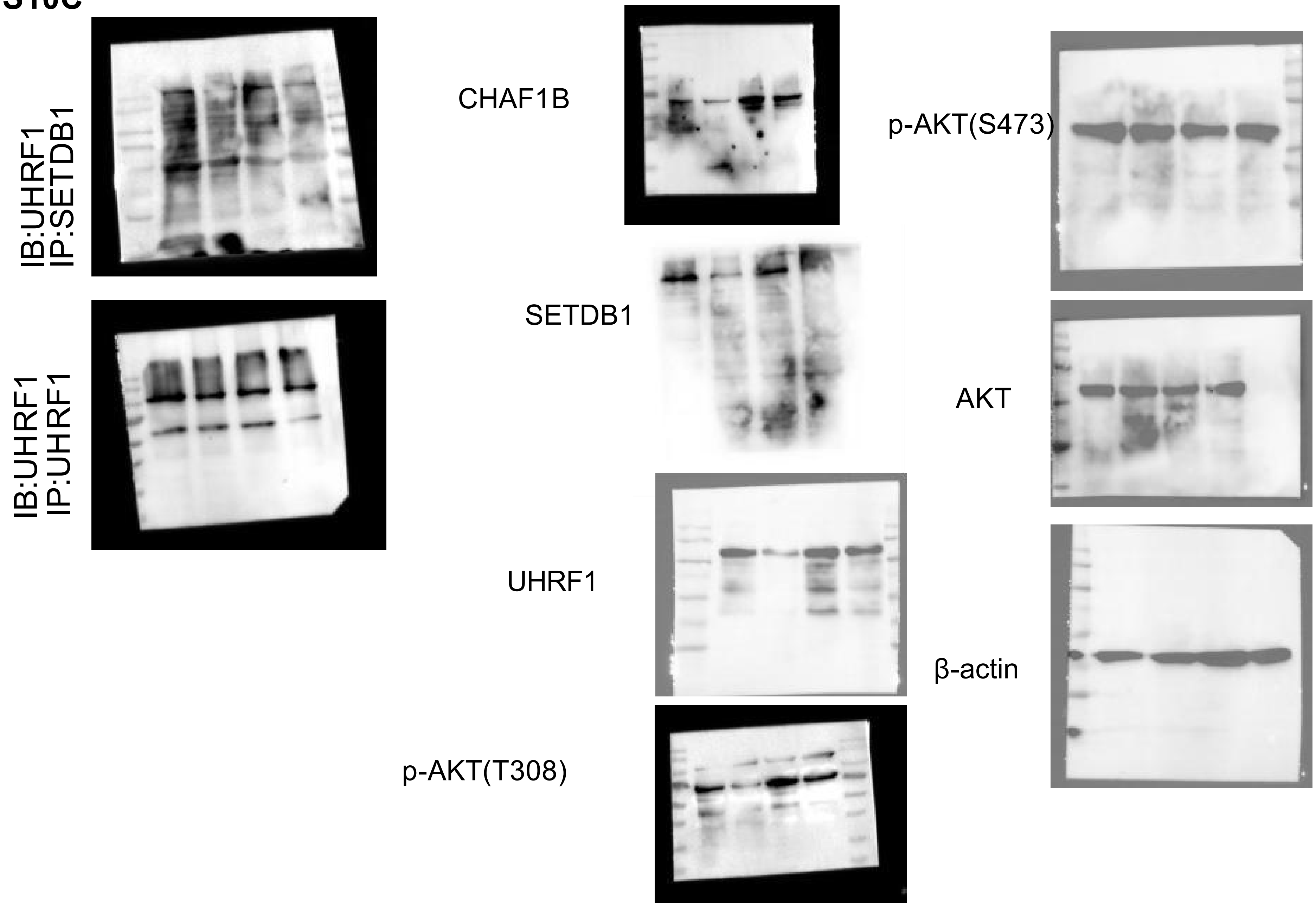
IP:SETDB1
IB:UHRF1
IP:UHRF1
IB:UHRF1
CHAF1B
SETDB1
UHRF1
p-AKT(T308)
p-AKT(S473)
AKT
β-actin

Fig. S10D

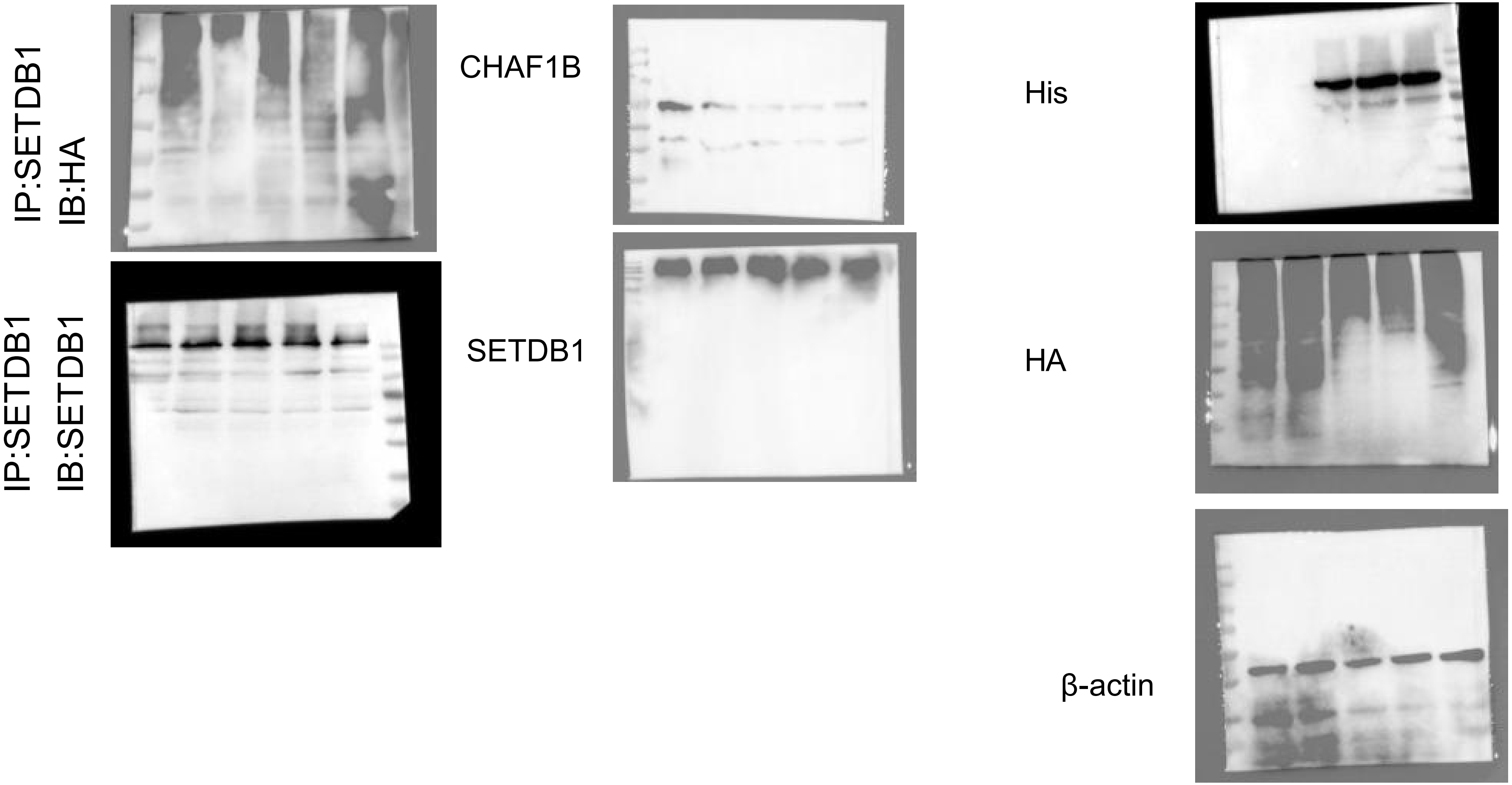
IP:SETDB1
IB:HA
IP:SETDB1
IB:SETDB1
CHAF1B
SETDB1
His
HA
β-actin

Fig. S10E

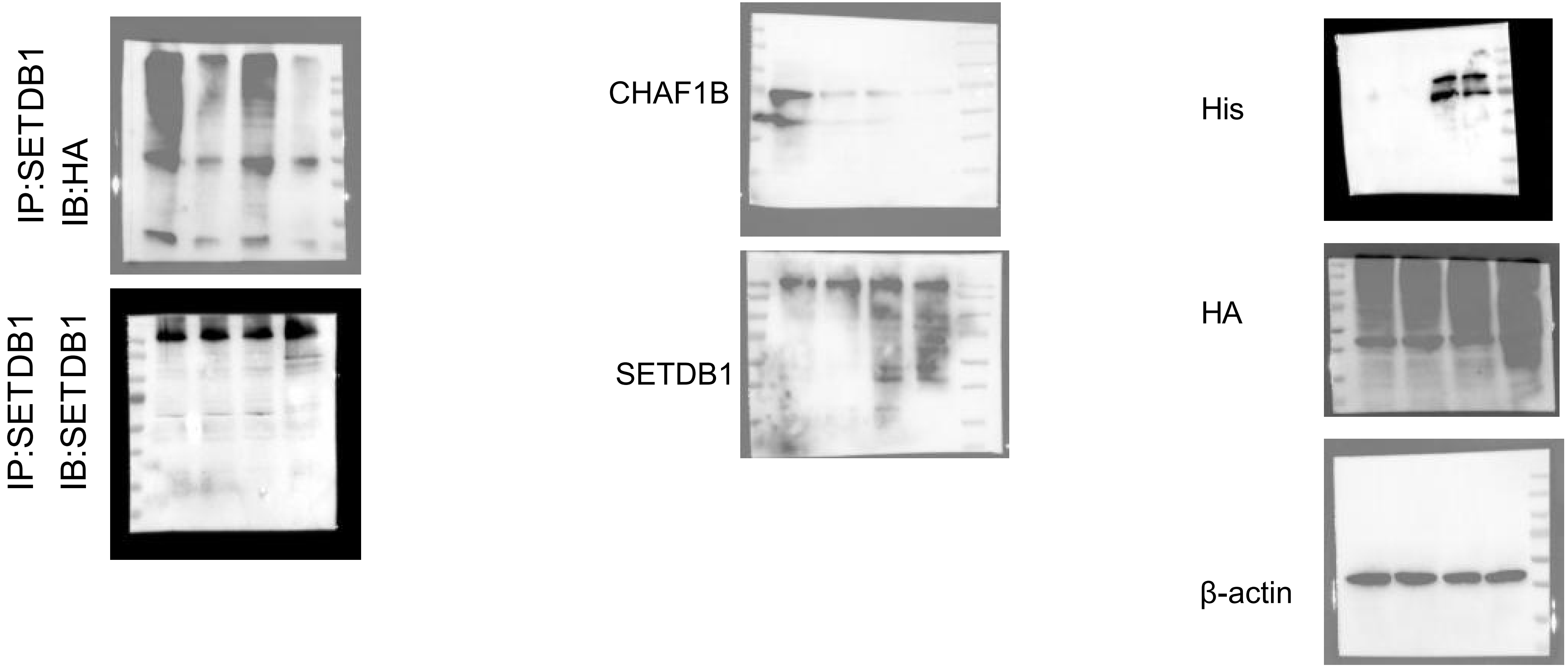
IP:SETDB1
IB:HA
IP:SETDB1
IB:SETDB1
CHAF1B
SETDB1
His
HA
β-actin

Fig S11A

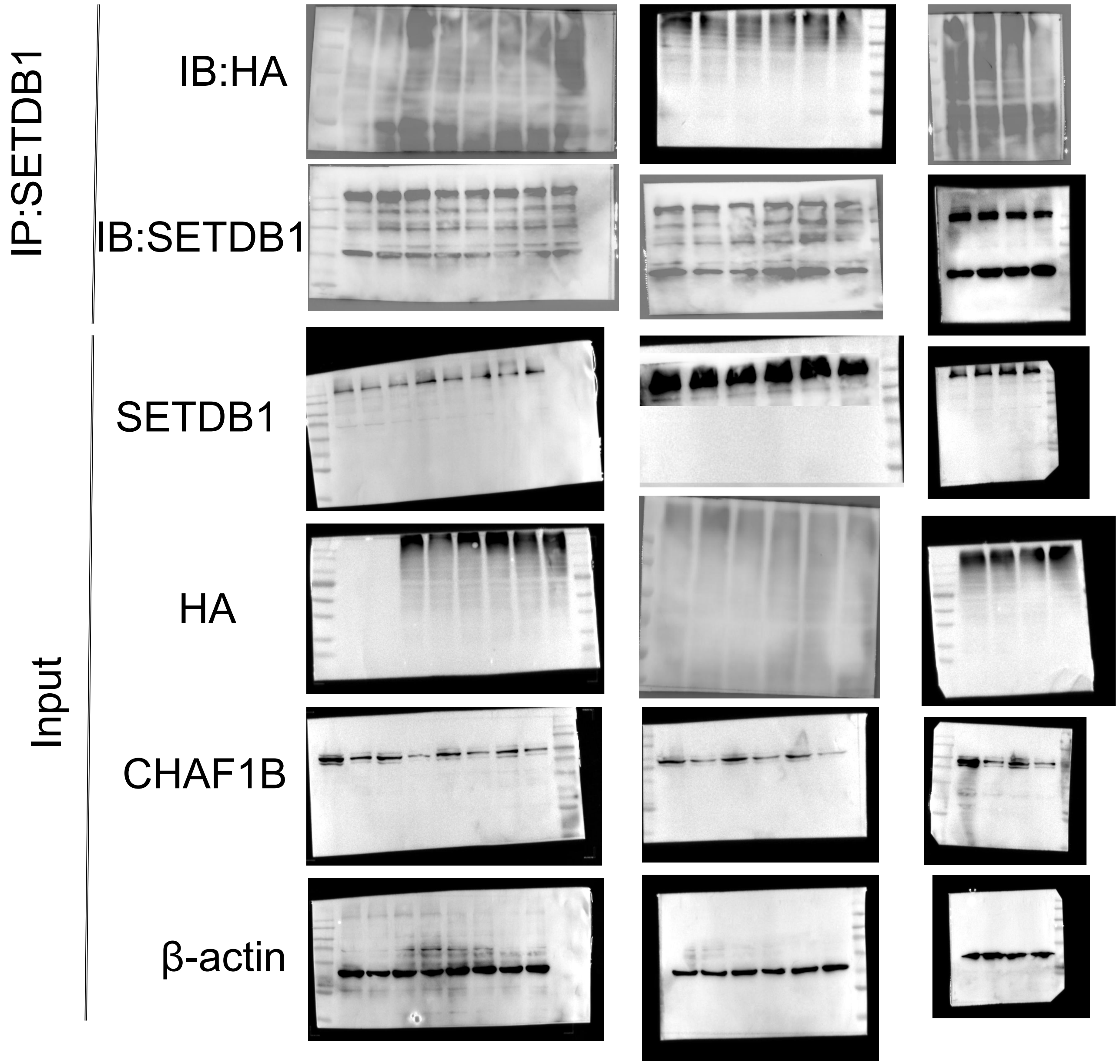

Fig S11B

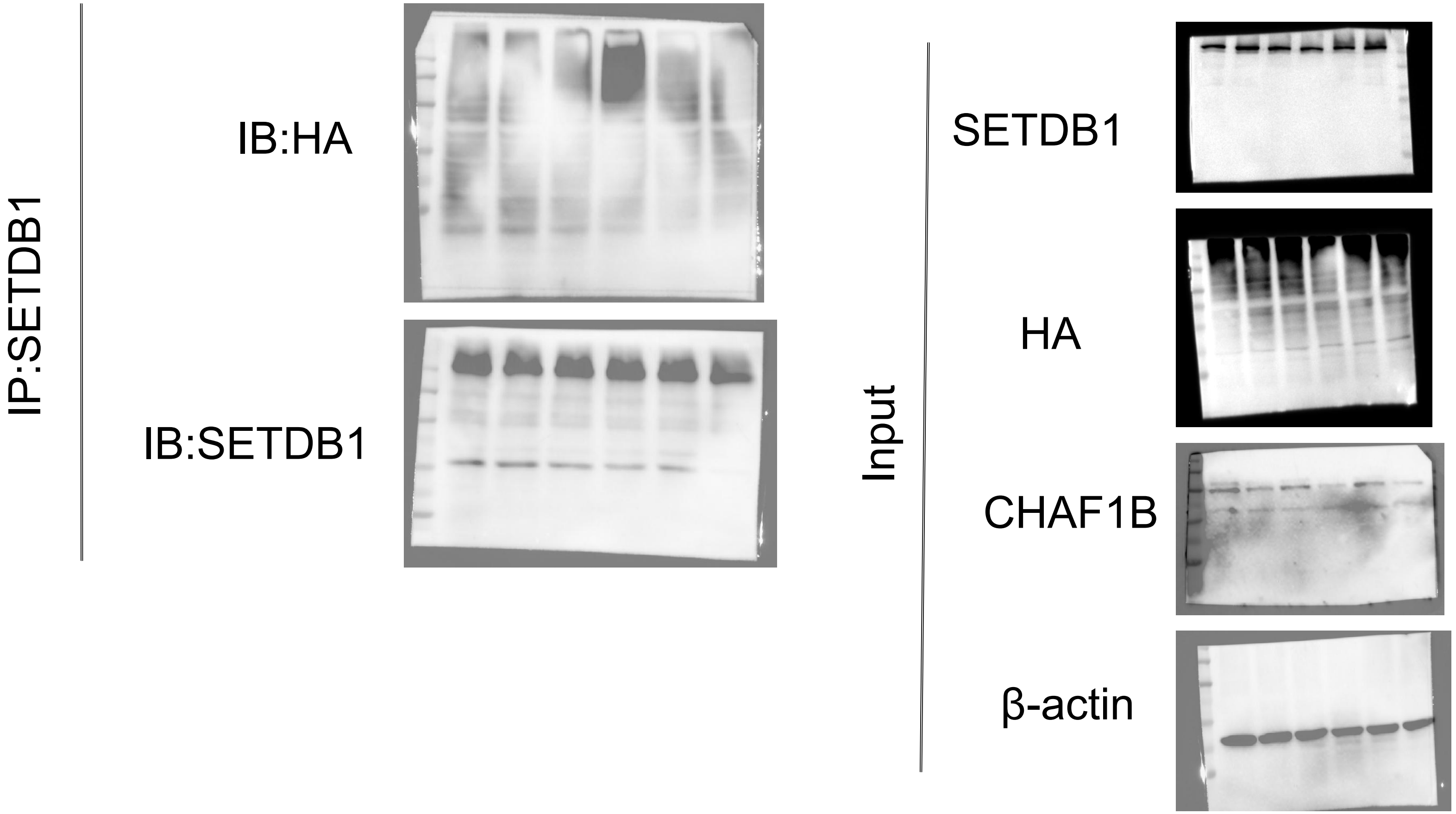

Fig S11C

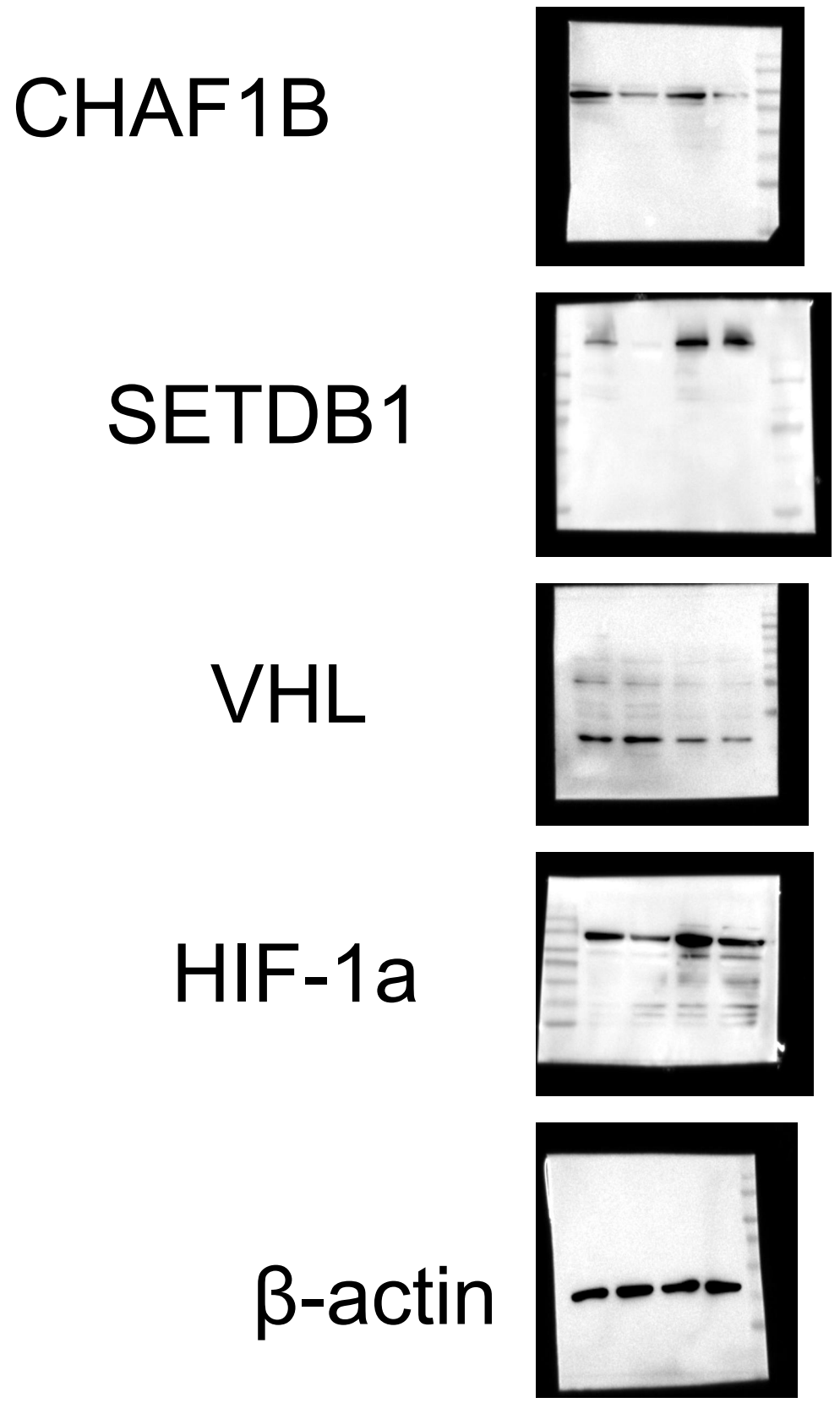

Fig S11D

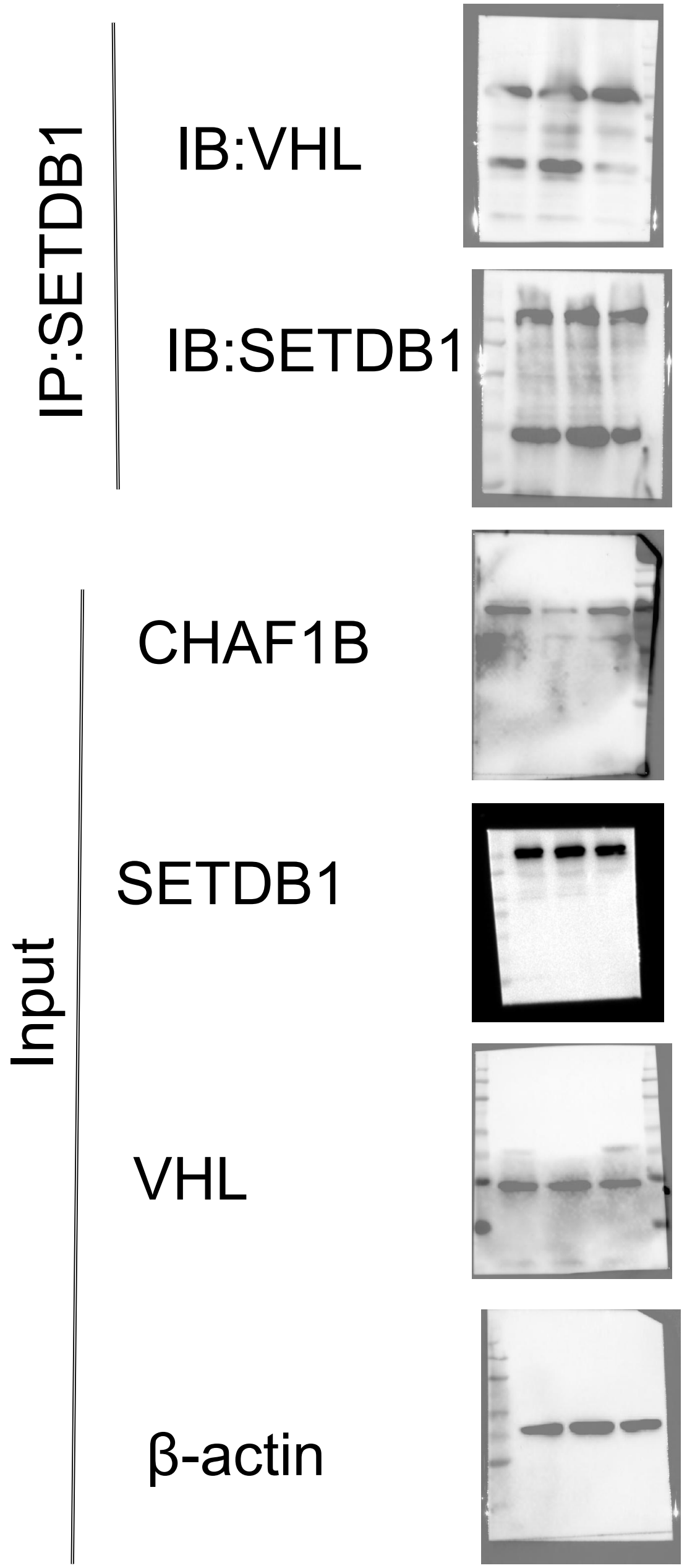

Fig S11E

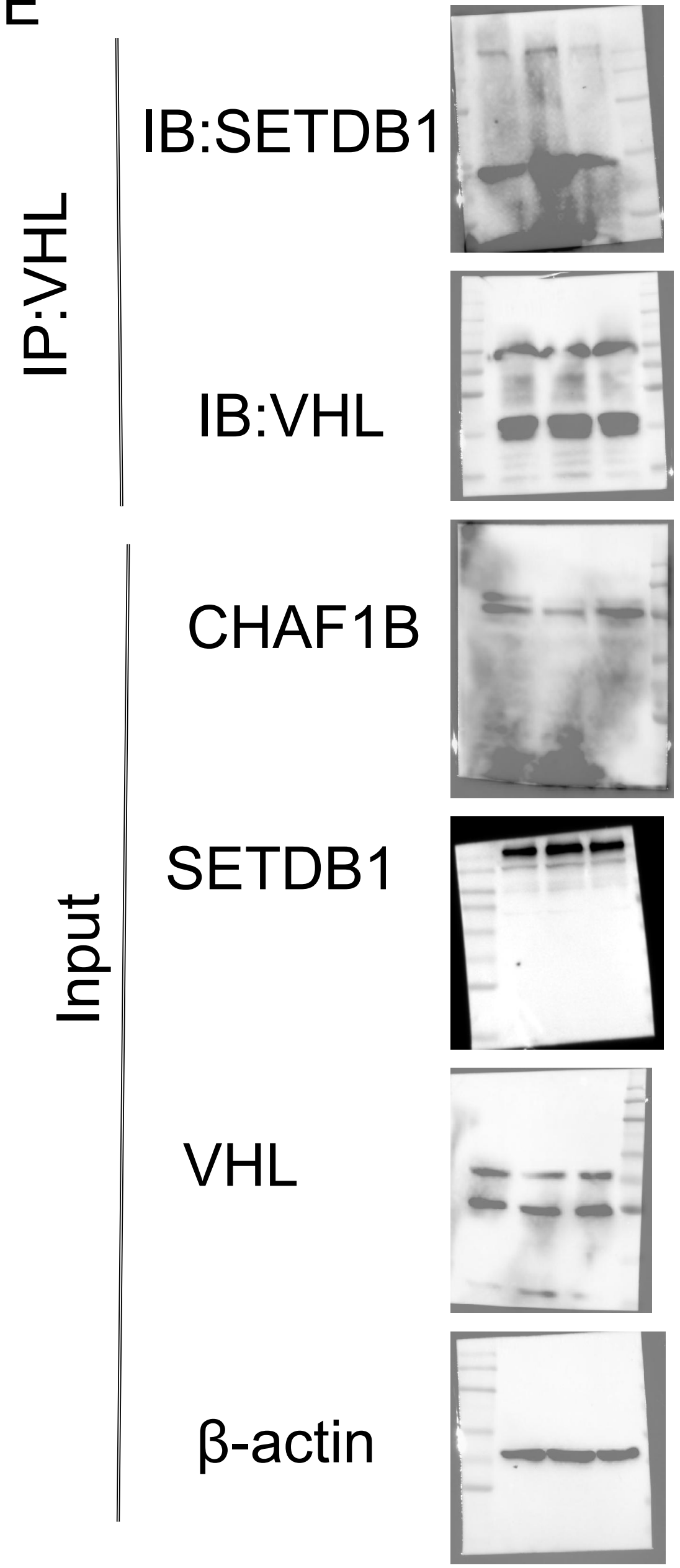

Fig S11F

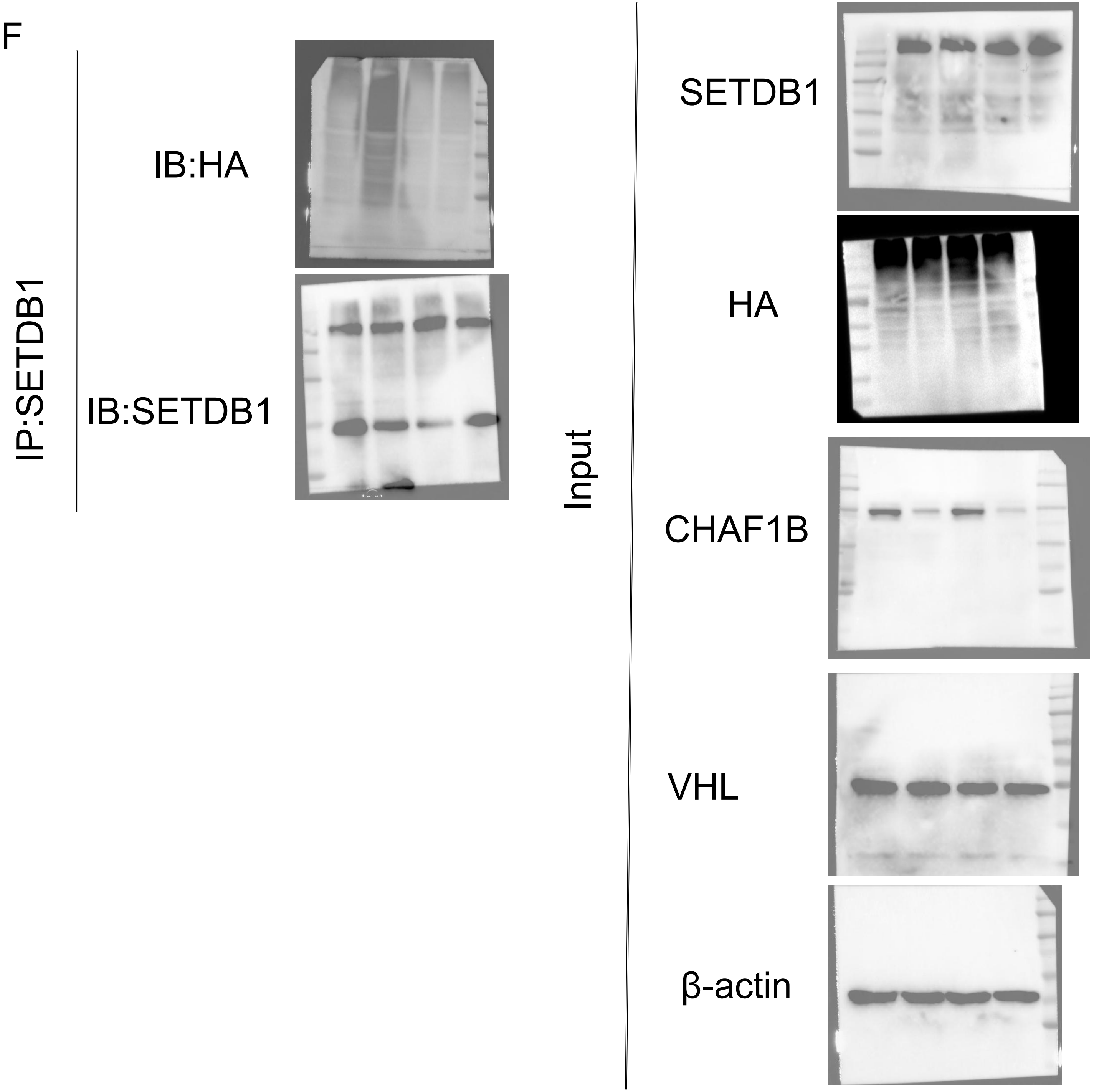

Fig. S12G

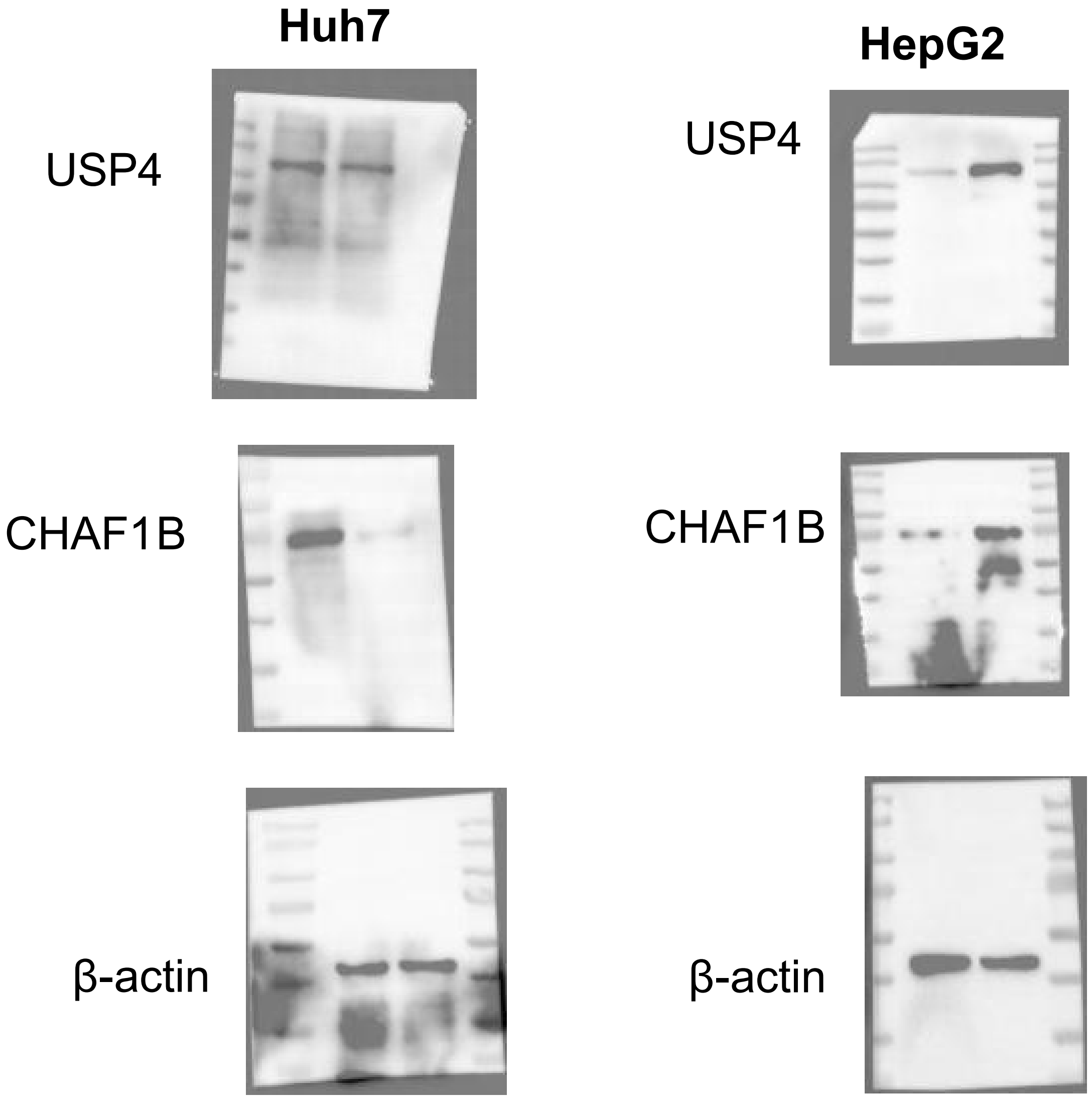

Fig. S12I

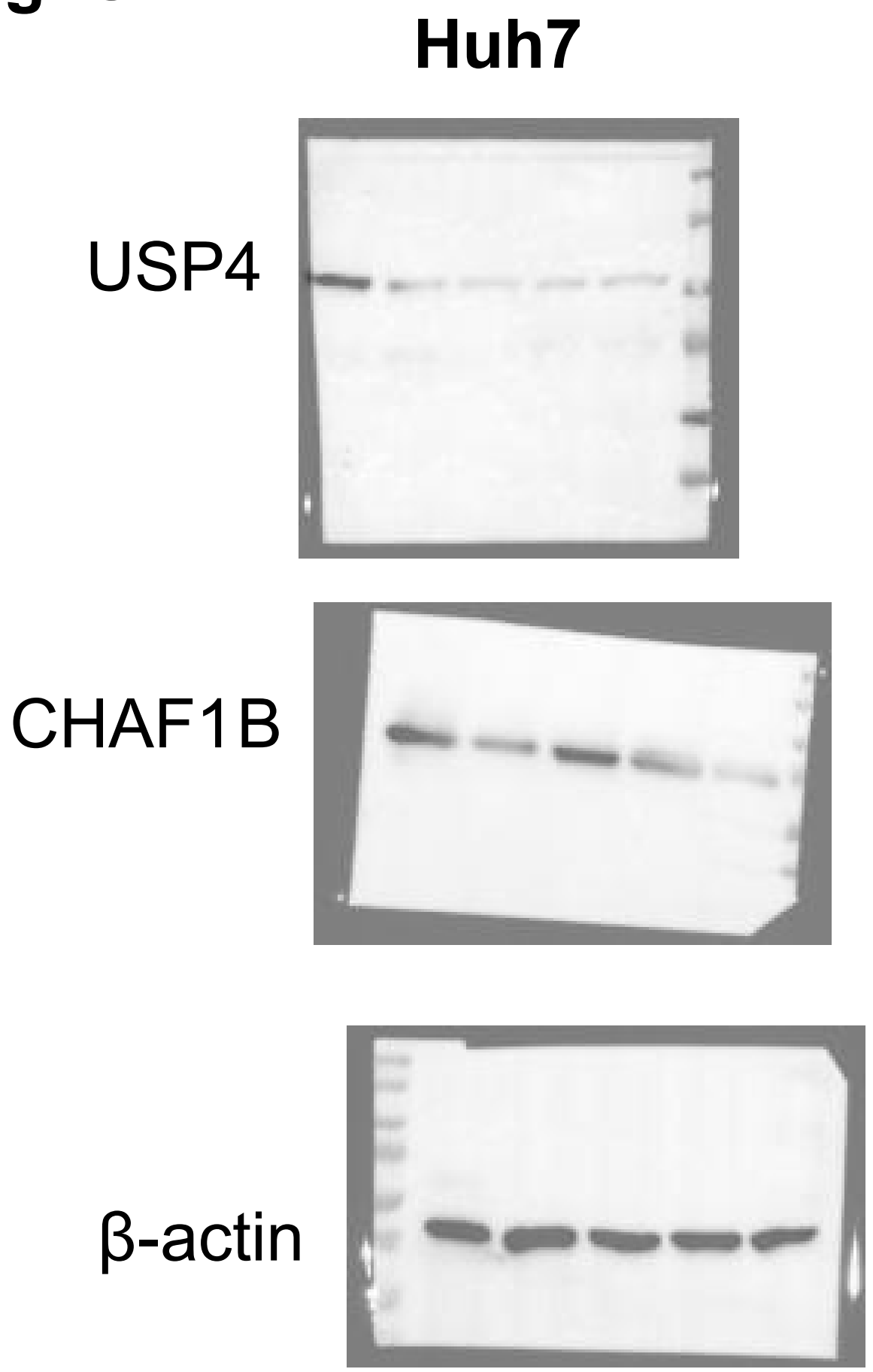

Fig. S12J

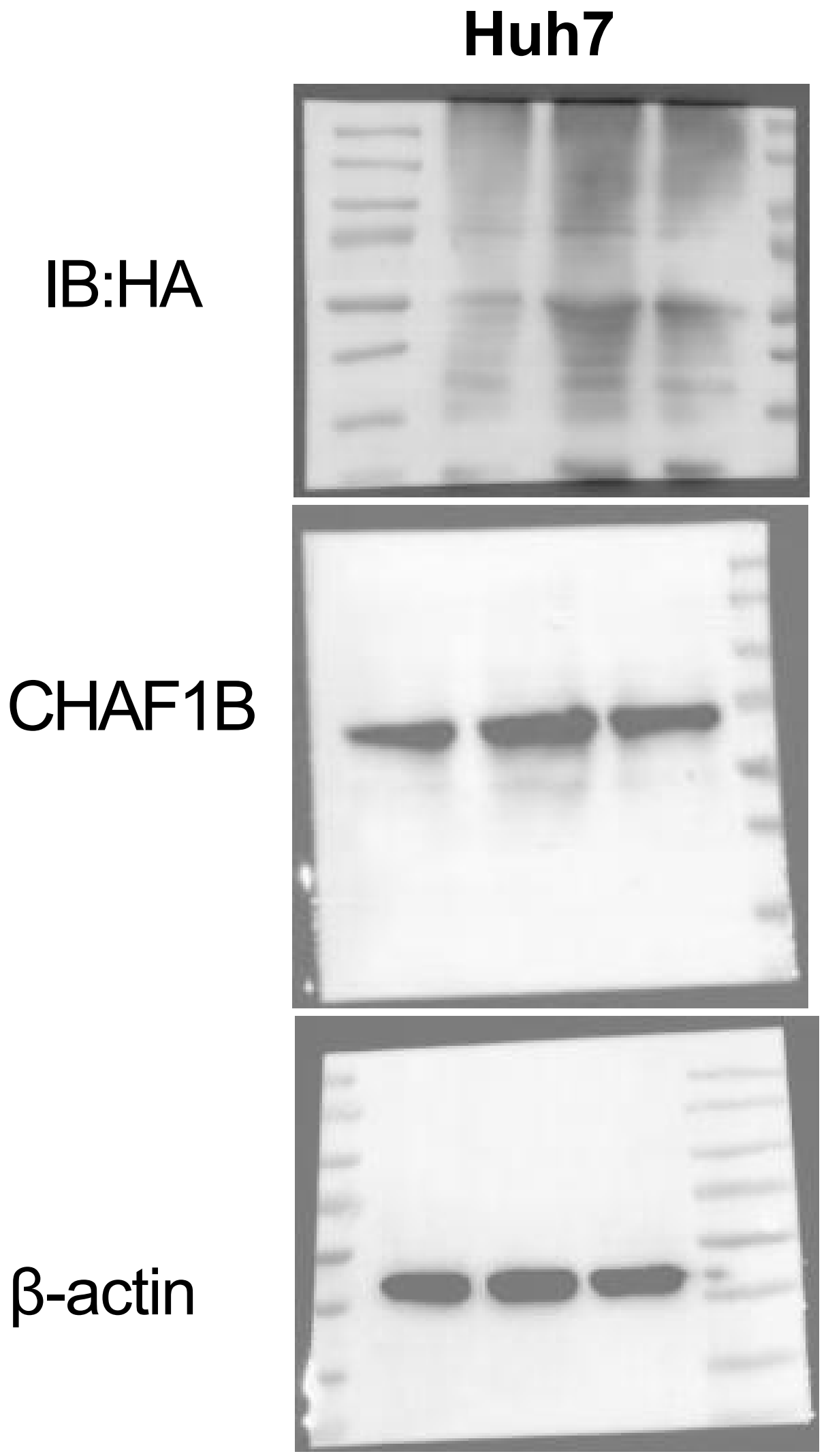

Fig. S12K

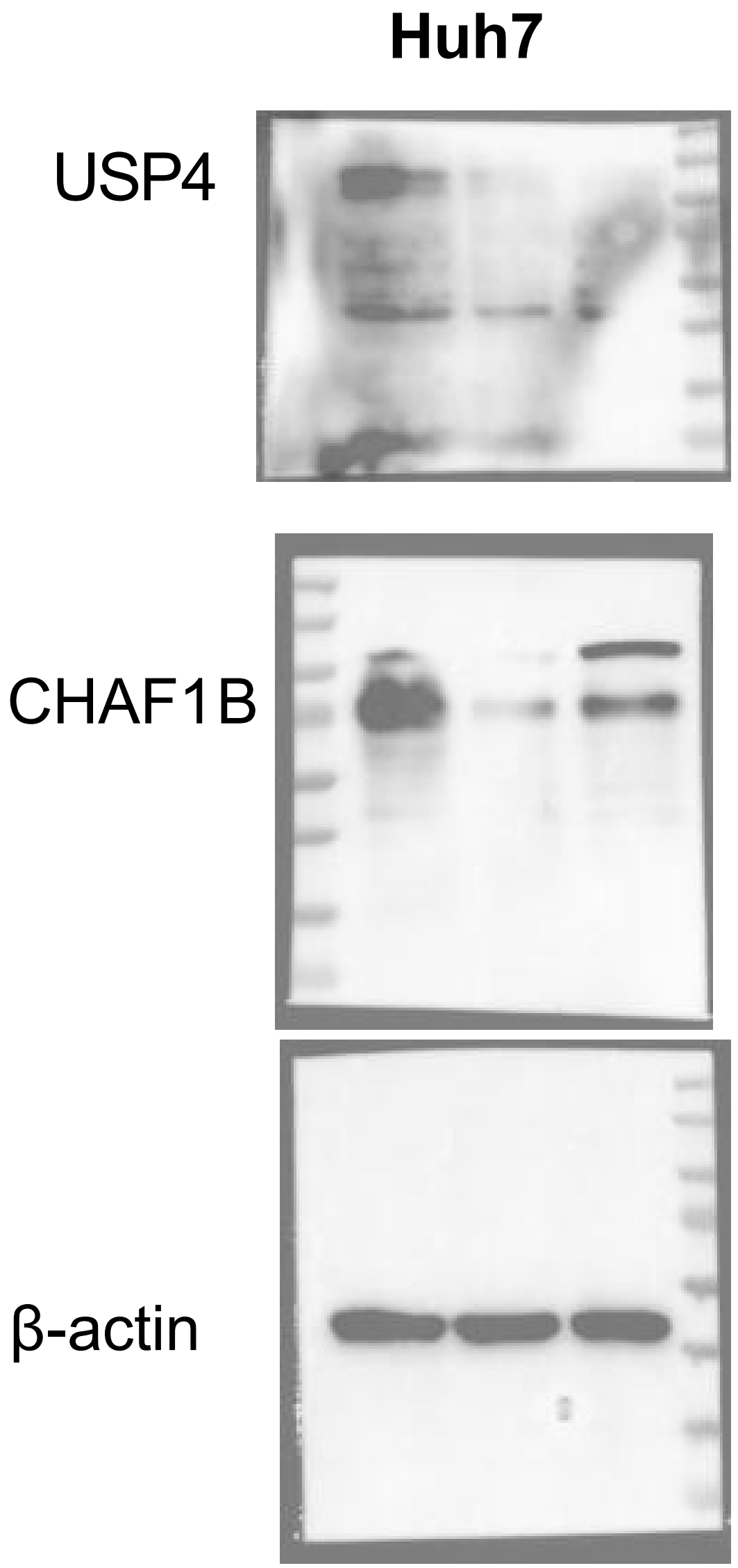

Fig. S15A

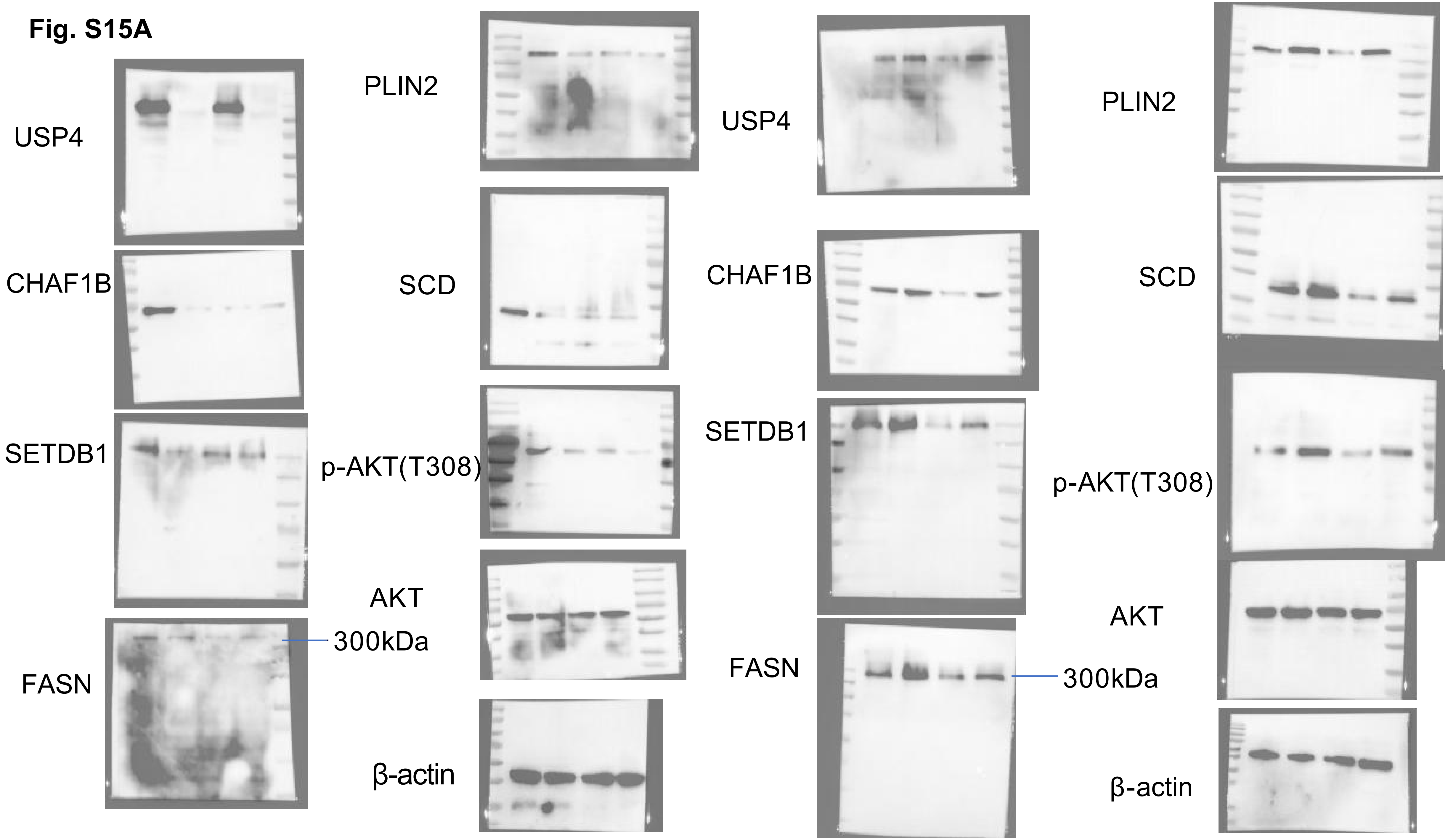
USP4
CHAF1B
SETDB1
FASN
300kDa
PLIN2
SCD
p-AKT(T308)
AKT
β-actin
USP4
CHAF1B
SETDB1
FASN
300kDa
PLIN2
SCD
p-AKT(T308)
AKT
β-actin
