# Supplementary material for: USP4‐Dependent CHAF1B Stabilization Regulates Distinct SETDB1 Ubiquitin States Linked to AKT T308 Signaling and Lipogenic Remodeling in HCC
Source: Adv Sci (Weinh). 2026 Sep 29:e78039. Online ahead of print. doi: 10.1002/advs.78039 (PMC13624420; doi:10.1002/advs.78039)
Supplement: Supplementary file 3 [file ADVS-9999-e78039-s004.docx]

**Fig. S1 Multi-cohort discovery and validation identify CHAF1B as a candidate regulator of HCC**

**(A)** Volcano plots from five GEO cohorts (GSE46408, GSE60502, GSE67764, GSE147888, GSE207435) showing genes upregulated in HCC relative to adjacent liver. **(B)** Venn diagram intersecting the five cohorts and defining a 44-gene consensus tumor-high module. **(C)** Cross-platform validation in TCGA and TCPA selecting 11 genes that are tumor-high and associated with poor prognosis. **(D)** Correlation analyses showing positive associations between CHAF1B and PCNA/MMP9 in the TCGA dataset. **(E)** Spatial transcriptomics with cell–cell communication analysis demonstrating higher CHAF1B in malignant regions versus adjacent liver and linkage to malignant cell activity. **(F)** Western blotting and RT-qPCR analyses of 24 paired freshly frozen HCC and adjacent non-tumor liver tissues confirmed higher CHAF1B expression in tumors. **(G)** Representative immunohistochemistry from three paired cases showing strong CHAF1B staining in tumors (T) compared with adjacent liver (N). For the immunoblot panels with densitometric values shown below the lanes, all protein signals were independently normalized to β-actin, and the corresponding control group within each cell line was set to 1.0. ****, P < 0.0001; ***, P < 0.001; **, P < 0.01. For (F), n = 24 paired HCC and adjacent non-tumor tissues, and paired two-sided Student’s t-tests were used; (G) shows representative IHC from n = 3 paired cases. Pearson correlation analysis was used for the TCGA analyses in (D).

**Fig. S2 Baseline CHAF1B expression, associated cellular phenotypes, and validation of CHAF1B gain- and loss-of-function models in HCC cells**

**(A)** Western blot analysis of CHAF1B, SETDB1, lipogenic proteins, and AKT signaling proteins in the indicated HCC cell lines. Relative band intensities are shown below the corresponding lanes. **(B)** RT–qPCR analysis of baseline CHAF1B mRNA expression across HCC cell lines. **(C)** Representative Nile Red staining showing lipid-droplet accumulation in the indicated cell lines. **(D)** Representative EdU incorporation assays and quantification of proliferative activity. **(E)** Representative colony formation assays comparing clonogenic capacity among HCC cell lines. **(F)** Western blot validation of CHAF1B knockdown in Huh7 and SK-Hep1 cells and CHAF1B overexpression in HepG2 and MHCC97H cells. **(G)** RT–qPCR validation of CHAF1B knockdown and overexpression in the indicated cell models. For the immunoblot panels with densitometric values shown below the lanes, all protein signals, including phosphorylated and total proteins, were independently normalized to β-actin, and the corresponding control group within each cell line was set to 1.0. *P < 0.05; **P < 0.01; ***P < 0.001; ****P < 0.0001. Quantitative analyses in (B,D,G) were performed in three independent experiments (n = 3) and are presented as mean ± SD. Comparisons among the five cell lines in (B,D) were analyzed using one-way ANOVA followed by Tukey’s multiple-comparisons test, whereas the two-group comparisons in (G) used unpaired two-sided Student’s t-tests.

**Fig. S3 Untargeted metabolomics reveals coordinated lipid remodeling upon CHAF1B overexpression**

**(A)** Volcano plots for G2_OE vs G2_vec in ESI− (left) and ESI+ (right). Each dot is a metabolite; x-axis: log2(FC), y-axis: −log10(P). Color denotes up-/down-regulated calls; dot size encodes VIP. Thresholds: VIP > 1.0, |FC| ≥ 1.2 (or ≤ 0.833), P < 0.05. **(B)** Chord diagrams showing correlated, differentially abundant metabolites. Lipid classes (oxylipins/eicosanoids, PC/LPC/PE including ether species, sphingomyelins, acylcarnitines) cluster as cooperative modules. **(C)** Correlogram of differential metabolites highlighting network-level concordance among oxylipins, glycerophospholipids, and sphingomyelins, supporting lipid-pathway remodeling in CHAF1B-overexpressing cells. Metabolomic profiling used n = 3 biological replicates per group. Differential metabolites in (A) were identified using two-sided Student’s t-tests together with the stated VIP and fold-change thresholds. Pairwise correlations in (B,C) were assessed using Pearson’s correlation coefficient with two-sided correlation tests; P < 0.05 was considered significant.

**Fig. S4 PI3K inhibition attenuates CHAF1B-associated AKT signaling and malignant phenotypes**

**(A**) Western blotting analysis in HepG2 and MHCC97H cells shows that LY294002 suppresses CHAF1B-induced phosphorylation of AKT at T308 and of GSK-3β at S9, with comparatively limited changes in total AKT and total GSK-3β. **(B)** Transwell migration assays indicate that CHAF1B overexpression enhances cell motility in MHCC97H, which is reduced by LY294002 treatment. **(C)** Phase-contrast imaging shows that CHAF1B promotes a spindle-like, mesenchymal morphology in MHCC97H, whereas LY294002 restores a more epithelial appearance. **(D)** 3D spheroid assays demonstrate accelerated spheroid growth and increased Ki-67 positivity with CHAF1B overexpression, both of which are attenuated by LY294002. For the immunoblot panels with densitometric values shown below the lanes, all protein signals, including phosphorylated and total proteins, were independently normalized to β-actin, and the corresponding control group within each cell line was set to 1.0. ****P < 0.0001. For the quantitative spheroid analysis in (D), n = 3 independently cultured spheroids per group. Data are presented as mean ± SD and were analyzed using one-way ANOVA followed by Tukey’s multiple-comparisons test.

**Fig. S5 SREBP1C knockdown attenuates CHAF1B-associated lipogenic remodeling and malignant phenotypes in HepG2 cells**

**(A)** Western blot analysis of lipogenic proteins and mTORC1-associated readouts in control, CHAF1B-overexpressing, and CHAF1B-overexpressing/siSREBP1C HepG2 cells. Relative band intensities are shown below the corresponding lanes. **(B)** Heatmap of FASN, ACACA, and SCD mRNA expression in the indicated groups. **(C)** Nile Red staining showing that SREBP1C knockdown reduces CHAF1B-induced lipid-droplet accumulation. **(D)** EdU incorporation assay and quantification showing that SREBP1C knockdown partially attenuates CHAF1B-associated cell proliferation. **(E)** Representative colony formation assays showing reduced clonogenic growth after SREBP1C knockdown in CHAF1B-overexpressing cells. **(F)** Representative Transwell migration assays showing that SREBP1C knockdown attenuates CHAF1B-associated cell migration. For the immunoblot panels with densitometric values shown below the lanes, all protein signals, including phosphorylated and total proteins, were independently normalized to β-actin, and the corresponding control group within each cell line was set to 1.0. **P < 0.01; ****P < 0.0001. The EdU quantification in (D) was performed in three independent experiments (n = 3). Data are presented as mean ± SD and were analyzed using one-way ANOVA followed by Tukey’s multiple-comparisons test.

**Fig. S6 CHAF1B regulates SETDB1 protein abundance in HCC**

**(A)** Spatial transcriptomic analyses showing elevated SETDB1 expression in malignant compared to adjacent non-malignant liver tissue, with cell–cell communication analysis highlighting its association with malignant hepatocyte activity. **(B&C)** Western blotting and qPCR analyses demonstrating that CHAF1B regulates SETDB1 protein, with comparatively limited effects on mRNA levels. **(D)** Immunoblotting of xenograft tumors showing increased SETDB1 abundance, AKT T308 phosphorylation, and GSK3β S9 phosphorylation in CHAF1B-overexpressing tumors, with comparatively limited effects on AKT S473 phosphorylation. For the immunoblot panels with densitometric values shown below the lanes, all protein signals, including phosphorylated and total proteins, were independently normalized to β-actin, and the corresponding control group within each cell line was set to 1.0. ns, not significant. For the RT–qPCR analyses in (C), n = 3 independent experiments and data are presented as mean ± SD; two-group comparisons used unpaired two-sided Student’s t-tests. The xenograft immunoblots in (D) show individual tumors from n = 5 mice per group.

**Fig. S7 The methyltransferase activity of SETDB1 contributes to CHAF1B-associated lipogenic and malignant phenotypes in HCC cells.**

**(A)** Western blot analysis of CHAF1B, FLAG-SETDB1, and the lipogenic proteins FASN, PLIN2, and SCD. Relative band intensities are shown below the corresponding lanes after normalization to β-actin, with the shNC group set to 1.0. **(B)** RT–qPCR analysis of FASN, ACACA, and SCD mRNA expression in the indicated groups. **(C)** Representative Nile Red staining showing lipid-droplet accumulation and EdU incorporation assays assessing cell proliferation. EdU-positive cells were quantified as a percentage of total DAPI-positive cells. **(D)** Representative Transwell migration and colony formation assays showing the effects of SETDB1-WT and SETDB1-H1224K on the malignant phenotypes of CHAF1B-depleted Huh7 cells. **(E)** Representative images of three-dimensional spheroids at Days 3, 5, and 7. Spheroid volume at Day 7 was quantified in the indicated groups. SETDB1-WT restored CHAF1B depletion-associated lipogenic and malignant phenotypes, whereas SETDB1-H1224K showed substantially reduced rescue activity. Data are presented as mean ± SD. For the spheroid analysis, three independently cultured spheroids from separate wells were analyzed per group, and each data point represents one spheroid. For the immunoblot panels with densitometric values shown below the lanes, all protein signals were independently normalized to β-actin, and the corresponding control group within each cell line was set to 1.0. ns, not significant; **P < 0.01; ****P < 0.0001. Quantitative cell-based analyses were performed in three independent experiments (n = 3), and the spheroid analysis in (E) used n = 3 independently cultured spheroids per group. Data are presented as mean ± SD. Comparisons among the three groups were analyzed using one-way ANOVA followed by Tukey’s multiple-comparisons test.

**Fig. S8 K63-linked ubiquitination and CRM1-dependent export support SETDB1 persistence and cytoplasmic redistribution.**

**(A)** CHX-chase analysis showing that LMB or HA-Ub-K63R accelerates SETDB1 loss in CHAF1B-overexpressing cells. Relative SETDB1 levels were normalized to β-actin and the corresponding 0-h group. **(B)** SETDB1 ubiquitination assays using HA-Ub-WT, HA-Ub-K48, and HA-Ub-K63 in Huh7 cells. CHAF1B depletion reduced total and K63-linked SETDB1 ubiquitination, whereas K48-linked ubiquitination showed no reproducible increase. **(C)** SETDB1 ubiquitination assays comparing HA-Ub-WT, HA-Ub-K48R, and HA-Ub-K63R in CHAF1B-overexpressing HEK-293T cells. HA-Ub-K63R more strongly attenuated CHAF1B-induced SETDB1 ubiquitination than HA-Ub-K48R. **(D)** Confocal imaging showing that SETDB1 re-expression partially restores cytoplasmic SETDB1 accumulation in CHAF1B-depleted cells, whereas HA-Ub-K63R impairs this redistribution. **(E)** Nuclear/cytoplasmic fractionation confirming increased cytoplasmic SETDB1 after SETDB1 re-expression in CHAF1B-depleted cells. **(F)** Nuclear/cytoplasmic fractionation showing that MG132 increases SETDB1 abundance under HA-Ub-K63R or LMB treatment but does not restore cytoplasmic SETDB1 accumulation. Lamin B1 and α-tubulin were used as nuclear and cytoplasmic markers, respectively. For immunoblot panels with relative band intensities shown below the corresponding lanes, whole-cell lysate signals were independently normalized to β-actin. Nuclear and cytoplasmic protein signals in the subcellular fractionation panels were normalized to Lamin B1 and α-tubulin, respectively. Within each cell line and experiment, the corresponding control group was set to 1.0. The CHX-chase analysis in (A) was generated from three independent experiments (n = 3).

**Fig. S9 CHAF1B regulation of SETDB1 and AKT T308-linked signaling is not strictly dependent on the tested ASF1A-binding interface**

**(A)** Schematic of CHAF1B domains (WD repeats; p150-interaction region) and the RR482/483AA mutation that disrupts ASF1A binding. **(B)** Immunoblotting in HEK-293T and HepG2 cells showing that CHAF1B ^RR482/483AA^ retains the ability to increase SETDB1 abundance and AKT T308 phosphorylation, with comparatively limited changes in AKT S473 phosphorylation. **(C)** Ubiquitination assays showing that CHAF1B ^RR482/483AA^ retains CHAF1B-mediated ubiquitination of SETDB1. For the immunoblot panels with densitometric values shown below the lanes, all protein signals, including phosphorylated and total proteins, were independently normalized to β-actin, and the corresponding control group within each cell line was set to 1.0.

**Fig. S10 CHAF1B facilitates the UHRF1–SETDB1 association and** **supports UHRF1-promoted K63-dependent ubiquitination of SETDB1**

**(A)** UHRF1 co-immunoprecipitation showing that CHAF1B depletion reduces the endogenous UHRF1–SETDB1 association in Huh7 cells. IgG was used as a negative control. **(B)** Endogenous Co-IP assays examining the association of AKT and p-AKT(T308) with the CHAF1B–UHRF1–SETDB1 regulatory module. CHAF1B, SETDB1, and UHRF1 were enriched in their corresponding immunoprecipitates, whereas AKT and p-AKT(T308) showed no specific enrichment above the corresponding IgG controls under the conditions tested. **(C)** CHAF1B depletion and rescue analysis showing that CHAF1B-WT restores the UHRF1–SETDB1 association and SETDB1 abundance, whereas CHAF1B-ΔWD40 shows a comparatively weaker rescue. AKT T308 phosphorylation is shown as a downstream signaling readout, while total AKT and AKT S473 phosphorylation show comparatively limited changes. **(D)** SETDB1 ubiquitination assays using HA-Ub-WT and linkage-restricted HA-Ub-K48 and HA-Ub-K63 constructs. UHRF1 overexpression-associated restoration was most evident with the linkage-restricted HA-Ub-K63 construct. **(E)** SETDB1 ubiquitination assays showing that HA-Ub-K63R markedly attenuates the UHRF1 overexpression-associated restoration of SETDB1-associated ubiquitination.

**Fig. S11 CHAF1B depletion promotes VHL-dependent K11-associated degradative ubiquitination of SETDB1.**

**(A)** SETDB1 ubiquitin-linkage profiling in HEK-293T cells expressing shNC or shCHAF1B together with HA-Ub-WT or the indicated linkage-restricted HA-Ub constructs. **(B)** SETDB1 ubiquitination assays in Huh7 cells comparing HA-Ub-WT, linkage-restricted HA-Ub-K11, and HA-Ub-K11R in control and CHAF1B-depleted cells. **(C)** Immunoblot analysis of CHAF1B, SETDB1, VHL, and HIF-1α in Huh7 cells expressing shNC or shCHAF1B with or without siVHL. Relative band intensities are shown below the corresponding lanes after normalization to β-actin, with the corresponding control group set to 1.00. **(D)** Endogenous SETDB1 immunoprecipitation followed by VHL immunoblotting showing increased SETDB1–VHL association after CHAF1B depletion and attenuation of this association after CHAF1B re-expression. **(E)** Reciprocal VHL immunoprecipitation followed by SETDB1 immunoblotting confirming increased SETDB1–VHL association after CHAF1B depletion and attenuation following CHAF1B re-expression. **(F)** SETDB1 ubiquitination assays using linkage-restricted HA-Ub-K11 in Huh7 cells expressing shNC or shCHAF1B with or without siVHL. VHL depletion markedly reduced the increase in K11-associated SETDB1 ubiquitination following CHAF1B depletion.

**Fig. S12 Functional and mechanistic validation of USP4 as an upstream regulator of CHAF1B**

**(A)** IP–MS identification of USP4 in CHAF1B-containing complexes. **(B–E)** Functional assays showing that USP4 knockdown suppresses, whereas USP4 overexpression promotes, HCC cell proliferation, clonogenic growth, migration, and 3D spheroid expansion. **(F)** Nile Red staining indicating reduced lipid-droplet accumulation after USP4 silencing and increased accumulation after USP4 overexpression. **(G)** Immunoblotting in Huh7 and HepG2 showing that USP4 depletion decreases, while USP4 overexpression increases, CHAF1B protein abundance. **(H)** RT-qPCR showing that USP4 modulation does not materially change CHAF1B mRNA levels. **(I)** Proteostasis assays showing that MG132, but not chloroquine (CQ) or 3-MA, restores CHAF1B protein abundance reduced by USP4 knockdown, consistent with a proteasome-dependent mechanism. **(J, K)** Ubiquitination assays using the K48R ubiquitin mutant supporting a predominant contribution of K48-linked ubiquitination to USP4-dependent regulation of CHAF1B. **(L)** Rescue experiments showing that CHAF1B re-expression in USP4-deficient Huh7 cells restores migration. For the immunoblot panels with densitometric values shown below the lanes, all protein signals were independently normalized to β-actin, and the corresponding control group within each cell line was set to 1.0. ns, not significant. ****P < 0.0001; ***P < 0.001; **P < 0.01. Quantitative cell-based analyses in (B–E,H,L) were performed in three independent experiments (n = 3) and are presented as mean ± SD. Two-group comparisons in (B–E,H) were analyzed using unpaired two-sided Student’s t-tests, whereas the three-group rescue comparison in (L) was analyzed using one-way ANOVA followed by Tukey’s multiple-comparisons test.

**Fig. S13 Clinical association of USP4 with CHAF1B protein expression and unfavorable clinical outcome in human HCC**

**(A)** Overview of USP4 immunohistochemical staining in the HCC tissue microarray containing tumor (T) and adjacent non-tumor (N) tissues. **(B)** Representative USP4 staining in adjacent non-tumor liver and HCC tissues. **(C)** Quantitative comparison of USP4 IHC scores between HCC and adjacent non-tumor tissues. **(D)** Proportions of patients with and without metastasis in the USP4-low and USP4-high groups. **(E)** Pearson correlation analysis of USP4 and CHAF1B IHC scores in the same HCC tissue cohort. Each point represents one patient specimen. **(F)** Kaplan–Meier analysis of overall survival according to USP4 expression and combined CHAF1B/SETDB1/USP4 expression status. IHC scores were calculated by multiplying staining intensity by the percentage of positively stained tumor cells, yielding a score ranging from 0 to 300. Expression groups were defined using the median IHC score of the corresponding protein, as described in the Methods. *P < 0.05; ***P < 0.001. The tissue microarray cohort comprised n = 158 HCC cases. Paired tumor-versus-adjacent comparisons in (C) were analyzed using paired two-sided Student’s t-tests; categorical variables in (D) were analyzed using the χ² test or Fisher’s exact test, as appropriate; the correlation in (E) was analyzed using Pearson correlation; and survival curves in (F) were compared using two-sided log-rank tests.

**Fig. S14 ASD attenuates malignant phenotypes in vitro and CHAF1B provides partial rescue**

**(A)** EdU assays in Huh7 showing a dose-dependent reduction in DNA synthesis after ASD treatment. **(B)** Colony-formation assays showing decreased clonogenic growth with ASD. **(C)** Transwell assays demonstrating reduced migration with ASD. **(D)** 3D spheroid assays showing inhibited spheroid growth with ASD. **(E)** Rescue experiment showing that ectopic CHAF1B restores migration suppressed by ASD. ****P < 0.0001, **P < 0.01. Quantitative analyses were performed in three independent experiments (n = 3) and are presented as mean ± SD. Comparisons among three or more groups were analyzed using one-way ANOVA followed by Tukey’s multiple-comparisons test.

**Fig. S15 Genetic epistasis and USP4 rescue support a USP4-dependent component of ASD activity**

**(A)** Western blot analysis showing that USP4 depletion attenuates the additional effects of ASD on CHAF1B, SETDB1, AKT T308 phosphorylation, and lipogenic proteins, whereas USP4 overexpression partially restores these molecular readouts after ASD treatment. **(B)** EdU incorporation assays showing that USP4 depletion reduces the additional antiproliferative effect of ASD, whereas USP4 overexpression partially restores cell proliferation. **(C)** Colony formation assays showing that USP4 depletion attenuates ASD-induced suppression of clonogenic growth, whereas USP4 overexpression partially restores colony formation. **(D)** Transwell migration assays showing that USP4 depletion reduces the additional inhibitory effect of ASD, whereas USP4 overexpression partially restores cell migration. For the immunoblot panels with densitometric values shown below the lanes, all protein signals, including phosphorylated and total proteins, were independently normalized to β-actin, and the corresponding control group within each cell line was set to 1.0. ns, not significant; **P < 0.01; ***P < 0.001; ****P < 0.0001. Quantitative analyses in (B–D) were performed in three independent experiments (n = 3) and are presented as mean ± SD. Comparisons among the indicated groups were analyzed using one-way ANOVA followed by Tukey’s multiple-comparisons test.

**Fig. S16 Proposed model of the USP4–CHAF1B–SETDB1 regulatory network in HCC.**

Working model integrating distinct ubiquitin-dependent regulatory states, downstream signaling, and pharmacologic perturbation. (1) USP4 limits K48-linked polyubiquitination of CHAF1B, thereby reducing proteasome-dependent CHAF1B degradation and supporting CHAF1B protein stability. Akebia saponin D (ASD) is shown as a pharmacologic probe that perturbs the USP4–CHAF1B axis. (2) Higher CHAF1B abundance facilitates the UHRF1–SETDB1 regulatory module. UHRF1, a candidate CHAF1B-associated E3 ligase, promotes predominantly K63-dependent ubiquitination of SETDB1, supporting a non-degradative state associated with CRM1/exportin-1-dependent cytoplasmic redistribution. Leptomycin B (LMB) inhibits CRM1-dependent nuclear export. In contrast, CHAF1B depletion enhances the association of SETDB1 with VHL, the substrate-recognition component of the CRL2^VHL E3 ubiquitin ligase complex, and favors a VHL-dependent, predominantly K11-associated degradative ubiquitin state linked to proteasome-dependent SETDB1 loss. (3) Cytoplasmic SETDB1 and its methyltransferase activity contribute to AKT methylation and AKT T308-linked signaling. These events are associated with increased mTORC1-related readouts, SREBP1C nuclear localization, induction of lipogenic genes and proteins, lipid remodeling, and aggressive HCC phenotypes. LY294002 inhibits PI3K-dependent signaling. The schematic was created with BioRender.com under a BioRender publication license (Agreement number: PU2A2FBWEI).

**Fig. S17 Longitudinal body-weight monitoring across mouse experiments.**

(A) HepG2 subcutaneous xenograft model corresponding to Fig. 1I (n = 5 mice per group). (B) Orthotopic Huh7 liver-tumor model corresponding to Fig. 1J (n = 3 mice per group). (C) c-Myc/myr-AKT Sleeping Beauty liver-tumor model with vector control or CHAF1B co-expression, corresponding to Fig. 1K–L (n = 3 mice per group). (D) Sleeping Beauty liver-tumor model treated with vehicle, ASD, or ASD combined with CHAF1B overexpression, corresponding to Fig. 8L (n = 3 mice per group). (E) Subcutaneous Huh7 xenograft model treated with vehicle, ASD, or ASD combined with CHAF1B overexpression, corresponding to Fig. 8M (n = 5 mice per group). In (E), the first measurement represents the pre-treatment baseline. Data are presented as mean ± SD. Individual-animal measurements are provided in Supplementary Data S1.

**Supplementary Table S1. Antibodies used in this study**

| **Antibody** | **Company** | **Cat. Number** |
| --- | --- | --- |
| CHAF1B | ABclonal | A5073 |
| SETDB1 | Proteintech | 11231-1-AP |
| HA | Proteintech | 51064-2-AP |
| Ub | Santa Cruz | sc-53509 |
| FLAG | ABclonal | AE005 |
| His | Proteintech | 66005-1-Ig |
| β-actin | Proteintech | 66009-1-Ig |
| E-cadherin | Cell Signaling Technology | #3195 |
| N-cadherin | Cell Signaling Technology | #13116 |
| Vimentin | Proteintech | 10366-1-AP |
| p-AKT(S473) | Cell Signaling Technology | #4060 |
| p-AKT(T308) | Cell Signaling Technology | #99275 |
| AKT | Cell Signaling Technology | #9272 |
| p-GSK3β(S9) | Cell Signaling Technology | #5558 |
| GSK3β | Cell Signaling Technology | #9315 |
| α-Tubulin | Proteintech | 80762-1-RR |
| Lamin B1 | Abcam | ab229025 |
| UHRF1 | Proteintech | 21402-1-AP |
| Ki-67 | Abcam | ab15580 |
| FASN | Cell Signaling Technology | #3180 |
| p-S6K | Cell Signaling Technology | #5364 |
| S6K | Cell Signaling Technology | #2217 |
| ACACA | Abcam | ab45174 |
| SCD | Abcam | ab19826 |
| PLIN2 | Abcam | ab168348 |
| p-4E-BP1 | Santa Cruz | sc293124 |
| 4E-BP1 | Santa Cruz | sc9977 |
| USP4 | Proteintech | 66822-1-Ig |
| pan-methyl-lysine antibody | zenbio | 350024 |
| IgG | Proteintech | 30000-0-AP |
| SREBP1C | Santa Cruz | sc-365513 |
| VHL | Proteintech | 24756-1-AP |
| HIF-1α | Abcam | ab179483 |

**Supplementary Table S2. shRNA, siRNA, and sgRNA sequences used for downstream validation**

| **Oligonucleotide** | **Strand/construct** | **Sequence (5′–3′)** |
| --- | --- | --- |
| **shCHAF1B** | Forward oligo | CCGGGCGAGTATACAGTATACAGAACTC |
|  | Reverse oligo | GAGTTCTGTATACTGTATACTCGCTTTTTT |
| **siSREBF1 (targeting**  **SREBP1C)** | Sense | GUGACUUCCCUGGCCUAUUUGdTdT |
|  | Antisense | CAAAUAGGCCAGGGAAGUCACdTdT |
| **siVHL** | Sense | UAUCACACUGCCAGUGUAUACdTdT |
|  | Antisense | GUAUACACUGGCAGUGUGAUAdTdT |
| **sgUSP4** | sgUSP4-1 | AAATGGCAACTCAGCACAT |
|  | sgUSP4-2 | AGCTGGGACATGTACAATG |

For the focused CRISPR-based DUB screen, two independent sgRNAs were used for each target gene as a pooled sgRNA pair. The sgUSP4-1 and sgUSP4-2 sequences used for downstream validation are listed above.

**Supplementary Table S3. Primer sequences used for RT-qPCR**

| **Gene** | **Forward primer (5′–3′)** | **Reverse primer (5′–3′)** |
| --- | --- | --- |
| **FASN** | CCTGGCTGCCTACTACATCG | CACATTTCAAAGGCCACGCA |
| **ACACA** | TATTGCGGCCAATGTCTTTGC | CACTGGAGTGATAGACTCAACCA |
| **SCD** | TCTAGCTCCTATACCACCACCA | TCGTCTCCAACTTATCTCCTCC |
| **CHAF1B** | CCAATGAGAGCGGAAATGAGA | ATCTAGACCAACACCAATTAGA |
| **SETDB1** | TCTTTCGAGTTCTTTACTTCTC | CGACTGGCTGTTCCTGGCTTTTA |
| **ACTB** | AGAACTGGCCCTTCTTGGAGG | GTTTTTATGTTCCTCTATGGG |
